# Supplementary material for: Remote blood pressure monitoring and behavioral intensification for stroke: A randomized controlled feasibility trial
Source: PLoS One. 2020 Mar 11;15(3):e0229483. doi: 10.1371/journal.pone.0229483 (PMC7065804; doi:10.1371/journal.pone.0229483)
Supplement: S2 Checklist — (PDF) [file pone.0229483.s002.pdf]

CONSORT 2010 checklist of information to include when reporting a pilot or feasibility randomized trial in a journal or conference abstract

Item	Description	Reported on line number
Title	Identification of study as randomised pilot or feasibility trial	53
Authors *	Contact details for the corresponding author	29
Trial design	Description of pilot trial design (eg, parallel, cluster)	53
Methods		
Participants	Eligibility criteria for participants and the settings where the pilot trial was conducted	51
Interventions	Interventions intended for each group	54-58
Objective	Specific objectives of the pilot trial	51
Outcome	Prespecified assessment or measurement to address the pilot trial objectives**	59-62
Randomization	How participants were allocated to interventions	53
Blinding (masking)	Whether or not participants, care givers, and those assessing the outcomes were blinded to group assignment	53
Results		
Numbers randomized	Number of participants screened and randomised to each group for the pilot trial objectives**	63-64
Recruitment	Trial status†	
Numbers analysed	Number of participants analysed in each group for the pilot objectives**	63-64
Outcome	Results for the pilot objectives, including any expressions of uncertainty**	
Harms	Important adverse events or side effects	72-73
Conclusions	General interpretation of the results of pilot trial and their implications for the future definitive trial	74-76
Trial registration	Registration number for pilot trial and name of trial register	77
Funding	Source of funding for pilot trial	78

Citation: Eldridge SM, Chan CL, Campbell MJ, Bond CM, Hopewell S, Thabane L, et al. CONSORT 2010 statement: extension to randomised pilot and feasibility trials. BMJ. 2016;355.

**this item is specific to conference abstracts*

***Space permitting, list all pilot trial objectives and give the results for each. Otherwise, report those that are a priori agreed as the most important to the decision to proceed with the future definitive RCT.*

†For conference abstracts.
