# Supplementary material for: Remote blood pressure monitoring and behavioral intensification for stroke: A randomized controlled feasibility trial
Source: PLoS One. 2020 Mar 11;15(3):e0229483. doi: 10.1371/journal.pone.0229483 (PMC7065804; doi:10.1371/journal.pone.0229483)
Supplement: S1 Appendix — (PDF) [file pone.0229483.s003.pdf]

S1 Appendix. Detailed selection criteria

Inclusion criteria

Acute ischemic stroke patient must meet all the inclusion criteria;

1) whose relevant ischemic lesions were documented by CT or MR imaging and admitted within 7 days after symptom onset
2) ≥19 year-old
3) medically and neurologically stabilized to administer BP-lowering medications
4) mean systolic blood pressure ≥135 mm Hg during two days before screening
5) able to take oral medication
6) expected to operate the bluetooth-equipped sphygmomanometer, understand blood pressure measurement instructions and follow behavioral interventions or call for breakthrough visit
7) give informed consent in accordance with International Conference on Harmonization (ICH) Good Clinical Practice (GCP) guideline and local regulation with his/her own will

Exclusion criteria

The patient must not meet any exclusion criteria;

1) pregnant, puerperium within 30 days or on breastfeeding
2) enrolled to other interventional clinical trials
3) being expected to transfer to rehabilitation centers or institutionalized
4) being expected to have cerebral artery (including carotid arteries) interventions within 3 months after screening or randomization
5) known allergic reactions to olmesartan, amlodipine or hydrochlorothiazide
6) known severe hepatic disease
7) advanced kidney dysfunction requiring dialysis
8) being unlikely, by the opinion of the investigator, to comply with the clinical trial protocol or being unsuitable for any other reasons
