# Supplementary material for: Remote blood pressure monitoring and behavioral intensification for stroke: A randomized controlled feasibility trial
Source: PLoS One. 2020 Mar 11;15(3):e0229483. doi: 10.1371/journal.pone.0229483 (PMC7065804; doi:10.1371/journal.pone.0229483)
Supplement: S2 Appendix — (PDF) [file pone.0229483.s004.pdf]

S2 Appendix. Suggested prescription algorithm of BP-lowering medication

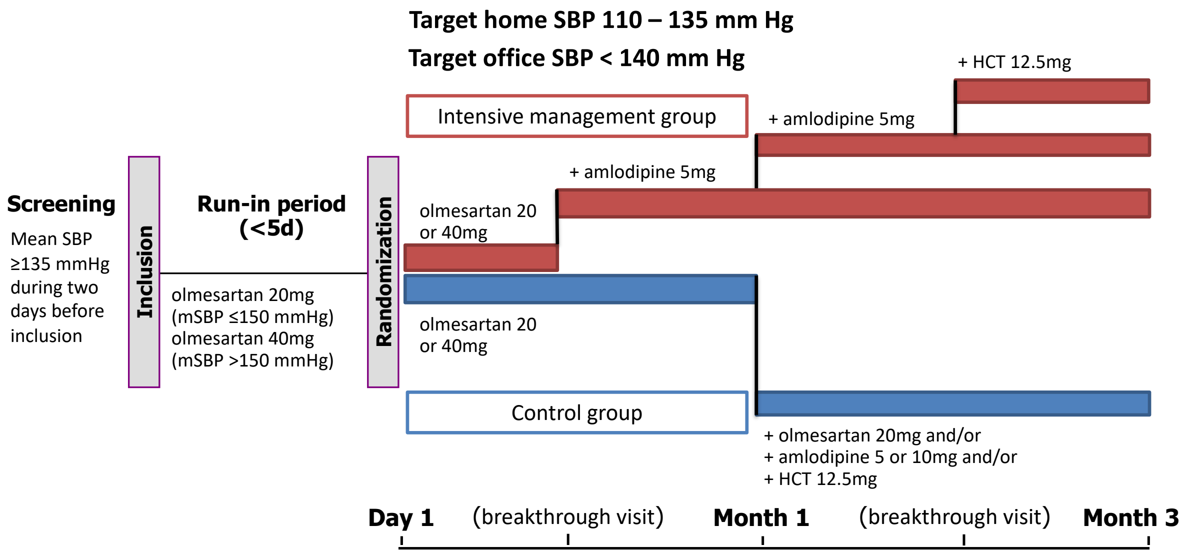

- Suggested prescription algorithm of BP-lowering medication was modified from similar clinical trials.[1, 2]
- Stepwise adjustment strategy of BP-lowering medication for the intensive management group
  - At randomization: olmesartan 20 mg (mean SBP ≤150 mm Hg) or olmesartan 40 mg (mean SBP >150 mm Hg)
  - Step 1: add amlodipine 5 mg to the previous step
  - Step 2: add amlodipine 5 mg to the previous step
  - Step 3: add hydrochlorothiazide 12.5 mg to the previous step
  - Removal of BP-lowering medication by an inverse order
- BP-lowering medication for control group
  - At randomization: olmesartan 20 mg (mean SBP ≤150 mm Hg) or olmesartan 40 mg (mean SBP >150 mm Hg)
  - adjustment by the responsible physicians' discretion

Reference

1. Sanford M, Keam SJ. Olmesartan medoxomil/amlodipine. *Drugs*. 2009;69:717-729
2. Neutel JM, Smith DHG, Weber MA, Wang AC, Masonson HN. Use of an olmesartan medoxomil-based treatment algorithm for hypertension control. *J Clin Hypertens*. 2004;6:168-174
