# Supplementary material for: Remote blood pressure monitoring and behavioral intensification for stroke: A randomized controlled feasibility trial
Source: PLoS One. 2020 Mar 11;15(3):e0229483. doi: 10.1371/journal.pone.0229483 (PMC7065804; doi:10.1371/journal.pone.0229483)
Supplement: S3 Appendix — (PDF) [file pone.0229483.s005.pdf]

S3 Appendix. Description of trial endpoints: Half-day blocks and weighted average proportion of BP measurement outliers

Rate of number of blocks measured per patients

Step 1: Timings of BP measurements were enlisted by the unit of minutes.

Step 2: Duration of mechanical errors such as transmission failure or loss of connection were excluded from the period of participation.

Step 3: The period of participation was divided into “AM block” (00:00 - 11:59) and “PM block” (12:00 - 23:59).

Step 4: Timings of BP measurements were marked into respective half-day blocks. At least one BP measurements during the half-day block was checked as “measured interval.”

Step 5: Number of measured blocks was divided by number of total intervals.

Illustration for BP measurement and half-day block

Date and Time	Blood pressure
September 23, 2018 11:05	118/67
September 23, 2018 11:08	125/72
September 23, 2018 18:37	142/88
September 24, 2018 21:23	136/71
September 24, 2018 21:31	128/65
September 25, 2018 07:09	121/63
September 25, 2018 16:22	139/74

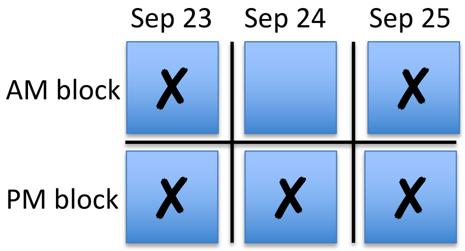

Number of marked blocks: 5
Number of total blocks: 6
Rate of number of blocks measured per patient: 0.83

weighted average proportion of BP measurement outliers

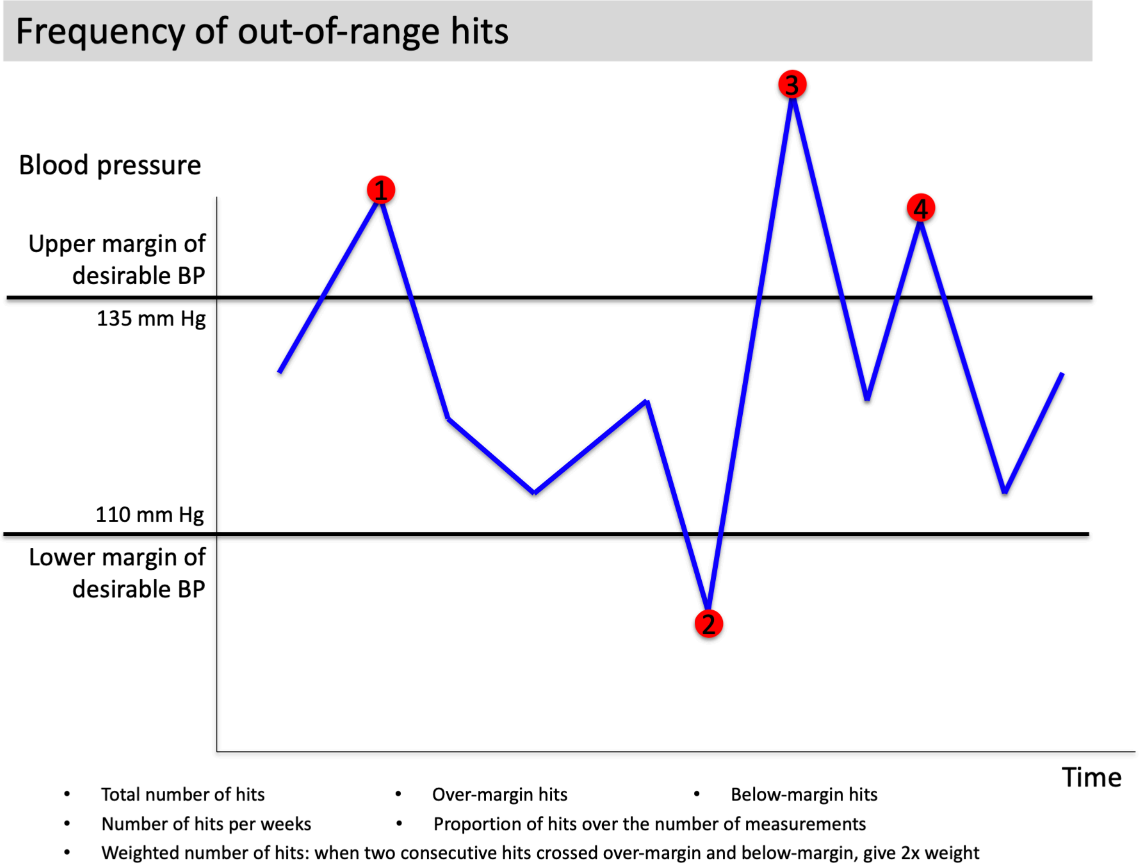

When two consecutive measurements were out of desirable range (measurement #2 and #3 in the depicted example), preceding out-of-range measurements were counted as two instead of one.

Two-sided OOR: measurement #1, #2, #3 and #4

Low OOR: measurement #2

High OOR: measurement #1, #3 and #4
