# Supplementary material for: Remote blood pressure monitoring and behavioral intensification for stroke: A randomized controlled feasibility trial
Source: PLoS One. 2020 Mar 11;15(3):e0229483. doi: 10.1371/journal.pone.0229483 (PMC7065804; doi:10.1371/journal.pone.0229483)
Supplement: S4 Appendix — (PDF) [file pone.0229483.s006.pdf]

S4 Appendix. Details on failure to reach success criteria for feasibility

1) Delays in response to breakthrough calls

Subject No.	Breakthrough visit #	Issue date of visit	Response date	Response delay (day)
**1**	1	17/05/2017	18/05/2017	1
**2**	1	24/07/2017	28/07/2017	4
**3**	1	18/09/2017	29/09/2017	11
**4**	1	19/09/2017	22/09/2017	3
**5**	1	31/10/2017	31/10/2017	0
**5**	2	08/11/2017	08/11/2017	0
**6**	1	17/01/2017	19/01/2017	2
**7**	1	03/03/2017	06/03/2017	3

2) Noncompliance to BP measurement requirements

Subject No.	Compliance (%)
**1**	7.35
**2**	21.81
**3**	20.39

3) Extended duration of mechanical errors

Subject No.	Randomized group	Duration (day)
**1**	Control group	11
**2**	Control group	60
**3**	Control group	26
**4**	Intensive management group	11
**5**	Intensive management group	6
**6**	Control group	37
**7**	Control group	3
**8**	Control group	10

4) Failure to reach required rate of number of measured half-day blocks

Subject No.	Randomized group	rate(%)
**1**	Control group	62.195
**2**	Control group	7.353
**3**	Intensive management group	76.404
**4**	Intensive management group	62.222

**5**	Control group	65.385
**6**	Control group	76.02
**7**	Intensive management group	65.169
**8**	Control group	68.391
**9**	Control group	21.809
**10**	Intensive management group	76.63
**11**	Control group	77.222
**12**	Intensive management group	74.731
**13**	Intensive management group	75.51
**14**	Control group	54.688
**15**	Intensive management group	50.595
**16**	Control group	20.388
**17**	Control group	51.724
**18**	Intensive management group	68.354
**19**	Intensive management group	79.333
**20**	Control group	64.607
**21**	Intensive management group	76.087
**22**	Control group	72.892
**23**	Control group	60
