# Supplementary material for: Remote blood pressure monitoring and behavioral intensification for stroke: A randomized controlled feasibility trial
Source: PLoS One. 2020 Mar 11;15(3):e0229483. doi: 10.1371/journal.pone.0229483 (PMC7065804; doi:10.1371/journal.pone.0229483)
Supplement: S5 Appendix — (PDF) [file pone.0229483.s007.pdf]

S5 Appendix. BP measurement outliers

Details of individual OOR measurements

	Intensive management group	Control group	Difference (95% CI)
Two-sided OOR			
Average	0.41 ± 0.17	0.43 ± 0.19	-0.02 (-0.11, 0.07)
Weighted	0.66 ± 0.33	0.67 ± 0.35	-0.01 (-0.18, 0.17)
Low OOR			
Average	0.13 ± 0.14	0.14 ± 0.16	-0.01 (-0.08, 0.07)
Weighted	0.20 ± 0.21	0.21 ± 0.26	-0.01 (-0.13, 0.12)
High OOR			
Average	0.28 ± 0.24	0.29 ± 0.24	-0.01 (-0.14, 0.11)
Weighted	0.45 ± 0.43	0.45 ± 0.41	0.01 (-0.21, 0.23)

Details of half-day interval OOR measurements

	Intensive management group	Control group	Difference (95% CI)
Two-sided OOR			
Average	0.38 ± 0.17	0.41 ± 0.20	-0.02 (-0.12, 0.07)
Weighted	0.57 ± 0.31	0.56 ± 0.30	-0.02 (-0.18, 0.15)
Low OOR			
Average	0.13 ± 0.13	0.13 ± 0.16	-0.01 (-0.09, 0.07)
Weighted	0.17 ± 0.20	0.19 ± 0.27	-0.02 (-0.14, 0.11)
High OOR			
Average	0.26 ± 0.23	0.27 ± 0.25	-0.02 (-0.14, 0.11)
Weighted	0.38 ± 0.38	0.38 ± 0.37	0 (-0.19, 0.19)

Values presented as mean±SD.
