# Supplementary figures and images for: Remote blood pressure monitoring and behavioral intensification for stroke: A randomized controlled feasibility trial
Source: PLoS One. 2020 Mar 11;15(3):e0229483. doi: 10.1371/journal.pone.0229483 (PMC7065804; doi:10.1371/journal.pone.0229483)

**S1 Figure. Recruitment time to prespecified number of subjects by recruiting center**

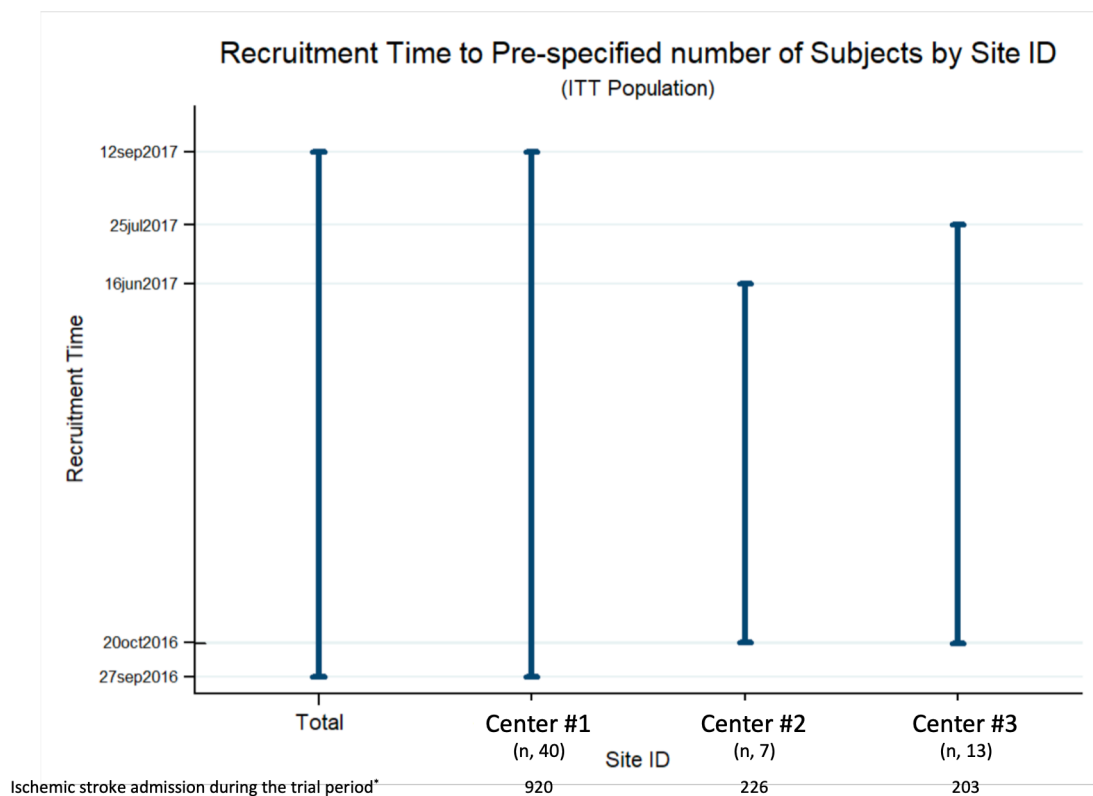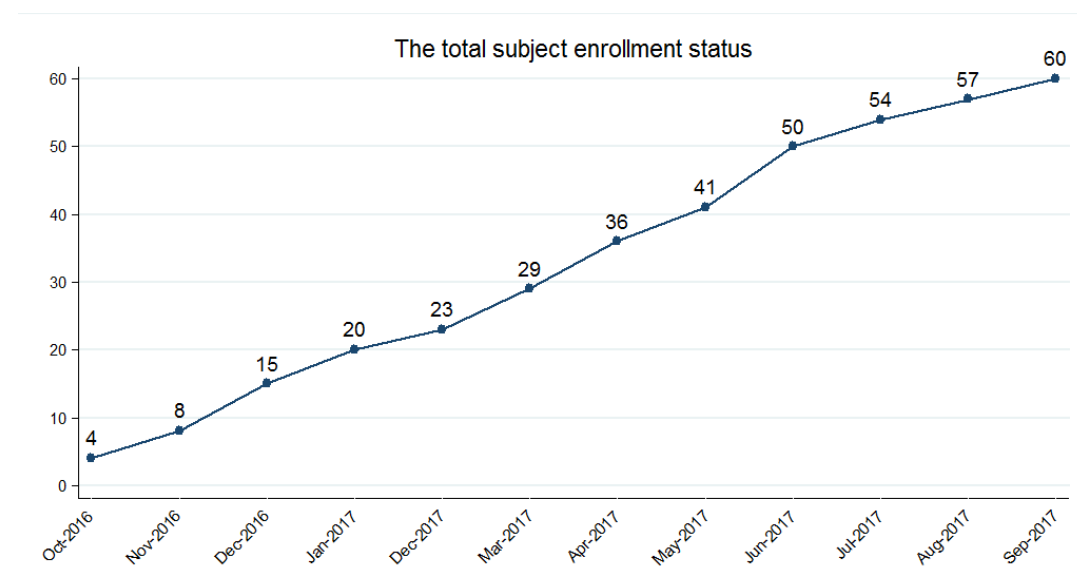

Supplement: S1 Fig — (PDF) [file pone.0229483.s008.pdf]

**S2 Figure. Individual BP plot**

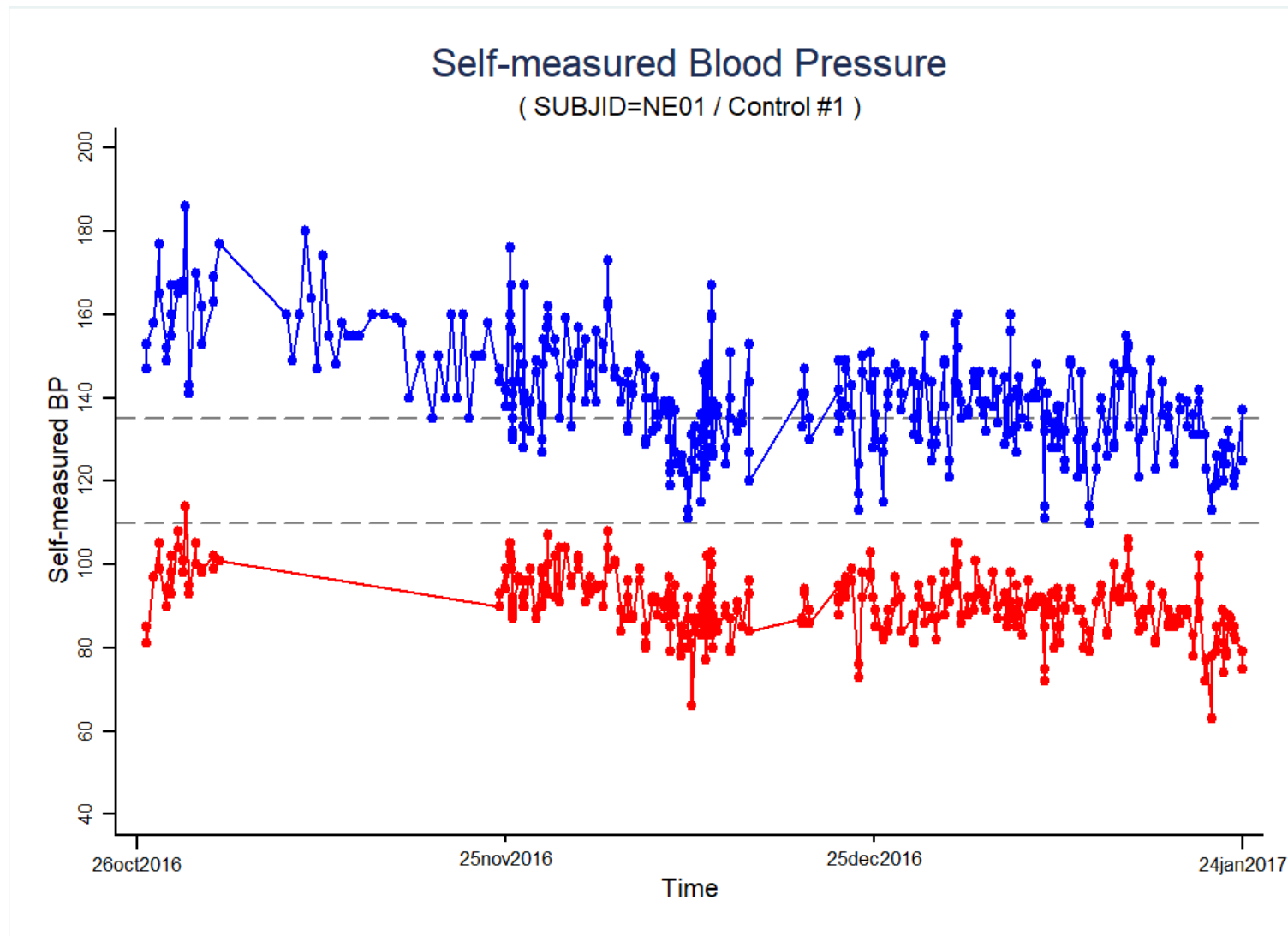

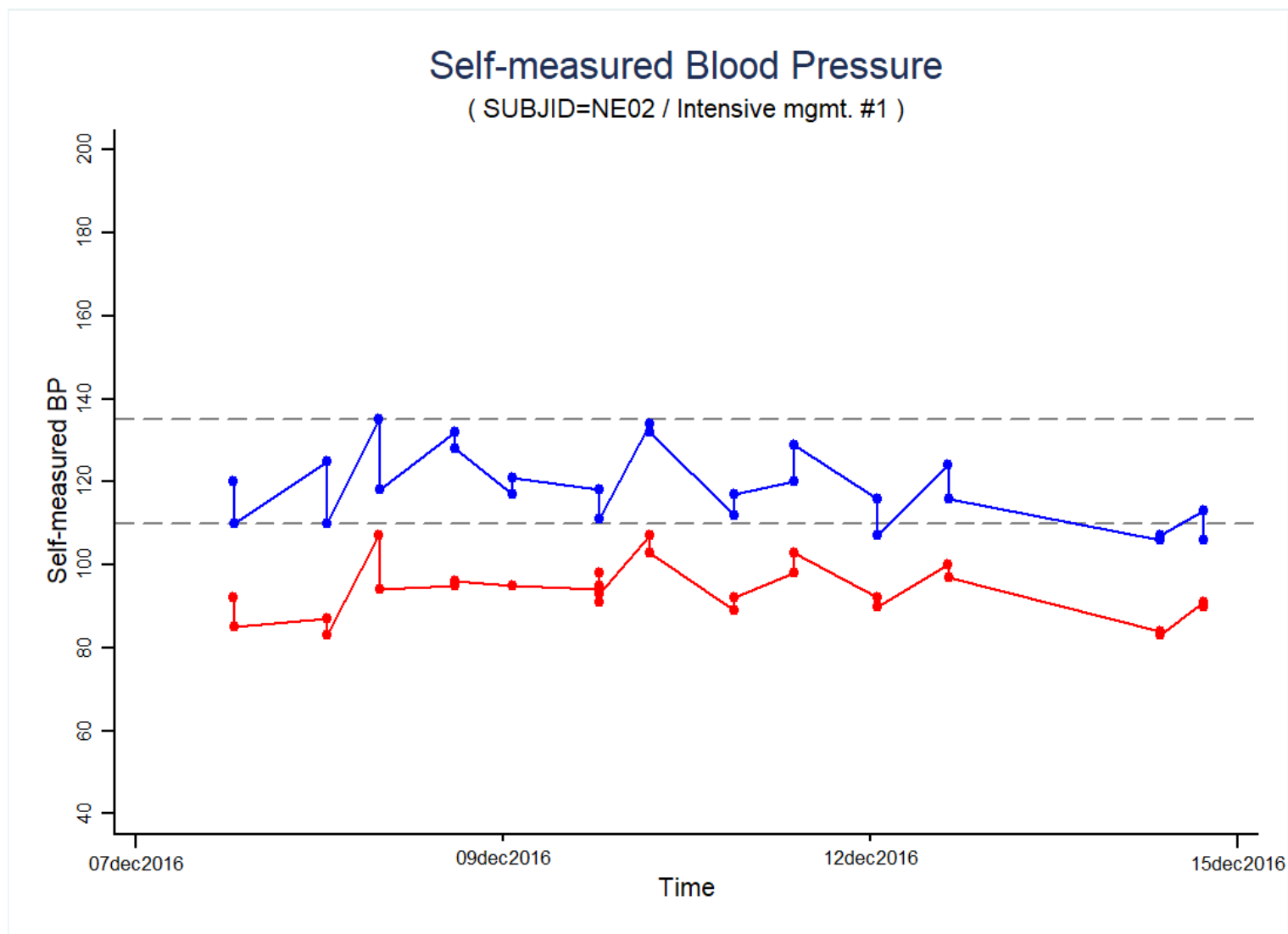

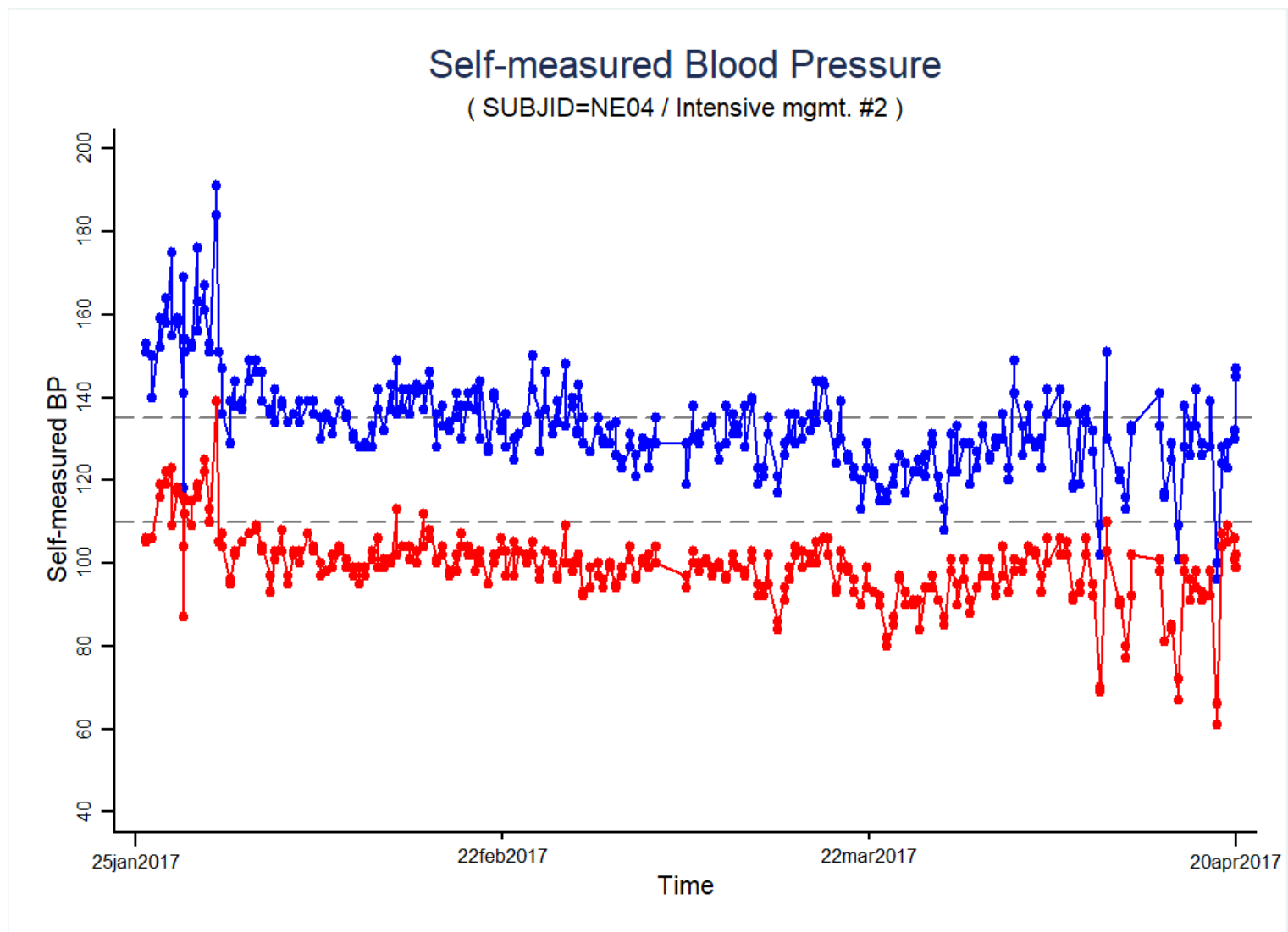

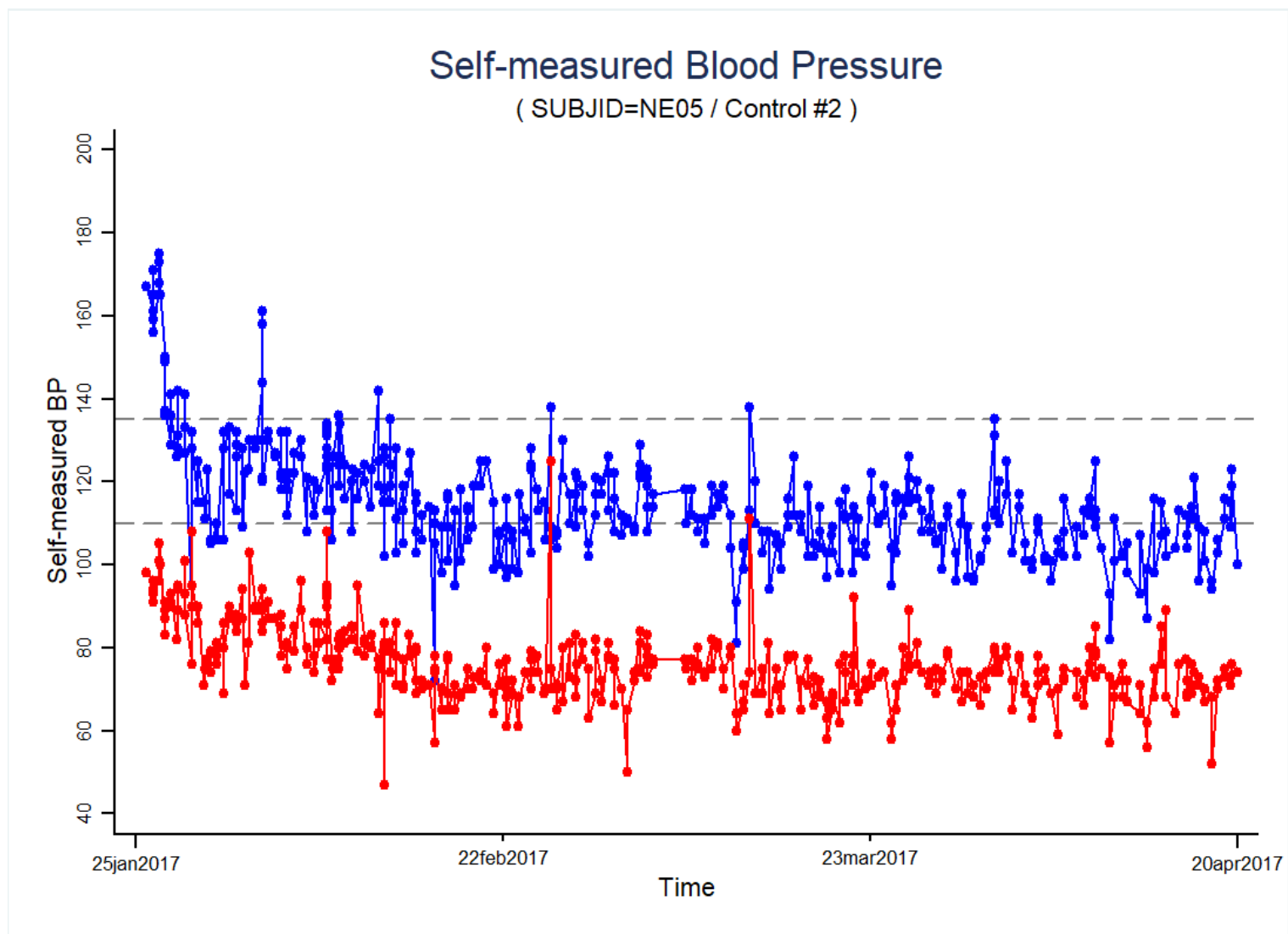

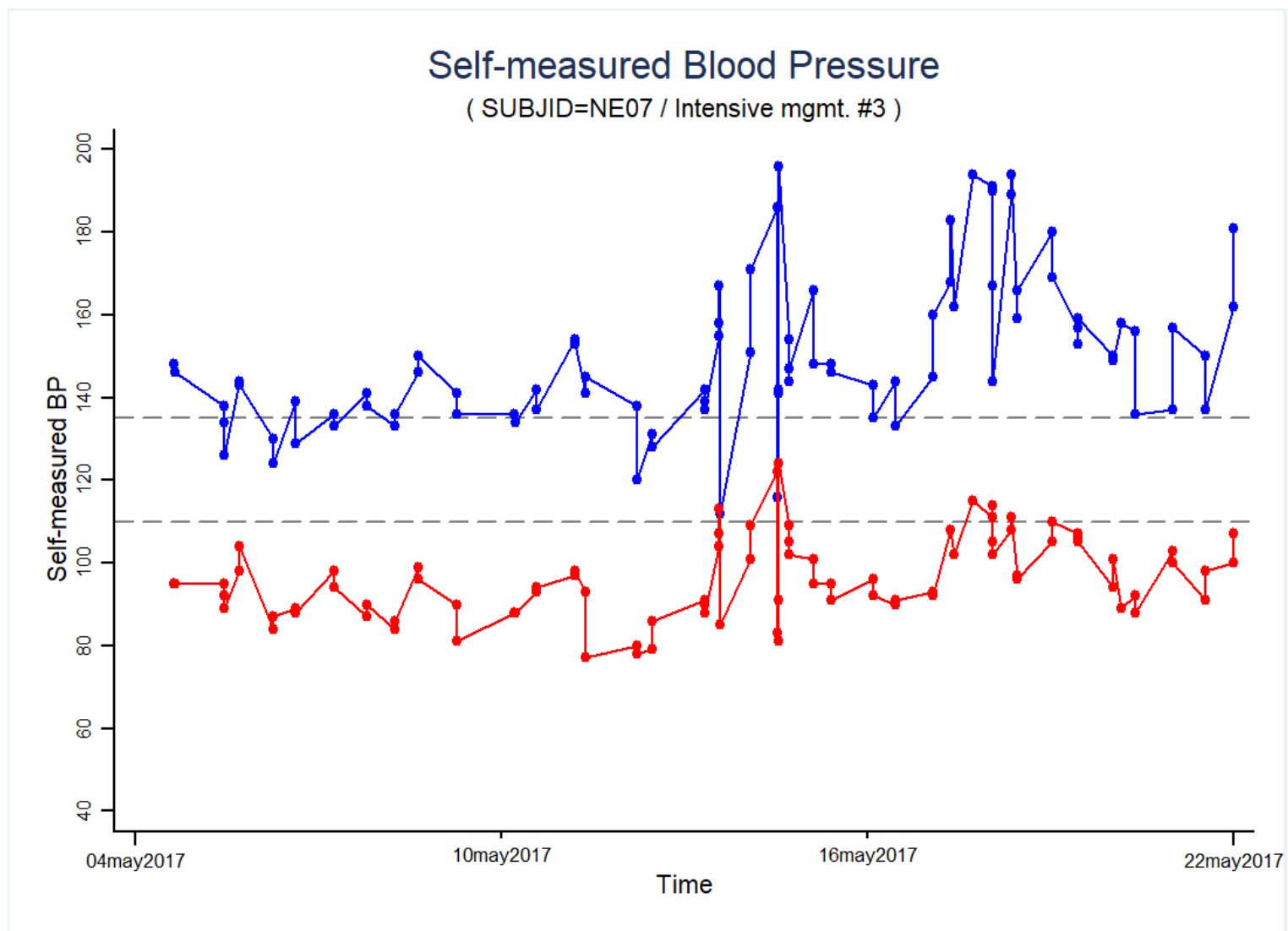

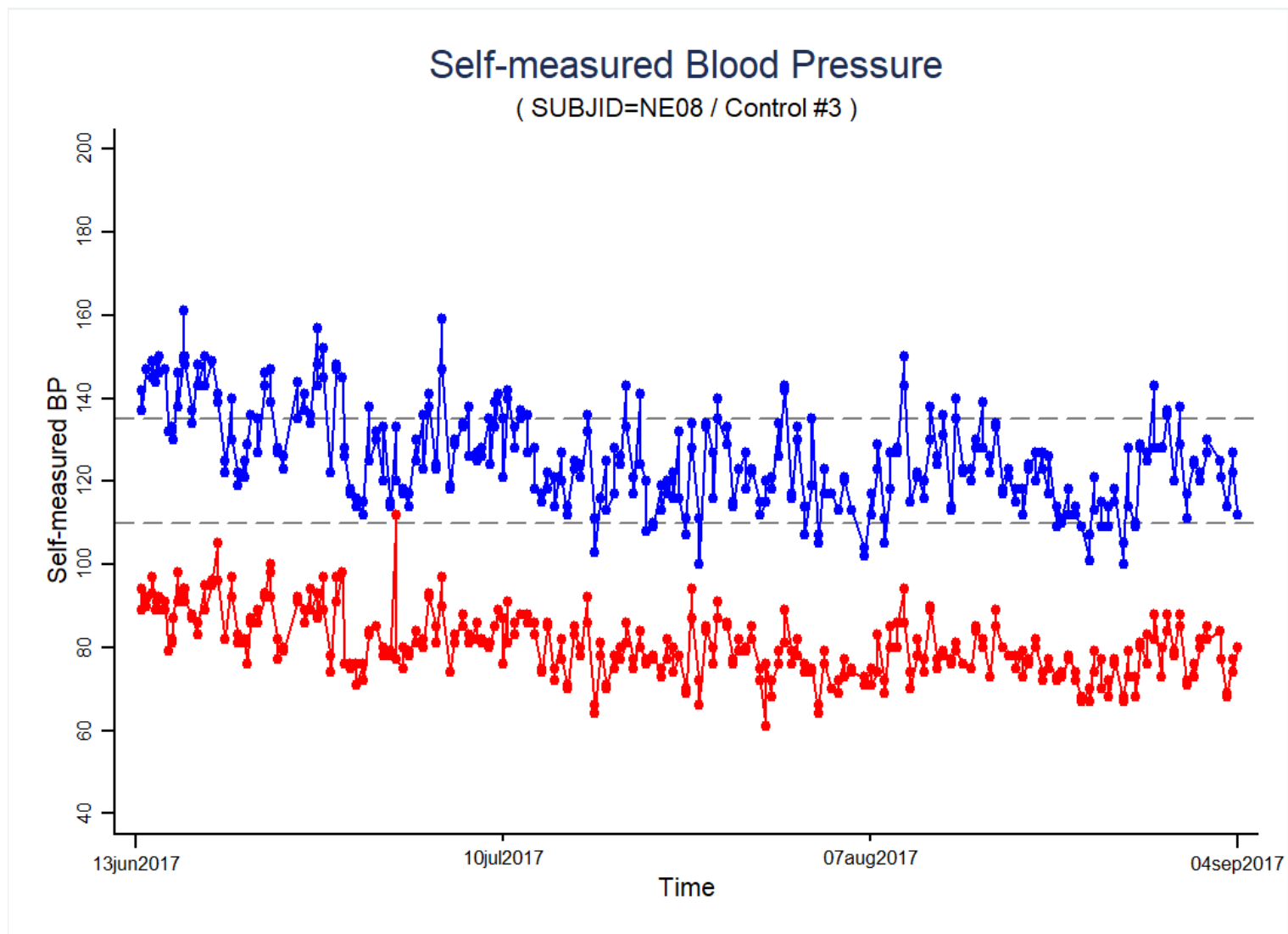

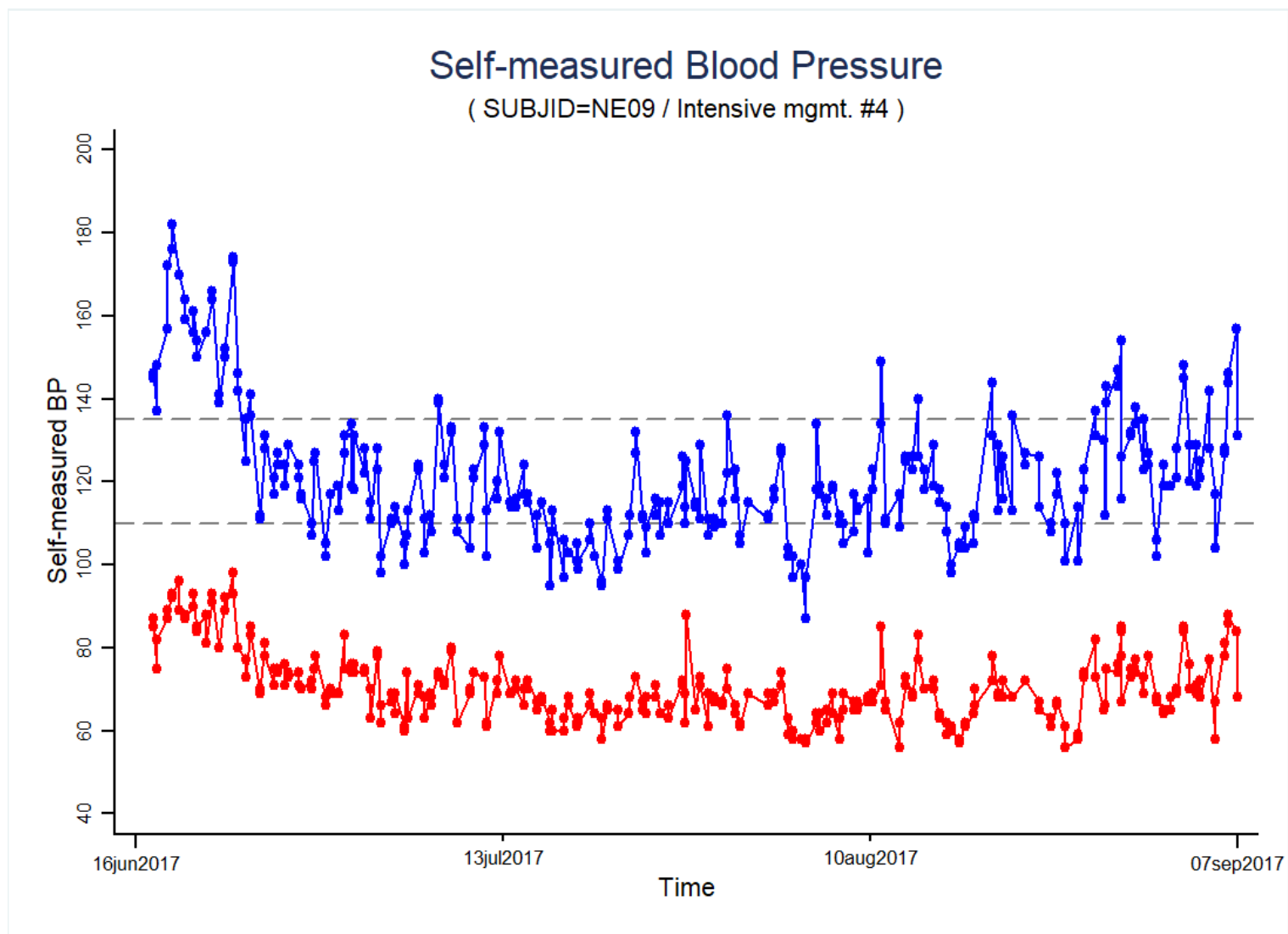

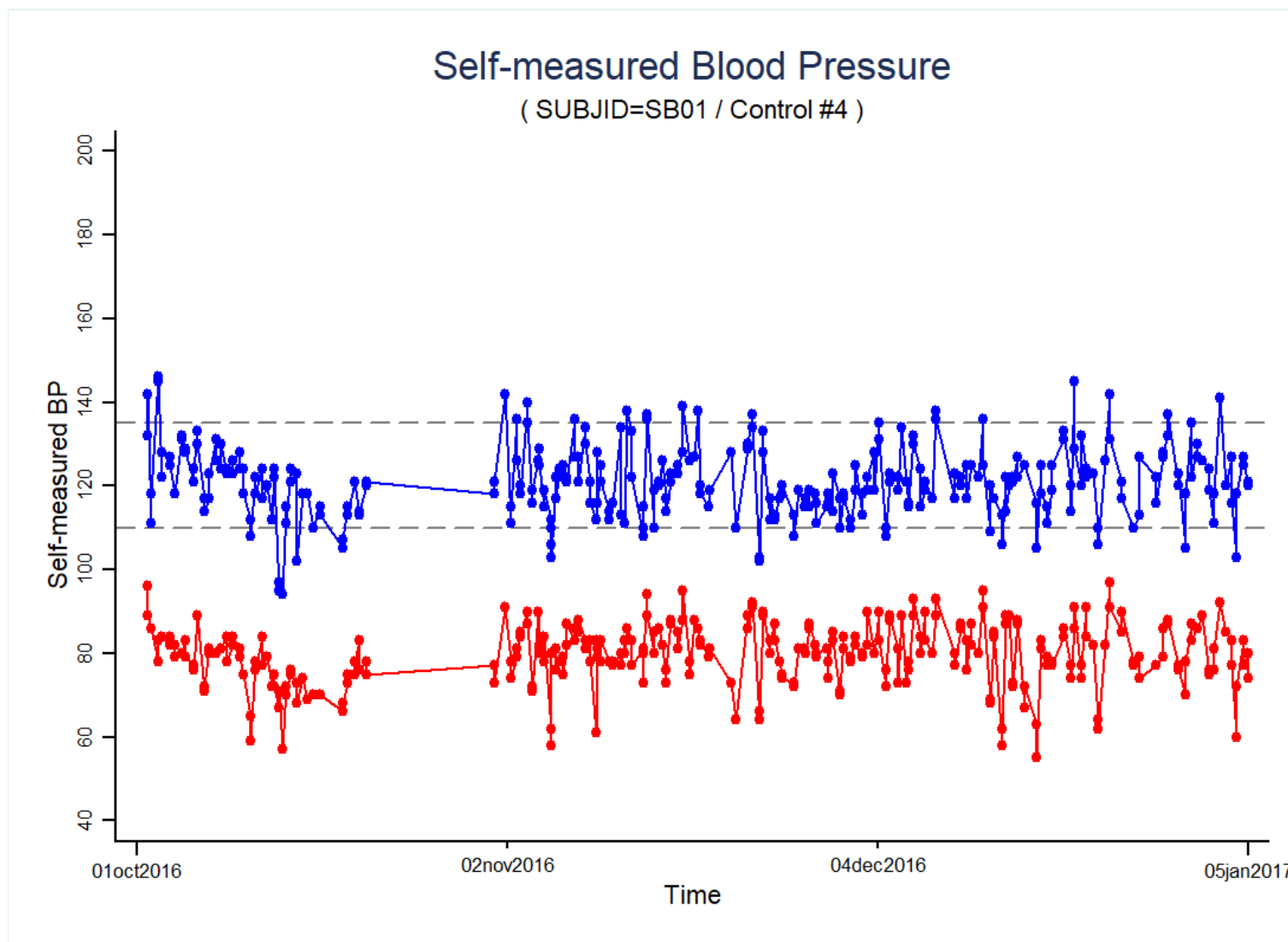

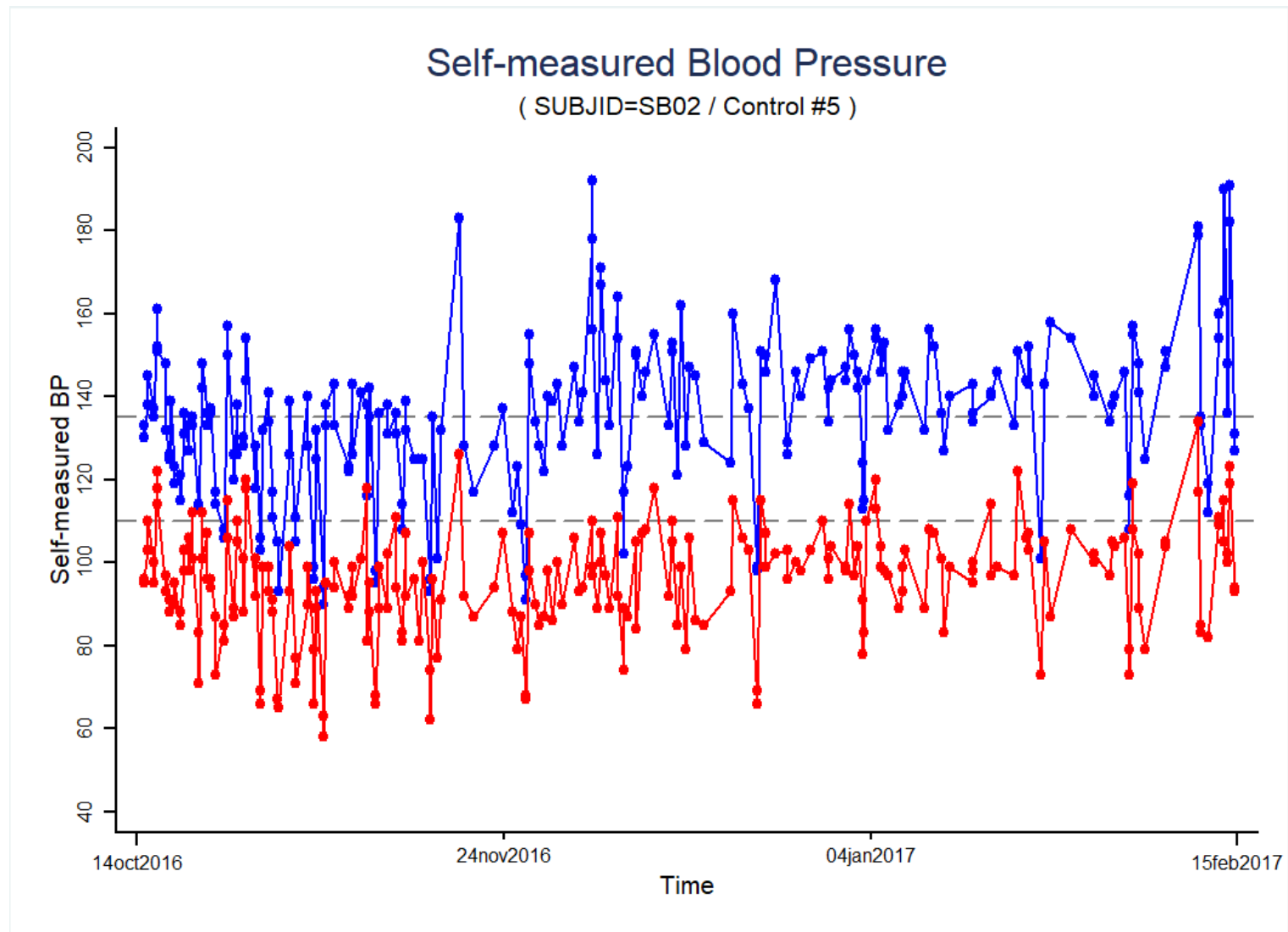

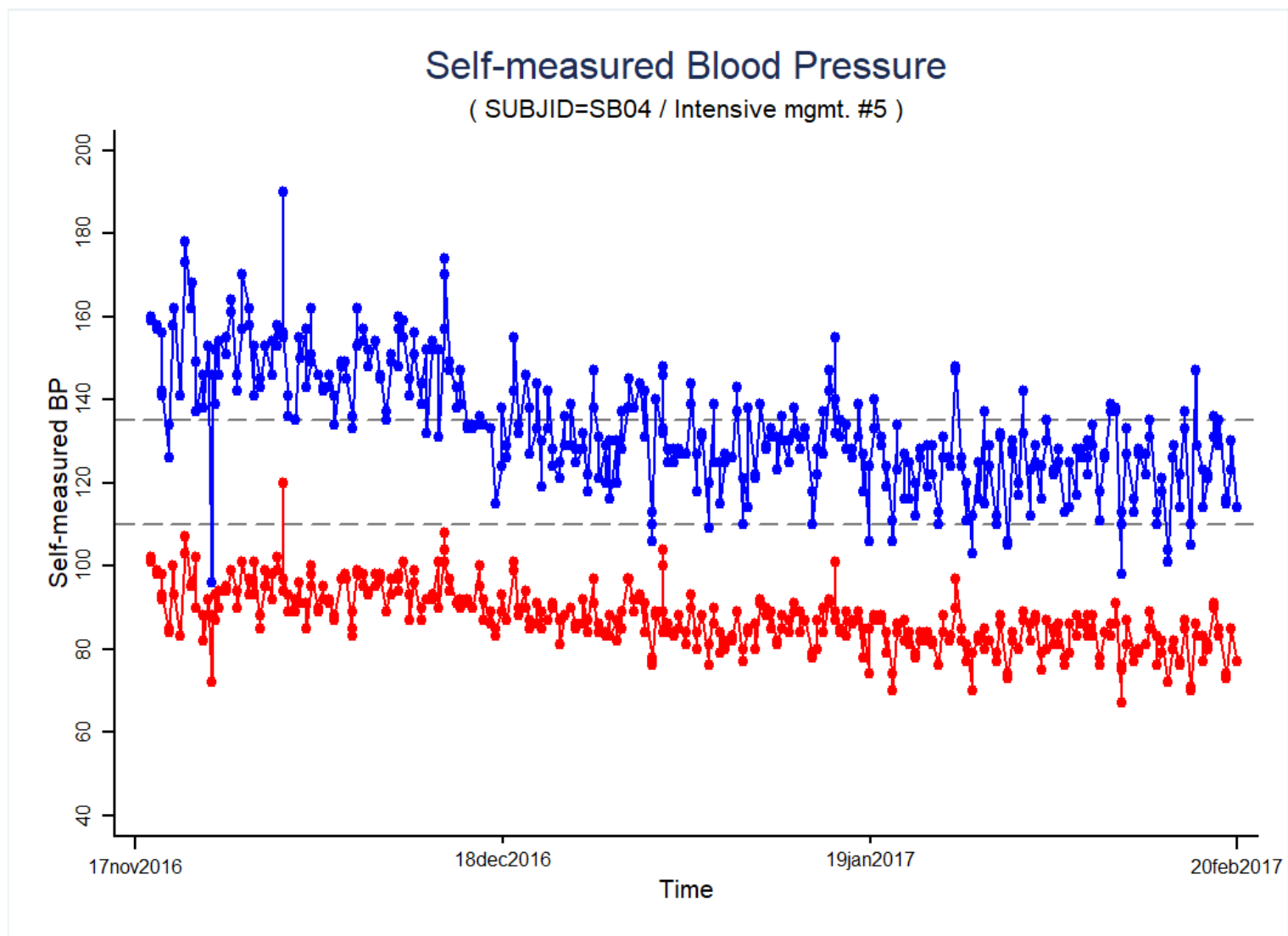

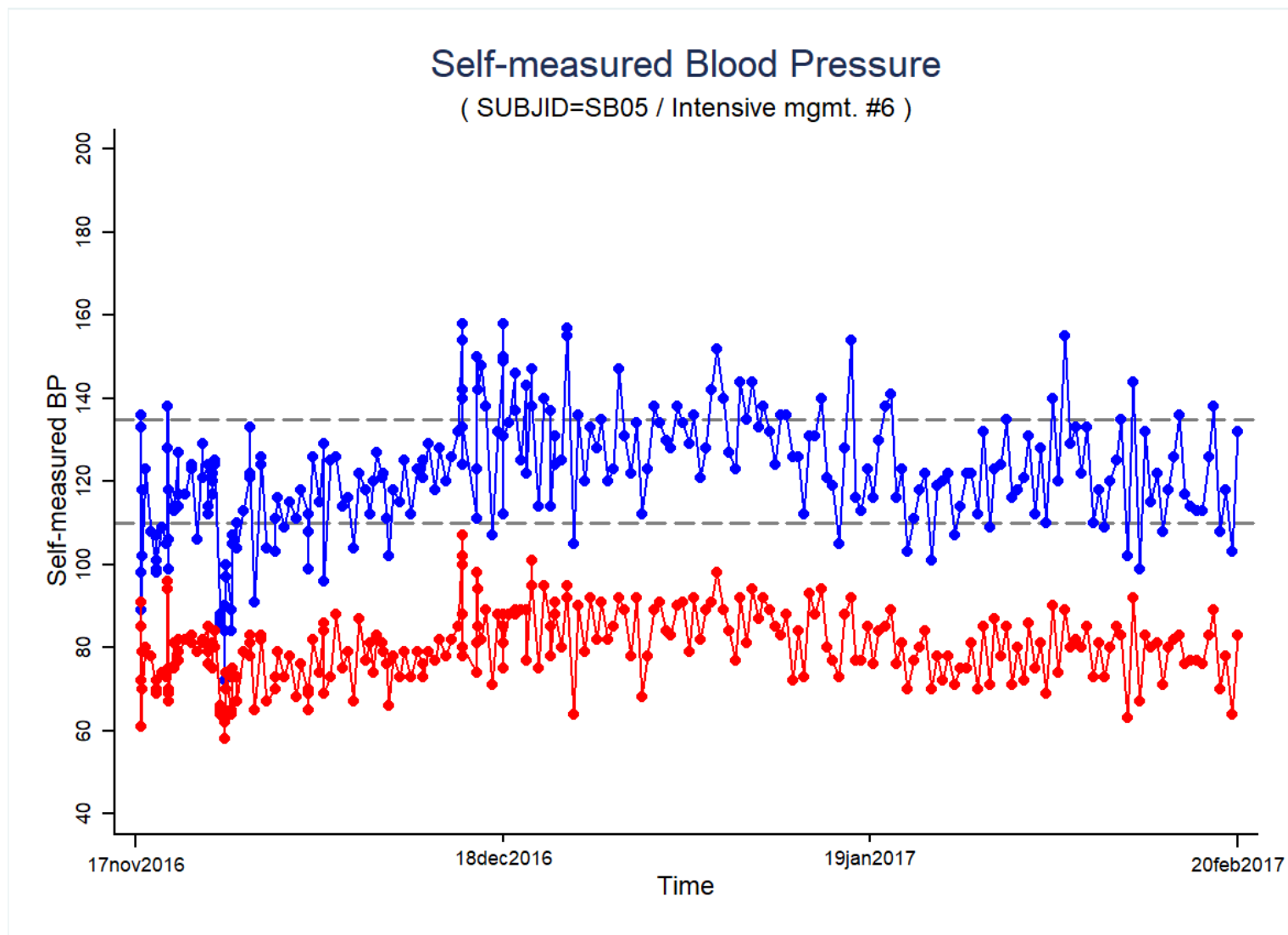

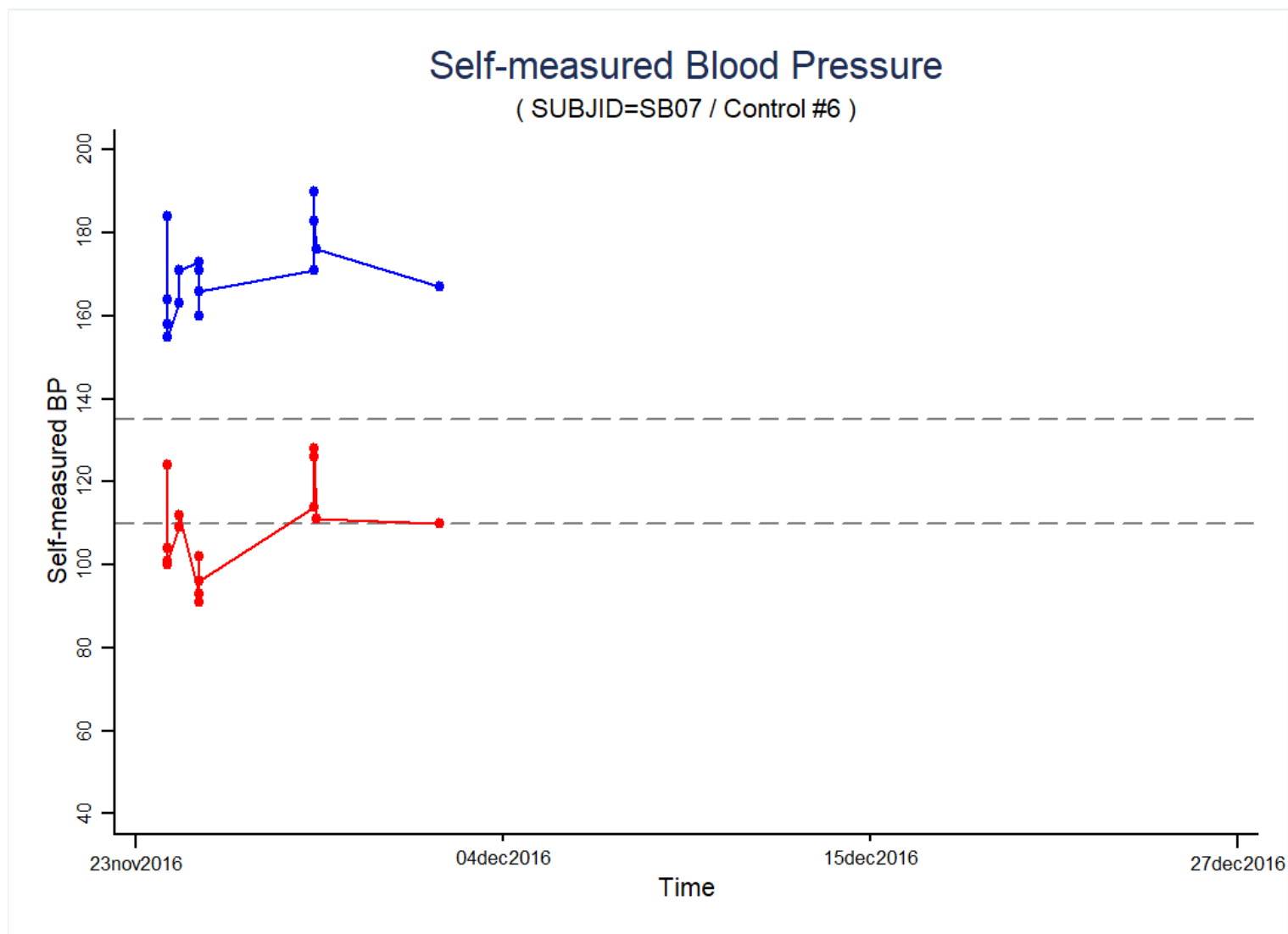

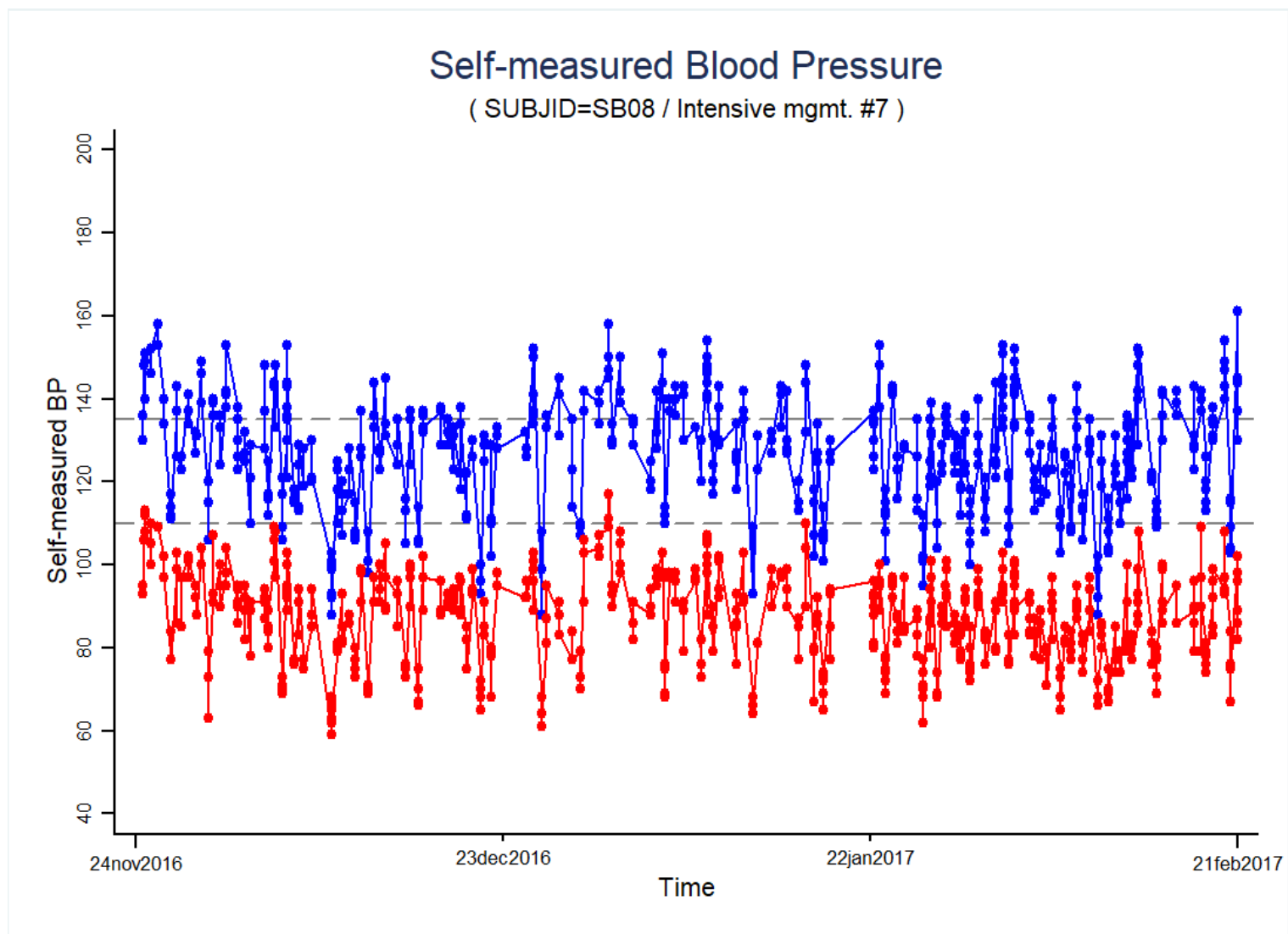

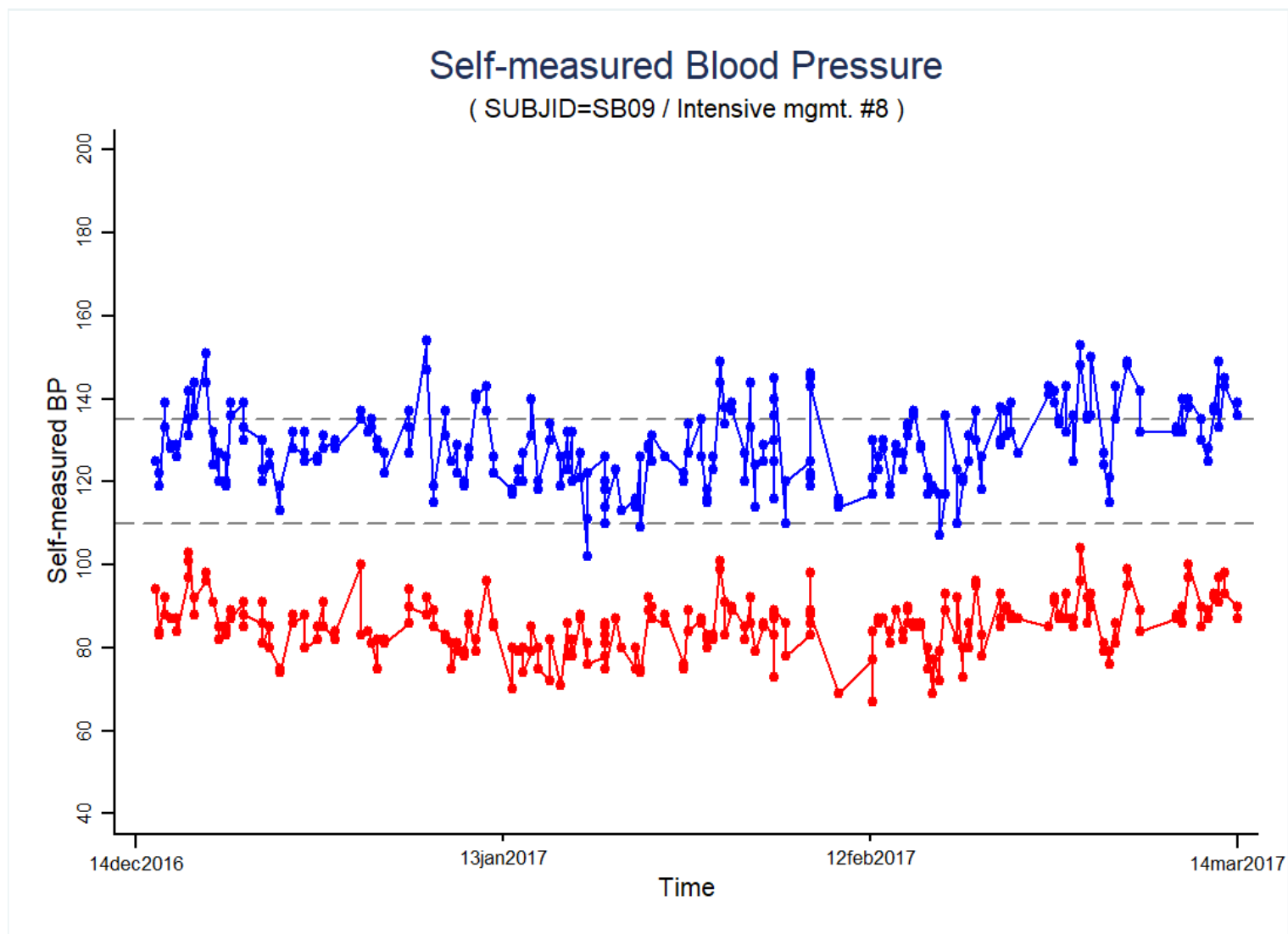

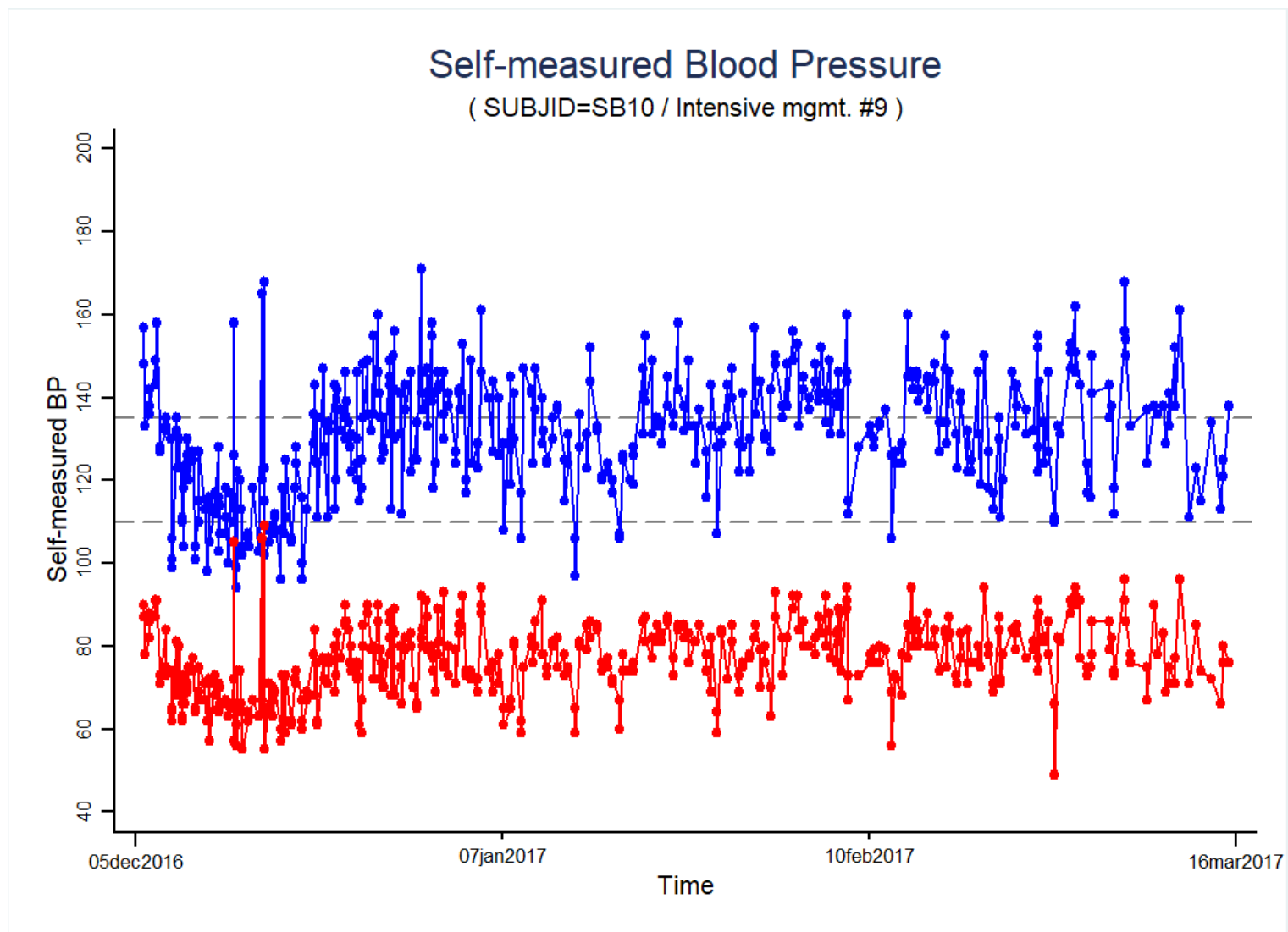

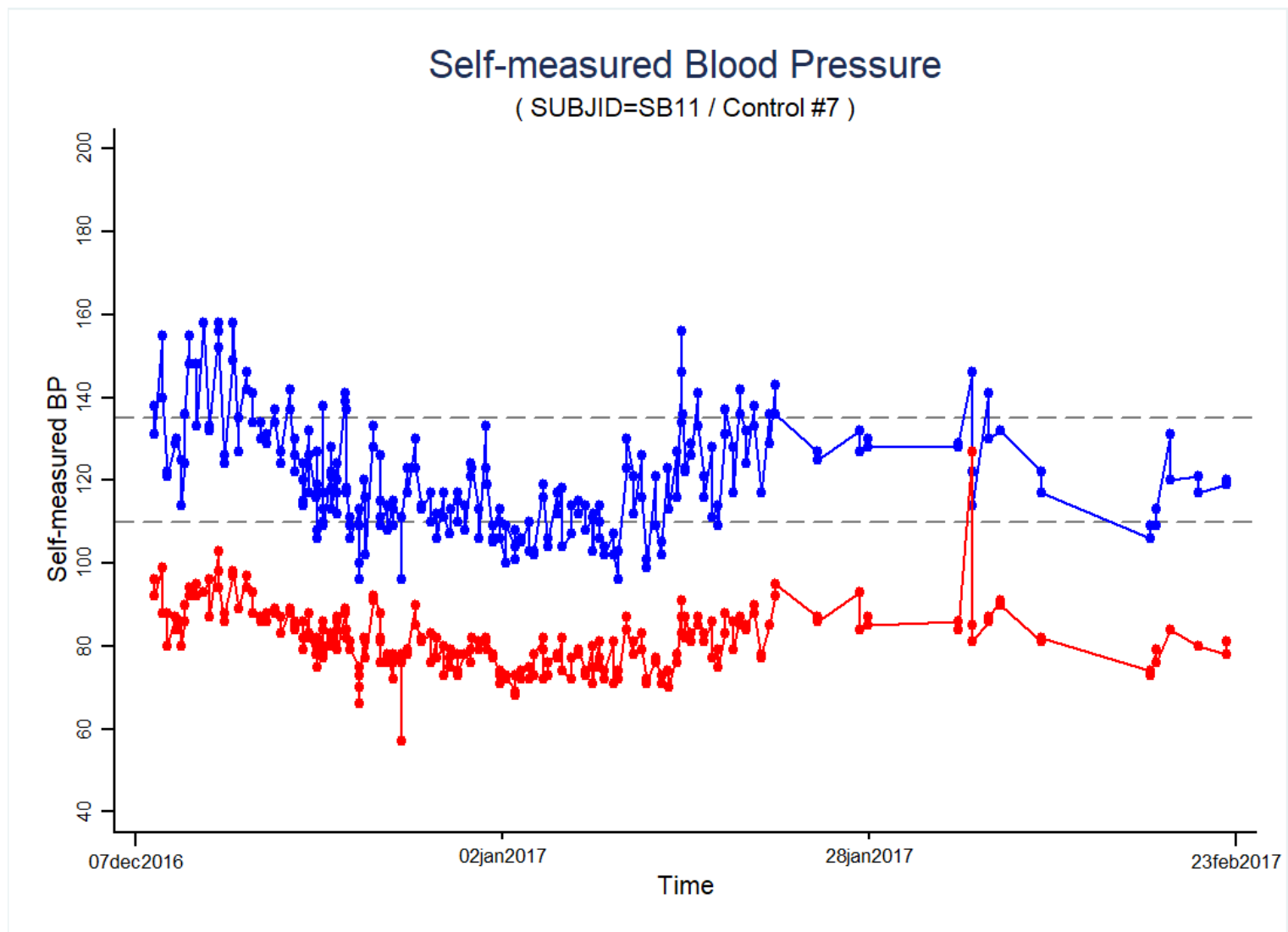

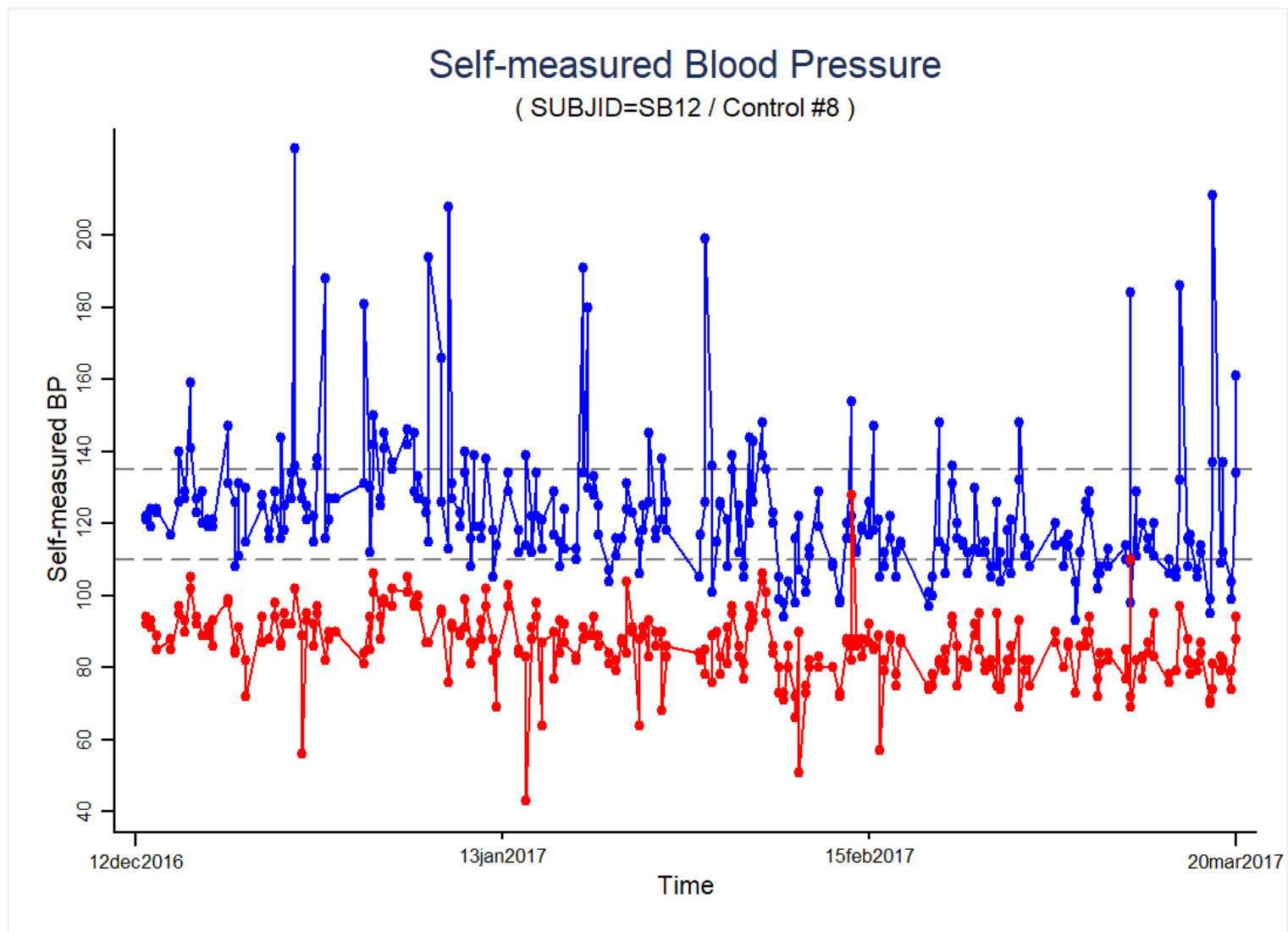

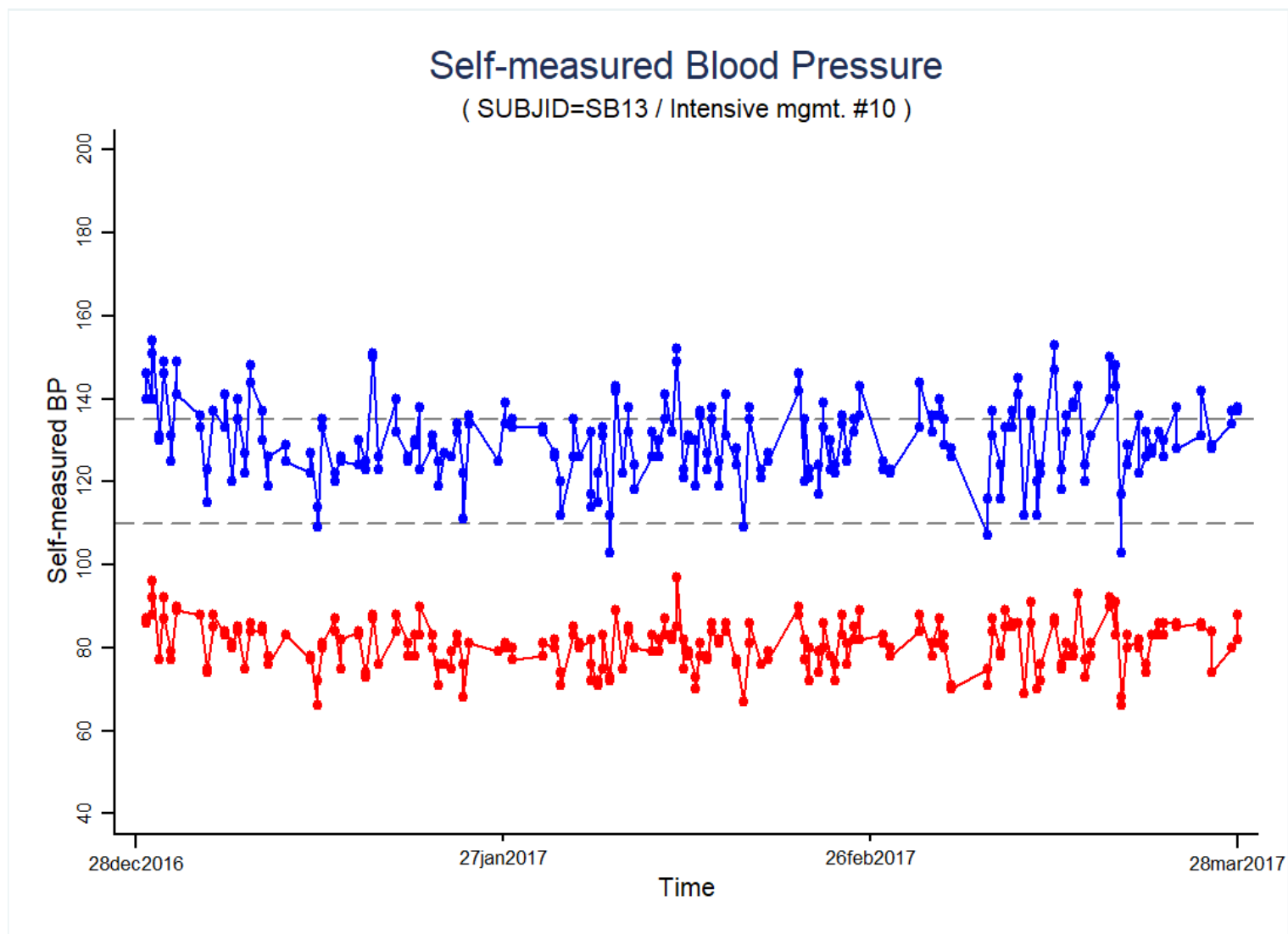

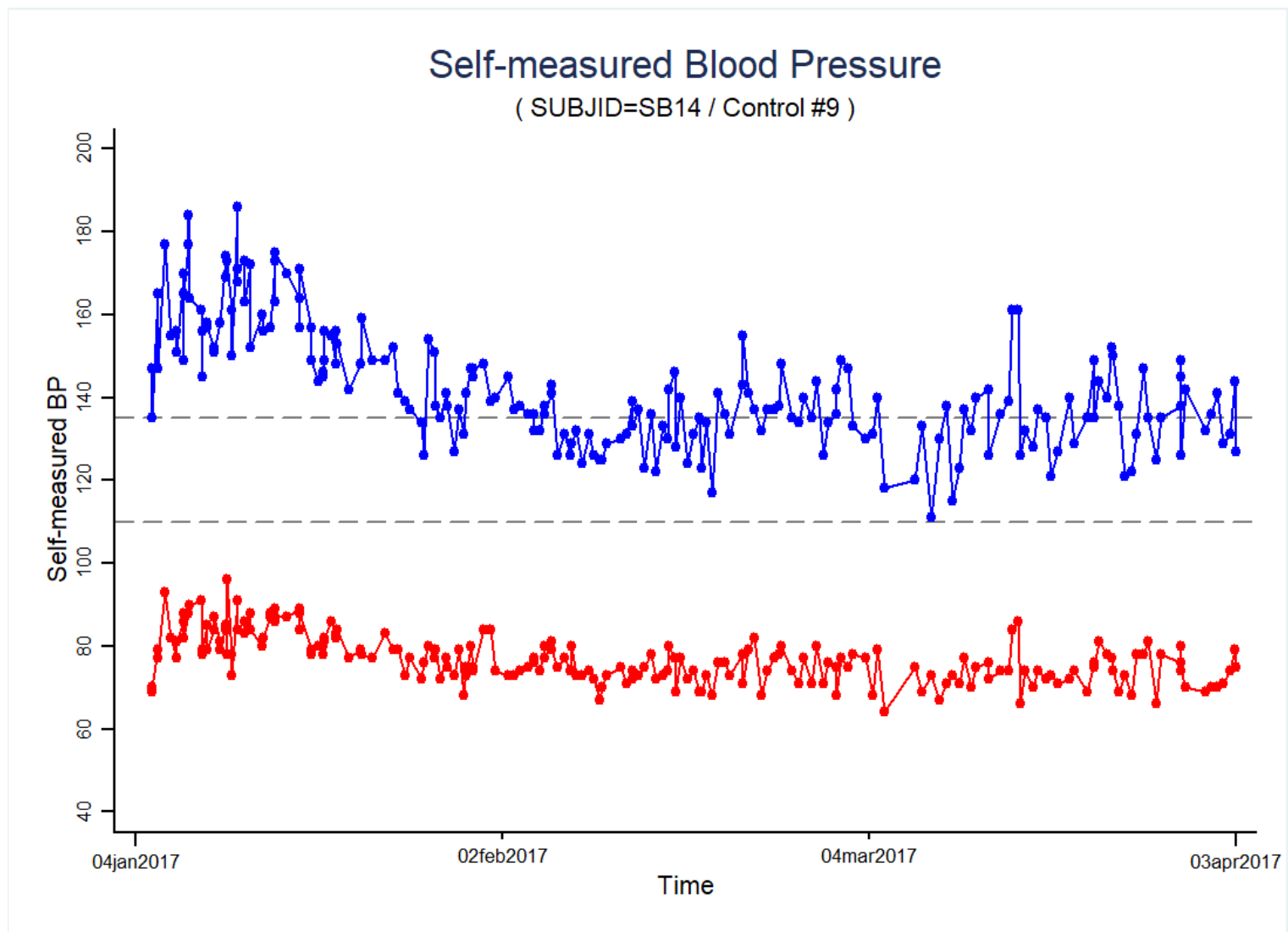

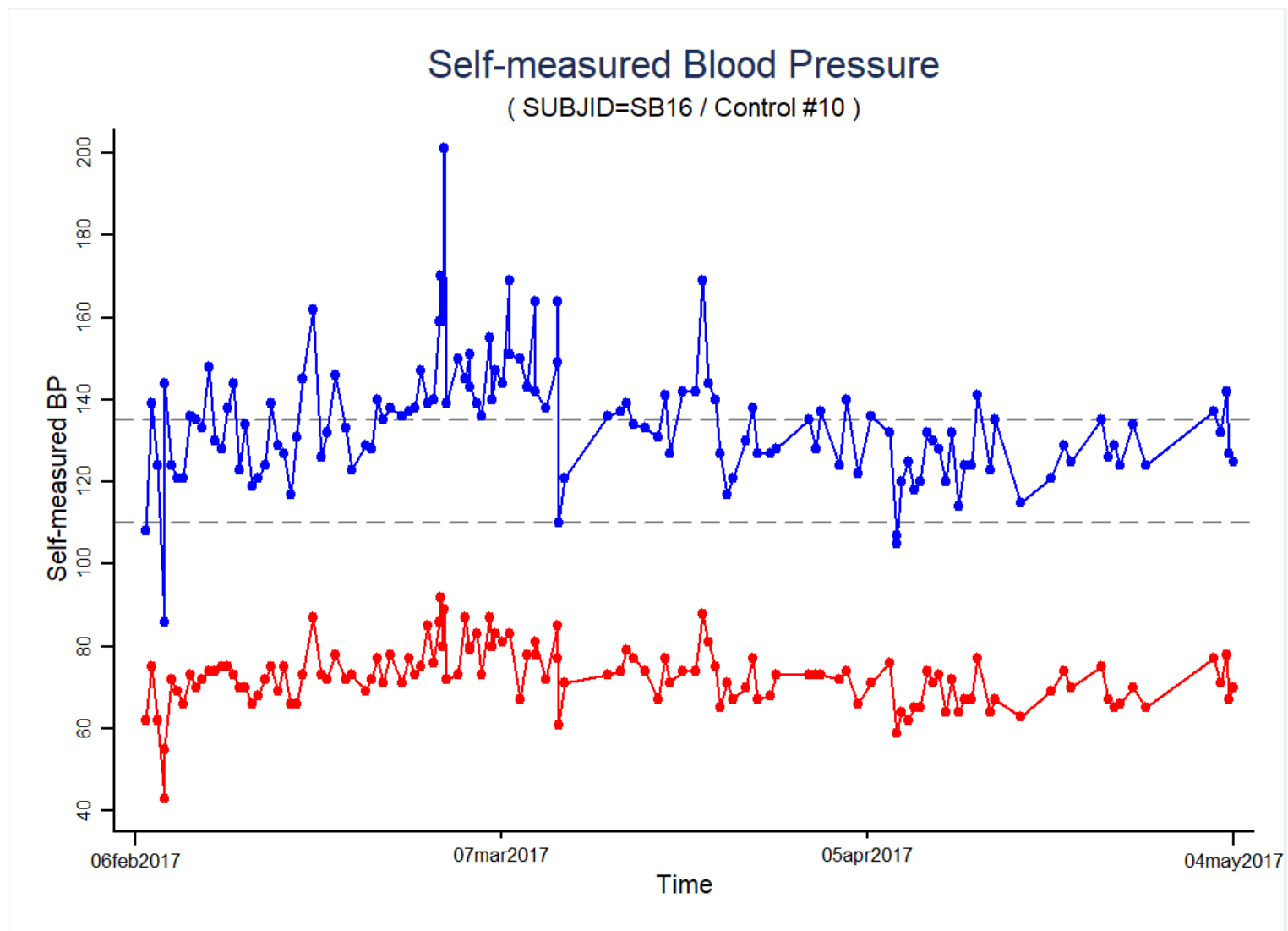

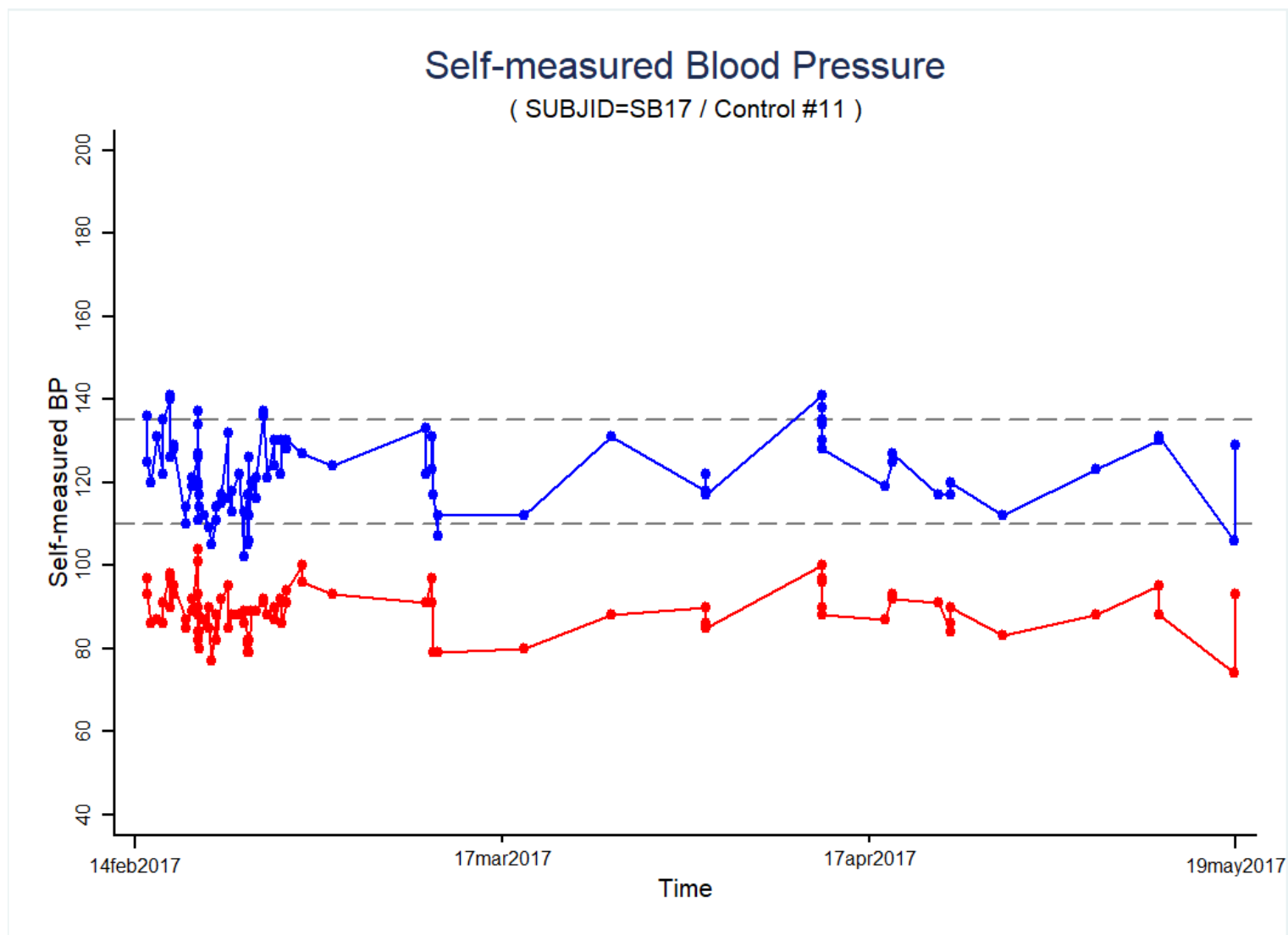

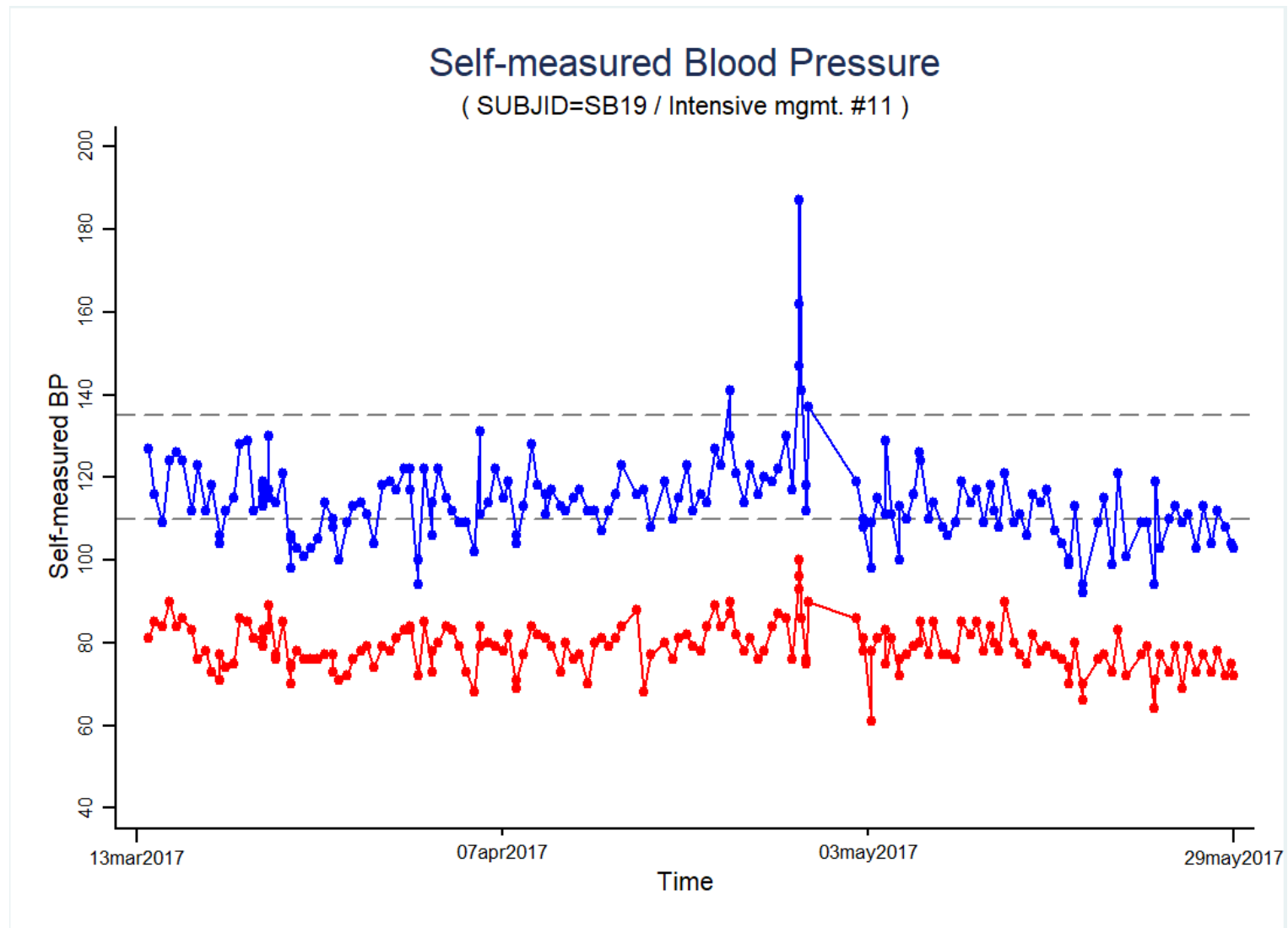

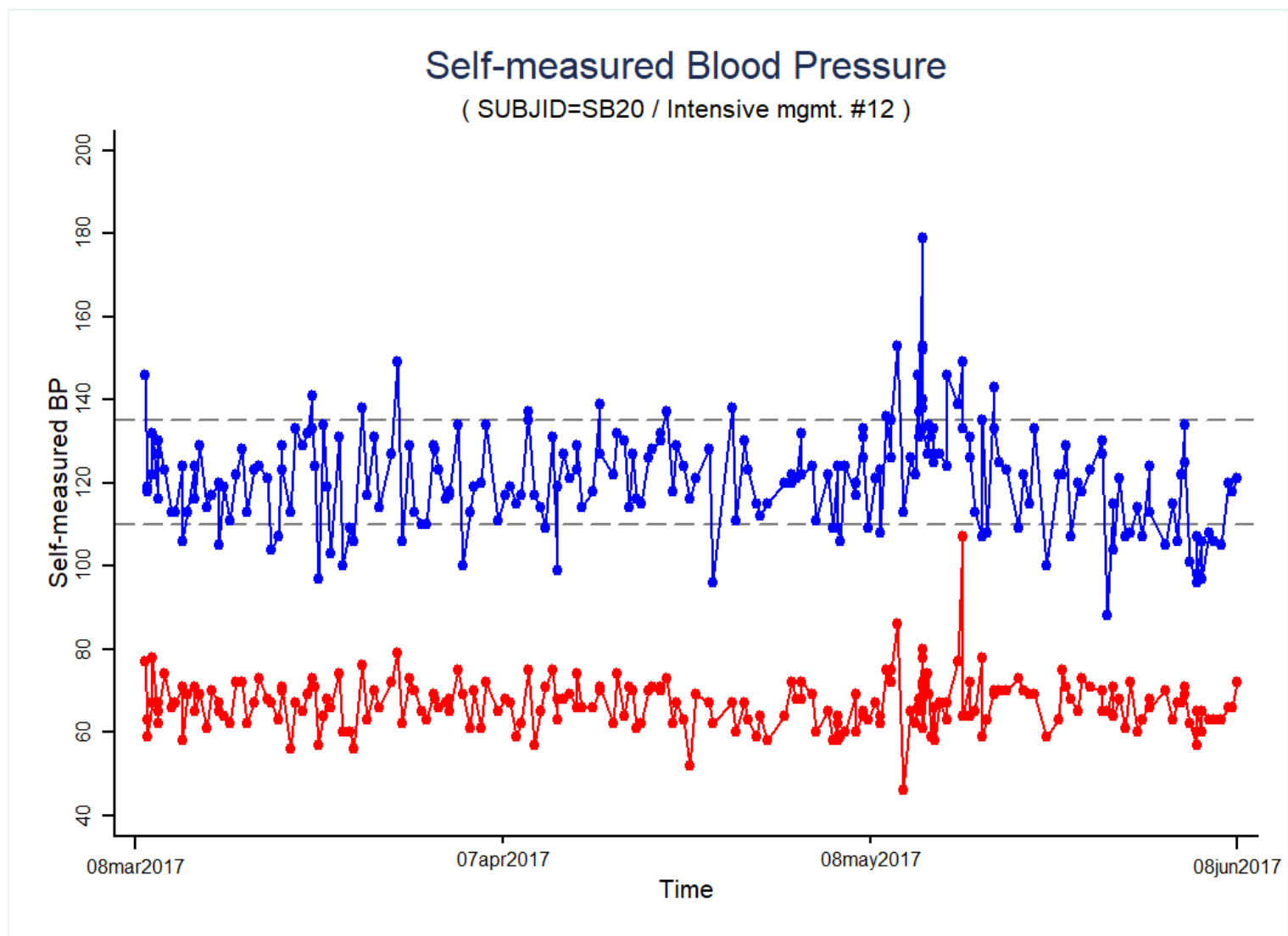

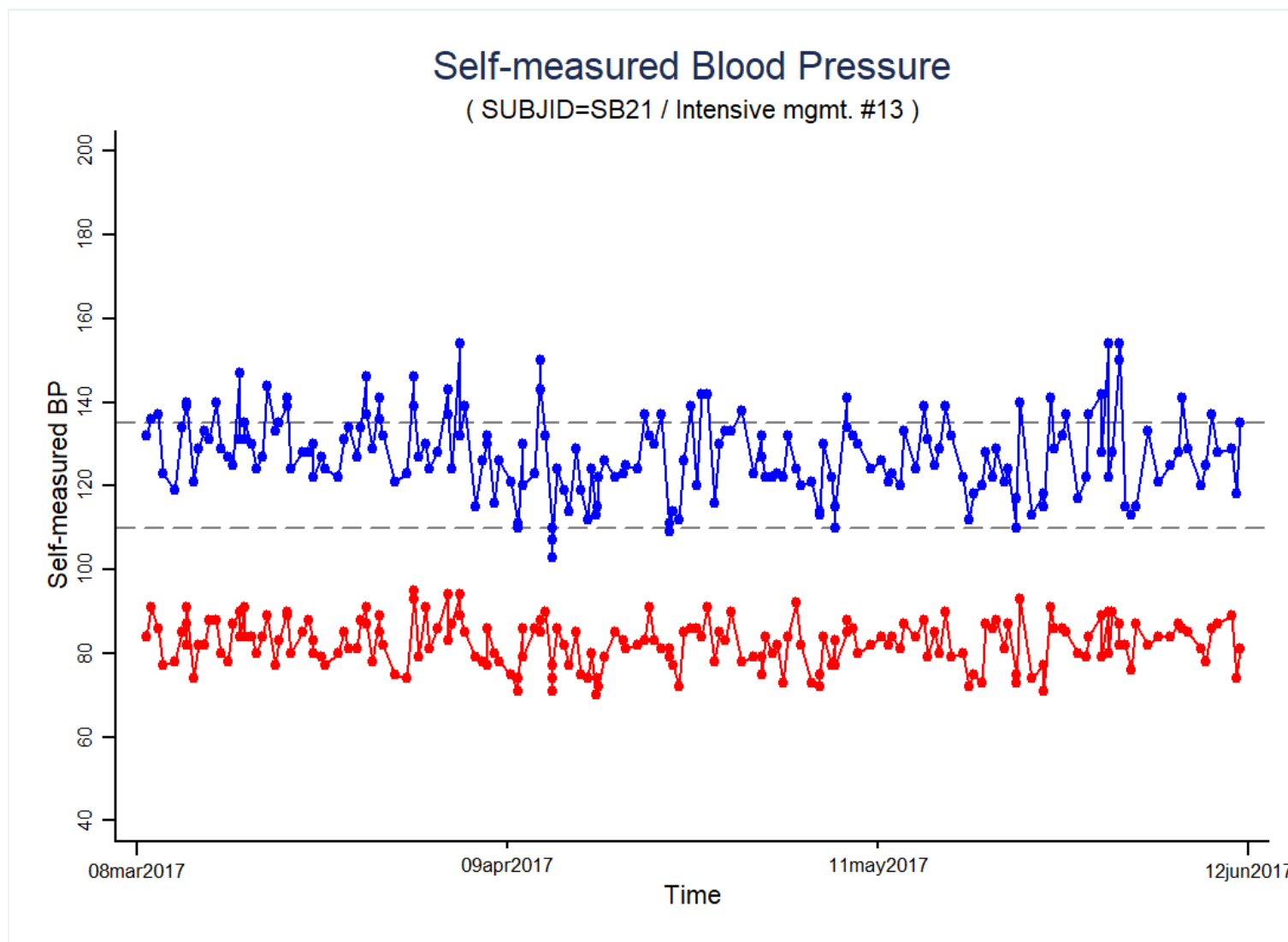

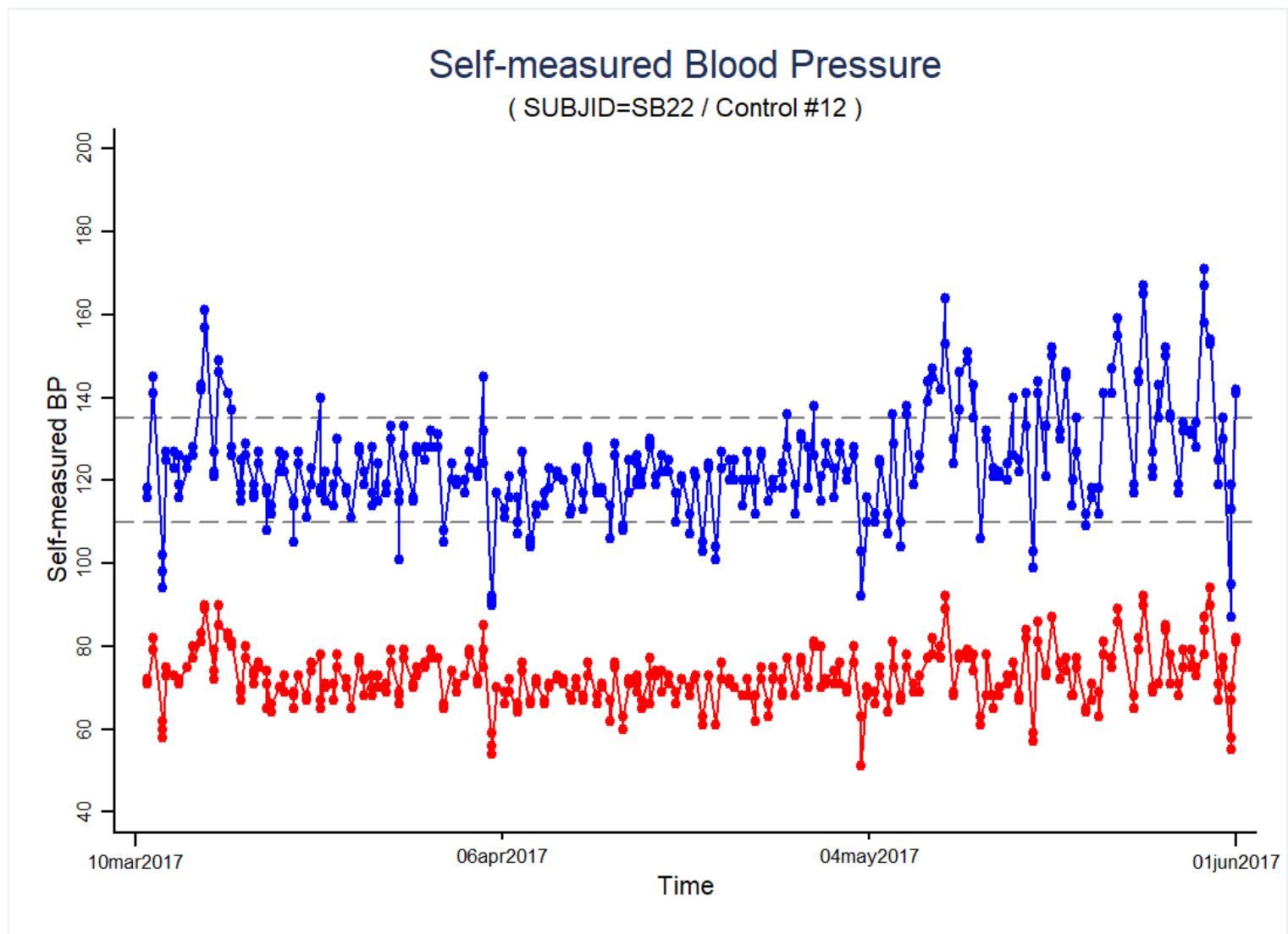

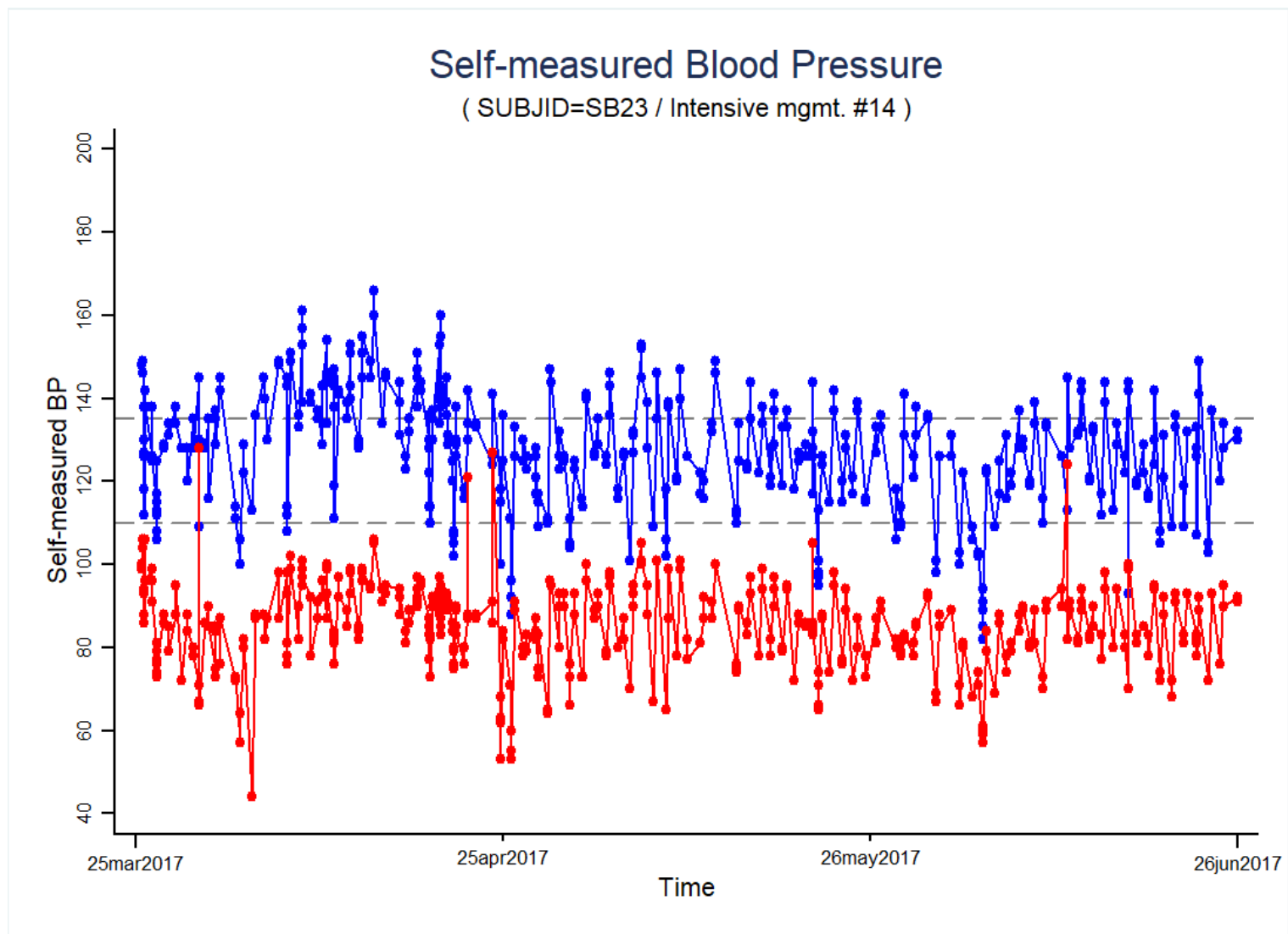

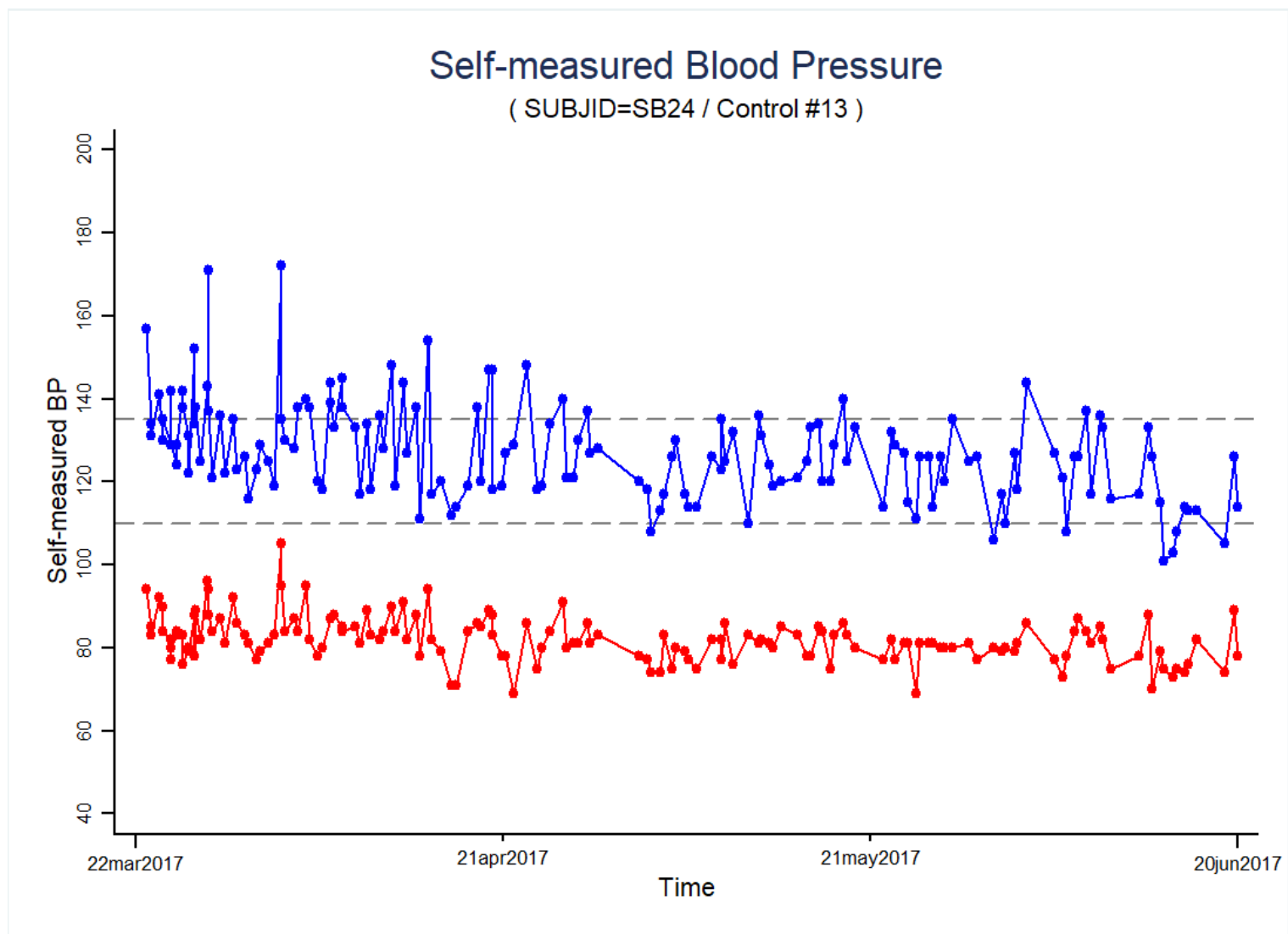

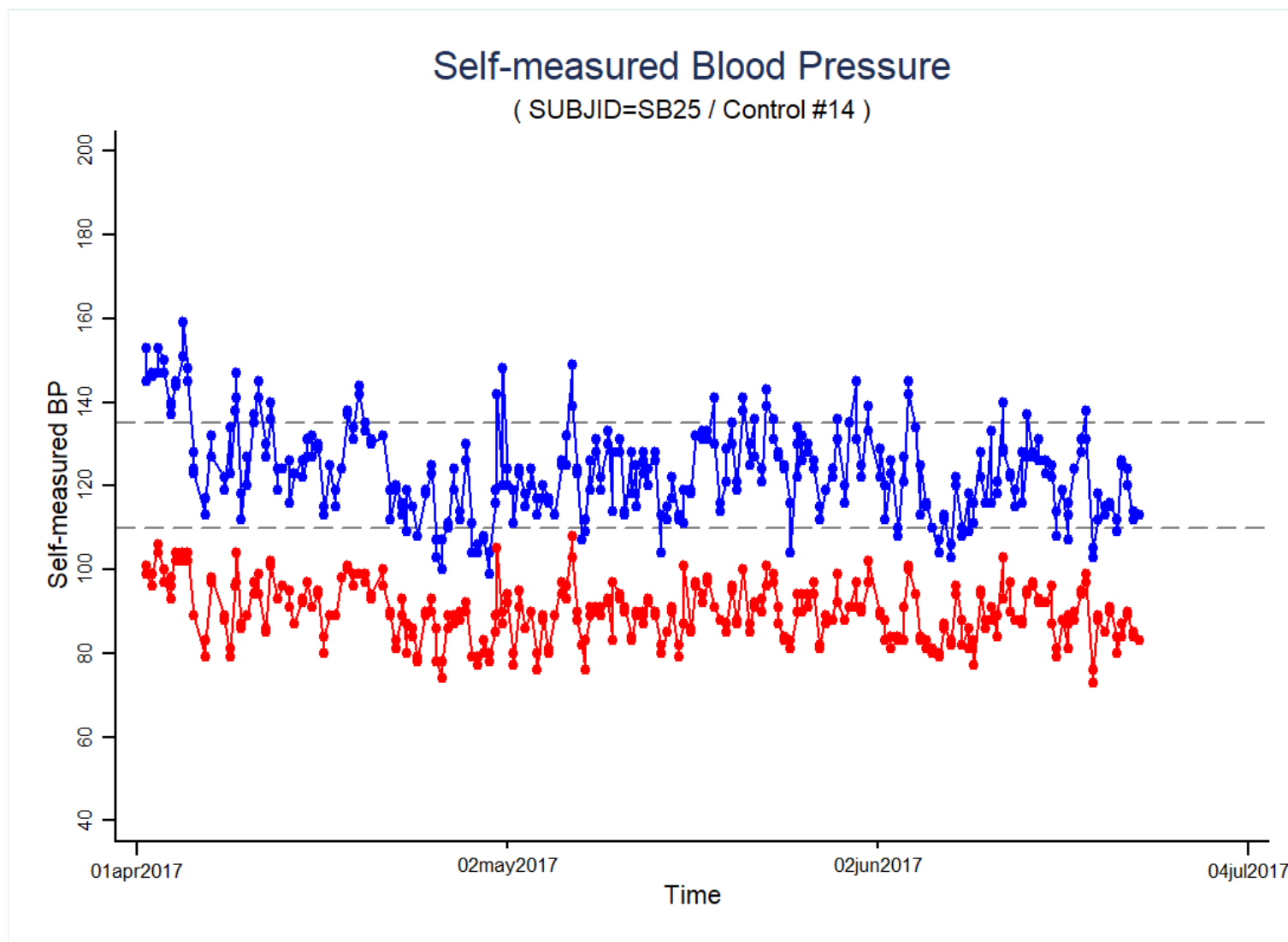

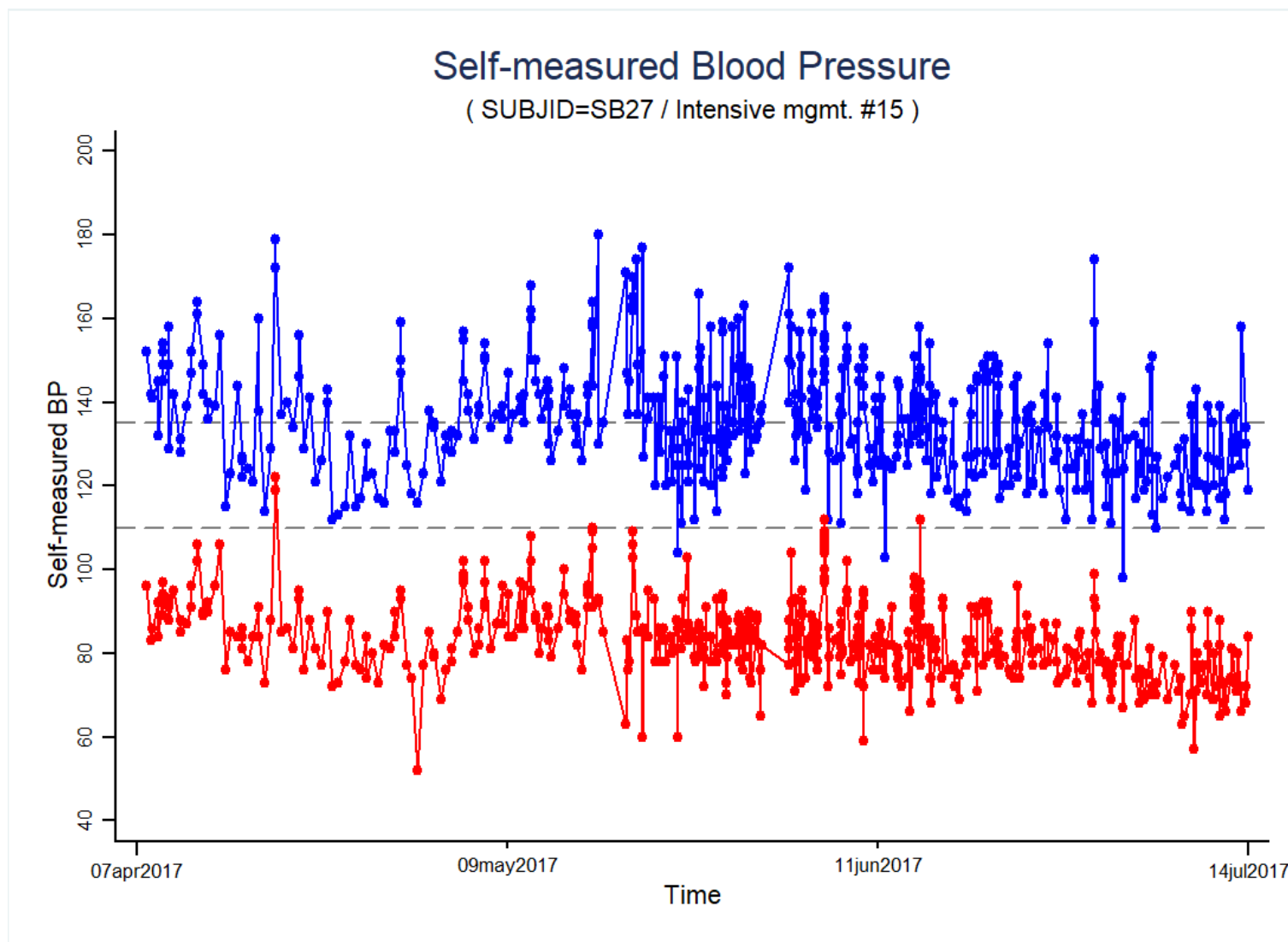

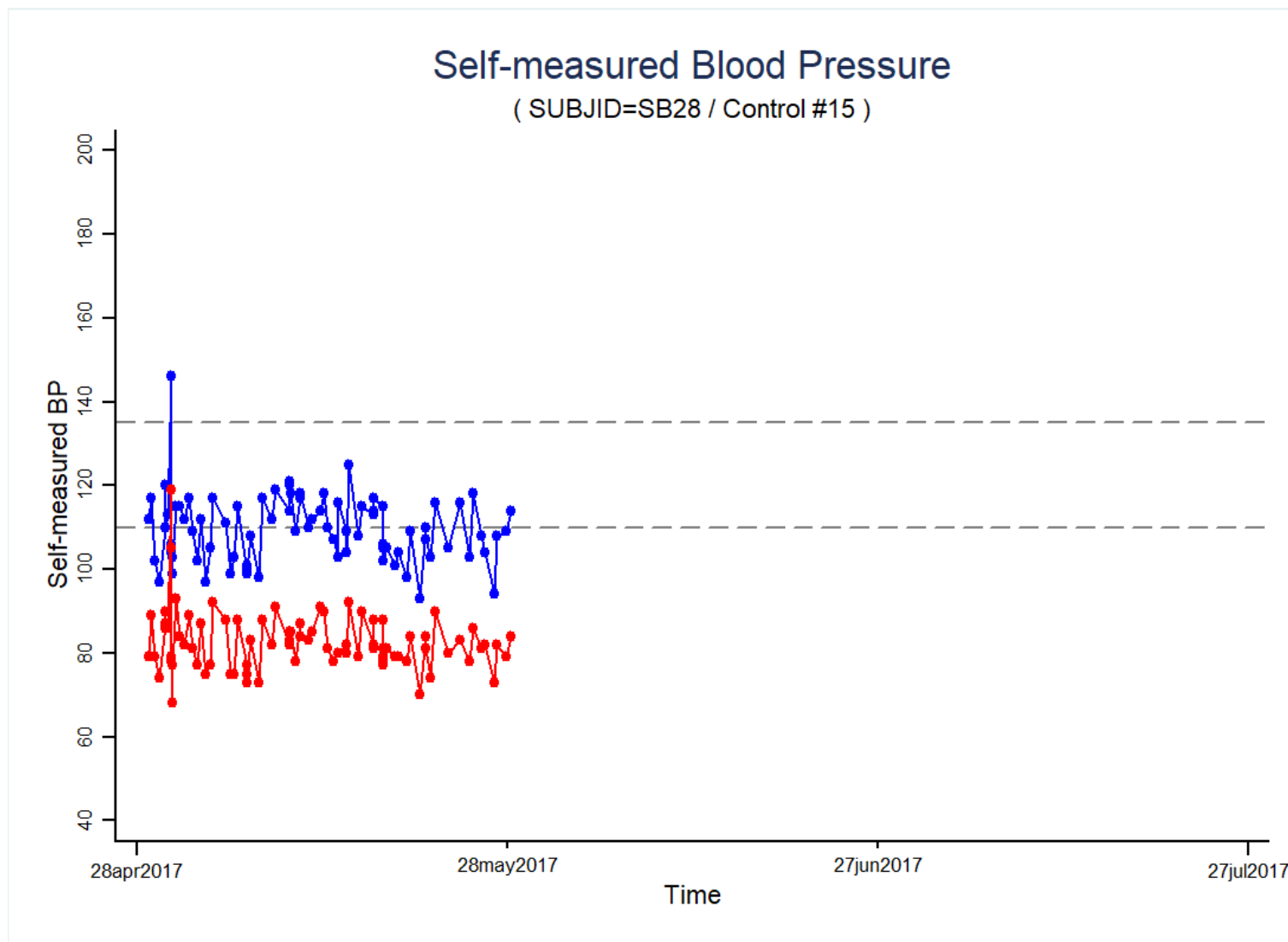

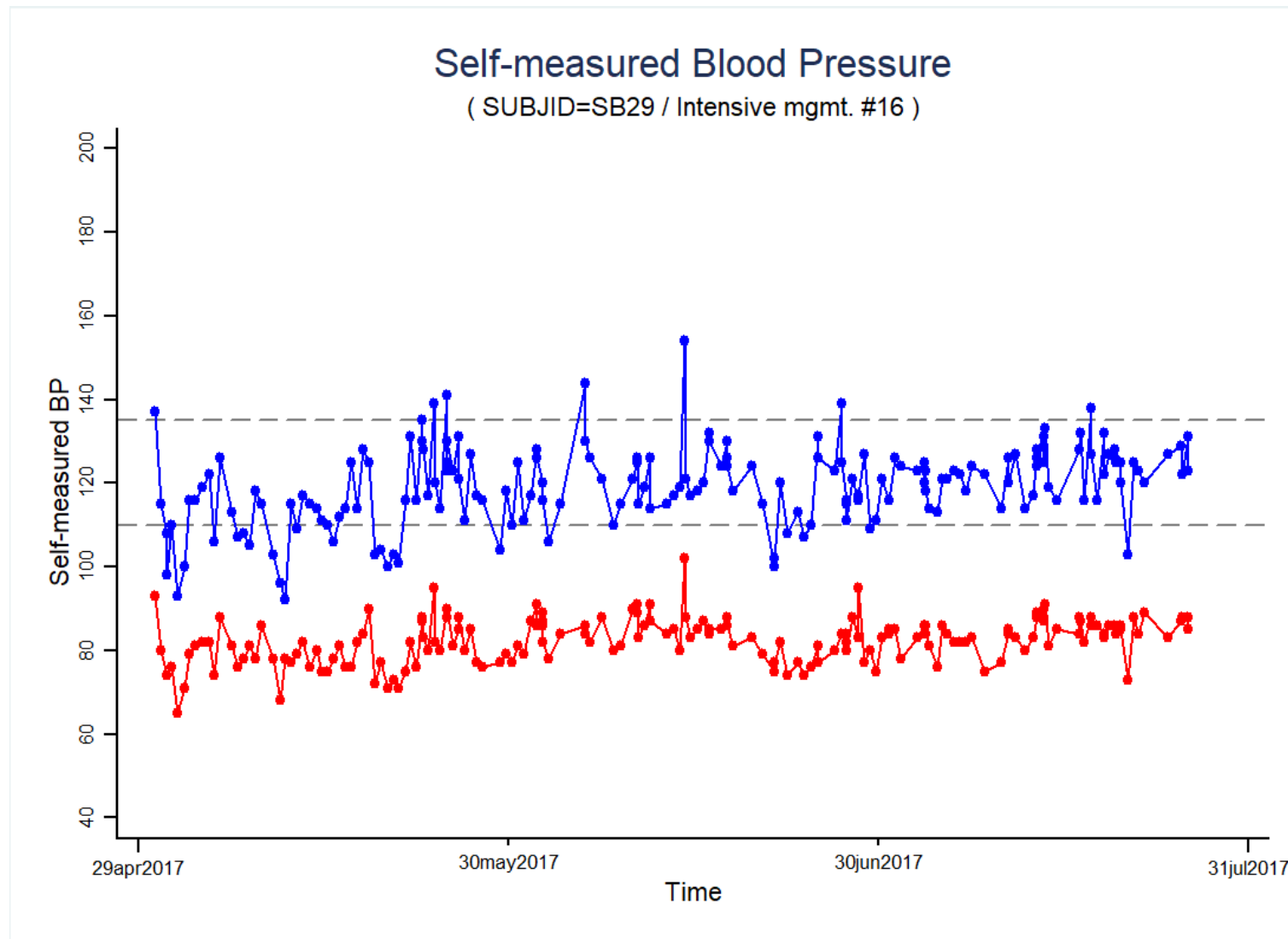

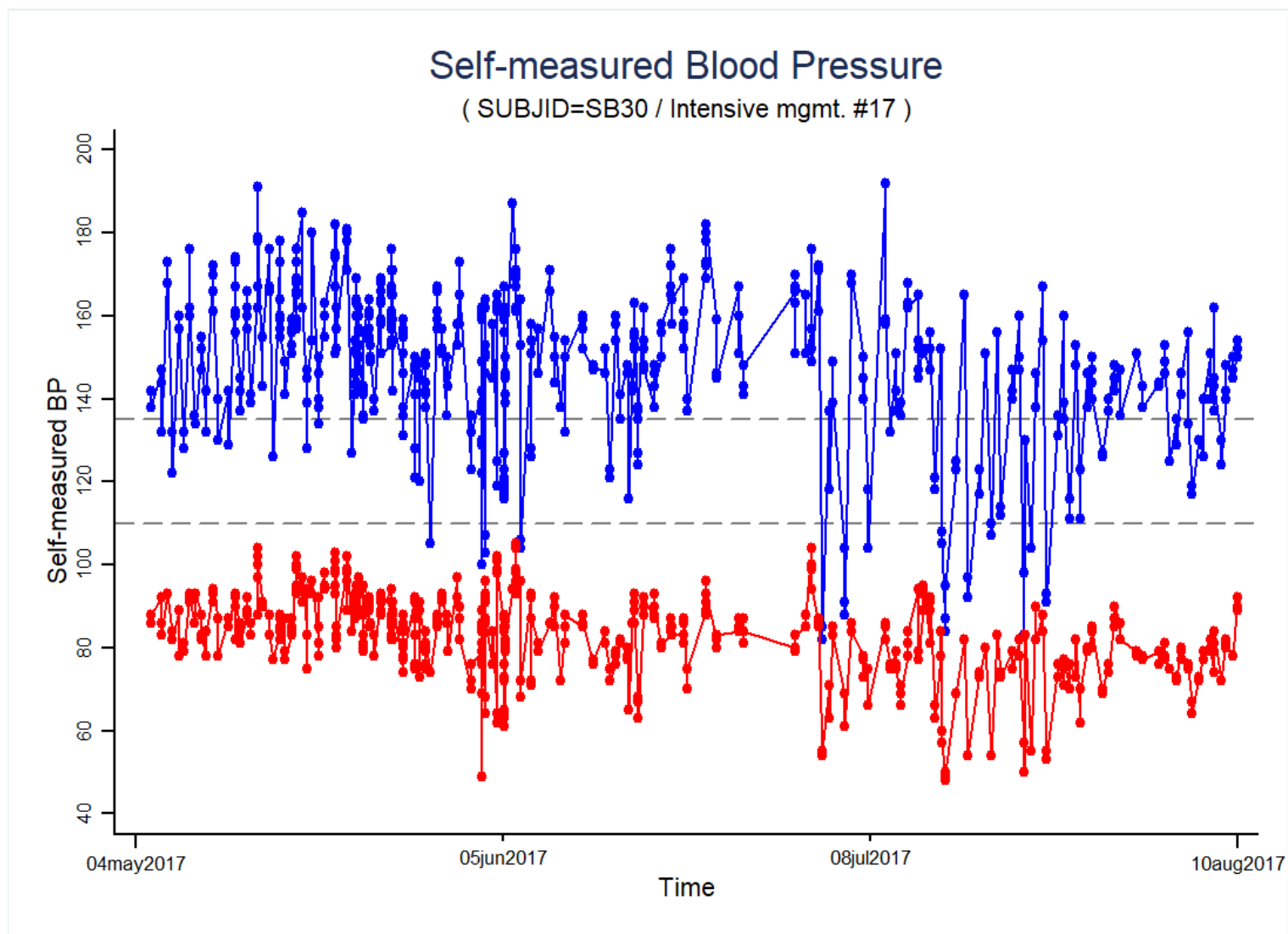

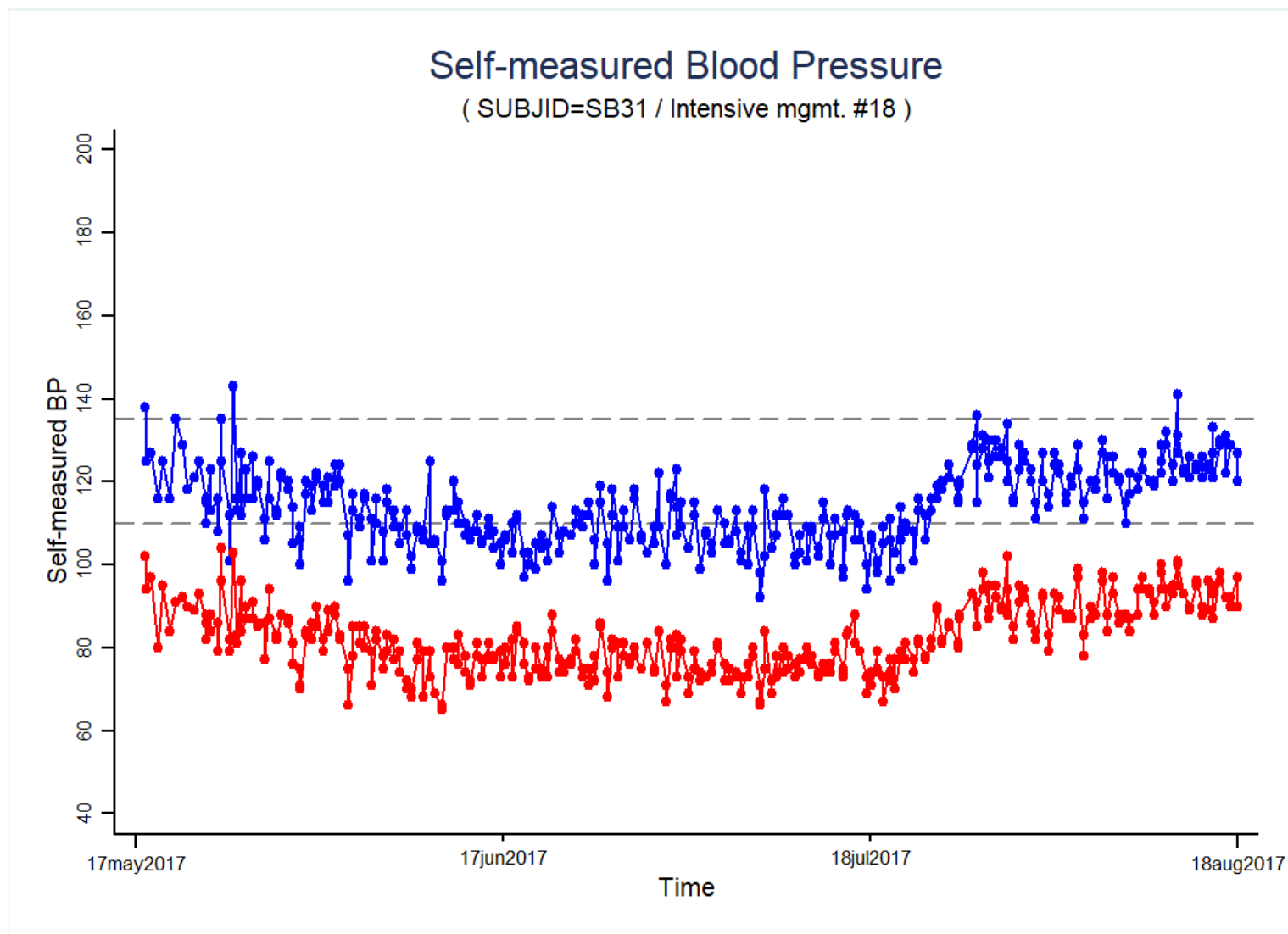

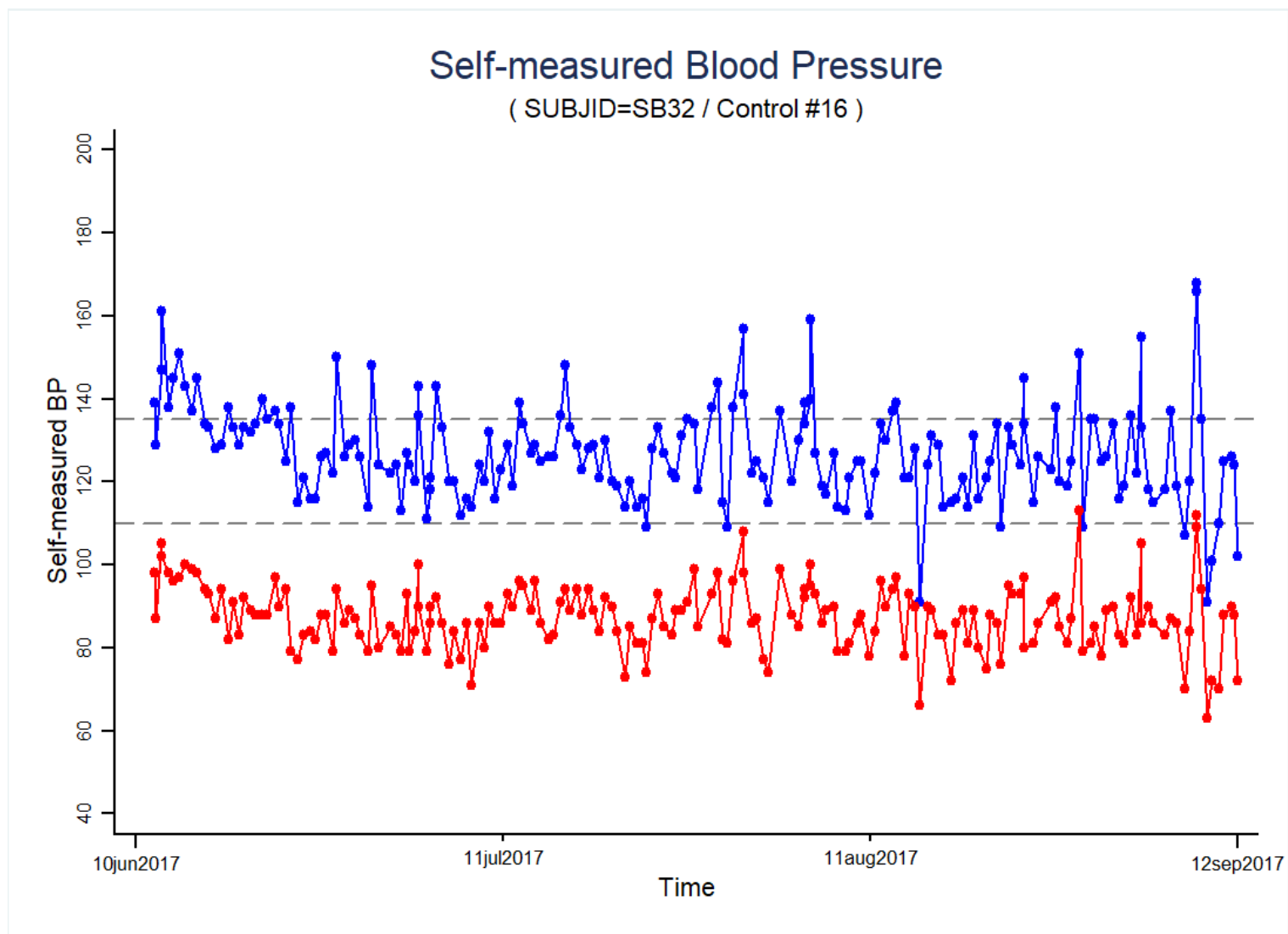

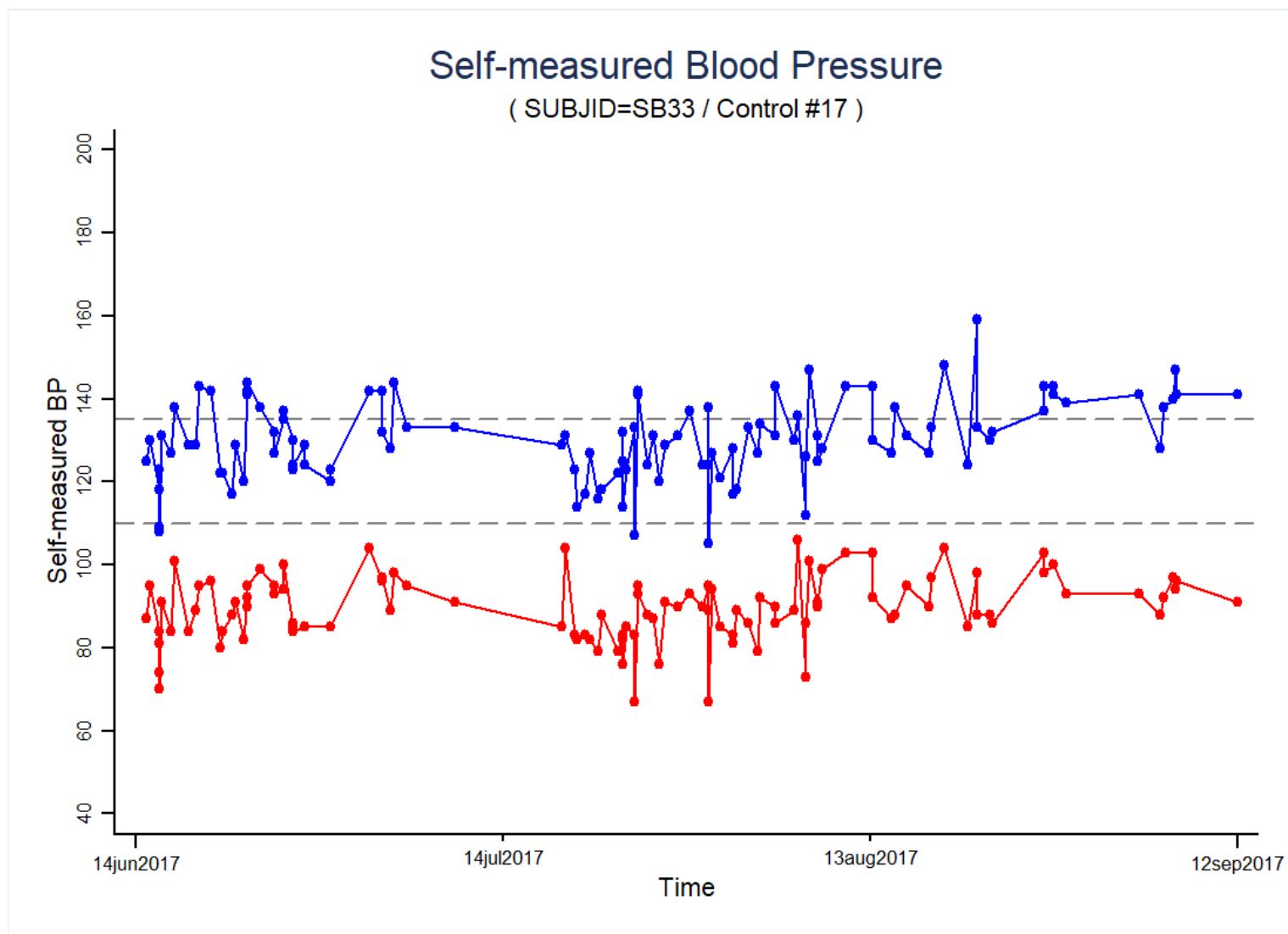

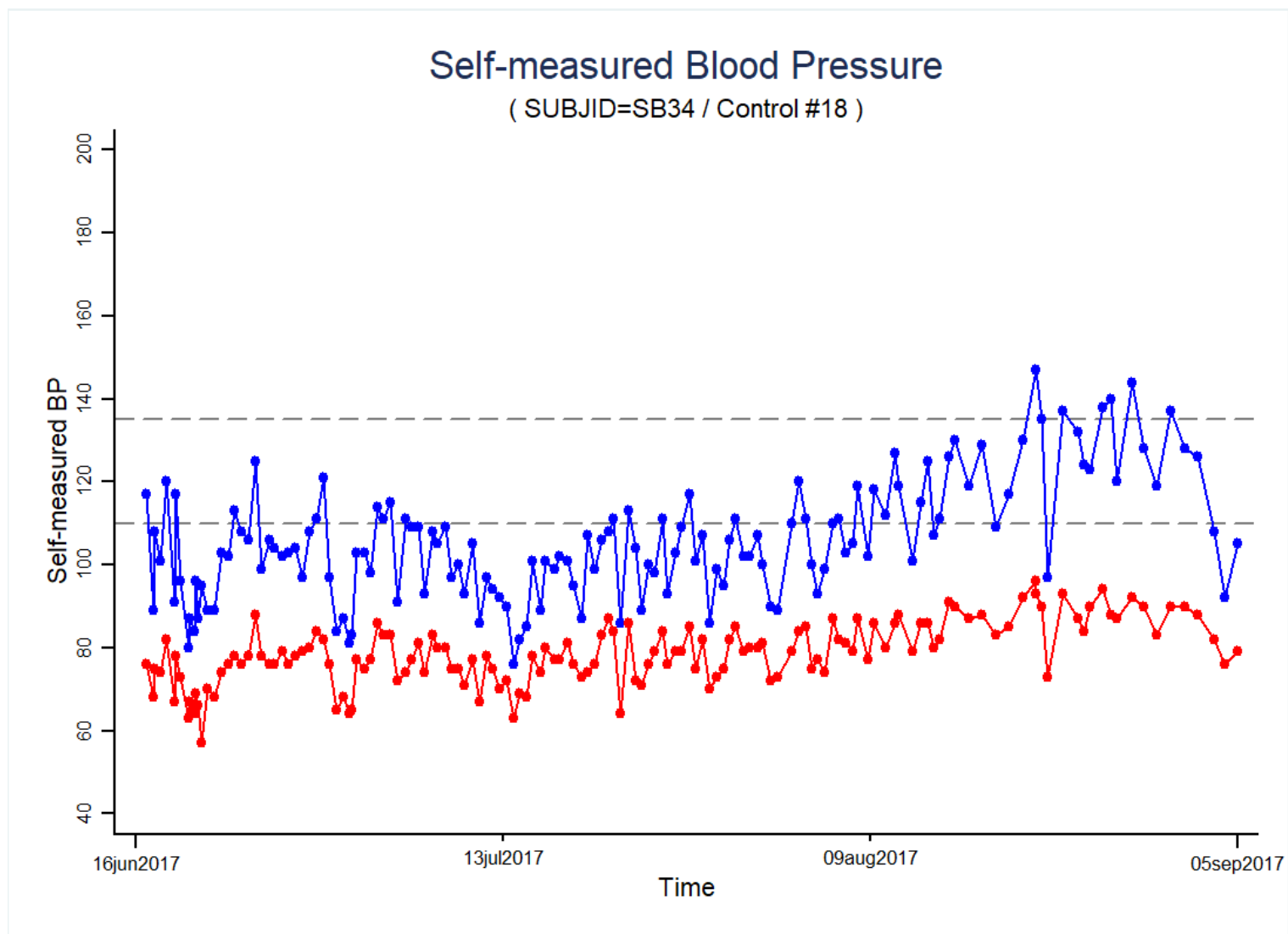

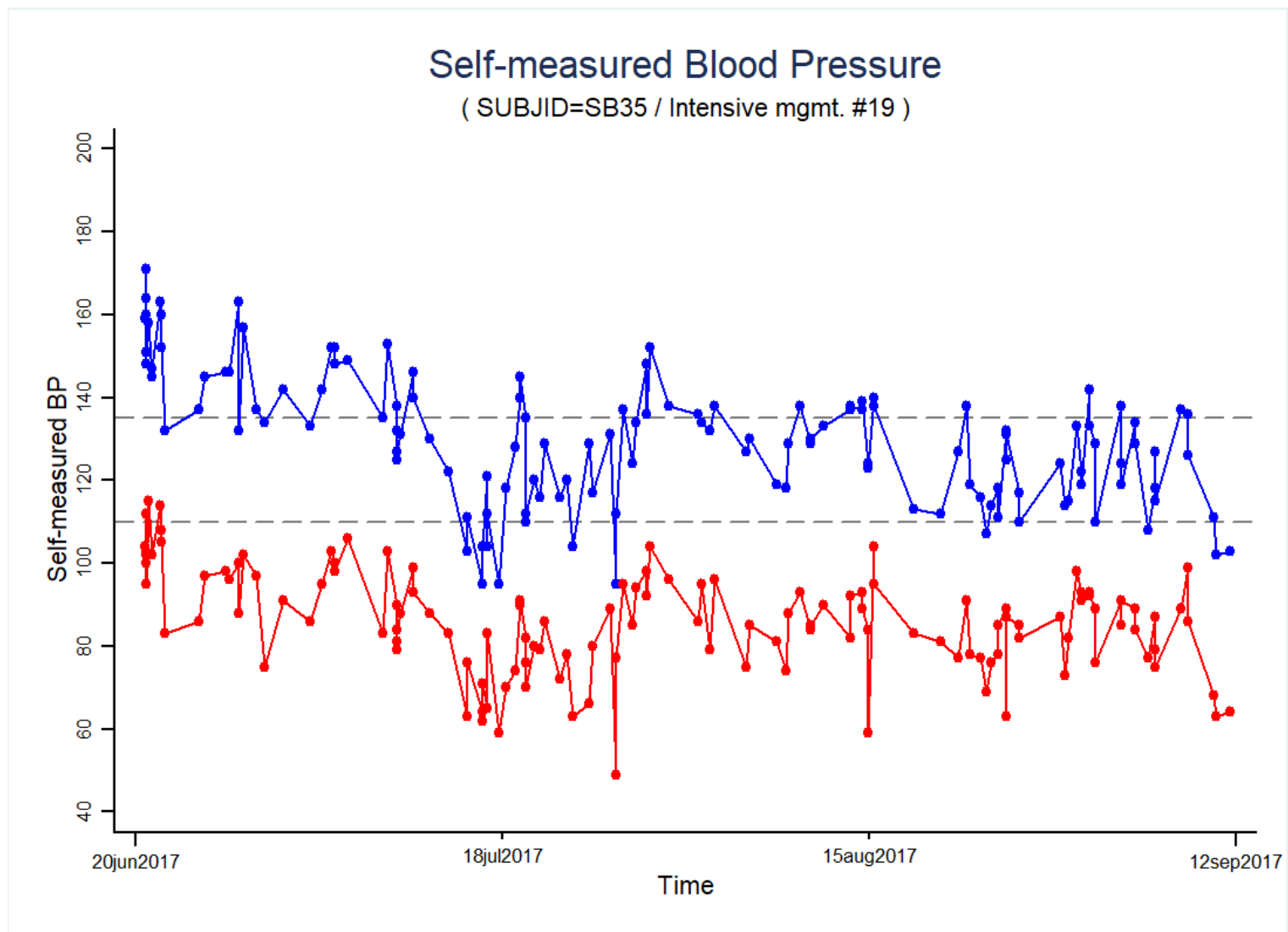

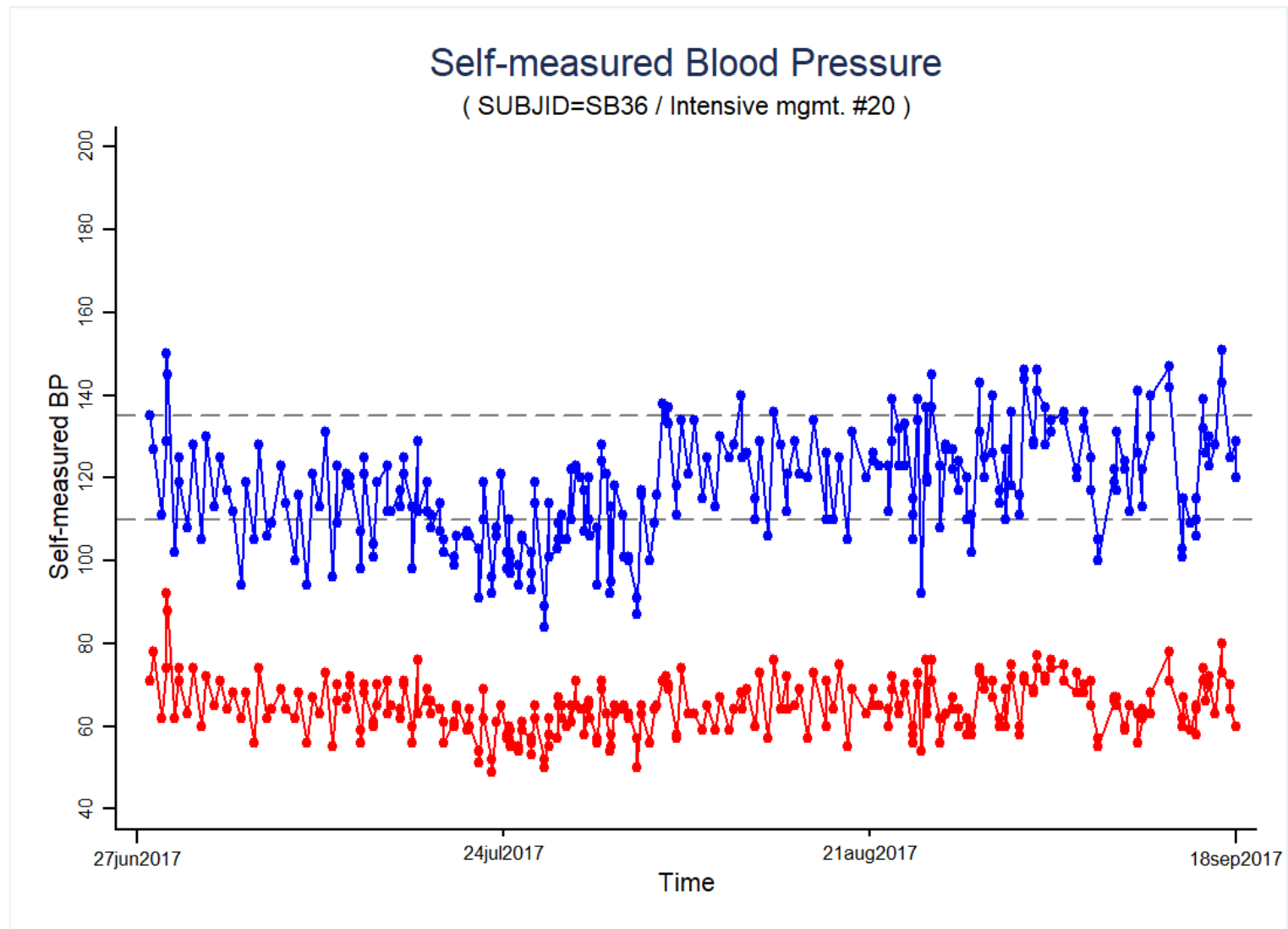

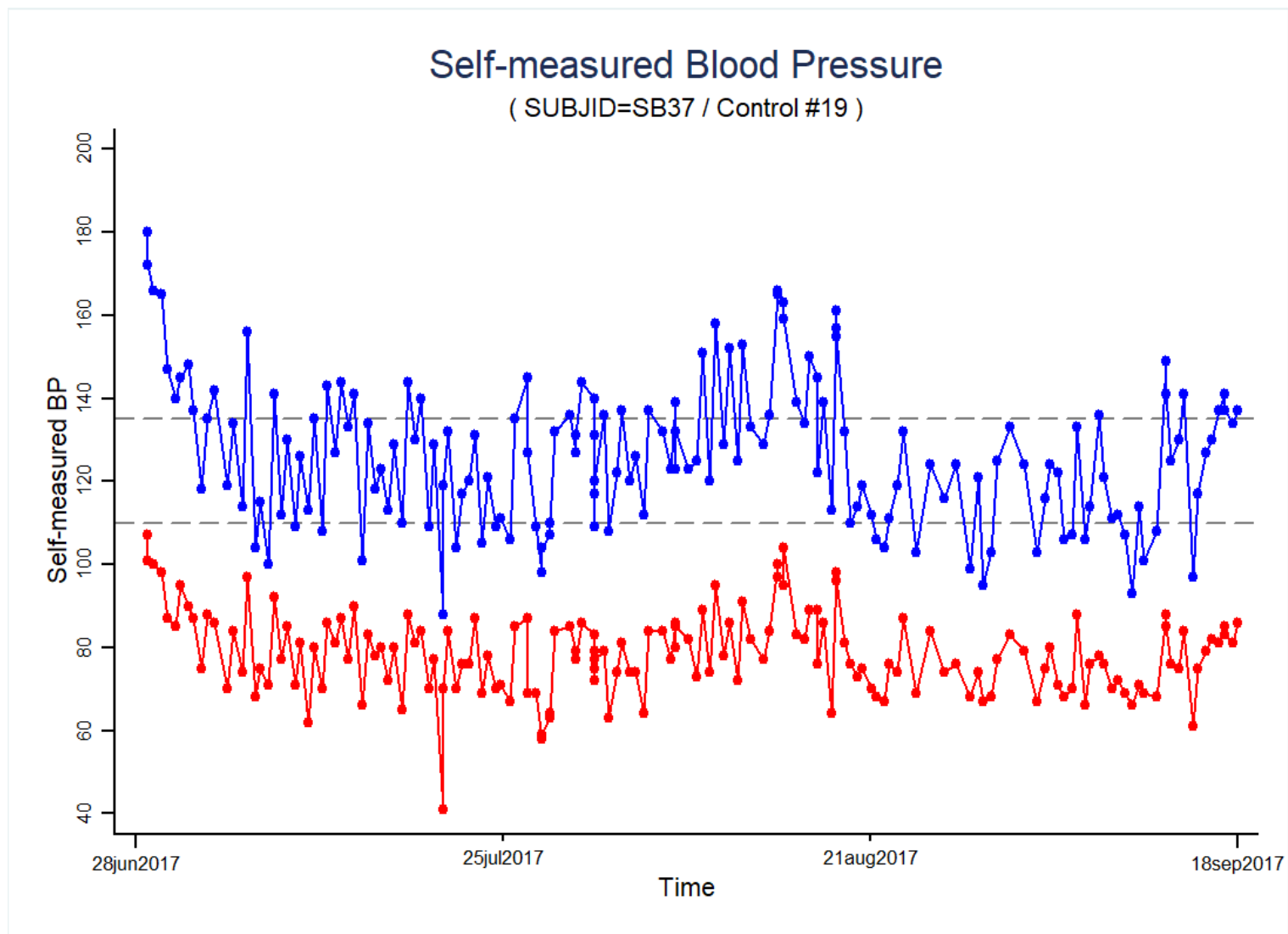

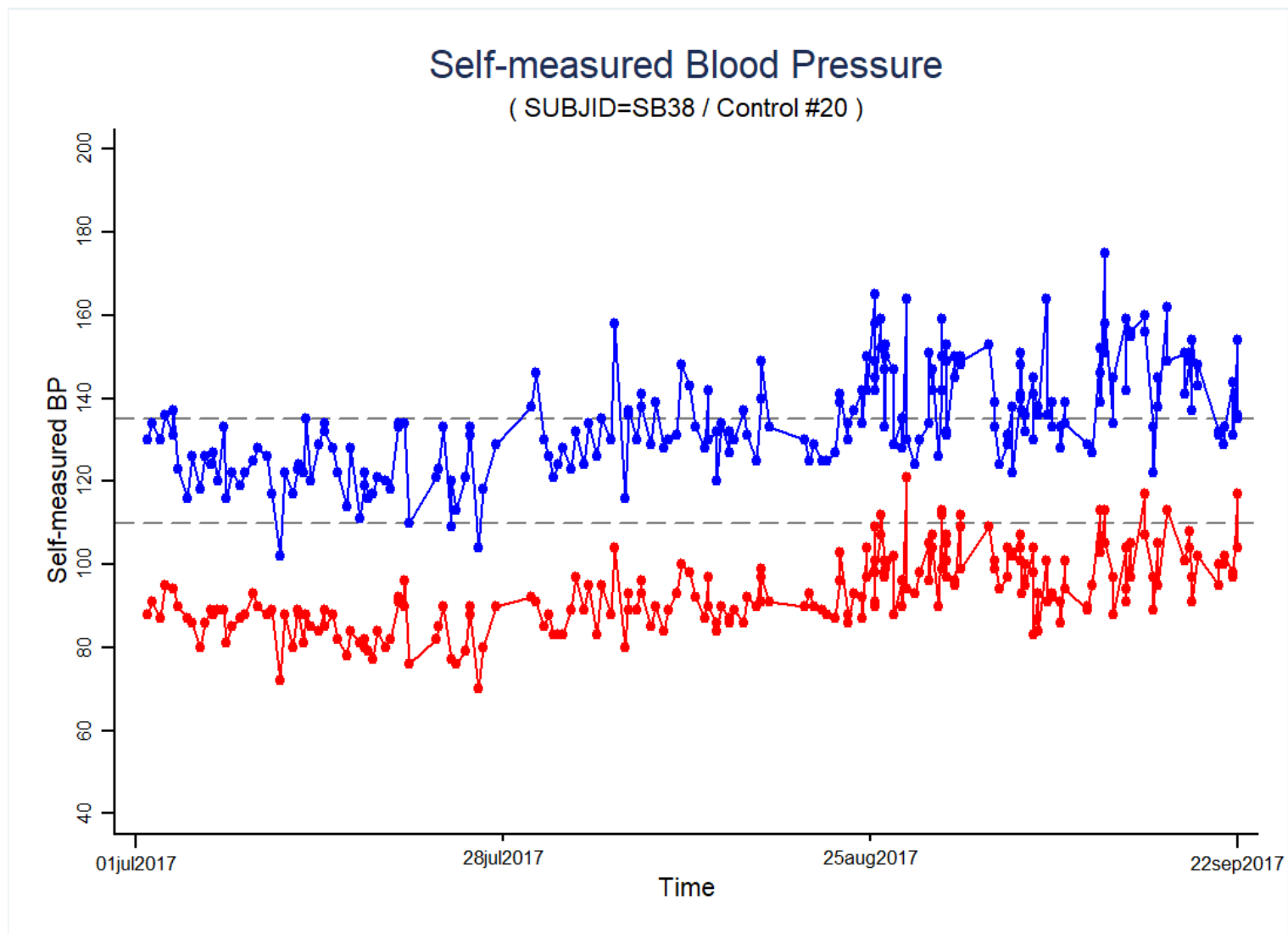

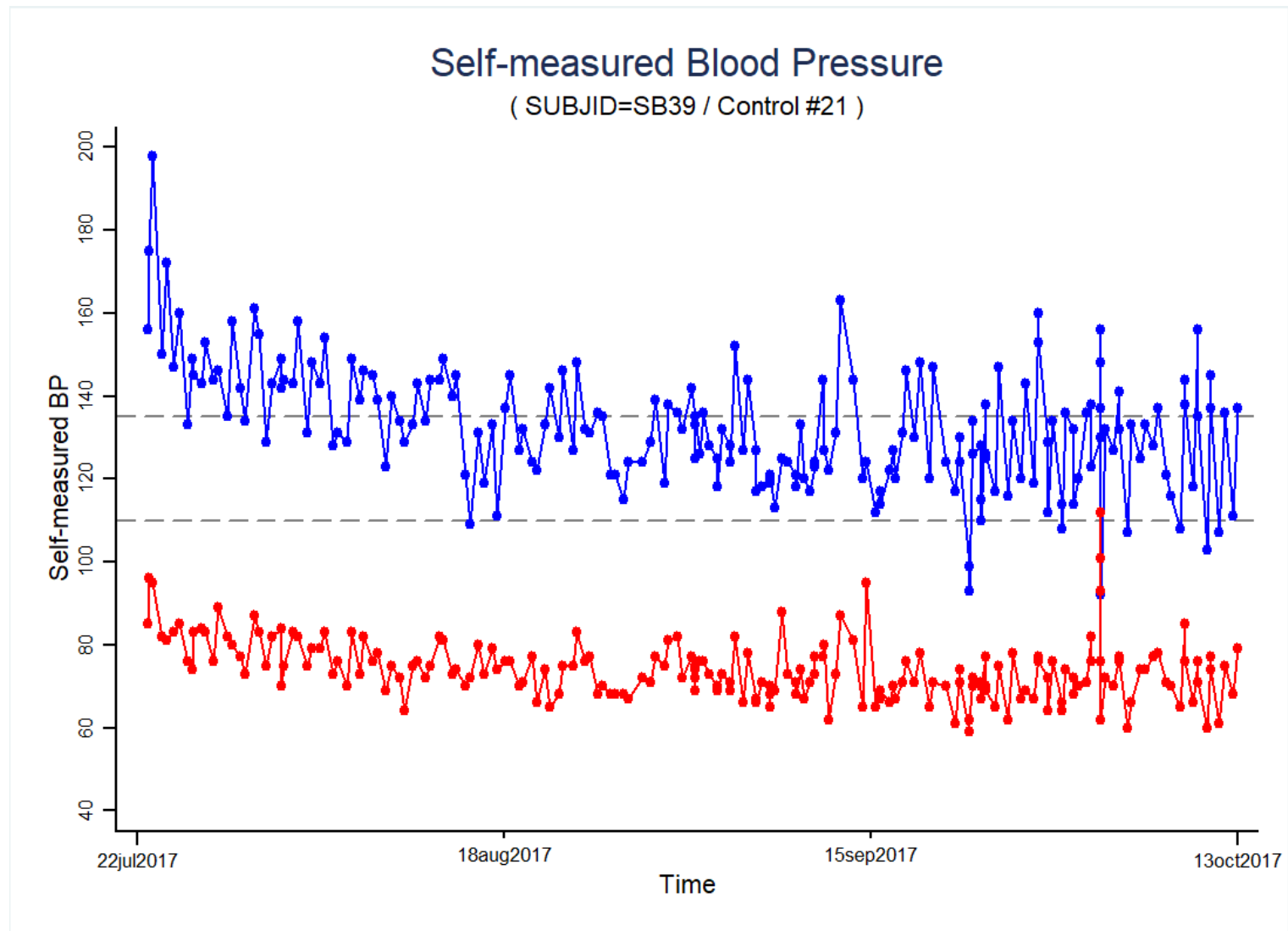

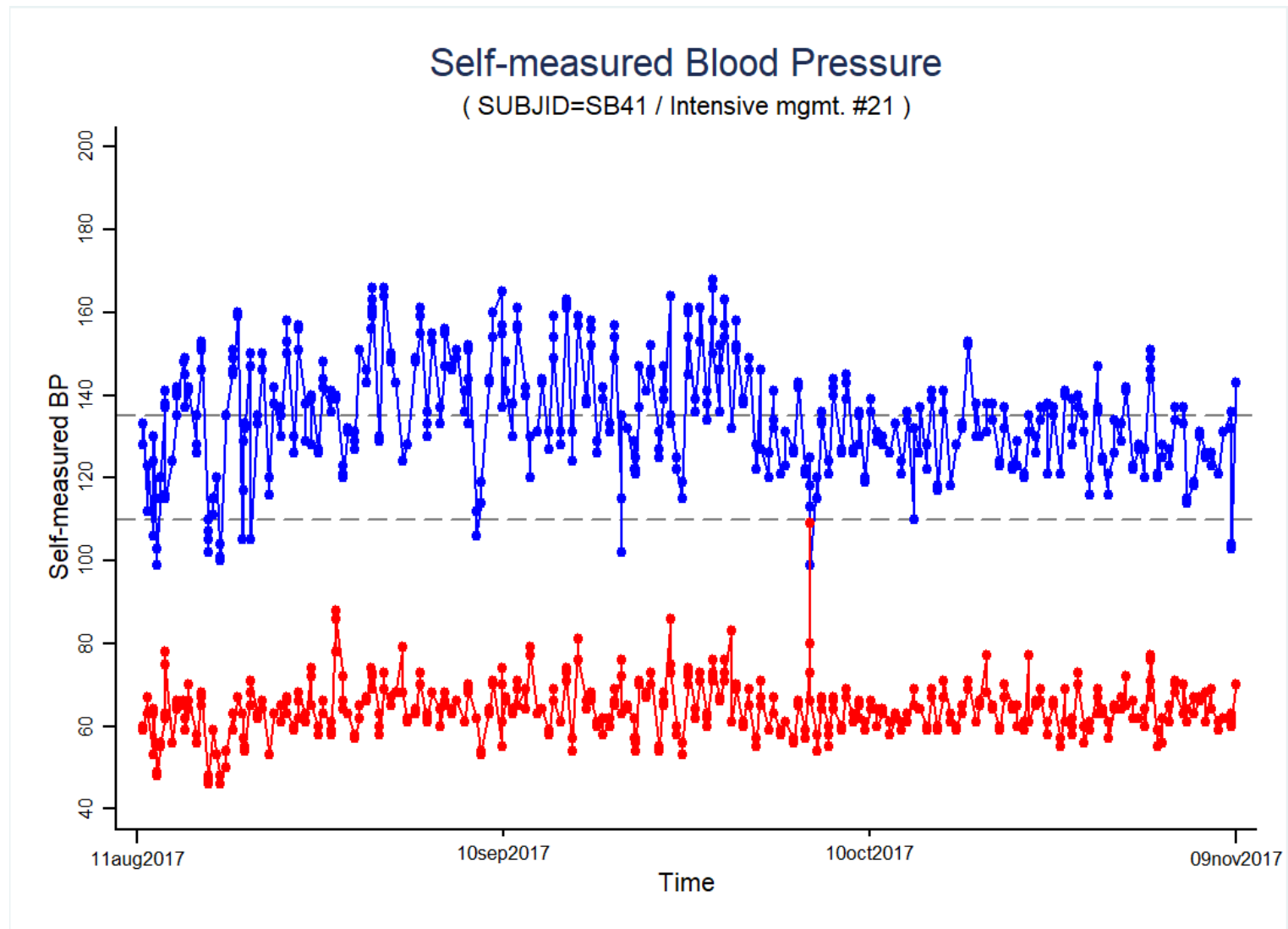

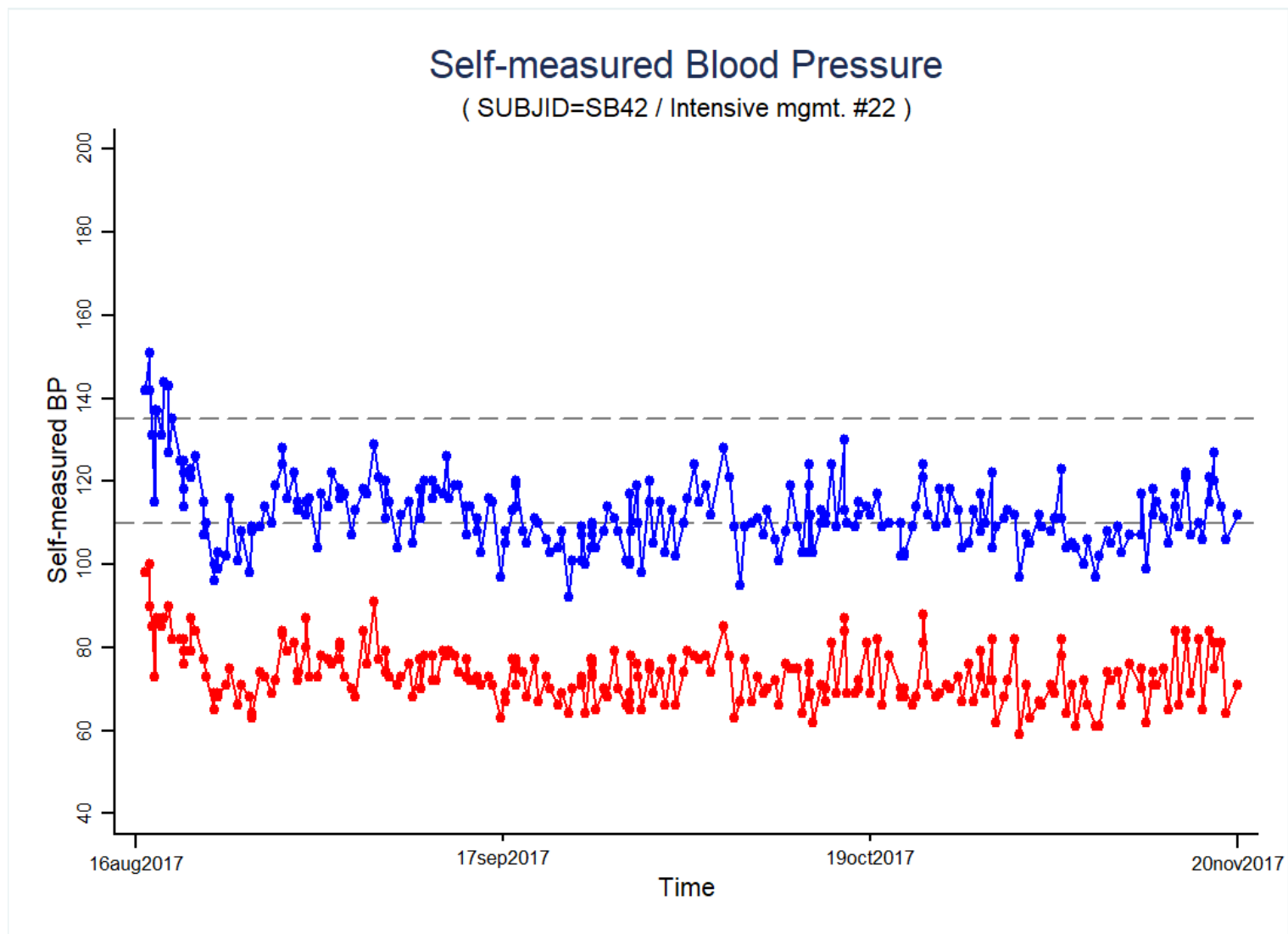

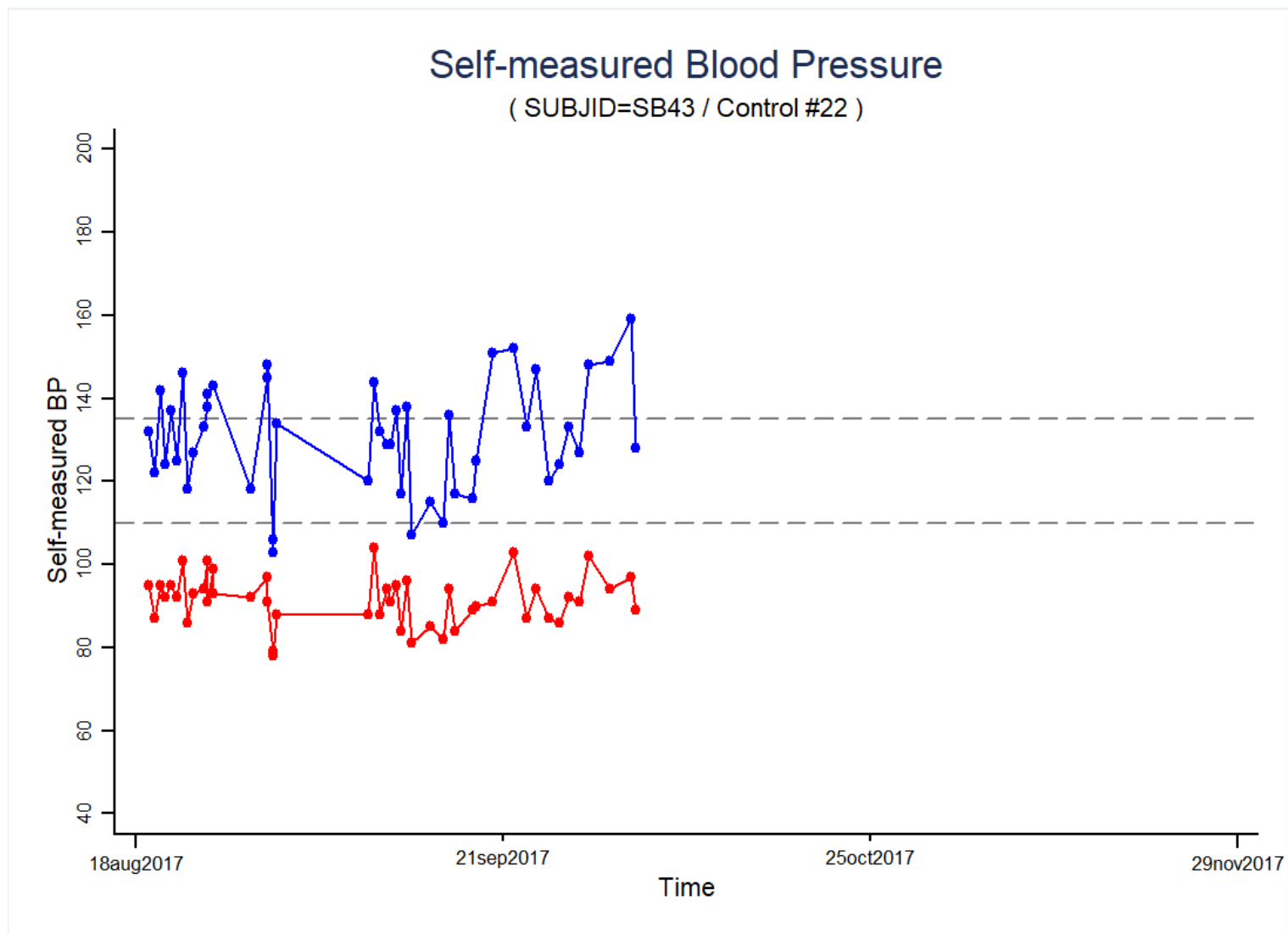

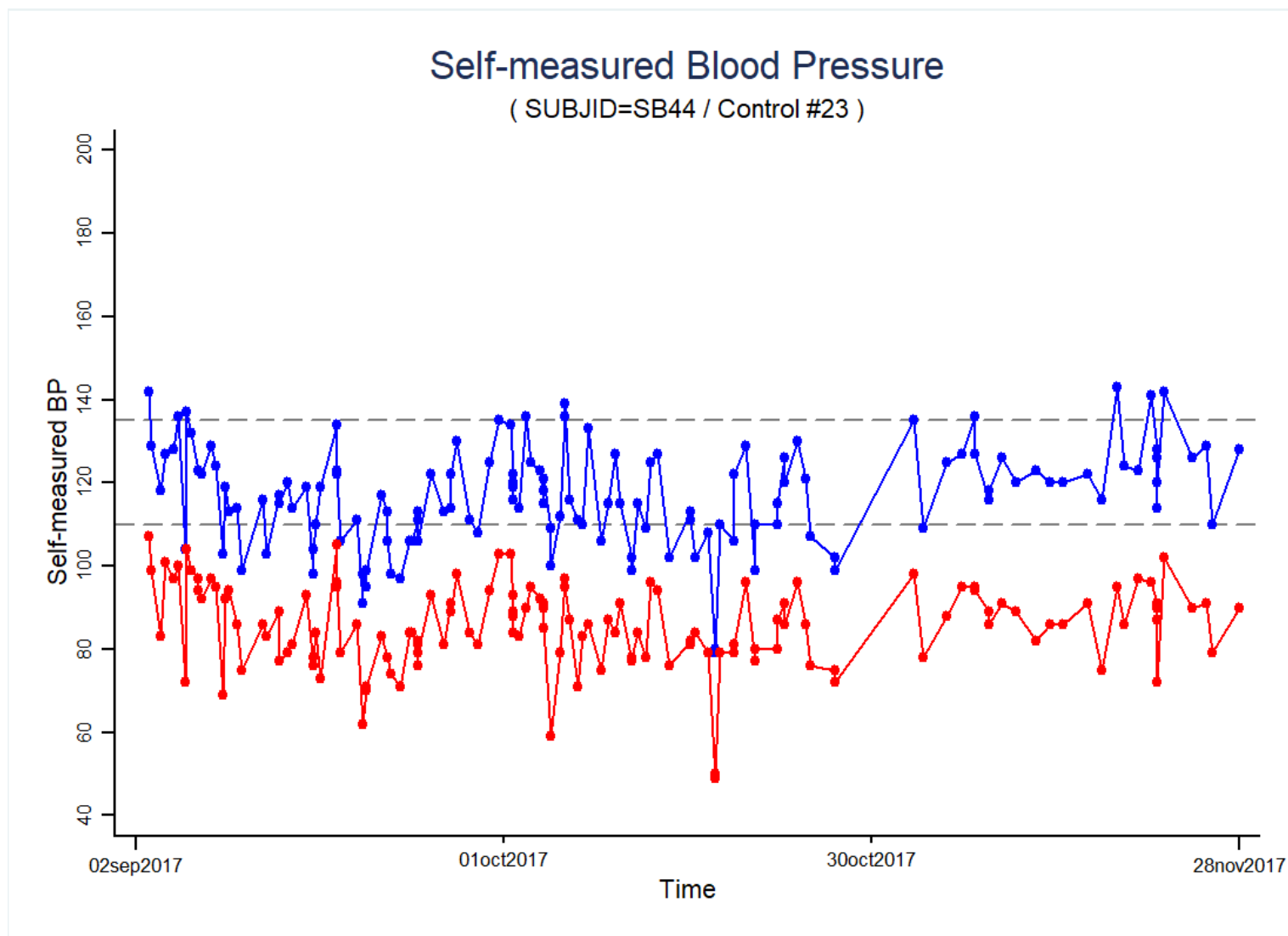

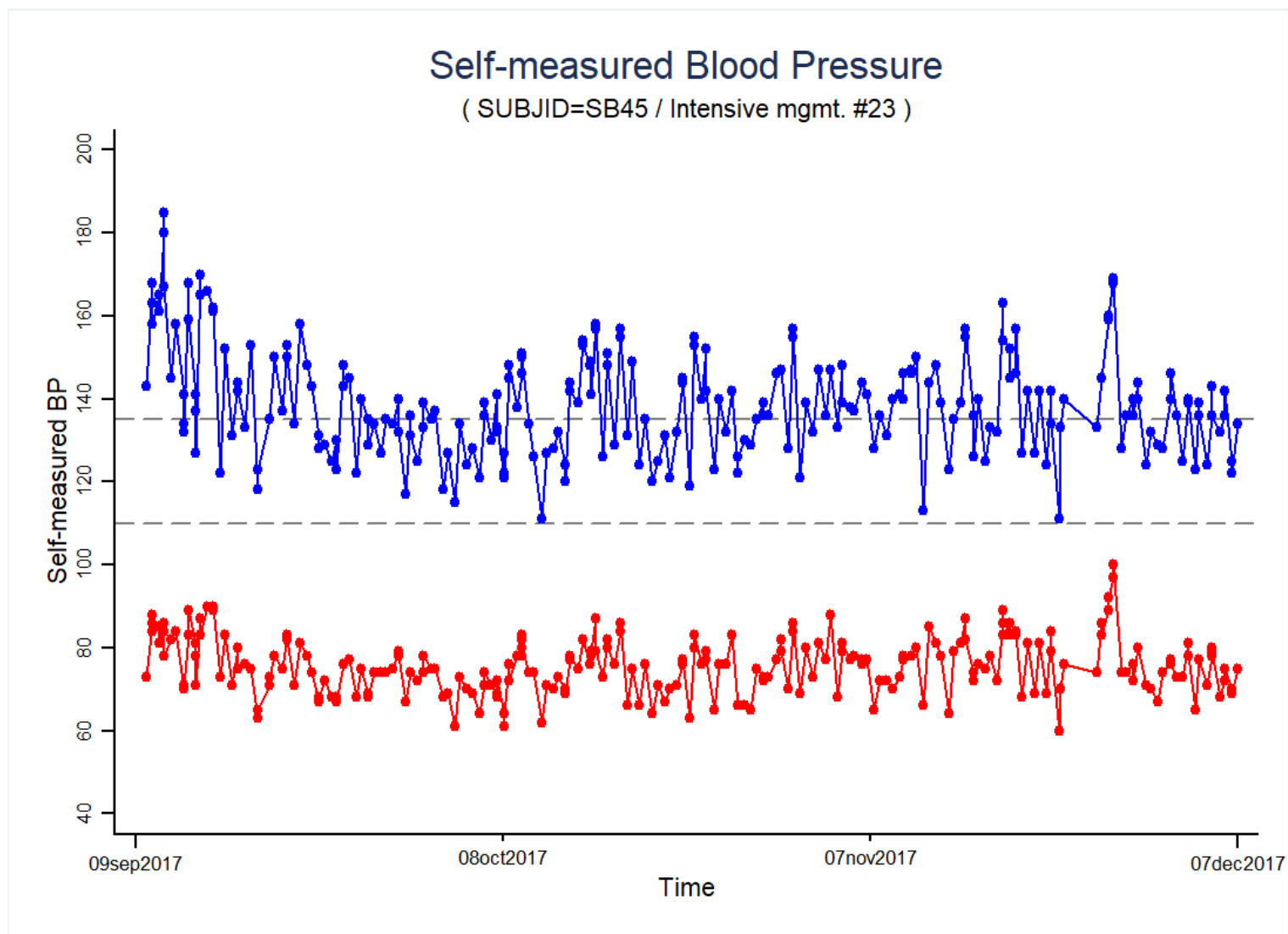

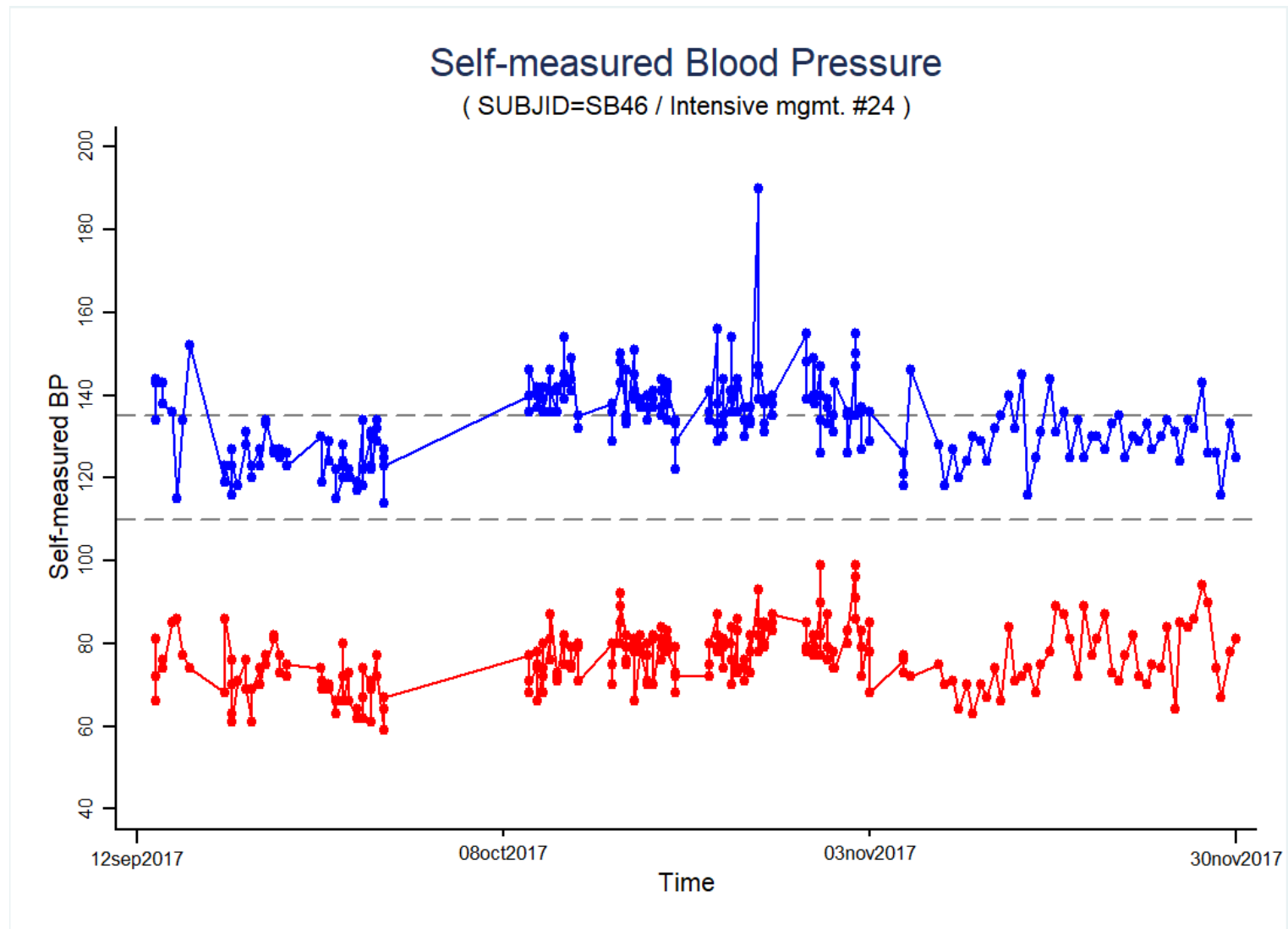

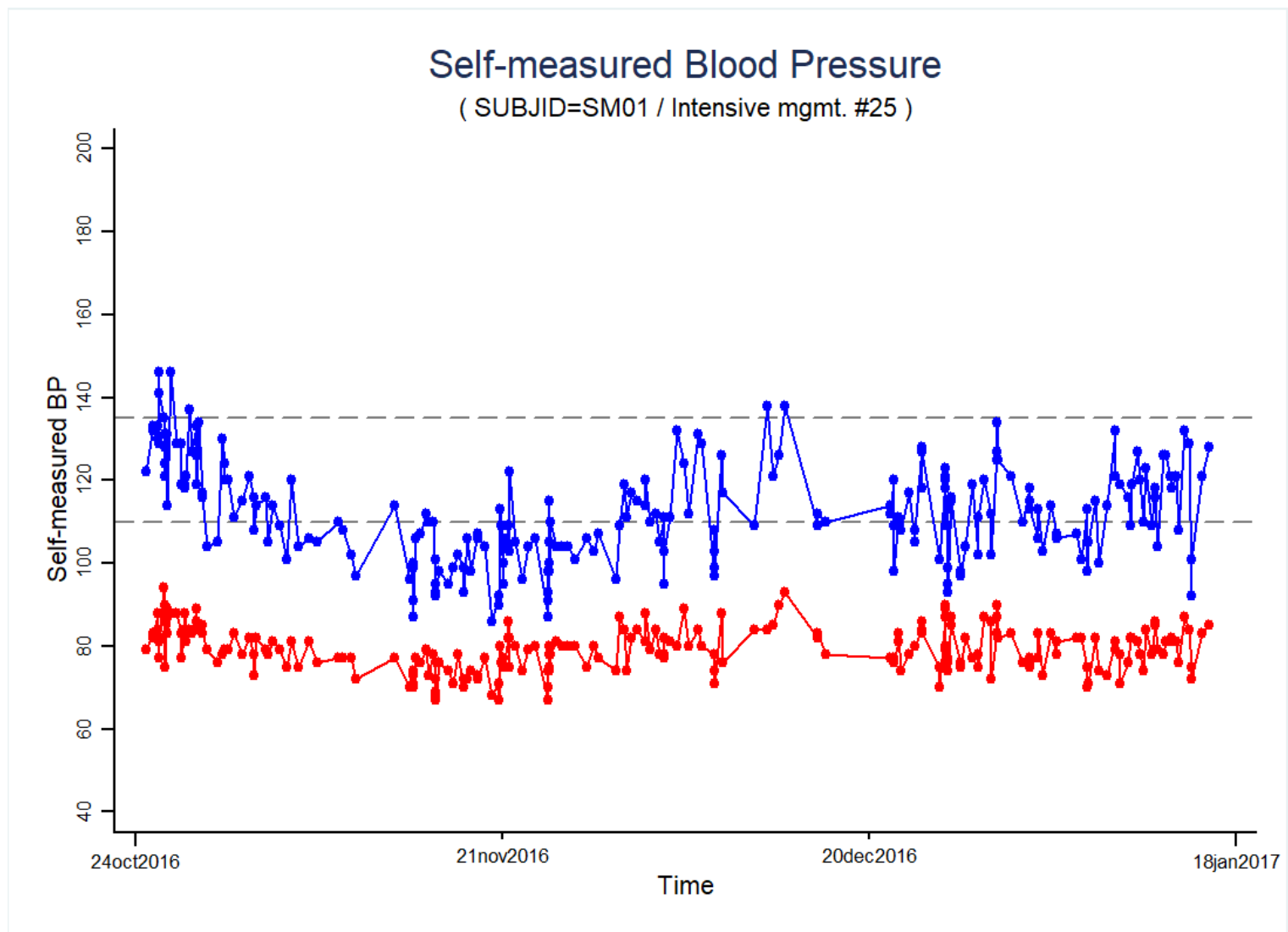

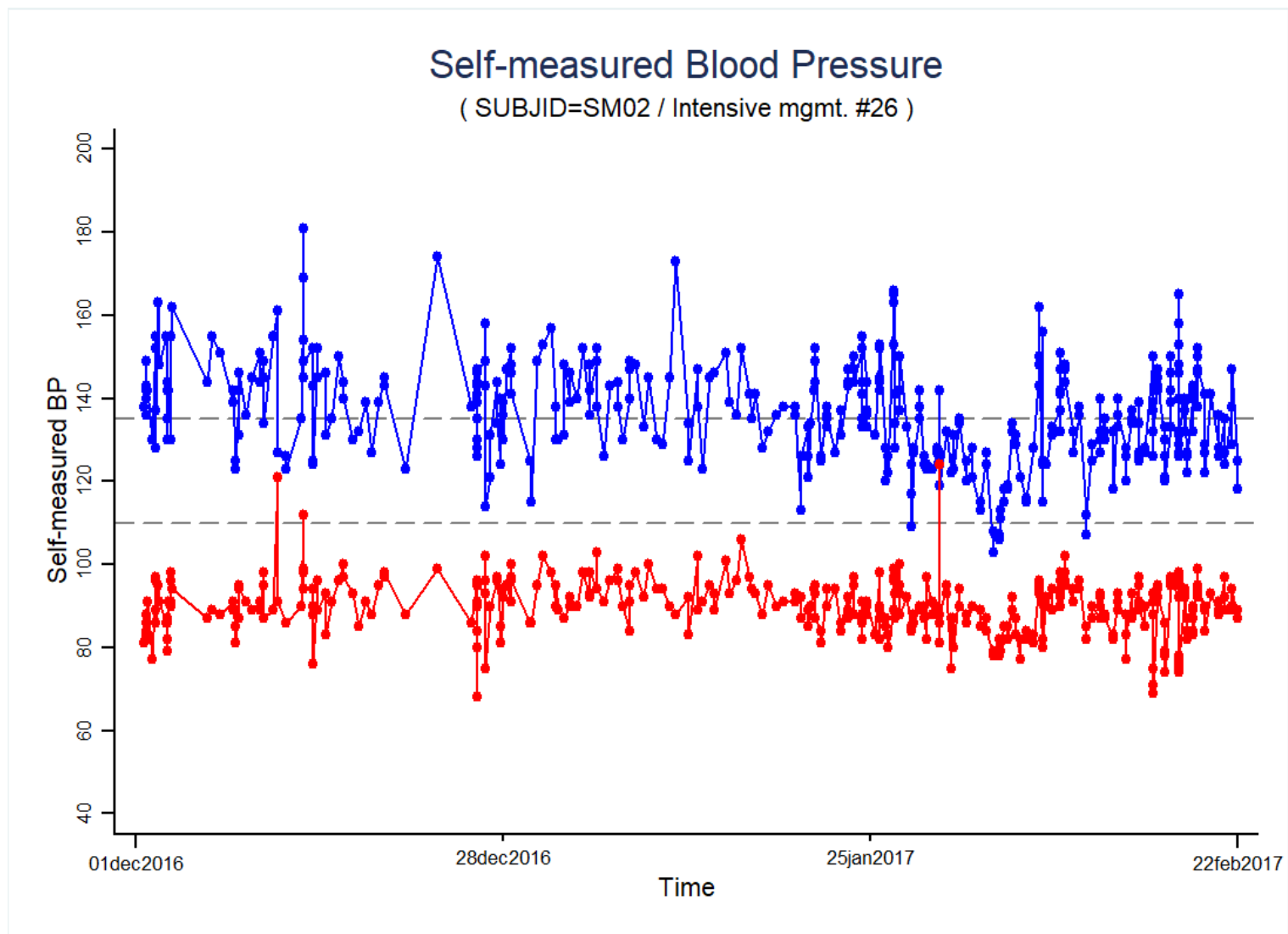

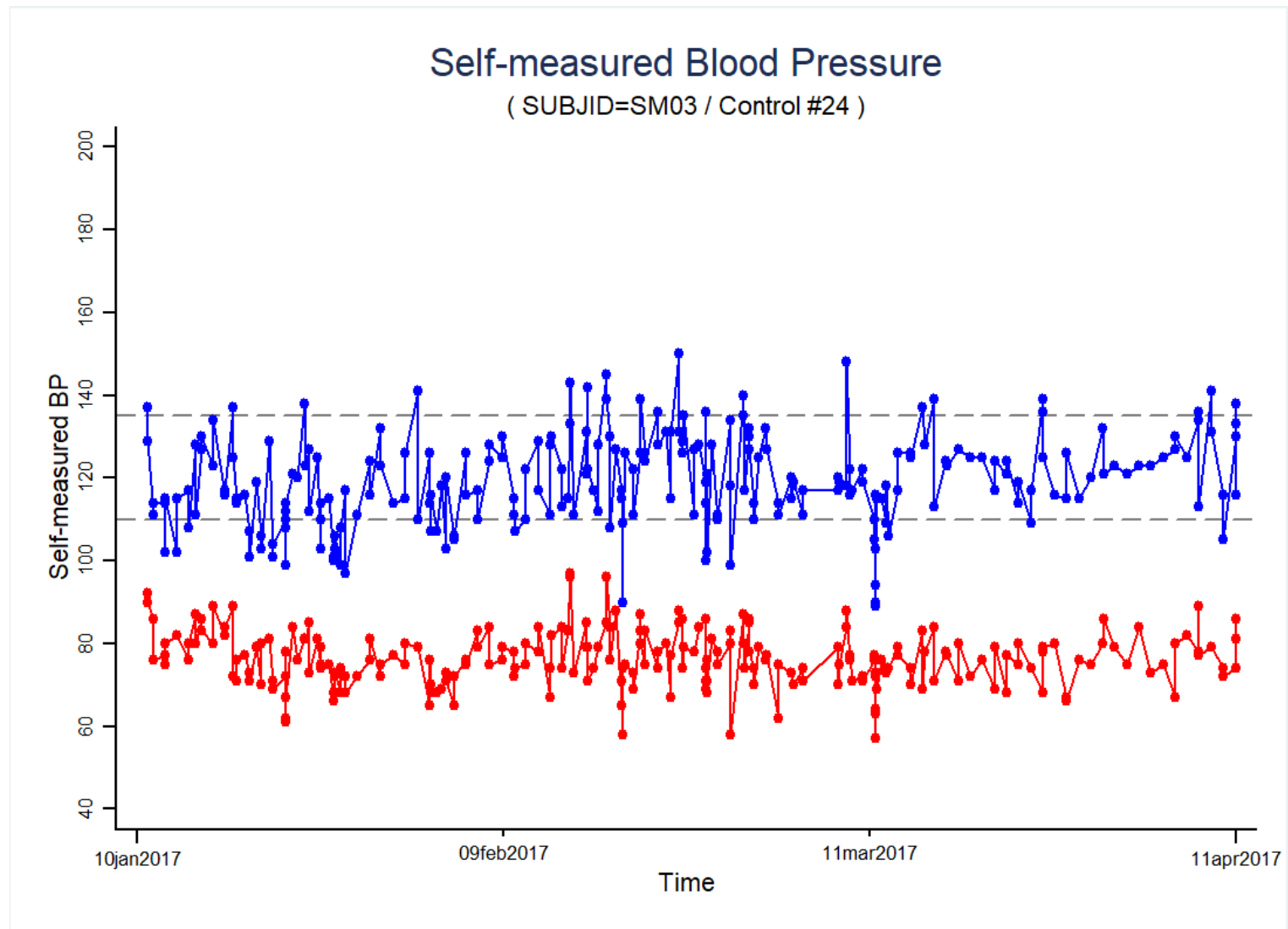

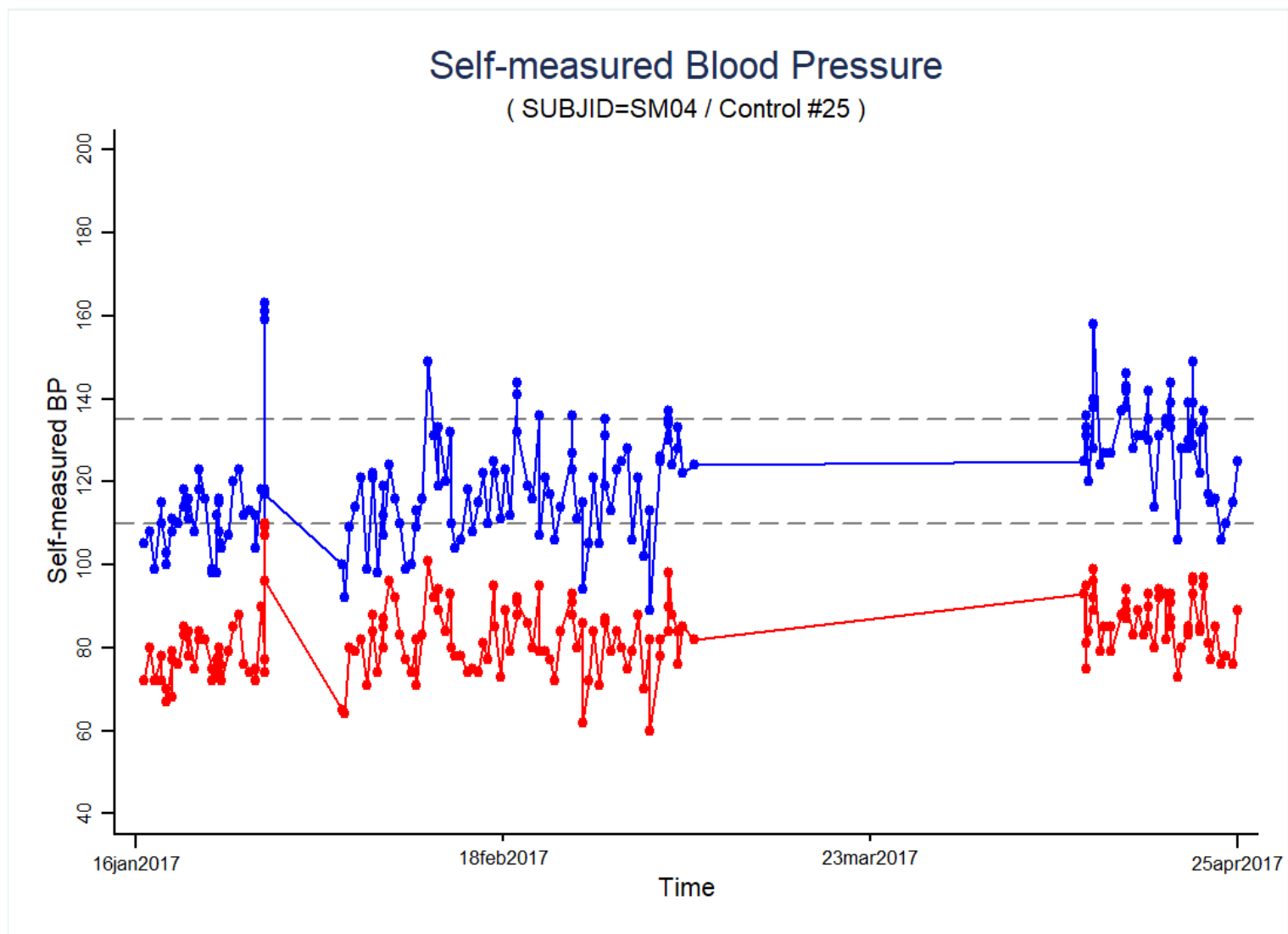

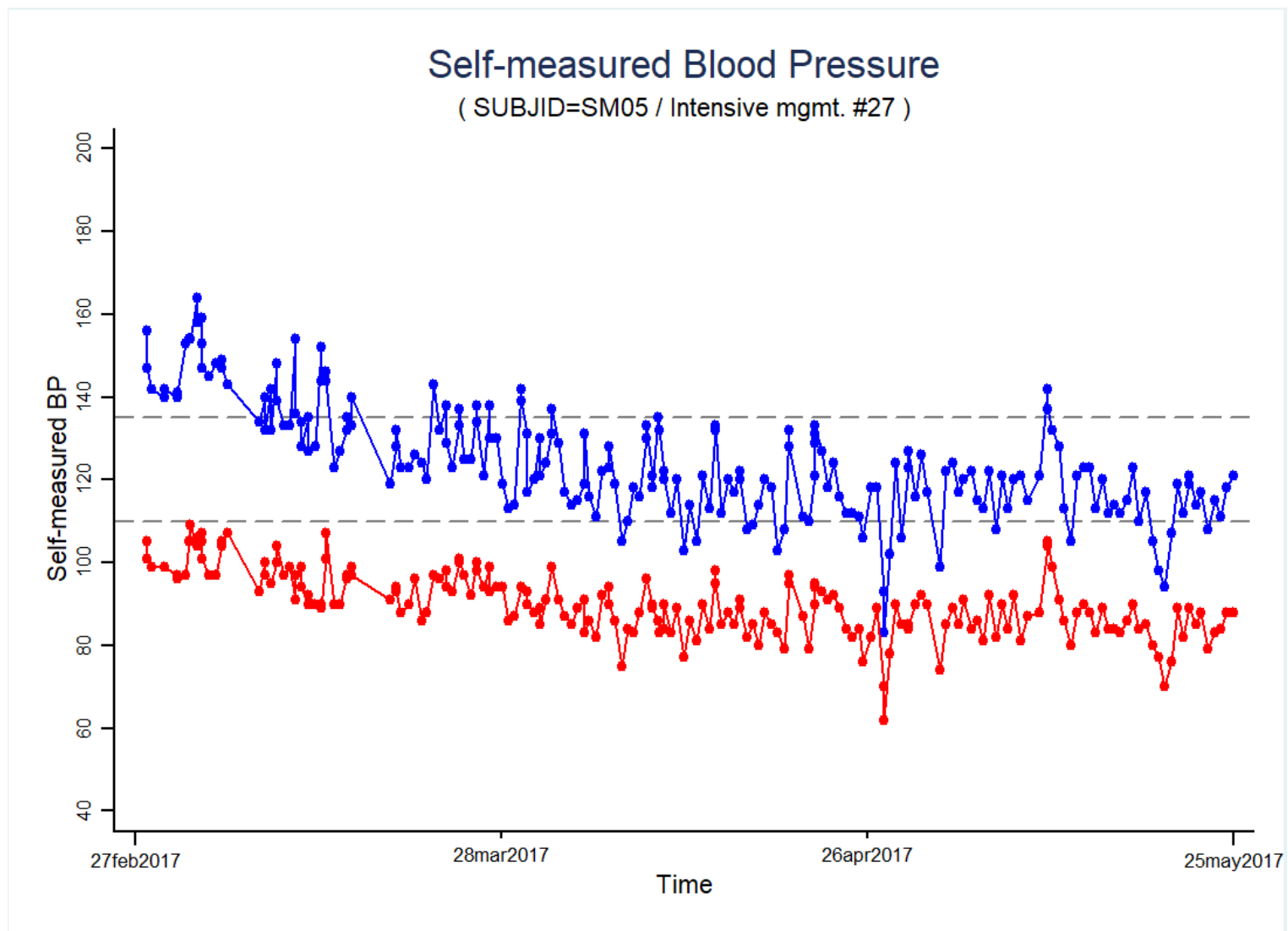

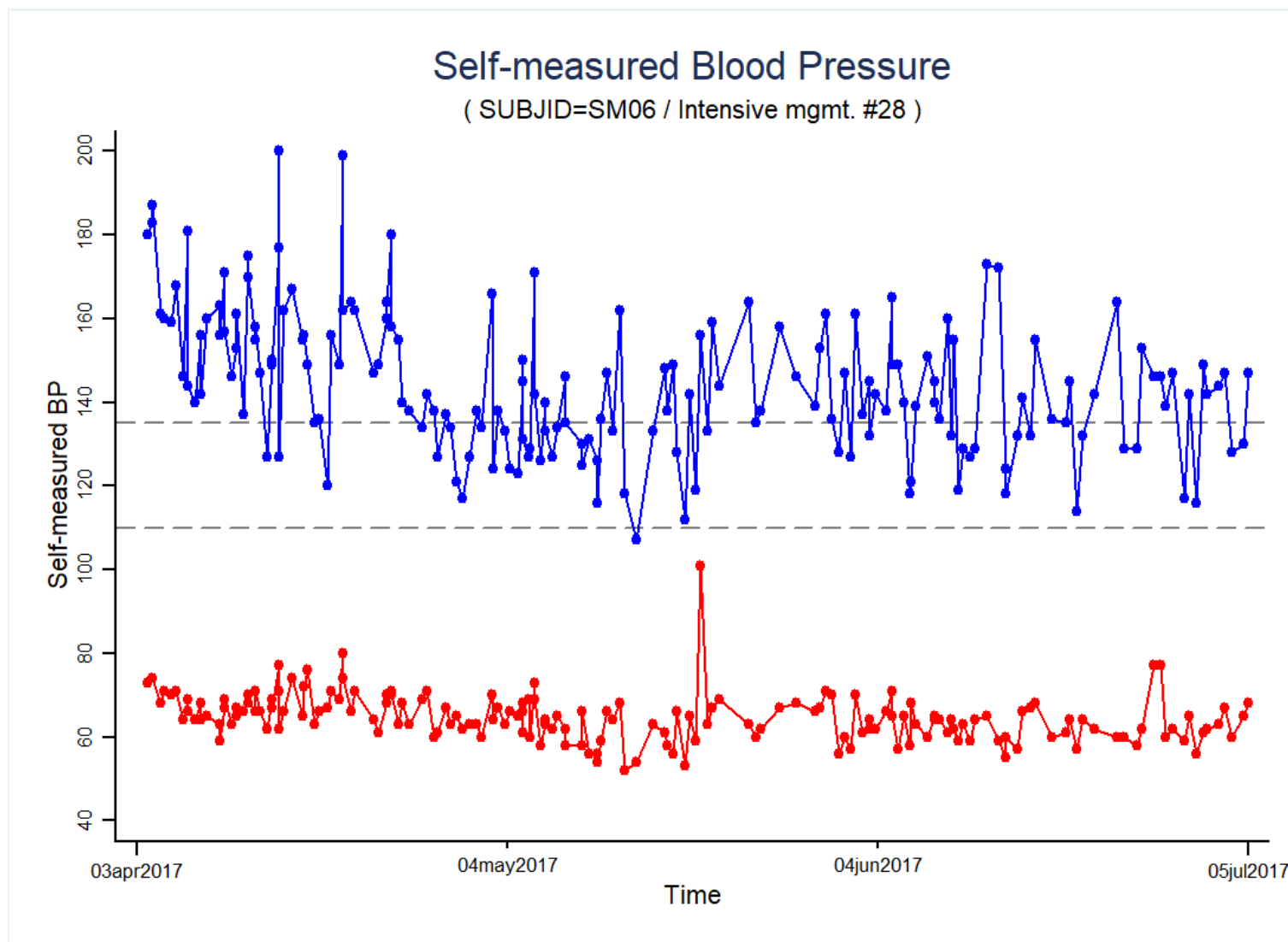

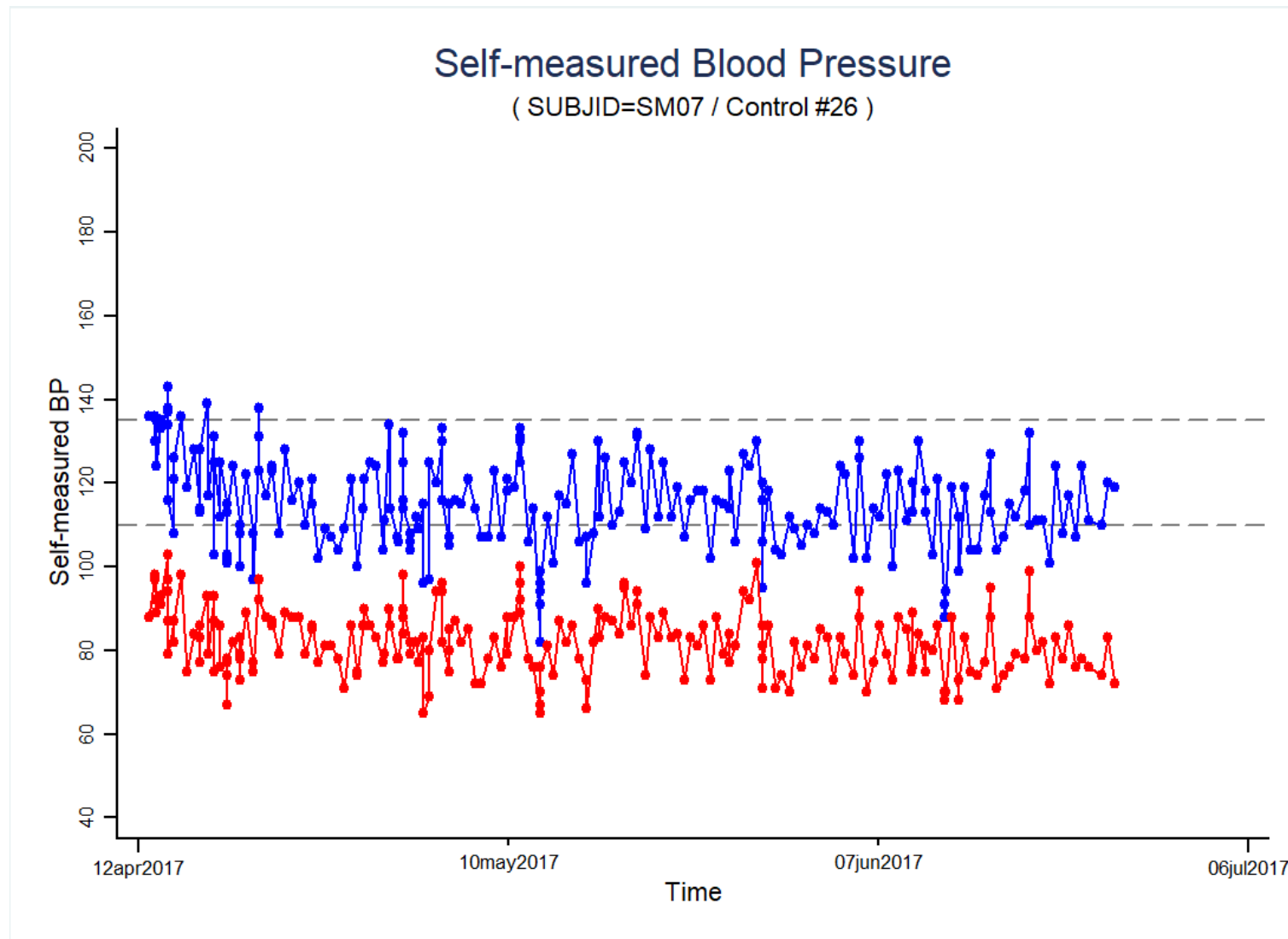

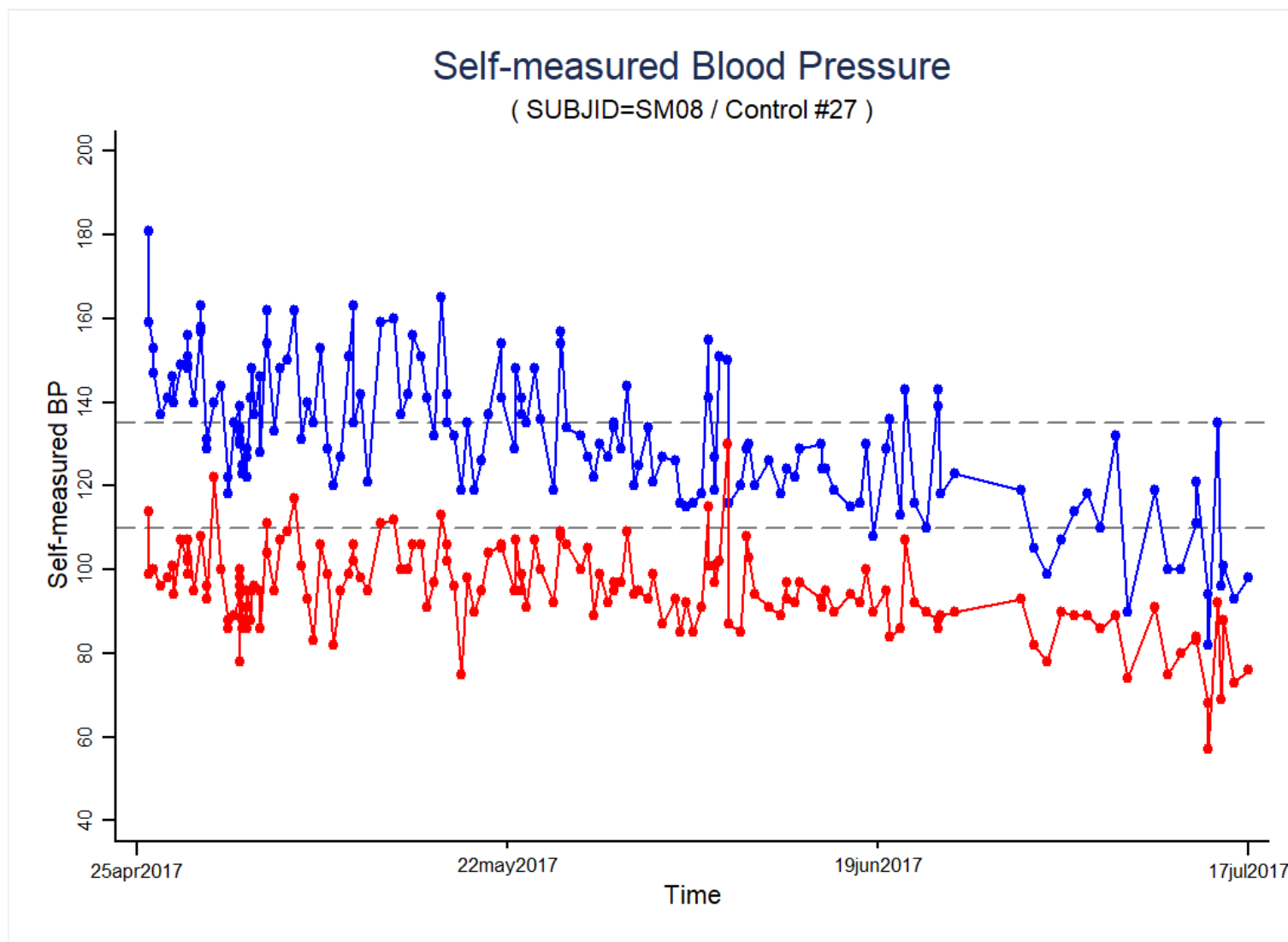

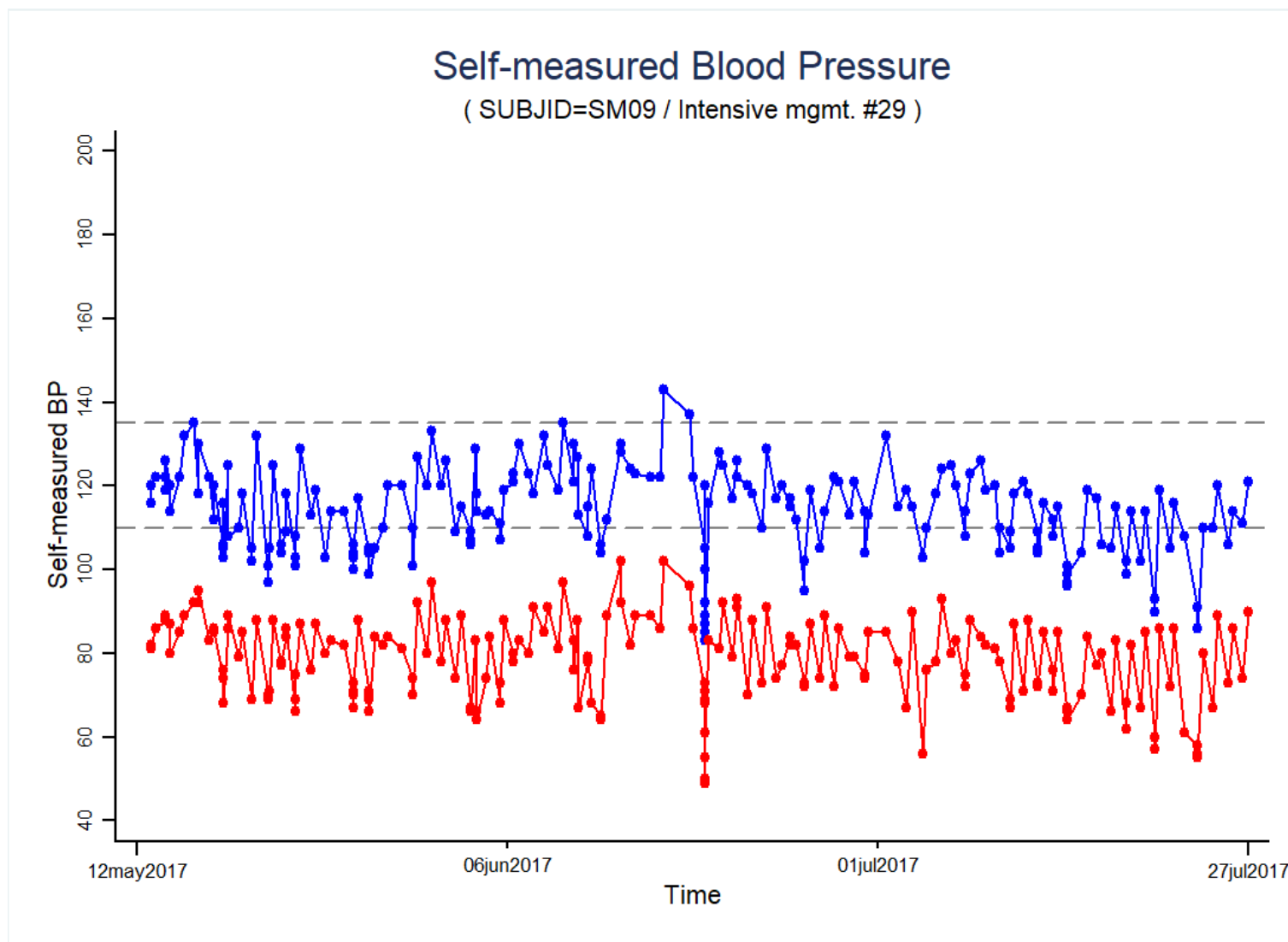

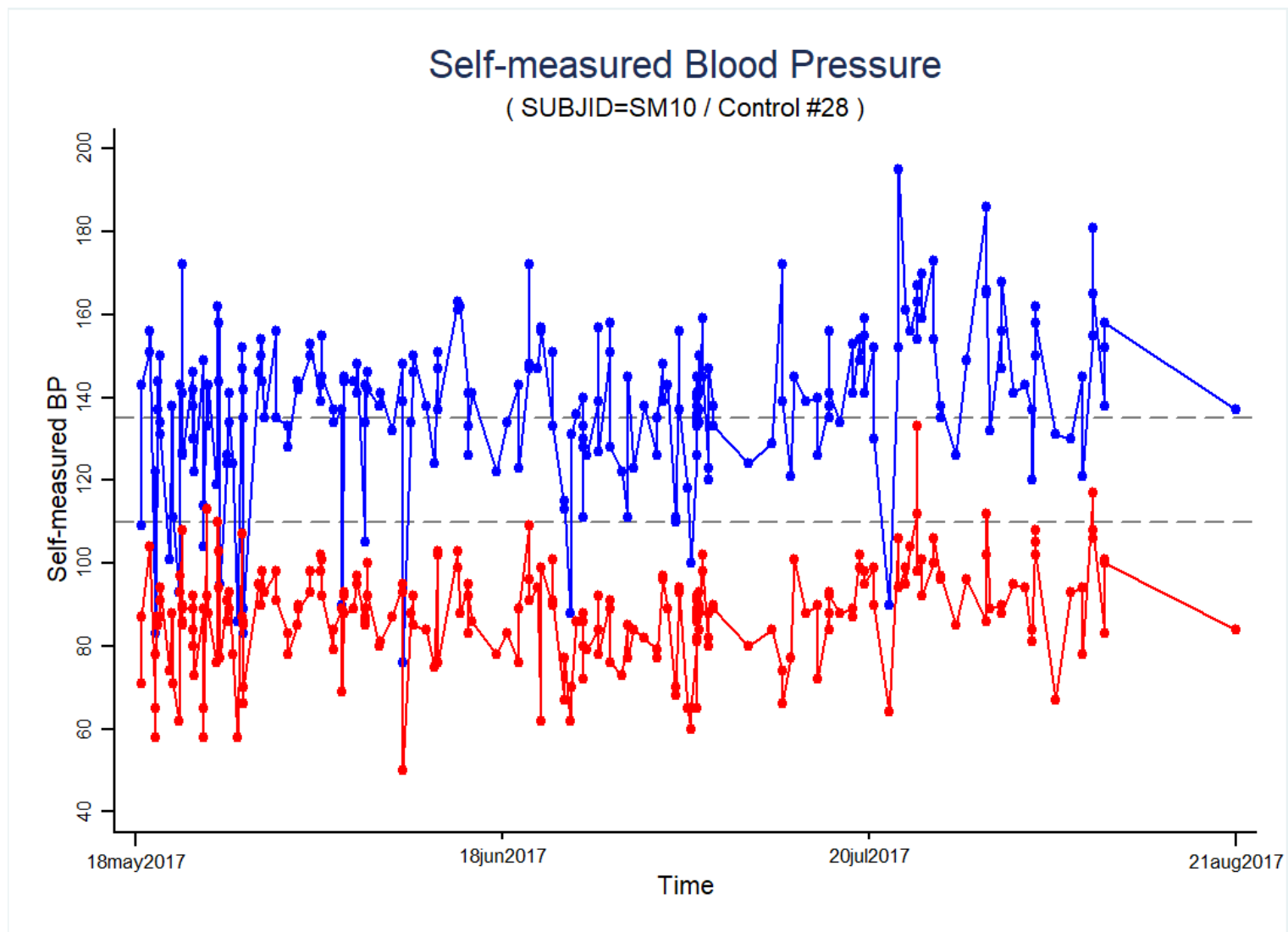

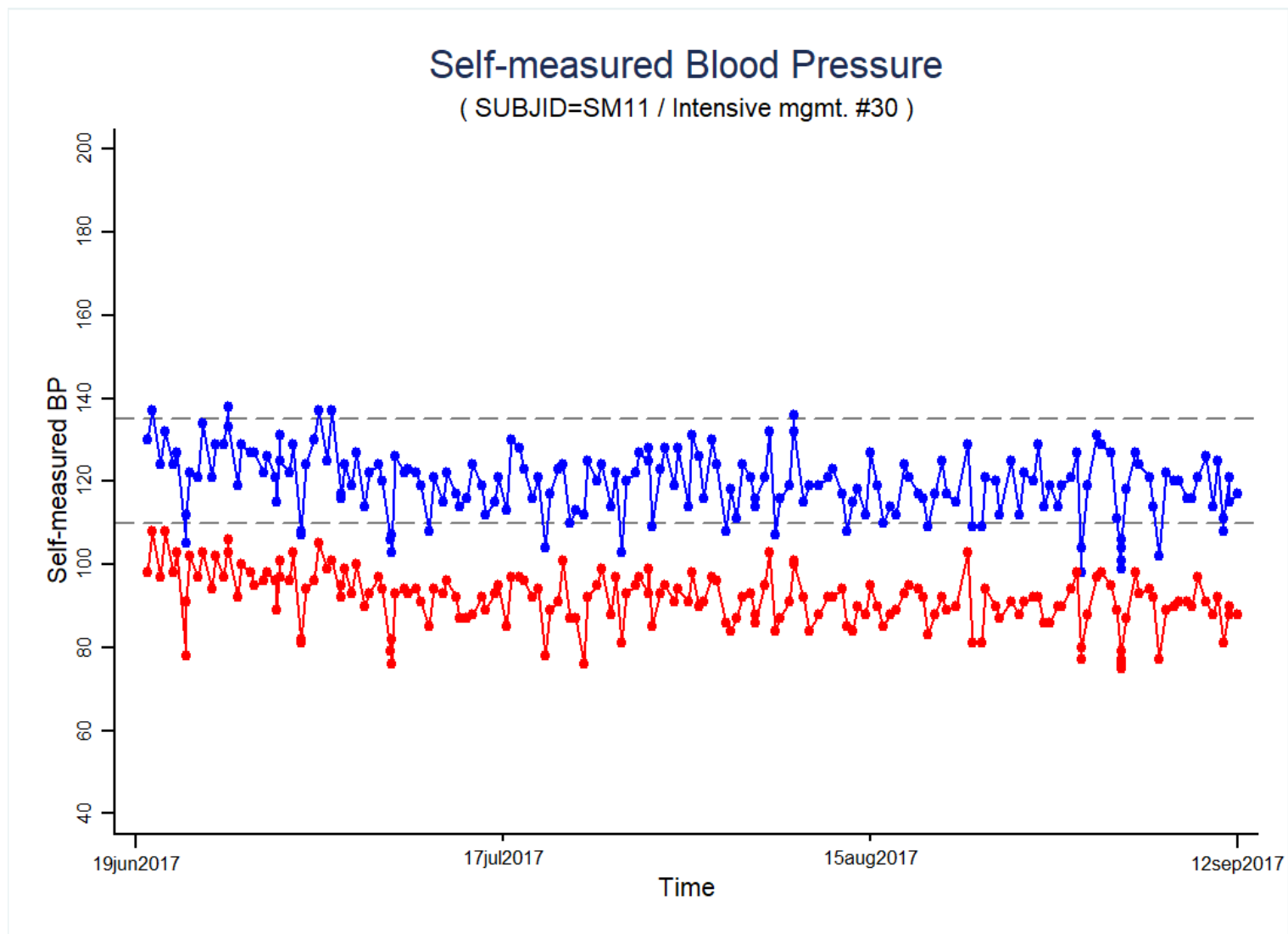

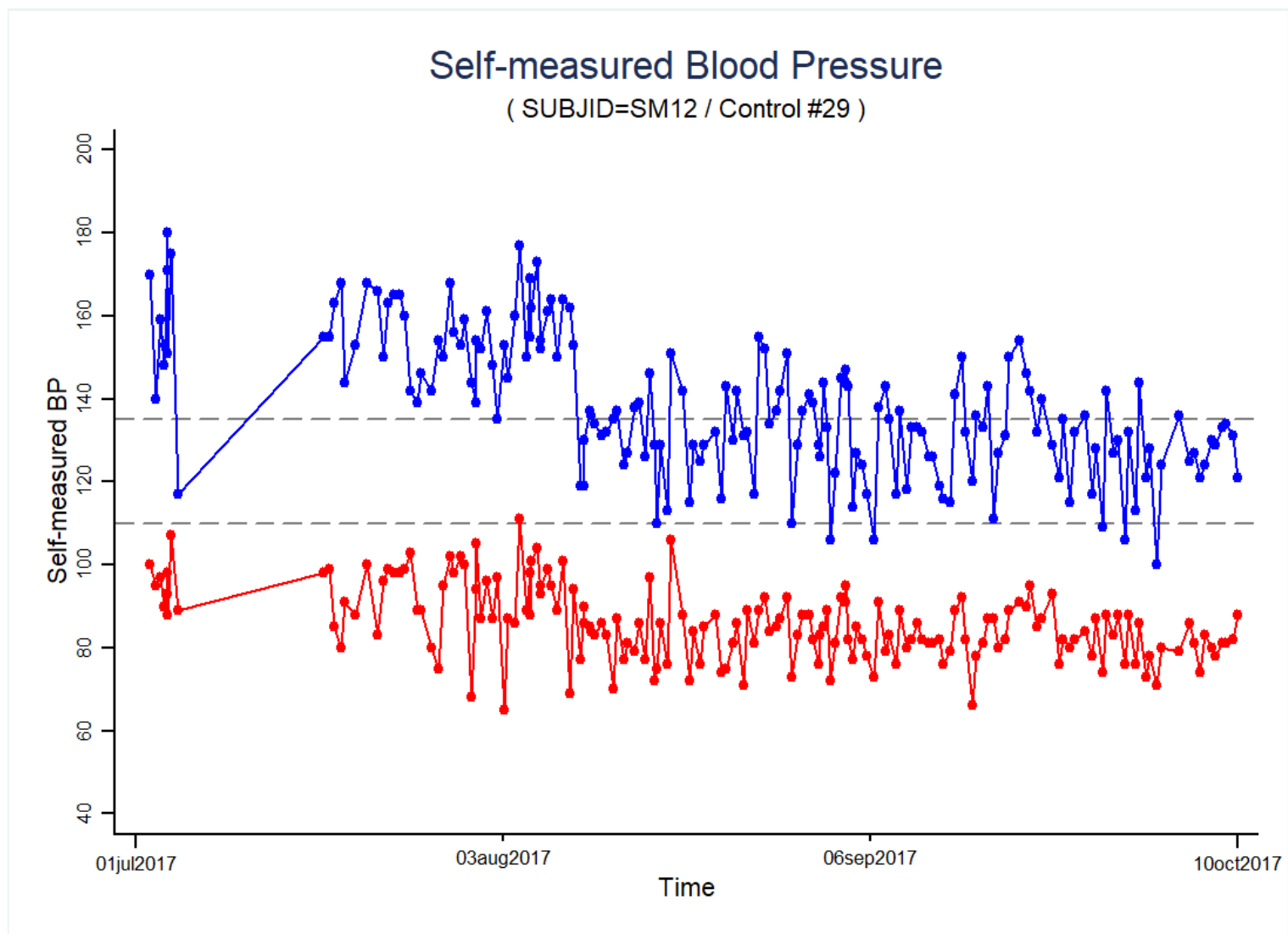

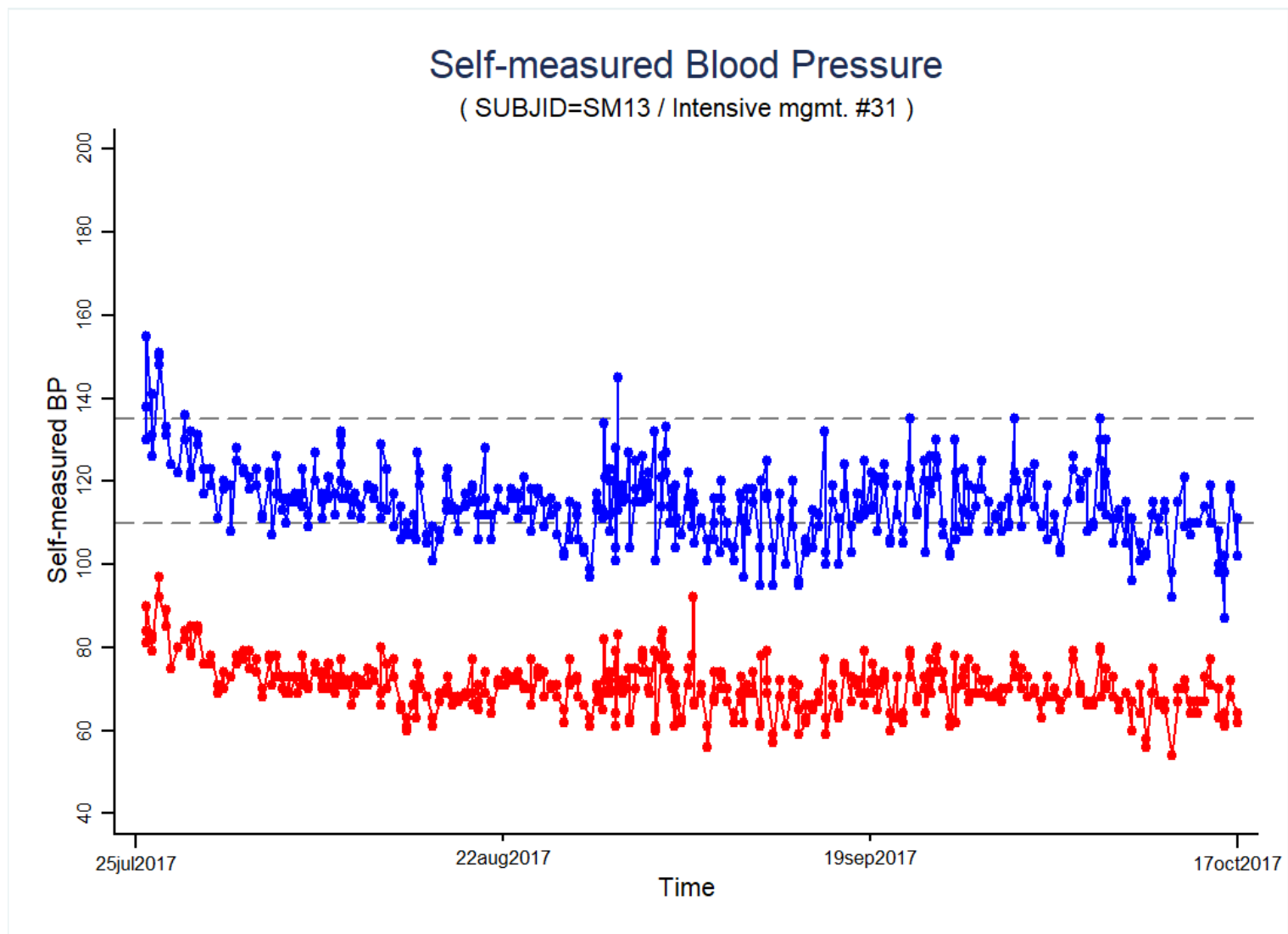

Supplement: S2 Fig — (PDF) [file pone.0229483.s009.pdf]
