# Supplementary material for: Remote blood pressure monitoring and behavioral intensification for stroke: A randomized controlled feasibility trial
Source: PLoS One. 2020 Mar 11;15(3):e0229483. doi: 10.1371/journal.pone.0229483 (PMC7065804; doi:10.1371/journal.pone.0229483)
Supplement: S1 Table — (PDF) [file pone.0229483.s011.pdf]

S1 Table. Proportion of patients who satisfies the criteria of BP measurement

	Intensive mgmt. group (n=31)	Control group (n=29)	Difference (95% CI)
Total duration of BP data collection (day) *	2609	2572	
Mean duration of BP data collection per subject (day), mean (SD)	84.16 (20.04)	88.69 (13.88)	
Total number of intervals	5218	5144	-4.53 (-13.49, 4.44)
Number of Total intervals per Patients			
Mean (SD)	167.03 (40.25)	167.03 (36.18)	0 (-19.83, 19.82)
Median (Q1, Q3)	178.00 (166.00, 186.00)	170.00 (164.00, 180.00)	
(Min, Max)	(16.00, 202.00)	(60.00, 246.00)	
Underwent transmission failure			
Number of subjects, n (%)	5 (16.13)	8 (27.59)	
Total duration of transmission failure occurrence (day)	20	150	
Mean duration of transmission failure occurrence per subject (day), mean (SD)	0.65 (2.21)	5.17 (13.50)	
Total Percentage of transmission failure occurrence (%)†	0.77	5.83	-4.53 (-9.72, 0.66)
Mean Percentage of transmission failure occurrence per subject (%), mean (SD)	0.01 (0.03)	0.06 (0.15)	
			-0.05 (-0.1, 0.01)
Proportion of blocks per patient that			

satisfied the criteria of BP measurement‡			
Mean (SD)	0.86 (0.13)	0.74 (0.24)	0.12 (0.02, 0.22)
Median (Q1, Q3)	0.91 (0.76, 0.97)	0.83 (0.64, 0.90)	
(Min, Max)	(0.51, 1.00)	(0.07, 0.99)	
Number of patients that satisfy the criteria of BP measurement by half-day intervals, n (%) §			
No	0 (0.00)	3 (10.34)	10.34% (-1.36, 22.05)
Yes	31 (100.0)	26 (89.66)	

* Total duration of BP data collection; Days of subjects' retention (including duration of mechanical failure)

† Total percentage of mechanical failure occurrence (%) = duration of mechanical failure / total duration of BP data collection * 100

‡ The proportion of blocks per patient that satisfied the criteria of BP measurement = number of half-day intervals during which more than one BP measurements were made / number of total intervals during BP data collection period without mechanical failure

§ Proportion of patients who satisfies the criteria of blood pressure measurement by half-day interval definitions (refer to Supplemental data #3)
