# Supplementary material for: Remote blood pressure monitoring and behavioral intensification for stroke: A randomized controlled feasibility trial
Source: PLoS One. 2020 Mar 11;15(3):e0229483. doi: 10.1371/journal.pone.0229483 (PMC7065804; doi:10.1371/journal.pone.0229483)
Supplement: S2 Table — (PDF) [file pone.0229483.s012.pdf]

S2 Table. Primary and secondary outcomes of the trial from per-protocol population

	Intensive management group (n, 29)	Control group (n, 27)	Difference (95% CI)
Primary endpoint			
Recruitment time to respecified number of subjects	328	340	N/A
Retention of included participants	29 (100%)	27 (100%)	0.00% (-)
Total number of frequency of calls for breakthrough visit	8*	N/A	
Breakthrough visit response	8 (100%)	N/A	
Days between calls and visit	2 [0, 3]		
Compliance to BP measurements	29 (100%)	25 (93%)	7.41% (-2.86, 17.67)
Duration of mechanical errors per subject (day)	0.7 ± 2.2	5.6 ± 13.9	-4.87 (-10.43, 0.7)
Rate of number of measured half-day blocks per patient (%)	86 ± 13	76 ± 21	9.79 (0.39, 19.2)
Secondary efficacy endpoints			
Average proportion of OOR measurements (%)	41 ± 15	41 ± 16	-0.41 (-8.51, 7.7)
Weighted average proportion of OOR measurements	65 ± 28	63 ± 28	1.81 (-13.35, 16.97)
Vascular events			
Recurrent stroke	2 (6%)	0 (0%)	6.90% (-)
Myocardial infarction	0	0	
All kinds of death	0	0	
