# Supplementary material for: Remote blood pressure monitoring and behavioral intensification for stroke: A randomized controlled feasibility trial
Source: PLoS One. 2020 Mar 11;15(3):e0229483. doi: 10.1371/journal.pone.0229483 (PMC7065804; doi:10.1371/journal.pone.0229483)
Supplement: S3 Table — (PDF) [file pone.0229483.s013.pdf]

S3 Table. Average BP measurements during the trial

Mean Systolic Blood Pressure over Weeks (ITT Population)

	Total (n=60)*			Intensive mgmt. group (n=31)*				Control group (n=29)*						
Week	N	M	Mean (SD)	N	M	Mean (SD)	p-value	N	M	Mean (SD)	p-value	p-value †	p-value	
1	60	1425	134.14 (15.14)	31	787	134.14 (14.22)	Ref	29	638	134.14 (16.32)	Ref	0.9778	0.6343 (c)	Ref
2	59	1328	128.52 (15.16)	31	745	127.96 (15.30)	0.0003 (a)	28	583	129.13 (15.26)	0.0016 (b)	0.5466	<.0001 (d)	<.0001 (f)
3	57	1254	127.41 (14.68)	30	750	128.19 (14.72)	0.0016 (a)	27	504	126.53 (14.88)	0.0005 (b)	0.9881	<.0001 (e)	<.0001 (f)
4	55	1205	125.69 (13.29)	28	733	125.84 (12.68)	0.0008 (a)	27	472	125.54 (14.15)	0.0003 (b)	0.9094		<.0001 (f)
5	57	1262	125.25 (12.67)	29	751	125.59 (12.76)	0.0004 (a)	28	511	124.91 (12.80)	0.0003 (b)	0.9217		<.0001 (f)
6	56	1174	126.02 (10.63)	29	673	125.49 (11.32)	0.0005 (a)	27	501	126.59 (10.03)	0.0029 (b)	0.8133		<.0001 (f)
7	56	1241	126.37 (10.36)	29	690	127.61 (10.57)	0.0070 (a)	27	551	125.05 (10.17)	0.0005 (b)	0.4332		<.0001 (f)
8	54	1034	126.33 (10.77)	29	639	126.54 (11.79)	0.0013 (a)	25	395	126.09 (9.68)	0.0070 (b)	0.9657		<.0001 (f)
9	54	1125	125.68 (10.54)	29	693	124.79 (10.98)	<.0001 (a)	25	432	126.72 (10.12)	0.0172 (b)	0.4864		<.0001 (f)
10	54	1152	124.53 (9.80)	29	731	124.08 (9.91)	<.0001 (a)	25	421	125.04 (9.86)	0.0046 (b)	0.6883		<.0001 (f)
11	54	1090	125.06 (10.49)	29	689	124.55 (9.21)	<.0001 (a)	25	401	125.64 (11.97)	0.0094 (b)	0.6450		<.0001 (f)
12	52	1000	125.43 (10.32)	28	634	124.38 (9.55)	<.0001 (a)	24	366	126.64 (11.23)	0.0085 (b)	0.4101		<.0001 (f)
13	38	602	126.77 (10.47)	21	400	125.90 (10.13)	<.0001 (a)	17	202	127.85 (11.08)	0.0067 (b)	0.5827		<.0001 (f)
14	20	265	126.73 (8.13)	11	160	127.34 (7.95)	0.0002 (a)	9	105	125.98 (8.78)	0.0035 (b)	0.7794		<.0001 (f)
15	7	31	134.22 (12.76)	3	11	135.29 (14.49)	0.1020 (a)	4	20	133.41 (13.56)	0.4831 (b)	0.5363		0.1124 (f)

* Average systolic blood pressure measurements by week (N, number of subjects; M, number of measurements)

All p-values are calculated by mixed model for repeated measures (MMRM) analysis (Estimated variance of Site ID variable as a random factor, $\widehat{\sigma^2_{\text{Site ID}}}$, is 0.0)

† p-value for mean difference of SBP between groups within each of weeks by using contrast test within MMRM

(a) p-value for mean difference of SBP from week 1 to each of weeks for intensive management group (i.e., reference = SBP of week 1)

(b) p-value for mean difference of SBP from week 1 to each of weeks for control group (i.e., reference = SBP of week 1)

(c) p-value for group*week interaction effect

(d) p-value for mean equality of SBP across weeks with total patients

(e) p-value for linear trend of SBP across weeks with total patients

(f) p-value for mean difference of SBP from week 1 to each of weeks with total patients (i.e., reference = SBP of week 1)

Mean Diastolic Blood Pressure over Weeks (ITT Population)

	Total (n=60)*			Intensive mgmt. group (n=31)*				Control group (n=29)*						
Week	N	M	Mean (SD)	N	M	Mean (SD)	p-value	N	M	Mean (SD)	p-value	p-value †	p-value	
1	60	1424	86.65 (10.25)	31	786	85.20 (11.30)	Ref	29	638	88.19 (8.95)	Ref	0.2124	0.7138 (c)	Ref
2	59	1328	83.51 (9.81)	31	745	81.51 (10.67)	<.0001 (a)	28	583	85.73 (8.39)	0.0149 (b)	0.0641	<.0001 (d)	<.0001 (f)
3	57	1252	82.16 (9.53)	30	750	80.98 (10.55)	0.0003 (a)	27	502	83.47 (8.25)	0.0006 (b)	0.1764	<.0001 (e)	<.0001 (f)
4	55	1203	81.46 (9.20)	28	733	79.52 (9.89)	<.0001 (a)	27	470	83.47 (8.13)	0.0007 (b)	0.1083		<.0001 (f)
5	57	1262	81.06 (8.71)	29	751	79.28 (9.82)	<.0001 (a)	28	511	82.90 (7.10)	0.0005 (b)	0.1405		<.0001 (f)
6	56	1173	81.69 (8.95)	29	673	80.16 (9.76)	0.0009 (a)	27	500	83.34 (7.84)	0.0009 (b)	0.2091		<.0001 (f)
7	56	1241	81.50 (8.24)	29	690	80.46 (9.16)	0.0023 (a)	27	551	82.62 (7.12)	0.0002 (b)	0.3976		<.0001 (f)
8	54	1032	81.00 (8.42)	29	637	79.46 (9.13)	0.0002 (a)	25	395	82.79 (7.31)	0.0027 (b)	0.1146		<.0001 (f)
9	54	1125	80.87 (9.18)	29	693	78.59 (8.51)	<.0001 (a)	25	432	83.51 (9.38)	0.0083 (b)	0.0341		<.0001 (f)
10	54	1152	80.58 (8.33)	29	731	78.49 (7.73)	<.0001 (a)	25	421	83.01 (8.48)	0.0036 (b)	0.0485		<.0001 (f)
11	54	1090	80.63 (8.88)	29	689	78.70 (8.55)	<.0001 (a)	25	401	82.86 (8.90)	0.0032 (b)	0.0620		<.0001 (f)
12	52	999	80.67 (8.91)	28	634	78.65 (8.36)	<.0001 (a)	24	365	83.02 (9.12)	0.0023 (b)	0.0494		<.0001 (f)
13	38	602	82.04 (9.49)	21	400	80.34 (10.65)	<.0001 (a)	17	202	84.15 (7.62)	0.0019 (b)	0.1432		<.0001 (f)
14	20	263	80.69 (7.91)	11	160	78.27 (8.20)	0.0002 (a)	9	103	83.66 (6.83)	<.0001 (b)	0.7325		<.0001 (f)
15	7	31	84.69 (9.51)	3	11	79.67 (9.24)	0.0642 (a)	4	20	88.46 (8.92)	0.0901 (b)	0.3436		0.0065 (f)

* Average diastolic blood pressure measurements by week (N, number of subjects; M, number of measurements)

All p-values are calculated by mixed model for repeated measures (MMRM) analysis (Estimated variance of Site ID variable as a random factor, $\widehat{\sigma^2_{\mathrm{Site\ ID}}}$, is 0.0)

† p-value for mean difference of DBP between groups within each of weeks by using contrast test within MMRM

(a) p-value for mean difference of DBP from week 1 to each of weeks for intensive management group (i.e., reference = DBP of week 1)

(b) p-value for mean difference of DBP from week 1 to each of weeks for control group (i.e., reference = DBP of week 1)

(c) p-value for group*week interaction effect

(d) p-value for mean equality of DBP across weeks with total patients

(e) p-value for linear trend of DBP across weeks with total patients

(f) p-value for mean difference of DBP from week 1 to each of weeks with total patients (i.e., reference = DBP of week 1)

Mean Systolic Blood Pressure over Weeks (PP Population)

	Total (n=56)*			Intensive mgmt. group (n=29)*				Control group (n=27)*						
Week	N	M	Mean (SD)	N	M	Mean (SD)	p-value‡	N	M	Mean (SD)	p-value‡	p-value †	p-value ‡	
1	56	1351	133.29(14.44)	29	737	134.51(14.46)	Ref	27	614	131.98(14.57)	Ref	0.4091	0.5170 (c)	Ref
2	56	1284	127.81(14.11)	29	702	127.89(14.81)	0.0001 (a)	27	582	127.73(13.59)	0.0025 (b)	0.9196	<.0001 (d)	<.0001 (f)
3	55	1224	126.25(13.58)	29	726	127.02(13.48)	0.0003 (a)	26	498	125.39(13.90)	0.0009 (b)	0.5985	<.0001 (e)	<.0001 (f)
4	54	1193	125.15(12.79)	28	733	125.84(12.68)	0.0002 (a)	26	460	124.41(13.12)	0.0004 (b)	0.5382		<.0001 (f)
5	56	1247	124.78(12.26)	29	751	125.59(12.76)	<.0001 (a)	27	496	123.91(11.89)	0.0006 (b)	0.573		<.0001 (f)
6	55	1155	125.55(10.12)	29	673	125.49(11.32)	0.0002 (a)	26	482	125.61(8.82)	0.0051 (b)	0.8449		<.0001 (f)
7	55	1226	126.31(10.45)	29	690	127.61(10.57)	0.0028 (a)	26	536	124.86(10.33)	0.0022 (b)	0.2918		<.0001 (f)
8	53	1022	126.22(10.84)	29	639	126.54(11.79)	0.0004 (a)	24	383	125.84(9.81)	0.0282 (b)	0.8544		<.0001 (f)
9	53	1110	125.50(10.55)	29	693	124.79(10.98)	<.0001 (a)	24	417	126.36(10.17)	0.0553 (b)	0.6508		<.0001 (f)
10	53	1135	124.43(9.87)	29	731	124.08(9.91)	<.0001 (a)	24	404	124.85(10.03)	0.0188 (b)	0.8523		<.0001 (f)
11	53	1076	124.99(10.58)	29	689	124.55(9.21)	<.0001 (a)	24	387	125.53(12.21)	0.0351 (b)	0.8039		<.0001 (f)
12	51	988	125.20(10.29)	28	634	124.38(9.55)	<.0001 (a)	23	354	126.19(11.26)	0.0236 (b)	0.6161		<.0001 (f)
13	37	589	126.82(10.61)	21	400	125.90(10.13)	<.0001 (a)	16	189	128.02(11.42)	0.0340 (b)	0.693		<.0001 (f)
14	19	254	126.84(8.34)	11	160	127.34(7.95)	0.0001 (a)	8	94	126.15(9.37)	0.0337 (b)	0.7333		<.0001 (f)
15	6	24	135.11(13.74)	3	11	135.29(14.49)	0.1000 (a)	3	13	134.93(16.19)	0.3193 (b)	0.2056		0.0001 (f)

* Average systolic blood pressure measurements by week (N, number of subjects; M, number of measurements)

All p-values are calculated by mixed model for repeated measures (MMRM) analysis (Estimated variance of Site ID variable as a random factor, $\widehat{\sigma^2_{\text{Site ID}}}$, is 0.0)

† p-value for mean difference of SBP between groups within each of weeks by using contrast test within MMRM

(a) p-value for mean difference of SBP from week 1 to each of weeks for intensive management group (i.e., reference = SBP of week 1)

(b) p-value for mean difference of SBP from week 1 to each of weeks for control group (i.e., reference = SBP of week 1)

(c) p-value for group*week interaction effect

(d) p-value for mean equality of SBP across weeks with total patients

(e) p-value for linear trend of SBP across weeks with total patients

(f) p-value for mean difference of SBP from week 1 to each of weeks with total patients (i.e., reference = SBP of week 1)

Mean Diastolic Blood Pressure over Weeks (PP Population)

Week	Total (n=56)*			Intensive mgmt. group (n=29)*				Control group (n=27)*						
	N	M	Mean (SD)	N	M	Mean (SD)	p-value‡	N	M	Mean (SD)	p-value‡	p-value †	p-value ‡	
1	56	1350	85.89(10.06)	29	736	84.66(11.48)		27	614	87.22(8.28)	Ref	0.2829	0.7868 (c)	Ref
2	56	1284	82.75(9.21)	29	702	80.80(10.60)	<.0001 (a)	27	582	84.84(7.05)	0.0152 (b)	0.0925	0.0001 (d)	<.0001 (f)
3	55	1222	81.66(9.25)	29	726	80.27(9.97)	<.0001 (a)	26	496	83.21(8.30)	0.0013 (b)	0.187	0.0001 (e)	<.0001 (f)
4	54	1191	81.25(9.17)	28	733	79.52(9.89)	<.0001 (a)	26	458	83.12(8.09)	0.0007 (b)	0.1321		<.0001 (f)
5	56	1247	80.87(8.68)	29	751	79.28(9.82)	<.0001 (a)	27	496	82.59(7.04)	0.0008 (b)	0.1663		<.0001 (f)
6	55	1154	81.50(8.92)	29	673	80.16(9.76)	0.0004 (a)	26	481	83.00(7.80)	0.0014 (b)	0.2477		<.0001 (f)
7	55	1226	81.51(8.31)	29	690	80.46(9.16)	0.0013 (a)	26	536	82.68(7.26)	0.0009 (b)	0.3732		<.0001 (f)
8	53	1020	80.98(8.50)	29	637	79.46(9.13)	<.0001 (a)	24	383	82.82(7.46)	0.0086 (b)	0.1053		<.0001 (f)
9	53	1110	80.79(9.25)	29	693	78.59(8.51)	<.0001 (a)	24	417	83.45(9.57)	0.0195 (b)	0.0332		<.0001 (f)
10	53	1135	80.53(8.40)	29	731	78.49(7.73)	<.0001 (a)	24	404	83.01(8.66)	0.0097 (b)	0.0453		<.0001 (f)
11	53	1076	80.60(8.96)	29	689	78.70(8.55)	<.0001 (a)	24	387	82.90(9.09)	0.0088 (b)	0.0573		<.0001 (f)
12	51	987	80.57(8.96)	28	634	78.65(8.36)	<.0001 (a)	23	353	82.89(9.31)	0.0048 (b)	0.0538		<.0001 (f)
13	37	589	82.04(9.62)	21	400	80.34(10.65)	<.0001 (a)	16	189	84.26(7.86)	0.0075 (b)	0.1293		<.0001 (f)
14	19	252	80.77(8.12)	11	160	78.27(8.20)	0.0002 (a)	8	92	84.21(7.09)	0.0002 (b)	0.6266		<.0001 (f)
15	6	24	85.17(10.33)	3	11	79.67(9.24)	0.0639 (a)	3	13	90.67(9.50)	0.9332 (b)	0.1321		0.1582 (f)

* Average diastolic blood pressure measurements by week (N, number of subjects; M, number of measurements)

All p-values are calculated by mixed model for repeated measures (MMRM) analysis (Estimated variance of Site ID variable as a random factor, $\widehat{\sigma^2_{\text{Site ID}}}$, is 0.0)

† p-value for mean difference of DBP between groups within each of weeks by using contrast test within MMRM

(a) p-value for mean difference of DBP from week 1 to each of weeks for intensive management group (i.e., reference = DBP of week 1)

(b) p-value for mean difference of DBP from week 1 to each of weeks for control group (i.e., reference = DBP of week 1)

(c) p-value for group*week interaction effect

(d) p-value for mean equality of DBP across weeks with total patients

(e) p-value for linear trend of DBP across weeks with total patients

(f) p-value for mean difference of DBP from week 1 to each of weeks with total patients (i.e., reference = DBP of week 1)
