# Supplementary material for: Remote blood pressure monitoring and behavioral intensification for stroke: A randomized controlled feasibility trial
Source: PLoS One. 2020 Mar 11;15(3):e0229483. doi: 10.1371/journal.pone.0229483 (PMC7065804; doi:10.1371/journal.pone.0229483)
Supplement: S4 Table — (PDF) [file pone.0229483.s014.pdf]

S4 Table. Adverse events

Incidence of Adverse Event by Severity, Preferred Term and Treatment group (Safety population)

	Event Rate						Number of Event					
	Intensive mgmt. group (n=31)			Control group (n=29)			Intensive mgmt. group			Control group		
Preferred Term, n (%)	Mild	Moderate	Severe	Mild	Moderate	Severe	Mild	Moderate	Severe	Mild	Moderate	Severe
Subjects with any AE	4 (12.9)	0 (0.0)	0 (0.0)	3 (10.3)	3 (10.3)	0 (0.0)	5	0	0	3	3	0
Abdominal distension	1 (3.2)	0 (0.0)	0 (0.0)	0 (0.0)	0 (0.0)	0 (0.0)	1	0	0	0	0	0
Abdominal pain upper	0 (0.0)	0 (0.0)	0 (0.0)	1 (3.4)	0 (0.0)	0 (0.0)	0	0	0	1	0	0
Angina pectoris	1 (3.2)	0 (0.0)	0 (0.0)	0 (0.0)	0 (0.0)	0 (0.0)	1	0	0	0	0	0
Cerebral infarction	0 (0.0)	0 (0.0)	0 (0.0)	0 (0.0)	1 (3.4)	0 (0.0)	0	0	0	0	1	0
Headache	0 (0.0)	0 (0.0)	0 (0.0)	1 (3.4)	0 (0.0)	0 (0.0)	0	0	0	1	0	0
Hemiparaesthesia	1 (3.2)	0 (0.0)	0 (0.0)	0 (0.0)	0 (0.0)	0 (0.0)	1	0	0	0	0	0
Oedema	0 (0.0)	0 (0.0)	0 (0.0)	1 (3.4)	0 (0.0)	0 (0.0)	0	0	0	1	0	0
Retinal hemorrhage	0 (0.0)	0 (0.0)	0 (0.0)	0 (0.0)	1 (3.4)	0 (0.0)	0	0	0	0	1	0
Road traffic accident	1 (3.2)	0 (0.0)	0 (0.0)	0 (0.0)	0 (0.0)	0 (0.0)	1	0	0	0	0	0
Seizure	0 (0.0)	0 (0.0)	0 (0.0)	0 (0.0)	1 (3.4)	0 (0.0)	0	0	0	0	1	0
Transient ischemic attack	1 (3.2)	0 (0.0)	0 (0.0)	0 (0.0)	0 (0.0)	0 (0.0)	1	0	0	0	0	0

Comparison of Safety Events Prevalence between groups (Safety population)

n (%)	Intensive mgmt. group (n=31)	Control group (n=29)	p-value
Dizziness, Fall, or Orthostatic Hypotension related events			-
Yes	0 (0.00)	0 (0.00)	
No	0 (0.00)	0 (0.00)	
Other Adverse event potentially related			1.0000
Yes	3 (9.68)	3 (10.34)	
Cerebral infarction	0 (0.00)	1 (3.45)	
Headache	0 (0.00)	1 (3.45)	
Oedema	0 (0.00)	1 (3.45)	
Hemiparaesthesia	1 (3.23)	0 (0.00)	
Angina pectoris	1 (3.23)	0 (0.00)	
Road traffic accident	1 (3.23)	0 (0.00)	
Transient ischemic attack	1 (3.23)	0 (0.00)	
No	28 (90.32)	26 (89.66)	
Mortality			-
Yes	0 (0.00)	0 (0.00)	
No	0 (0.00)	0 (0.00)	

p-value by Fisher’s exact test
