# Supplementary material for: Remote blood pressure monitoring and behavioral intensification for stroke: A randomized controlled feasibility trial
Source: PLoS One. 2020 Mar 11;15(3):e0229483. doi: 10.1371/journal.pone.0229483 (PMC7065804; doi:10.1371/journal.pone.0229483)
Supplement: S5 Table — (PDF) [file pone.0229483.s015.pdf]

S5 Table. Compliance to study drug

Compliance to Study drug (ITT Population)

	Total (n=60)	Intensive mgmt. group (n=31)	Control group (n=29)	Difference (95% CI)
Compliance to Study drug * at Visit 3				-1.6 (-11.67, 8.47)
N	57	29	28	
Mean (SD)	95.61 (18.81)	86.21 (35.09)	96.43 (18.90)	
Median (IQR)	100.00 (100.00, 100.00)	100.00 (100.00, 100.00)	100.00 (100.00, 100.00)	
(Min, Max)	(0.00, 100.00)	(0.00, 100.00)	(0.00, 100.00)	
Compliance to Study drug at Visit 3 ≥ 67%, n(%)	55 (91.67)	25 (86.21)	27 (96.43)	-10.22% (-25.44, 5.00)
Compliance to Study drug at Visit 4				-10.34 (-22.13, 1.44)
N	57	29	28	
Mean (SD)	94.74 (22.53)	89.66 (30.99)	100.00 (0.00)	
Median (IQR)	100.00 (100.00, 100.00)	100.00 (100.00, 100.00)	100.00 (100.00, 100.00)	
(Min, Max)	(0.00, 100.00)	(0.00, 100.00)	(100.00, 100.00)	
Compliance to Study drug at Visit 4 ≥ 67%, n(%)	54 (90.00)	26 (89.66)	28 (100.00)	-10.34% (-22.05, 1.36)

(a) p-value by Student's t-test
(b) p-value by Fisher's exact test
* Compliance to Study drug = [1-(Missed medication during the preceding 10 day before visit)/10]× 100
