# Supplementary material for: Remote blood pressure monitoring and behavioral intensification for stroke: A randomized controlled feasibility trial
Source: PLoS One. 2020 Mar 11;15(3):e0229483. doi: 10.1371/journal.pone.0229483 (PMC7065804; doi:10.1371/journal.pone.0229483)
Supplement: S1 Protocol — (PDF) [file pone.0229483.s016.pdf]

임상시험계획서

제목: 급성 허혈뇌졸중에서 혈압관리의 최적화 전략에 관한 제 2 상 전향적, 무작위 대조군, 공개, 실현가능성 평가 임상시험 [BOSS Trial I]

Protocol Version: Version 1.2

Protocol Date: 2016.07.29

책임 연구자

소 속 : 서울대학교 의과대학 분당서울대병원

전공 과목 : 신경과

직 위 : 교 수

성 명 : 배희준

공동연구자

분당서울대학교병원 신경과 한문구, 김범준

을지병원 신경과 박종무

서울의료원 신경과 박태환

고려대학교 의과대학 의학통계학교실 이준영

서울아산병원 임상의학연구소 이지성

목차

1. 연구 요약 (국문)
2. 연구 흐름도
3. 연구 계획서에서 사용된 약자
4. 서론
5. 연구 목적
6. 연구 설계
   1) 개요
   2) 연구 대상
   3) 무작위 배정 방법 및 절차
   4) 중재적 개입
      i. 임상시험 기간 중 혈압 관리의 기본적 원칙
      ii. 집중 관리군을 위한 혈압 강하제 조절 알고리듬 및 행동 강화를 위한 개입
      iii. 대조군의 혈압 관리 원칙
      iv. 약물 공급 및 수불관리
   5) 가정에서의 혈압 측정
   6) CRF 에 기반한 임상 연구 관리 시스템
   7) 결과 변수
   8) 공변수
7. 연구 절차
8. 병용 금지 약물 및 임상시험 계획 위반
9. 연구의 조기 종료
10. 이상 반응
11. 데이터 취급 및 품질 보증
12. 통계 방법, 표본 수 및 검정력
13. 연구 윤리
14. 참고 문헌
15. 동의서 및 환자 설명서
16. CRF 및 이상 반응 보고 서식

1. 연구 요약 (국문)

제목:	급성 허혈뇌졸중에서 혈압관리의 최적화 전략에 관한 제 2 상 전향적, 무작위 대조군, 공개, 실현가능성 평가 임상시험 [BOSS – Trial I]
후원:	한국 다이찌산쿄 ㈜
책임연구자	분당서울대학병원 신경과 배희준
참여연구대상수	총 60명 (본원 40명)
참여기관	3 곳 (분당서울대학병원, 노원을지병원, 서울의료원)
임상시험단계	제 2 상 (IIb) 임상시험으로<br>첫째, 블루투스 혈압기를 이용한 무선 혈압수집시스템의 실현가능성과,<br>둘째, 제시된 혈압관리알고리즘의 실현가능성과 안전성,<br>그리고 마지막으로 본 임상시험설계 자체의 실현가능성 등을 입증하기 위해 계획되었음.
배경	• 고혈압은 뇌경색 환자에서 재발 및 혈관성 사건 발생의 이미 확립된 위험인자임.<br>• 그러나 현 임상진료지침 (JNC 8)은 아래와 같은 실제적이면서 중요한 측면에 대해 간과하고 있음.<br>○ 언제 어떻게 혈압을 측정해야 할지<br>○ 병원에서 측정한 혈압과 집에서 측정한 혈압에 같은 기준을 적용하는 것이 타당한지<br>○ 뇌졸중 환자 유무에 상관없이 같은 혈압기준을 가지고 치료하는 것이 타당한지<br>○ 뇌졸중 환자에서 뇌졸중의 발병기전과 관계없이 동일한 혈압기준을 적용하는 것이 타당한지<br>○ 혈압 조절 그 자체로 충분한지 아니면 혈압을 넘어서는 발병기전이 존재하는지<br>• 약물치료에 생활습관의 교정이 동반되어 되어야 하나 일반적으로 행동변화를 일으키기에는 불충분함.<br>• 집에서 자주 혈압을 측정하는 것이 병원방문시에만 측정하는 것보다 상세하고 신뢰할 수 있는 정보를 줄 것임.
시험설계	• 전향적인, 무작위 배정, 공개, 실현가능성 평가 임상시험

시험일정	IRB 승인일 ~ 2018 년 3 월
치료기간	무작위 배정 후 3 개월
포함기준	1) 발병 일주일 내에 입원하여 뇌영상에서 급성 뇌경색이 확인된 환자<br>2) 만 19 세 이상의 남성 또는 여성<br>- 폐경기 이전이거나 폐경 후 1 년이 경과하지 않은 경우에는 Urine HCG test 가 음성임을 확인해야 함.<br>- 폐경은 특별한 원인없이 최소 1 년 이상 무월경인 것으로 정의함.<br>3) 환자의 상태가 안정되어 본 임상시험에서 규정된 혈압약의 투여가 가능한 환자<br>4) 뇌졸중 증상을 처음으로 인지한 시점에서 최소한 24 시간이 경과한 이후, 연구대상으로 등록되기 전 이틀 동안 측정한 평균 수축기 혈압이 135mmHg 이상인 환자 (혈압약의 처방 여부는 상관없음)<br>5) 경구 약물 투여가 가능한 환자<br>6) 블루투스 기반의 혈압측정계를 사용할 수 있어야 하며 계획된 방문, Breakthrough visit 를 비롯한 중재 계획, 기타 시험 절차를 따를 것으로 합리적으로 기대되는 환자<br>7) 임상시험의 모든 관련된 측면에 대하여 설명을 듣고 동의서에 직접 서명한 환자
제외기준	1) 임신부, 30 일 이내의 출산부, 수유중인 피험자<br>1) 다른 중재적 (interventional) 임상시험에 참여 중인 연구대상자<br>2) 요양/재활병원, 요양원, 혹은 다른 급성기 치료병원으로 전원하는 경우<br>3) 3 개월의 시험기간 동안 경동맥 내막절제술 또는 경동맥 스텐트 시술로 제한되지 않는 다른 혈관 또는 두개내 시술이 계획된 연구대상자; 이러한 시술이 등록 전에 실시되었고 등록 전 48 시간에 환자의 상태가 안정적인 경우 수용 가능하다.

	4) Olmesartan, amlodipine, hydrochlorothiazide 에 알려진 과민반응<br>5) 중증 간질환(예: 복수 또는 혈액응고병증의 징후)이 알려진 환자<br>6) 투석을 요하는 신부전<br>7) 기타 시험자의 의견에 따라, 본 임상시험에의 완전한 참여를 불가능하게 할 수 있는 모든 의학적 상태
연구대상 약물,<br>용량, 투여 방법	개관을 위해 아래 연구진행 흐름도를 참고하세요.<br>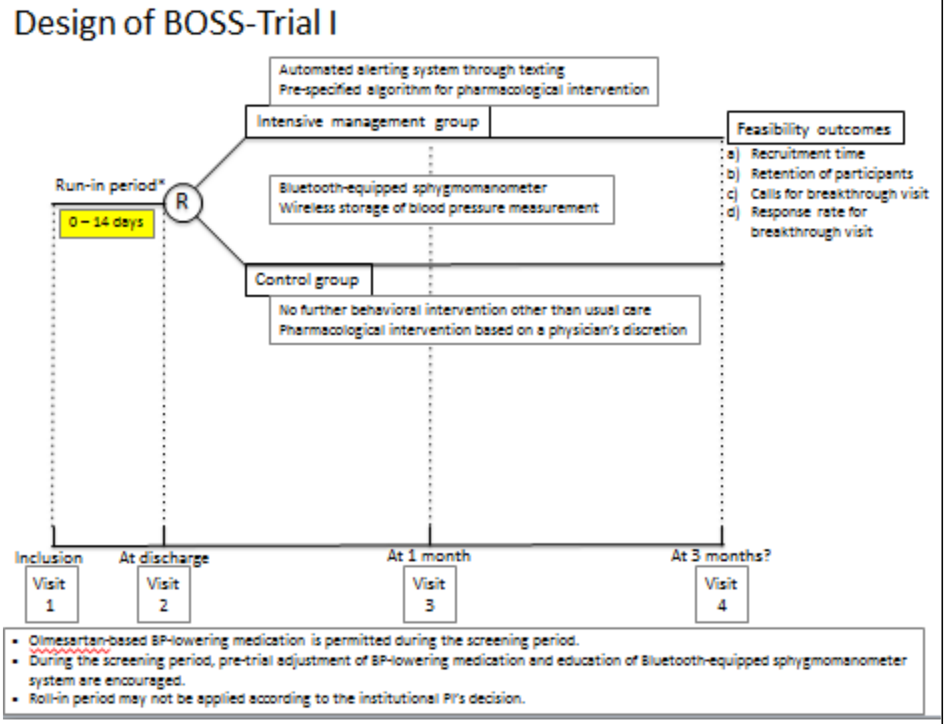<br><br>• 환자 모집은 뇌졸중의 급성기에 시행할 것을 권고함. 시험기간동안 강압제의 변화나 불충분한 혈압조절을 최소화하기 위해 Run-in Period 동안에도 Olmesartan 계열의 약물이 제공될 것임.<br>• 최대 14 일간의 Run-in period 동안 혈압약의 조정 및 블루투스 혈압계의 사용법에 대한 교육이 있을 것임.<br>• 경우에 따라 기관 연구책임자의 결정에 의해 Run-in period 없이 바로 무작위 배정으로 진행할 수 있음.<br>• 무작위 배정은 퇴원 시점에서 이루어지며 연구 약물을 동반한 중재는 퇴원 이후 제공될 것임.<br>• 연구대상자에게 연구대상자의 스마트폰을 통해 메인서버로

	연결될 수 있는 무선 블루투스 혈압계를 제공받을 것임 (치료군과 대조군 모두)<br>• 모든 측정된 혈압 및 맥박은 암호화되어 메인서버에 저장될 것임 (치료군과 대조군 모두)<br>• 혈압약은 Olmesartan 계열의 약물로 구성될 것임. 임상적으로 필요한 경우 베타차단제의 사용은 허용될 것임 (치료군과 대조군 모두에서)<br><br>혈압 측정에 관한 권고사항 (치료군/대조군 모두)<br>• 아침- 기상후 15 분, 식사하지 않고 약 10 분 휴식 후<br>• 저녁- 저녁 식사후 2 시간, 약 10 분 휴식 후<br>• 측정시 2 번 이상 측정하도록<br>• 적어도 주 5 일 이상 (10 번/주 이상)<br><br>치료군:<br>• 연구대상자는 권고되는 횟수 이상으로 혈압을 측정하도록 격려받을 것임. 권고되는 횟수를 채우지 못한 경우 메인서버에서 자동적으로 격려 문자가 발송될 것임.<br>• 측정된 혈압이 바람직한 범위를 벗어날 경우 연구대상자와 참여센터 연구자에게 문자로 이 사실이 통보되어 예정된 방문보다 일찍 외래를 방문하도록 할 것임 (돌발성 방문).<br>• 혈압약의 조절은 아래에서 제시된 알고리즘을 따르도록 함.<br>• 행동 강화를 위한 알고리즘 (치료군)<br>○ 혈압측정횟수가 주 10 회 미만이면 문자 메시지 전송<br>○ 혈압측정획수가 주 6 회 미만이면 전화 접촉<br>○ 집에서 적정혈압의 범위는 110-135mmHg 로<br>○ 주 6 회 이상 혈압이 측정되었고 이중 적정범위를 이탈한 횟수가 50%를 초과하였고 무작위배정 후 2 주를 경과하였으면 전화접촉함. 참여센터 연구자의 판단에 따라 임상적으로 필요한 경우 돌발성 방문을 계획할 수 있음.<br>○ 주 10 회 이상 혈압측정이 이루어 졌고 적정범위 이탈이 없는 경우 혈압조절이 잘되고 있음을 알리는 문자 메시지를 전송함.<br>• 약물 조절을 위한 알고리즘 (치료군)

	○ 제 1 단계:<br>2 일 동안 평균 수축기 혈압이 150mmHg 이하인 경우 Olmesartan 20 mg 로 시작<br>2 일 동안 평균 수축기 혈압이 150mmHg 를 초과한 경우 Olmesartan 40 mg 로 시작<br>○ 제 2 단계: olmesartan 20 mg or 40 mg + amlodipine 5 mg<br>○ 제 3 단계: olmesartan 20 mg or 40 mg + amlodipine 10 mg<br>○ 제 4 단계: olmesartan 20 mg or 40 mg + amlodipine 10 mg + hydrochlorothiazide 12.5 mg<br>○ 임상적으로 의미있는 혈압변동성이 관찰되는 경우 hydrochlorthiazide 의 중단이나 olmesartan 의 감량을 고려함.<br>○ 임상적으로 필요한 경우 베타차단제의 사용이 허용됨.<br>○ 정규 외래 방문이든 돌발성 방문이든 관계없이 상술한 원칙에 따를 것이 권고됨.<br><br>대조군:<br>• 행동강화를 위한 자동화된 문자메시지 발송을 포함한 알고리즘이 적용되지 않을 것임.<br>• 혈압약의 조절은 Olmesartan 계열의 약물 기반으로 이루어지되 담당의사의 재량에 따라 이루어 질 것임.
임상시험의 목적	• 실제 임상에서 블루투스 혈압계과 메인서버를 무선으로 연결하여 활용하는 것이 가능한지를 입증함.<br>• 미리 결정된 혈압의 적정범위, 혈압관리 알고리즘, 문자메시지를 이용한 행동강화요법 등으로 이루어진 혈압관리전략이 실현가능한지를 입증함.<br>• 혈압변동성 지표을 치료 지침으로 활용하는 제 3 상 임상시험의 가능성에 대한 정보를 얻고자 함.
유효성 평가변수에 대한 평가	• 실현가능성 평가변수<br>○ 연구대상자 모집 시간<br>○ 등록된 연구대상자의 연구 참여 완료율<br>○ 돌발성 방문 요청 빈도

	○ 돌발성 방문 요청 응답률<br>○ 혈압측정 기준을 만족하는 환자의 분율<br><br>• 이차 유효성 평가변수<br>○ 적정범위 이탈율 (OOR, 수축기 혈압에 대해서 아래 참조)<br>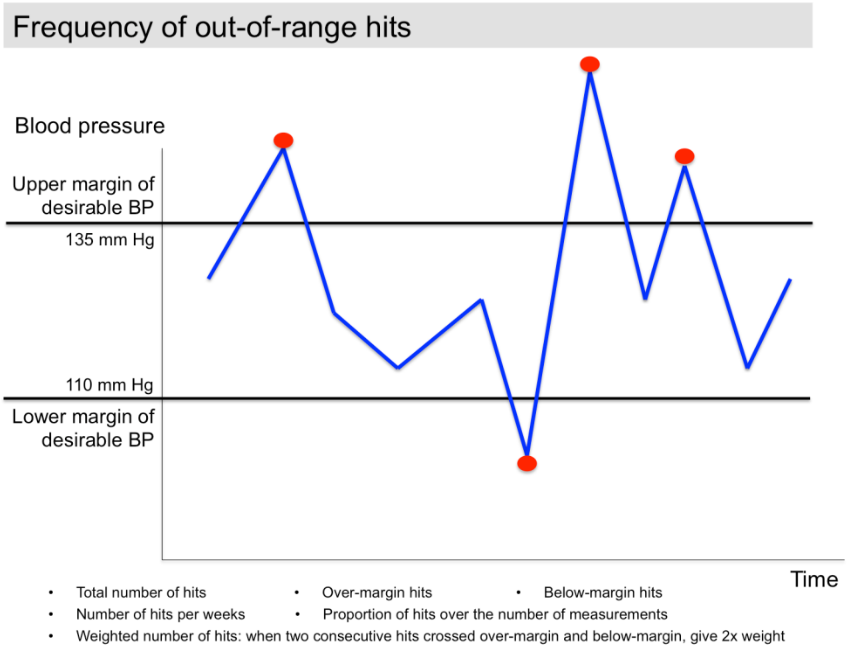<br>○ 가중된 적정범위 이탈율: 연구대상자 별로 적정범위 이탈 횟수를 평가 횟수로 나눈 분율(%)을 전체 연구대상자에 대해 평균을 구한 것. 두번 연속해서 범위에서 벗어 나면 2 를 곱해 가중치를 부여함.<br>○ 심혈관계 사건 발생율
안정성 평가 변수에 대한 평가	• 어지럼증, 낙상, 기타 기립성 저혈압관련 사건의 발생<br>• 혈압과 관련이 있을 것으로 예상되는 기타 이상반응 발생율<br>• 사망률
참여 환자수에 대한 통계적인 고려	• 혈압 관리 전략에 대한 실현 가능성을 확인하는 임상시험으로 별도의 통계적인 고려없이 참여환자수가 결정됨.<br>• 치료군 30; 대조군 30
윤리적인 고려	• 임상시험 시작 전 IRB 의 동의를 득할 것.<br>• 참여 연구대상으로부터 서면동의를 받을 것.
기대되는 결과	• 무선 블루투스 혈압계를 활용하고 Olmesartan 계열 약물을

	기반으로 하는 적극적인 혈압조절이 뇌졸중 환자에서 집에서 측정한 혈압이 치료범위를 벗어나는 것을 줄일 수 있다.
연구비 총액	327,117,470 원

2. 연구 흐름도

방문	Visit 1		Visit 2	Visit 3	Visit 4
단계(Day)	Screening	Run-in period (-14 to 0)	Randomization (0)	Month 1 (30)	Month 3 (90)
방문 허용 기간 (일)			Screening 으로부터 14 일 이내	±10	±14
				Breakthrough visit	
평균 혈압을 포함 한 선정/제외 기준 확인	V				
*임신반응검사	V				
혈압 측정에 대한 교육		V			
블루투스 모듈 연결 점검		V			
선정/제외 기준 확인			V		
동의서	V				
인구 통계학적 정보	V				
병력 및 뇌졸중 위험 인자	V				
치료 및 뇌졸중 진단	V				
병용 금기 약물 확인	V			V	V
혈압측정 및 데이터 전송 충실도				V	V
혈압 강하제 복용의 순응도				V	V
임상시험의 결과 변수 수집				V	V
부작용, 이상 반응 수집				V	V
혈압과 관련된 약물 변경				V	

*폐경기 이전이거나 폐경 후 1 년이 경과하지 않은 경우에는 Urine HCG test 실시

3. 연구 계획서에서 사용된 약자

ALCOA	Attributable, Legible, Contemporaneous Original, Accurate	귀속성, 읽을수 있는, 동시성, 원본, 정확성
DMP	Data Management Plan	데이터 관리계획
eCRF	Electronic case report form	전자증례기록서
Home BP	Home blood pressure	자택 측정 혈압
ICH	International Conference on Harmonisation	의약품국제조화회의
IRB	Institutional Review Board	생명윤리위원회
ITT	Intent-To-Treat	
IWRS	Interactive web-based response system	자동웹응답시스템
KGCP	Korea Good Clinical Practice	임상시험관리기준
MAGIC	MRI-based Algorithm for Acute Ischemic Stroke Subtype Classification	자기공명영상 기반 뇌졸중 분류법
NIHSS	National Institute of Health Stroke Scale	미국립보건원 뇌졸중척도
Office BP	Office blood pressure	진료실 측정 혈압
OOR	Out of range	적정범위이탈
PI	Principal Investigator	연구책임자
PP	Per Protocol	
PT	Preferred Term	
SBP	Systolic blood pressure	수축기혈압
SMS	Short message service	단문메세지
SOC	System Organ Class	
SOP	Standard Operating Procedure	표준작업지침서

4. 서론

고혈압은 뇌졸중을 포함한 각종 심혈관계 질환의 중요한 위험인자이다.[1-3] 뿐만 아니라 고혈압은 뇌졸중 이후 기능적 회복을 지연시키며 혈관성 질환의 재발 위험을 증가시키는 요인으로 알려져 있다.[4, 5] 따라서 뇌졸중의 예방뿐만 아니라 뇌졸중 이후의 임상 경과를 호전시키기 위하여, 혈압을 엄격하게 조절하는 것은 매우 중요하다.[4] 고혈압 관리 전략이 고도화되고[6] 다양한 치료 전략이 개발되면서,[7, 8] blood pressure control rates 는 점차 향상되고 있다.[9, 10] 그렇지만 뇌졸중 환자의 고혈압 관리에 있어 제기된 많은 질문들이 아직 충분히 해명되지 않은 채 남아 있다.

우선적으로, 현재 뇌졸중 환자에서 혈압을 조절하고 이를 유지하기 위한 알고리듬은 현재 제시되어 있지 않다. 대부분의 임상 진료 지침은 최신의 고혈압 조절 지침을 참고하라는 권고를 하고 있으나, 개별 환자의 뇌졸중 중증도, 기능적 의존 상태, 뇌로의 혈류 공급 상태는 각각 달라 개별적인 고혈압 조절 목표가 필요한 상황이 흔히 발생한다. 또한 혈압 강하제를 복용하고 있는 환자에서 혈압이 상승 혹은 감소할 때 이에 따른 혈압 강하제의 증량 혹은 교체에 대해서도, 아직 충분한 근거에 기반한 변경 전략은 고안되지 않았다. Olmesartan 은 미세알부민뇨의 발생을 억제하고,[11] 죽상경화증의 진행을 예방하는 효능을 갖고 있으며,[12] 칼슘 채널 길항제 등과 병용 투여할 때 혈압 감소 효과가 더 크며 이 알려져 있다.[13, 14] 따라서 칼슘 채널 길항제 및 이뇨제 등과 다양한 조합이 가능한 olmesartan 에 기반한 혈압 강하제 조절 알고리듬을 통해, 객관적 지표에 근거한 혈압 조절이 가능할 수 있을 것이다.

두 번째로, 퇴원 이후 병원에 거주하지 않는 환자에서 혈압의 측정 횟수, 혈압 측정치의 해석, 혈압의 상승 혹은 저하에 따른 혈압 강하제의 조절 전략에 대해서도 아직 신뢰할 수 있는 임상 자료가 존재하지 않는다. 일반적으로 office BP 에 비하여 home BP 측정치는 다소 낮게 측정된다.[15] 따라서 이에 home BP 의 측정치가 있는 환자는 이에 맞추어 혈압의 조절 목표와 치료 전략을 수정하는 것이 이론적으로 적절할 것이나, 이러한 혈압 관리 전략은 아직 임상에서 검증된 바 없다. 환자의 혈압은 circadian rhythm, fatigue, daily activity 등에 의하여 다양한 시간을 단위로 한 변동성을 보이게 마련인데, 이러한 변동성은 office BP 에 기반한 혈압 조절 전략으로는 충분히 고려할 수

없을 수밖에 없다.[16,17] IT 기술의 발전에 의하여, 혈압계를 블루투스 기술로 스마트폰을 통하여 중앙 서버와 결합할 수 있게 되었다. 최근 영국에서 TIA 나 뇌졸중으로 약물치료가 필요한 환자를 대상으로 블루투스 기반의 telemetric home BP monitor 를 이용하여 혈압을 수집, Day-to-Day home BP variability 에 관한 연구를 성공적으로 수행하여 결과를 보고한 바도 있다.[18] 4000 만명이 넘는 국내 스마트폰 가입자 수를 감안하면, 이러한 데이터 수집을 큰 추가 비용 없이 현실화할 수 있을 것이라 생각된다.

세 번째로, 고혈압을 가진 뇌졸중 환자에게 지속적으로 혈압을 관리할 수 있도록 유도하는, 개별 환자 대상의 맞춤형 치료로 환자를 유인하는 전략이 아직 충분하지 않다는 점이다. 혈압은 non-invasive measurement 가 가능하며 측정 기기의 가격도 높지 않다. 따라서 뇌졸중 환자가 가정에서 자주 혈압을 측정하고 이에 대해 관심을 기울이도록 하며, 비정상적인 혈압 측정치가 누적될 때 병원을 찾도록 하는 behavioral intervention 이 효과적일 것이라 추측할 수 있다. 일부 무작위 배정 임상시험에서 개별 환자에 대한 전화 상담 및 positive-affect intervention 을 적용한 결과, 혈압 강하제에 대한 환자의 adherence 가 증가한다는 보고가 있었다.[9,19] 그렇지만 이 임상시험은 환자의 행동에 대한 intervention 에 그쳤을 뿐, 환자의 home BP measurement 에 근거한 pharmacological modification 까지는 진행하지 못하였다는 한계를 갖고 있다.

5. 연구 목적

본 임상시험은 다음과 같은 목적을 달성하기 위하여 수행된다.

- 일차 목적: 다음과 같은 복합적 혈압 관리 전략의 실현 가능성을 검증한다.

1. 블루투스 기반의 혈압측정계와 원격지 주서버를 연결한 혈압 모니터링 체계
2. 미리 정해진 목표 혈압 범위 및 이를 달성하기 위한 Olmesartan 기반의 혈압치료 알고리듬
3. SMS (short message service) 등을 이용한 행동강화기법 (Behavioral Intensification)

- 이차 목적: 혈압 변동성 지수를 치료 지침으로 이용하는 제 3 상 임상시험의 설계에 필요한 정보 (Efficacy, safety, expected sample size)를 획득한다.

6. 연구 설계

1) 개요

본 임상시험은 급성 뇌경색으로 입원치료를 받는 환자들 중 초급성기를 지난 상태(뇌졸중 증상을 처음으로 인지한 시점에서 최소한 24 시간이 경과한 시점)에서 평균 수축기 혈압이 135mmHg 가 넘는 고혈압 환자를 대상으로 원격 혈압 모니터링 체계 및 Olmesartan 기반의 혈압치료 알고리즘을 특징으로 하는 혈압 관리 전략의 실현 가능성을 검증하는 것을 주요 목적으로 한다. 비교성의 확보를 위해 대조군에서도 Olmesartan 기반의 혈압약이 제공될 것이다. 초급성기가 막 지난 환자들이 모집될 것이며 등록 후 0-14 일 동안(Run-In period) Olmesartan 계열의 혈압약이 투여 되면서 블루투스 기반의 혈압측정계에 대한 사용법을 교육 받을 것이다. 퇴원시점에서 무작위 배정이 일어나며 퇴원시 집중관리군(Intensive management group)에서는 SMS 서비스와 Breakthrough visit 에 대한 안내가 제공될 것이다. 대조군에서는 퇴원 1 달째 다시 내원하여 한달 동안 측정된 혈압을 바탕으로 혈압약을 조정받고 다시 3 개월째 방문하면 연구가 종료된다. 집중관리군에서는 퇴원 1 개월, 3 개월째 방문 외에 원격 혈압 모니터링에서 정해진 기준을 넘어가는 경우, SMS, 직접전화, Breakthrough visit 와 같은 중재를 받게 될 것이다.

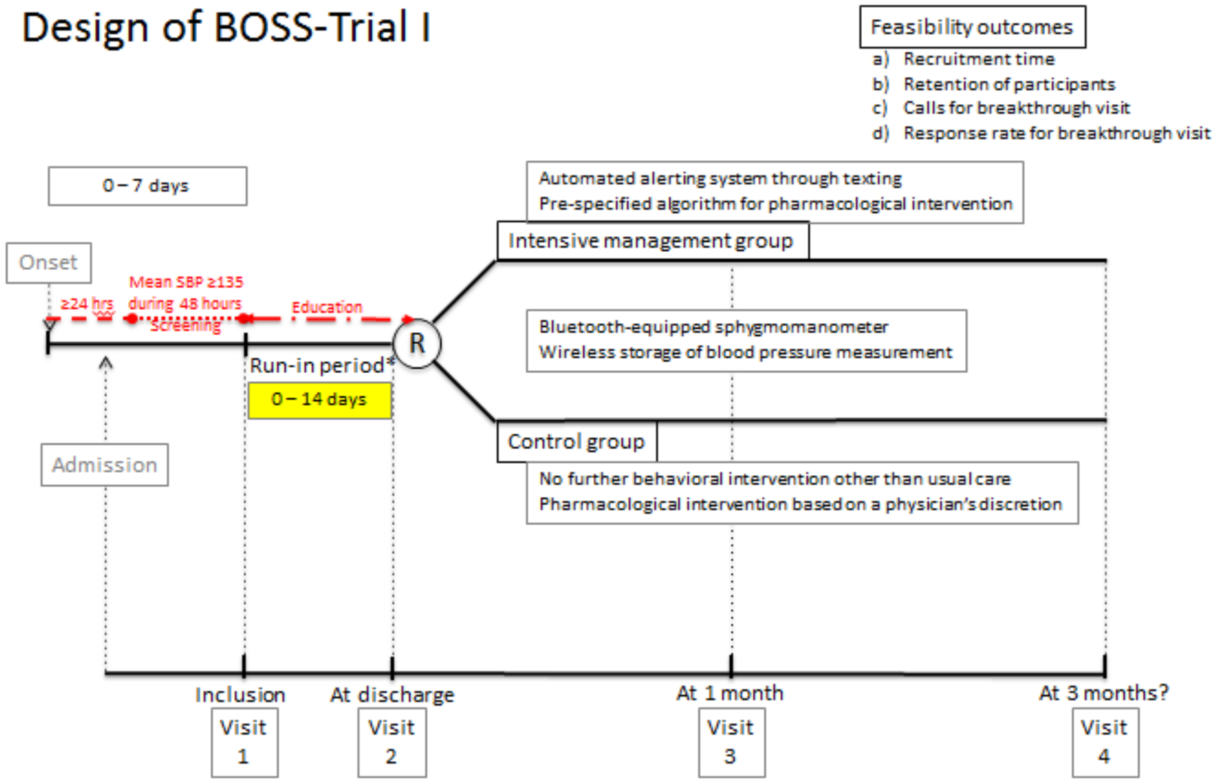

- Olmesartan-based BP-lowering medication is permitted during the screening period.
- During the screening period, pre-trial adjustment of BP-lowering medication and education of Bluetooth-equipped sphygmomanometer system are encouraged.
- Run-in period may not be applied according to the institutional PI's decision.

2) 연구 대상

이 시험은 적절한 연구대상자가 등록되어야만 그 목적을 달성할 수 있다. 다음의 포함 및 제외 기준은 임상시험계획서에 따른 중재가 적절하다고 생각되는 연구대상자를 선택하기 위하여 설계된 것이다. 이 임상시험계획서가 특정한 연구대상자에게 적절한지 결정할 때에는 모든 관련된 의학적 및 비의학적 상태를 고려해야 한다.

포함 기준: 연구대상자가 시험에 선정되기 전 시험자의 임상시험 팀에서 적절한 자격을 갖춘 사람이 연구대상자의 적합성을 검토하고 기록해야 한다. 연구대상자는 시험에 등록되기에 적합하려면 다음의 선정기준들을 모두 만족해야 한다

(1) 발병 일주일 내에 입원하여 뇌영상에서 급성 뇌경색이 확인된 환자

(2) 만 19 세 이상의 남성 또는 여성

- 폐경기 이전이거나 폐경 후 1 년이 경과하지 않은 경우에는 Urine HCG test 가 음성임을 확인해야 함.
- 폐경은 특별한 원인없이 최소 1 년 이상 무월경인 것으로 정의함.

(3) 환자의 상태가 안정되어 본 임상시험에서 규정된 혈압약의 투여가 가능한 환자
(4) 뇌졸중 증상을 처음으로 인지한 시점에서 최소한 24 시간이 경과한 이후, 연구대상으로 등록되기 전 이틀 동안 측정한 평균 수축기 혈압이 135mmHg 이상인 환자 (혈압약의 처방 여부는 상관없음)
(5) 경구 약물 투여가 가능한 환자
(6) 블루투스 기반의 혈압측정계를 사용할 수 있어야 하며 계획된 방문, Breakthrough visit 를 비롯한 중재 계획, 기타 시험 절차를 따를 것으로 합리적으로 기대되는 환자
(7) 시험의 모든 관련된 측면에 대하여 설명을 듣고 동의서에 직접 서명한 환자

제외기준: 다음 중 어떠한 것에라도 해당되는 연구대상자는 시험에 선정되지 않을 것이다

(1) 임신부, 30 일 이내의 출산부, 수유중인 피험자
(2) 다른 중재적 (interventional) 임상시험에 참여 중인 연구대상자
(3) 요양/재활병원, 요양원, 혹은 다른 급성기 치료병원으로 전원하는 경우
(4) 3 개월의 시험기간 동안 경동맥 내막절제술 또는 경동맥 스텐트 시술로 제한되지 않는 다른 혈관 또는 두개내 시술이 계획된 연구대상자; 이러한 시술이 등록 전에 실시되었고 등록 전 48 시간에 환자의 상태가 안정적인 경우 수용 가능하다.
(5) Olmesartan, amlodipine, hydrochlorothiazide 에 알려진 과민반응
(6) 중증 간질환(예: 복수 또는 혈액응고병증의 징후)이 알려진 환자
(7) 투석을 요하는 신부전
(8) 기타 시험자의 의견에 따라, 본 임상시험에의 완전한 참여를 불가능하게 할 수 있는 모든 의학적 상태

Screening failure: Run-in period 동안 다음 중 어느 하나라도 해당되는 연구대상자는 연구에서 탈락하며 무작위 배정되지 않을 것이다.

(1) 본 임상시험에서 규정된 혈압약의 투여 후 과민반응을 비롯한 예측하지 못한 이상반응이 발생하여 임상시험의 지속이 힘들다고 의학적으로 판단되는 경우
(2) Run-in period 동안 신경학적 악화가 발생하거나 기타 임상시험의 지속이 힘들다고 연구책임자가 판단하는 경우

(3) 임상시험에 계획된 대로 블루투스 기반의 혈압측정계의 사용이 힘들 것으로 판단되는 경우
(4) 연구대상자가 연구에 계속 참여를 거부하는 경우

3) 무작위 배정 방법 및 절차

본 임상시험에 등록된 연구대상자들은 퇴원 시에 집중 관리군(intensive management group)과 대조군(control group) 중 하나에 배정된다. 연구대상자들을 각 군에 할당 시 개입될 수 있는 bias 를 막고, 알고 있거나 모르는, 연구대상자의 인구학적 및 기초 특성들이 두 군 간에 균형 있게 분포될 가능성을 증가시켜 군 간 비교성을 높이기 위해 무작위배정을 실시한다.

모든 포함기준을 만족하고 어떠한 제외기준에도 해당되지 않아 연구참여에 적합한 것으로 판단되는 연구대상자들은 사전에 의학통계학자에 의해 생성된 무작위배정 표에 근거해 두 군에 무작위배정 된다. 따라서 연구대상자에 대한 적합성 여부는 연구대상자가 각 군에 무작위배정 되기 전에 판단되게 된다.

무작위배정 방법으로는 두 군 간 균형 있는 분포를 보장하기 위해 본 임상시험 참여기관 별로 층화 한 뒤 특정 크기의 블록을 사용하여 무작위배정을 하는 층화 블록 무작위배정(stratified block randomization) 방법을 사용한다. 무작위 배정은 SAS(Ver. 9.2, SAS Institute, Cary, NC, USA)의 PLAN 프로시저(PROC PLAN Procedure)를 사용하며, seed 는 임상시험 참여기관 별로 다르게 지정한다. 따라서 연구대상자들은 각 참여기관(3 개) 층 내에서 집중 관리군과 대조군 중 하나에 각각 1:1 의 비율로 무작위배정 될 것이다.

무작위배정 절차는 다음과 같다: 연구대상자는 본 임상시험의 선정기준과 제외기준을 만족하면, 해당 연구대상자를 Web 상에 등록한 후, run-in period 단계를 거치도록 한다. Run-in period 에서 연구대상자가 screening 요구 조건을 만족하게 되면, 연구자(혹은 연구자가 지정한 연구담당자)는 해당 연구대상자에게 블루투스 모듈이 장착된 혈압계 사용법에 대해 설명한 후, web 상에서 설명이 완료되었음을 등록한다. 해당 연구대상자는 IWRS(interactive web-based response system)를 통해 집중 관리군 혹은 대조군 중 하나로 무작위 배정되게 된다. 무작위 배정 후, 연구자(혹은 연구자가 지정한 연구담당자)는 집중 관리군으로 배정된 연구대상자들에 대해서 가정 내 혈압측정 방법 및 향후 관리 절차에 대해 설명한다.

연구대상자가 임상시험을 중단하는 경우, 해당 연구대상자에게 배정된 무작위배정 번호는 다시 사용할 수 없으며, 해당 연구대상자 역시 본 임상시험에 다시 참여할 수 없다.

4) 중재적 개입

i. 임상시험 기간 중 혈압 관리의 기본적 원칙

- 연구대상자는 본인의 스마트폰과 연동되는 블루투스 혈압계를 지급받게 된다. 이를 통해 모든 혈압 및 맥박 정보는 암호화된 상태로 메인서버에 저장된다. (스마트폰과 연동되는 블루투스 혈압계는 이전 연구에서 적용된바 있다.;Webb et al. Stroke. 2014.)
- 본 연구에서 혈압조절을 위해 사용되는 약물로는 olmesartan 을 기반으로 한 항고혈압제를 이용한다.
- 임상적으로 필요한 베타차단제는 사용을 허가한다.
- 혈압약은 run-in period 에서부터 제공된다. (Inclusion 이전에 혈압약 사용이 필요한 경우 가급적 olmesartan 기반 혈압약 사용을 권장한다.)
- 혈압약 시작
  - Inclusion 직전 2 일간 평균 수축기혈압이 ≤ 150 mm Hg 인 경우 olmesartan 20mg 을 사용한다.
  - Inclusion 직전 2 일간 평균 수축기혈압이 > 150 mm Hg 인 경우 olmesartan 40mg 을 사용한다.
- 연구기간 중 목표혈압
  - Office SBP < 140 mm Hg
  - Home SBP 110-135 mm Hg

연구자는 블루투스 혈압계를 통해 측정된 Home SBP 이외에도, Office SBP 등 추가 정보를 토대로 종합적으로 혈압조절 여부를 판단하도록 한다

ii. 집중 관리군을 위한 혈압 강하제 조절 알고리듬 및 행동 강화를 위한 개입

(1) 혈압 강하제 조절 알고리듬

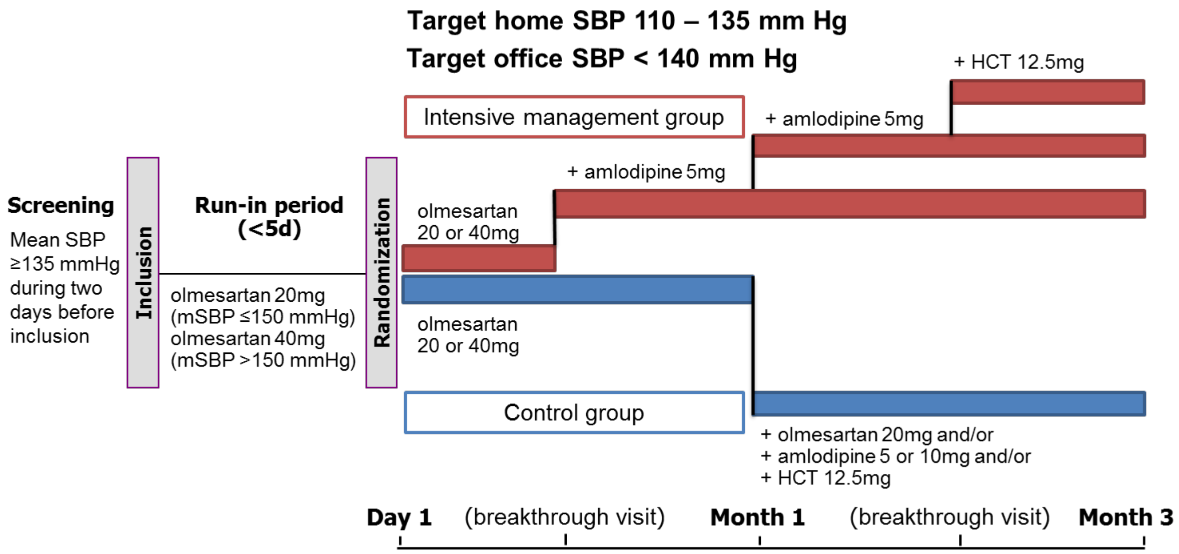

- 본 연구에서 사용된 혈압 조절 알고리듬은 유사한 target BP 를 목표로 olmesartan 기반의 혈압 조절을 사용한 선행연구들 (Sanford et al. Drugs. 2009.; Neutel et al. J Clin Hypertens. 2004.)에서 사용된 알고리듬을 변형한 것이다.
- 매방문시 혈압약 조절방법
  - 1 단계: amlodipine 5mg 추가 (olmesartan 20 mg or 40 mg + amlodipine 5 mg)
  - 2 단계: amlodipine 5mg 추가 (olmesartan 20 mg or 40 mg + amlodipine 10 mg)
  - 3 단계: hydrochlorothiazide 12.5 mg 추가 (olmesartan 20 mg or 40 mg + amlodipine 10 mg + hydrochlorothiazide 12.5 mg)
  - 3 단계 알고리듬 적용으로도 혈압이 조절되지 않고, 현재 처방되고 있는 olmesartan 용량이 20mg 인 경우 olmesartan 용량을 40mg 으로 증량할 수 있다.
- 3 단계 알고리듬 적용과 olmesartan 40mg 증량으로도 혈압이 조절되지 않는 경우 연구자의 임상적 판단으로 혈압약제를 추가 할 수 있다.
- 혈압이 목표혈압범위보다 지나치게 낮은 경우 (ex, Home mean SBP < 110 mm Hg) 추가된 혈압약을 역순으로 제거하거나, 현재 처방된 혈압약의 용량을 줄일 수 있다.

- 정규방문이나 breakthrough visit 나 혈압약 조절이 필요한 경우 본 혈압약조절 알고리듬을 따라야 한다.

(2) 행동 강화를 위한 개입

- 연구대상자는 본 연구에서 권장하는 횟수 이상(6-5)에 기술함)으로 자가혈압측정을 시행해야 한다. 만약 정해진 횟수 이하로 측정하는 경우 메인서버에서 자동으로 자가혈압측정을 독려하는 문자메세지를 연구대상자에게 보내게 된다
- 자가혈압측정이 일주일에 10 회 미만인 경우 연구대상자 및 연구자에게 문자메세지를 발송한다.
- 자가혈압측정이 일주일에 6 회 미만인 경우 연구간호사가 전화연락을 시행한다.
- 측정된 자가혈압이 목표범위 (수축기혈압 110-135 mm Hg)를 일주일에 50% 이상 벗어나는 경우 연구간호사가 전화연락을 하여 혈압약 복약상태 등을 점검한다.
  - 일주일에 최소 6 회 이상 측정된 경우에 한한다.
  - 무작위배정 후 2 주경부터 시행한다.
  - 연구자의 판단에 따라 혈압약조절 등의 필요성이 있는 경우 전화연락을 통해 breakthrough visit 를 권유할 수 있다.
  - 기타 조기방문이 필요하다고 판단되는 경우에도 breakthrough visit 를 권유할 수 있다.
- 일주일 간 자가혈압측정 횟수가 10 회 이상이고 측정된 자가혈압들이 목표범위를 벗어난 경우가 없으면 부지런한 혈압측정 및 우수한 혈압조절에 대한 감사 및 축하의 내용이 담긴 문자메세지를 연구대상자 및 연구자에게 발송한다.
- 자가혈압측정 횟수는 환자 자의로 규정된 오전 1 회, 오후 1 회의 측정 횟수를 초과하여 오전 혹은 오후에 2 회 이상 측정하더라도 오전 혹은 오후에 1 회 측정한 것으로 간주한다.
- 집중관리군에서의 혈압약 조절은 본 연구계획서에 명시된 알고리듬에 따라 시행해야 한다.

iii. 대조군의 혈압 관리 원칙

- 대조군에게는 자가혈압 측정횟수 및 자가혈압범위에 대한 문자메세지 발송되지

않는다.

- 대조군에게는 명확한 혈압약조절 알고리듬이 제시되지 않는다.
- 혈압약조절 방법 (1 개월 방문시)
    - Office BP 또는 Home BP 를 고려하여 임상의사의 판단에 따라 혈압약을 조절한다.
    - 혈압약조절시 권유되는 약물
        - Olmesartan 20mg 추가 and/or
        - Amlodipine 5mg 또는 10mg 추가 and/or
        - Hydrochlorothiazide 12.5mg 추가
    - 혈압이 목표혈압범위보다 지나치게 낮은 경우 (ex, Home mean SBP < 110 mm Hg) 현재 처방된 혈압약의 용량을 줄일 수 있다.

iv. 약물 공급 및 수불관리

임상시험조정자가 각 임상시험 실시기관에 Olmesartan 을 공급할 것이다.
관리약사는 의약품임상시험관리기준(GCP) 지침과 국내 또는 지역 요건을 준수하여 시험약의 수불관리(교부, 재고관리 및 기록유지)를 담당할 것이다.
어떠한 상황에서도 시험자에게 이 임상시험계획서에서 지시하는 것 이외의 목적으로 시험약을 사용하도록 허가하지 않을 것이다. 시험에 등록되지 않은 어떠한 개인에게도 시험약 공급품을 교부하지 않을 것이다.
시험기관은 다음과 같은 기록을 정확하고 시기 적절하게 유지해야만 한다: 시험약의 수령, 연구대상자에게 시험약 공급품의 교부, 연구대상자가 반납하거나 시험기관으로 배송되었지만 연구대상자에게 교부되지 않은 미사용 시험약 공급품의 수거 및 일치 확인 그리고 일치 확인된 시험약 공급품의 폐기. 여기에는 다음이 포함되지만, 이에 국한되지는 않는다.

- 시험약의 수령 기록
- 시험약 교부/반납 일치 확인 기록지
- 시험약 수불관리 기록지
- 모든 운송 서비스 영수증
- 시험기관에서 시행한 시험약의 모든 폐기에 대한 폐기 인증서.

연구대상자는 사용하지 않은 모든 시험약 공급품과 사용된 시험약의 비어있는 용기를 관리약사에게 반납한다.
연구대상자로부터 반납 된 시험약은 각 시험기관에서 모두 폐기하며 폐기와 관한 기록을 임상시험조정자에게 제출한다.
시험기관 방문 중과 시험 완료시에 약물 수불관리를 검토한다.

5) 가정에서의 혈압 측정

- 모든 연구대상자의 혈압은 블루투스가 장착된 동일한 혈압계로 측정되며 연구대상자의 핸드폰을 통해 주 서버에 저장한다.
- Run-in 기간 동안 혈압계의 사용법 교육 및 연구대상자 핸드폰과의 연동과정을 점검한다.

i. 측정 횟수

- 아침과 저녁, 하루 2 회 측정한다.
- 각각의 측정 시 1 분 이상의 간격으로 2 회 측정하며, 2 회 측정의 평균치를 취한다. (2013 ESH/ESC guidelines)
- 매일 같은 방식으로 혈압을 측정하며, 적어도 일주일에 5 일이상 (10 회 측정/주) 측정을 목표로 한다.

ii. 측정 방법

- 조용한 공간에서 편하게 앉아 혈압을 측정하는 팔과 등을 기댄 채 5 분 휴식 후 혈압을 측정
- 혈압의 측정은 오른손 사용자의 경우 좌측, 왼손 사용자의 경우 우측 팔에서 하며, 주로 사용하는 팔이 마비완(paralytic arm)일 경우 반대측에서 측정한다. 좌우 팔의 혈압차이가 20 mmHg 를 넘을 경우 높은 쪽 팔에서 재도록 한다. (Webb et al. Stroke. 2014)
- 아침혈압은 기상 후 최소 15 분이 지나고 측정하며 식사를 하지 않은 상태여야 한다.
- 저녁혈압은 저녁식사 후 최소 2 시간 경과 후 측정한다.
- 혈압 측정 전 데이터가 전송되는 연구대상자의 휴대폰 전원이 켜져 있고, 같은 공간(방)에 위치하는지 확인한다.

6) 임상시험 관리 시스템

본 임상시험 관리 시스템은 다음과 같은 요소로 구성된다.

- Screening 및 run-in period / randomization system

- 혈압 측정 자료의 보관 및 자동화된 알람 시스템
- CRF 에 기반한 임상시험 자료 보관 및 관리
- 임상시험 진행 현황의 모니터링을 위한 유저 인터페이스

i. Screening 및 run-in period / randomization system

임상시험 관리 시스템은, 임상시험 대상가 아직 randomization 을 받기 이전인 screening 및 run-in period 에서부터 예비 대상자를 등록하고 블루투스 모듈을 장착한 혈압계와 스마트폰의 연동 및 혈압 자료 전송 과정이 원활하게 이루어지도록 해야 한다. Screening 및 run-in period 에 포함된 대상자가 randomization 을 받지 않고 사전에 탈락되는 경우, 이 대상자에 관련된 모든 정보는 임상시험 관리 시스템에서 삭제되나 screening log 는 유지된다.

Randomization 은 IWRS 접속을 통하여 이루어진다.

ii. 혈압 측정 자료의 보관 및 자동화된 알람 시스템

임상시험 과정에서 수집된 혈압 측정치는 암호화된 형태로 보안이 유지되는 서버에 일시 보관된다. 이 때 서버에 보관되는 정보는 익명화된 대상자 식별 정보, 대상자의 연구 참여 시 등록 병원, 대상자의 소속 그룹(시험군 / 대조군 여부), 대상자의 혈압 조절 목표, 대상자의 혈압 강하제 처방 정보, 누적된 혈압 측정치 및 측정 시간이다. 서버의 데이터베이스는 사전에 규정된 절차에 따라 각 대상자의 주간 혈압 측정 횟수, 아침/저녁 측정 여부 주간 혈압 측정일을 계산하여 시험 책임자 및 등록 병원의 시험자에게 알린다. 또한 각 대상자의 혈압 측정치가 사전에 규정된 혈압 조절 목표를 벗어나는지 계산하여, 목표를 벗어나는 대상자가 발생하면 절차에 따라 시험자에게 알린다.

서버에 저장된 시험 대상자의 정보는, 대상자의 연구 참여 기간이 종료됨과 동시에 실물 형태로 출력하며 이 때 서버에 저장된 정보는 완전히 삭제된다. 본 임상시험의 마지막 환자가 추적 관찰을 종료함과 동시에 서버에 저장된 모든 기록은 삭제된다.

iii. CRF 에 기반한 임상시험 자료 보관 및 관리

임상시험 관리 시스템은 CRF 로 본 임상시험에서 수집되는 자료를 보관한다.

iv. 임상시험 진행 현황의 모니터링을 위한 유저 인터페이스

임상시험 관리 시스템은 임상시험의 연구 코디네이터 및 연구자가 임상시험의 진행 현황 및혈압 자료 수집 현황을 모니터링할 수 있는 유저 인터페이스를 제공한다. 이를 바탕으로 연구 코디네이터는 정기적으로 임상시험의 현황 및 혈압 자료 수집 현황을 연구자에게 보고한다.

7) 결과 변수

본 임상시험은 뇌경색으로 인한 뇌졸중 환자들을 대상으로, 퇴원 후 이들에 대한 기존 고식적인 관리 전략(conventional management strategy)에 비해 smartphone 을 통한 wireless Bluetooth-equipped sphygmomanometer system 을 사용하는 집중 관리 전략(intensive management strategy)의 사용 가능성을 평가하고, 이를 토대로 향후 집중 관리 전략의 유효성을 평가하는 제 3 상 임상시험으로의 진행 가능성을 파악하기 위한 feasibility 임상시험이다.

이에 따른 본 연구의 일차 실현가능성 변수들(primary feasibility endpoints)은 다음과 같다.

1) 연구대상자 모집시간 (recruitment time to pre-specified number of subjects): 각 시험군 별, 첫 번째 환자의 등록일과 최종 환자가 무작위배정일 간 차이 (day)
2) 등록된 연구대상자의 연구참여 완료율 (retention of included participants): 각 시험군 별, 무작위 배정된 연구대상자들 중 무작위 배정 후 3 개월째 follow-up 방문을 완료한 연구대상자 비율 (%)
3) 돌발성 방문 요청 빈도 (frequency of calls for breakthrough visit): 집중 관리군에서 환자 당 돌발성 방문이 요청된 평균 횟수 (= 집중 관리군 내 돌발성 방문 요청 총 횟수 / 집중 관리군 해당 인원 수) (mean, SD)
4) 돌발성 방문 요청 응답율 (rate of patients who responded to the calls for breakthrough visit): 집중 관리군에서 돌발성 방문이 요청된 횟수 대비 실제 방문 횟수 백분율 (= 집중 관리군 내 총 실제 방문 횟수 / 집중 관리군 내 돌발성 방문이 요청된 총 횟수 × 100) (%)
5) 혈압측정 기준을 만족하는 환자의 분율: 각 시험군별, 배정된 환자 중 제시된 혈압측정의 기준을 만족하는 환자의 비율 (= 혈압측정 기준을 만족하는 환자의

수 / 각 군 별 전체 환자 수) (%). 이때 혈압측정 만족 여부는 혈압측정 기준을 만족하는 횟수가 혈압이 측정된 총 횟수의 50% 이상인 경우로 정의한다.

본 연구의 이차 유효성 평가 변수들(secondary efficacy endpoints)은 다음과 같다. 아래 이차 유효성 평가변수들 중 군 간 비교 가능한 지표들에 대해서는 적절한 통계분석을 시행한다.

1) 수축기혈압의 적정범위이탈율 (OOR (out-of-range) measurement hits)

- 적정범위 이탈율 (proportion of OOR hits over the number of total measurements): 각 시험군 별, 환자 당 임상시험 기간 내 pre-specified OOR margin 을 초과하는 비율의 평균 (= 환자 별 [OOR 범위 초과 횟수 / OOR 범위 평가 횟수] 비율(%)의 전체 평균) (mean, SD). 상한, 하한 및 양측(상한 또는 하한) 이탈율을 각각 평가한다. ➔ 각 개별 환자 당 초과 비율을 계산해서 이들의 평균을 내는 것
- 가중된 적정범위 이탈율 (weighted hit score: 두번 연속에서 범위에서 벗어 나면 2 를 곱해 가중치를 부여): 각 시험군 별, 가중된 pre-specified OOR margin 초과 비율의 평균 (= 환자 별 [가중된 OOR 범위 초과 횟수 / OOR 범위 평가 횟수] 비율(%)의 전체 평균) (mean, SD). 상한, 하한 및 양측(상한 또는 하한) 이탈율을 각각 평가한다.

2) 심혈관계 사건 발생율 (vascular event)

- 뇌졸중 재발 (recurrent stroke)
- 심근경색 (myocardial infarction)
- 모든 원인으로 인한 사망 (all kind of death)

본 연구에서 평가될 안전성 평가변수들(safety endpoints)은 다음과 같다.

1) 어지러움, 추락 또는 기립성 저혈압과 관련된 사건들 (dizziness, fall, or orthostatic hypotension related events)
2) 혈압과 관련이 있을 것으로 예상되는 기타 이상반응 발생율 (other adverse events potentially related)
3) 사망률 (mortality)

8) 공변수

본 연구에서 기본적으로 수집하는 공변수는, 연령, 성별, 초기 NIHSS score, 고혈압 병력, 뇌졸중 발병 이전 혈압 강하제 투약 여부, 뇌졸중의 발생 위치 (좌/우측 및 해부학적 위치), 뇌졸중의 기전(MAGIC classification 에 의거함), 조기 신경학적 악화 발생 여부, 혈관성 위험인자(당뇨, 고지혈증, 흡연, 심방세동)이다.

7. 연구 절차

1) Screening 방문 및 Run-in period

뇌졸중 환자가 자발적 의사에 의하여 연구에 참여하기로 결정하면, 시험자는 연구 참여 동의서를 획득한다.

이 기간 동안 시험자는 연구 참여가 가능할 것으로 판단되는 뇌졸중 환자에게, 최신 임상 진료 지침 및 의사의 합리적 판단에 근거한 치료를 한다. 혈압 강하제의 시작 및 유지에 대한 결정 역시 진료 지침과 의사의 선의에 의거한 판단에 바탕으로 두어 내린다.

본 연구는 환자가 스마트폰을 사용할 수 있어야 하며 주기적으로 충전을 하는 등 적절한 관리를 할 수 있어야 한다. 또한 블루투스 모듈이 장착된 혈압계를 사용할 때, 혈압 측정자를 구분하는 절차를 준수할 수 있어야 한다. 따라서 시험자는 스크리닝 기간 중 환자의 인지 기능 및 이해력 등에 주의를 기울여야 한다.

임상시험자는 run-in period 동안 연구 대상자가 블루투스 모듈이 장착된 혈압계를 사용하고 이에 익숙해질 수 있도록 교육을 한다. 연구 대상자는 연구에 참여하면서 본인의 개인 스마트폰에 블루투스 모듈과 연계되는 어플리케이션을 설치하고, 혈압계와 안정적인 싱크가 유지되는지 확인한다. 이 과정에서 임상시험자가 연구 대상자를 도와줄 수 있다. 블루투스 모듈이 장착된 혈압계는 시험군 및 대조군 모두에게 제공된다.

혈압계는 시험 대상자가 측정하는 모든 혈압 측정치를 저장한다. 혈압계에 혈압 측정자를 구분할 수 있는 버튼 등의 기능을 부착하며, 임상시험자의 측정치만을 저장하도록 한다. 블루투스 모듈은 스마트폰이 액세스 가능 거리에 들어오면 자동으로 싱크를 하며, 이전 싱크 이후 측정되어 저장된 임상시험자의 혈압 측정치를 서버에 즉시 전송한다.

본 연구에서는 run-in period 부터 Olmesartan 이 제공된다.

이는 연구 참여가 결정된 이후, 혈압 강하제가 변경되면서 혈압의 급격한 변동이 발생하는 상황을 피하고자 함이다.

Olmesartan 은 약물 또는 그에 해당하는 약제비(보험 100%)로 연구대상자에게 지급된다.

시험자는 이 시기에 뇌졸중 환자의 연구 참여 기준 및 연구 제외 기준을 점검한다.

2) 무작위 배정 방문

시험자는 자발적으로 연구에 참여하기로 결정하고 동의를 취득한 연구대상자에 대해 6 – 3)에 규정된 절차에 따라 무작위 배정을 한다.
무작위 배정은 환자의 퇴원 시점에 이루어지며, 동의서를 취득한 스크리닝 방문으로부터 14 일 이내에 무작위 배정이 이루어져야 한다. 시험자는 임상시험과 관련된 부작용 및 이상 반응 여부를 확인하고 퇴원 시점에서 환자가 다음과 같은 교육을 모두 충실히 받고 교육 내용을 숙지할 수 있도록 해야 한다. 교육의 내용은, 1) 스마트폰에 블루투스 연동 어플리케이션을 설치하고 블루투스 싱크가 될 수 있도록 세팅, 2) 혈압의 측정 방법, 3) 임상시험 기간 중 약물 복용, 4) breakthrough call 발생 시 연락처, 5) breakthrough call 이외 환자의 요구로 방문을 원하는 경우의 연락처이다.

3) 추적 관찰 기간

i. 혈압 측정 권고

시험 대상자는 블루투스 모듈이 장착된 혈압계를 이용하여 혈압계를 측정한다. 이에 대해서는 6-5)에 규정된 절차에 의한다

ii. 집중 관리군

시험 대상자의 모든 혈압 측정치는 암호화되어 혈압계에 저장되며, 블루투스 싱크를 통하여 보안이 보장된 서버에 익명화된 형태로 저장된다.
시험 대상자는 6-4)-ii 에 정해진 약물 조절 원칙에 따른 약물을 제공받고 복용한다.
집중 관리군에 포함된 대상자는 사전에 정해진 원칙에 따라 혈압을 측정한다. 이 원칙에서 요구되는 혈압 측정 횟수의 규정 [(1) 1 주일에 5 일 이상 혈압을 측정, (2) 아침/저녁으로 혈압을 측정, (3) 1 주일에 10 회 이상 혈압 측정] 중 한 가지 이상의 조건을 충족하지 못하는 경우, 시험 대상자에게 자동화된 문자 메시지를 발송한다. 문자 메시지는 다음과 같은 내용으로 구성된다. 1) 혈압 측정 횟수 규정 중 대상자가 만족시키지 못한 조항, 2) 혈압 측정 횟수를 만족시키도록 하는 격려.
사전에 정해진 원칙에 따라 선정된 환자의 혈압 조절 목표 [6.4) 에 규정함]에서 시험 대상자의 혈압 측정치가 벗어나는 경우, 자동화된 메시지를 시험자에게 발송한다.

iii. 대조군

임상시험 대조군에 포함된 대상자는 블루투스 모듈이 장착된 혈압계를 제공 받으며, 블루투스 싱크 및 혈압 측정 방법에 대한 교육을 받는다. 시험 대상자의 모든 혈압 측정치는 암호화되어 혈압계에 저장되며, 블루투스 싱크를 통하여 보안이 보장된 서버에 익명화된 형태로 저장된다.

그러나 대조군에 포함된 대상자는 혈압 측정 횟수와 관련된 자동화된 문자 메시지를 받지 않는다. 이 대상자는 혈압 측정치가 목표를 벗어나더라도 별도의 메시지 및 구두 조언을 받지 않으며, 혈압 강하제 처방은 치료 의사의 경험에 의거하여 결정된다.

iv. Month 1 visit(visit 3)

무작위 배정 1 개월 시점 (±10 일)에 환자는 외래를 방문한다. 이 방문에서 시험자는 다음과 같은 사항을 확인한다.

1) 혈압측정
2) 혈압계 작동 및 데이터 전송 점검
3) 혈압 강하제 복용의 순응도
4) 임상시험과 관련된 결과 변수 수집
5) 임상시험과 관련된 이상 반응 여부
6) 혈압 측정치와 관련된 약물 변경

v. Month 3 visit (visit 4, 종료 방문)

무작위 배정 3 개월 (± 14 일) 시점에 환자는 외래를 방문한다. 이 방문에서 시험자는 다음과 같은 사항을 확인한다.

1) 혈압측정
2) 혈압계 작동 및 데이터 전송 점검
3) 혈압 강하제 복용의 순응도
4) 임상시험과 관련된 결과 변수 수집
5) 임상시험과 관련된 부작용 및 이상 반응 여부

이후 혈압 강하제 투약은 진료 지침과 진료 의사의 판단에 따라 결정한다.

vi. 중도 방문 (breakthrough visit)

중도 방문 시 시험자는 다음과 같은 사항을 파악하고 조치한다.

1) 혈압측정
2) 혈압계 작동 및 데이터 전송 점검
3) 혈압 강하제 복용의 순응도
4) 임상시험과 관련된 결과 변수 수집
5) 임상시험과 관련된 부작용 및 이상 반응 여부
6) 중도 방문 사유 확인 및 이에 따른 혈압 강하제 처방의 변경

vii. 개별 환자에서의 중도 중단 및 탈락

임상시험 대상자는 언제라도 본인의 요청에 따라 임상시험을 중단할 수 있으며, 안전 혹은 행정적 사유에 근거한 시험자의 판단에 의거하여 시험 대상자는 임상시험에서 탈락될 수 있다. 대상자는 동의를 철회하고 이를 가능한 방법으로 시험자에게 알림으로써 중도에서 참여를 중단할 수 있다. 시험자는 1) 연구를 지속하는 것이 대상자의 안전을 위협한다고 판단되는 경우, 2) 연구 대상자가 중도에 사망하거나 연구를 지속할 가망이 없는 상태가 된 경우, 3) 대상자가 연구 진행에 협조하지 않는 경우, 연구 대상자를 임상시험에서 탈락시킬 수 있다.

중단되거나 탈락된 연구 대상자는 대체되지 않는다. 중도 중단 혹은 탈락되는 환자에서도 시험자는 시험 대상자에서 발생한 혹은 발생할 가능성이 있는 모든 임상시험 관련 결과 변수를 수집하기 위한 노력을 기울여야 한다.

8. 병용 금지 약물 및 임상시험 계획 위반

1) 이전 약물 및 병용 약물

본 임상시험에 참여하게 되는 연구대상자들은 고혈압, 고지혈증, 당뇨 등과 같은 만성질환과 이로 인한 합병증을 갖고 치료를 요하는 상황에 처해 있을 수 있다. 따라서 연구대상자를 임상시험에 참여시키기 위한 목적으로 관련 치료를 중단하게 할 수는 없다. 그러므로 병용 금지 약물로 기술되는 약물 외에는 임상시험 기간 동안 병용투여를 할 수 있다.

따라서 연구자는 다음의 약물 군들을 반드시 임상시험 참여 전 복용여부를 확인하여야 하며, 혈압을 상승시킬 수 있는 약물을 복용하고 있는 경우에는 목표 혈압 도달 및 유지를 위해 약물 순응도 및 변경을 지속적으로 관찰하여야 한다.

- 항혈소판제제
- 당뇨약
- 콜레스테롤 저하제
- 베타차단제 - 심장보호 목적 등 혈압조절 외의 다른 임상적 목적으로 필요하거나 임상시험 전부터 사용하고 있었던 경우 사용을 허가한다.

2) 금지되는 약물

임상시험 기간 동안 기립성 저혈압 등 이상반응을 유발할 수 있는 종류의 약물의 사용은 금지되어야 한다. 따라서 임상시험에서 제공되는 혈압약을 제외한 다음 종류의 약물들은 혈압강하 목적으로 병용할 수 없다.

다만, 6.4.1 에 명시 된 3 단계 혈압강하제 조절 알고리즘 적용과 olmesartan 40mg 증량으로도 혈압이 조절되지 않는 경우 아래 종류의 약물들을 병용 할 수 있다.

- 칼슘채널차단제
- 안지오텐신 II 수용체 길항제
- 이뇨제
- 교감신경 알파차단제

3) 임상시험 계획서 위반

임상시험 계획서 위반은 연구자에 의해 중대한 또는 경미한 임상시험 계획서 위반으로 분류된다.

경미한 임상시험 계획서 위반에는 아래 기술된 중대한 임상시험 계획서 위반이 아닌 모든 위반이 포함되며, 일반적으로 시험대상자 또는 데이터 평가에 영향을 주지 않는다.

중대한 임상시험 계획서 위반에는 선정/제외 기준, 시험 수행, 시험대상자 관리, 또는 유효성 데이터 평가와 관련된 임상시험 계획서에 대한 모든 중대한 위반이 포함되며, 그 외에는 다음이 포함되나 이에 국한되지 않는다:

- 선정/제외기준 위반
- 무작위배정 위반
- 임상시험 종료방문을 하지 못한 경우
- ICF 문서에 서명하기 전에 시험 절차가 실시됨
- 시험 절차의 중대한 위반
- 시험 중 금지된 병용 약물을 복용함

9. 연구의 조기 종료

임상시험책임자는 언제라도 본 임상시험을 중지 또는 종료시킬 수 있으며, 임상시험의 중지나 조기종료 사유는 다음의 사항을 포함하지만, 반드시 이에 국한되지는 않는다.

1) 임상시험에서 이상반응 발생빈도나 중증도가 연구대상자의 건강에 위해가 될 가능성이 있는 경우.
2) 계획된 임상시험의 일정이나 연구대상자 모집이 불만족스러운 경우.
3) 시험자가 임상시험을 중단하고자 요청을 한 경우.
4) 시험자가 임상시험 수행에 있어 임상시험계획서, 임상시험과 관련된 계약서, KGCP와 같은 관련 규정이나 지침에 심각하게 위반하였거나 지속적으로 따르지 않는 경우.
5) 임상시험심사위원회가 임상시험이나 시험자에 대한 승인을 철회하거나 중지 결정을 내린 경우.

10. 이상 반응

이상반응, 시험군 적용 후 발현된 이상반응, 중대한 이상반응이 발현된 대상자 수, 발현율, 발현율에 대한 95% 신뢰구간, 발현례수를 집중 관리군과 대조군 별로 제시한다. 집중 관리군과 대조군간 발현율의 차이는 카이제곱 검정(chi-square test) 또는 피셔의 정확검정(Fisher's exact test)을 실시한다. 중증도, 인과관계, 관련조치, 결과의 각 범주에 해당하는 이상반응의 빈도, 백분율을 집중 관리군과 대조군별로 제시한다.
모든 이상반응은 MedDRA 를 사용하여 SOC(System Organ Class)와 PT(Preferred Term)으로 코딩하고, 집중 관리군과 대조군별로 코드화된 이상반응이 발현된 대상자 수, 발현률, 발현례수를 기재한다. 또한 코드화된 이상반응을 중증도, 인과관계, 관련조치, 결과에 따라 대상자수, 발현률, 발현례수를 기재한다.

11. 데이터 취급 및 품질 보증

1) 자료 관리

임상시험에서 얻어진 자료의 질적 수준을 확보하기 위한 자료관리는 KGCP 규정 및 ICH 지침에 근거한 표준작업지침서(Standard Operating Procedure, SOP)에 의해 실시한다. CRF 에 기록된 임상자료의 입력 및 관리를 위해 데이터 관리계획(Data Management Plan, 이하 DMP)을 정의하여 데이터의 완전성, 정확성 및 일치성을 점검한다. 임상시험 자료 입력의 정확성을 보장하고, 이상반응 및 병용약물은 코딩사전(Coding Dictionary)을 이용하여 코드화한다. 임상자료의 입력이 완료되면, 데이터 점검 과정(Data Validation) 및 Data Quality Control(QC) 과정을 실시하여 데이터의 완결성, 정확성 및 신뢰성을 보장할 수 있도록 하고, 이 후 데이터베이스 잠금(Database Lock)을 실시한다. 눈가림 해제(Code Open)를 실시하기 전에 Blind Meeting 을 실시하여 객관성을 유지한다.
임상시험 데이터 관리를 위해 사용된 데이터베이스(database) 및 데이터 관리 과정에서 발생되는 모든 문서들은 시스템 오류 또는 재난(system error or environmental disasters) 등으로 인한 데이터 손실을 방지하기 위해 정기적인 백업을 실시하여 복구가 가능하도록 관리한다.

2) 품질 보증

임상시험모니터요원(Clinical Research Associate)는 임상시험 중에 정기적으로 시험기관을 방문하여 근거문서와 증례기록서 대조 확인작업을 실시하고 임상시험계획서 및 관련지침에 따른 임상시험 수행 여부를 모니터링한다. 모니터링을 관련 SOP 에 따라 실행함으로써 임상시험 결과의 품질과 신뢰성을 확보한다. 최종적으로 임상시험 관련 자료의 수집·기록·문서·보고 등에 관한 제반 사항이 KGCP 및 관련규정을 준수하였음을 사전에 계획된 바에 따라 점검(audit)하여 임상시험의 신뢰성이 보증될 수 있도록 한다.

3) 자료안전성 모니터링 계획 (Data Safety Monitoring Plan)

(1) 본 모니터링 책임자: 배희준 (분당서울대학교병원 신경과)

(2) 모니터링 빈도: 각 기관에서 2 번째 대상자가 등재되었을 때 모니터링을 시행함. 3 개 기관이 참여하므로 최소 3 회 모니터링 시행 (기관별 최소 1 회)

(3) 모니터링 방법: 모니터링 책임자의 감독 하에 모니터링 요원 (분당서울대학교병원 연구간호사 중 1 인) 이 각각의 참여 기관을 방문하여 기본 문서 점검, 대상자 1 인에 대한 의무기록 대조를 시행

(4) 모니터링 시 발견된 사항에 대한 처리

A. 모니터링 책임자가 모니터링 결과 보고서를 기관 책임연구자에게 송부

B. 모니터링 결과 보고서에는 발견된 사항의 종류와 정도에 따라 각 기관 생명윤리심의위원회 보고, 재발방지 대책 마련 등을 요구할 것임

(5) 생명윤리심의위원회 보고 절차

A. 모니터링 결과 보고서는 각 기관 책임연구자가 각 기관 생명윤리심의위원회에 기타 보고로 보고하도록 함

B. 모니터링 시 발견된 사항에 대한 IRB 보고 기한

A) 이상약물/의료기기반응보고 (fatal/life-threatening): 사망이나 생명을 위협하는 사례는 7 일 이내(초기보고) + 8 일 이내(추적보고)

B) 이상약물/의료기기반응보고 (not fatal/life-threatening): 그 외의 (입원 또는 입원기간의 연장, 지속적인 또는 중대한 불구나 기능저하를 초래, 선천성 기형 또는 이상을 초래, 중요한 의학적 사건, 기타) 사례는 15 일 이내

C) 안전성 관련 정보 보고: 6 개월에 1 회

D) 예상하지 못한 문제 보고: 15 일 이내

E) 중대한 미준수 사례 보고: 15 일 이내

F) 사소한 미준수 사례 보고: 6 개월에 1 회

(6) 연구의 지속, 변경, 중단 결정을 하기 위한 주요 유효성 평가 변수에 대한 검토

A. 유효성에 대해 중간 분석을 시행하지 않음

B. 안전성에 대한 검토는 아래와 같이 시행하여 변경, 중단에 대한 결정을 함

A) 중대하고 예상하지 못한 이상반응이 발생하였을 때

(A) 책임연구자가 검토하여 연구를 변경, 중단 결정

(B) 생명윤리심의위원회 보고 결과 연구의 변경, 중단 조치를 통보 받은 경우

12. 통계 방법, 표본 수 및 검정력

1) 분석 대상군 정의

Intent-To-Treat (이하 ITT) 분석대상군은 집중관리군 또는 대조군의 적용여부에 상관 없이 모든 무작위 배정된 환자로 구성된다. ITT 원칙에 따라, 환자들은 무작위 배정된 군에 따라 분석된다. 달리 언급하지 않는 한, feasibility 및 유효성 평가변수에 대해 ITT 분석대상군을 사용하며, 이에 대한 결과로 연구결과의 최종판정을 내린다.

Per Protocol (이하 PP) 분석대상군은 ITT 분석대상군에서 중대한 시험계획서 위반을 하지 않은 모든 환자가 포함된다. 중대한 시험계획서 위반은 데이터베이스 잠금 및 임상시험의 무작위배정 해제 전에 검증 분석안 (validation analysis plan)에서 정의할 것이다. 환자들은 자신이 배정된 군에 따라 분석된다. PP 분석대상군은 feasibility 및 유효성 평가변수의 보조적 분석에 사용된다.

안전성(Safety) 분석대상군은 무작위 배정 여부에 상관 없이 실제로 집중관리군 또는 대조군을 적용 받은 모든 환자가 포함된다. 환자들은 자신이 적용 받은 군에 따라 분석된다. 안전성 분석대상군은 모든 안전성 평가변수의 분석에 사용된다.

2) 통계학적 고려 사항

i. 분석의 일반적 원칙

모든 통계분석은 SAS(Ver. 9.4, SAS Institute, Cary, NC, USA)를 사용하며, 모든 통계적 검정은 유의수준 5% 하에서 양측검정(two-sided test)을 원칙으로 한다.

ii. 중도 탈락자 (dropour) 혹은 결측치(missing data)의 처리

모든 유효성 평가변수 및 안전성 평가변수들에 대해서는 결측치 처리를 실시하지 않는다.

iii. 계획된 중간 분석

공식적인 중간 분석은 계획하고 있지 않다.

iv. 다중성 이슈 (multiplicity issues)

다수의 유효성 평가변수에 대한 분석에서 발생되는 다중성(multiple secondary analyses)에 의한 제 1 종 오류(type 1 error)의 증가에 대해 보정은 실시하지 않는다.

3) 통계 분석 방법

- 인구 통계학적 정보

인구통계학적 정보(Demographics)를 포함한 기저 특성이 연속형인 자료는 평균, 표준편차, 중앙값, 사분위수 범위, 최소값, 최대값 등을 구한다. 범주형 자료의 경우는 관찰빈도 및 백분율을 구한다.
임상시험에 등록된 모든 연구대상자에 대한 인구통계학적 정보(연령, 성별 등) 및 기저 특성에 대해서 처리군 간 차이가 없음을 검정한다. 처리군 간 비교를 위해서는 연속형 자료에 대해서는 Student's t-test 또는 윌콕슨 순위합 검정(Wilcoxon rank sum test)을 실시하고, 범주형 자료에 대해서는 카이제곱 검정(chi-square test) 또는 피셔의 정확검정(Fisher's exact test)을 실시하여 통계적 유의성을 검토한다. 이에 대한 분석대상군은 ITT 분석대상군이다.

- 활력징후(Vital Sign)

활력징후 결과에 대한 기술통계량(평균, 표준편차, 중앙값, 사분위수 범위, 최소값, 최대값)을 제시하고, 각 군 내 변화량에 대해 paired t-test 또는 윌콕슨 부호순위 검정(Wilcoxon sign rank test)를 실시하여 통계적 유의성을 살펴본다. 군 간 비교를 위해 Student's t-test 또는 윌콕슨 순위합 검정(Wilcoxon rank sum test)을 실시한다.

- Feasibility 평가 결과의 분석 (Statistical Analysis of Feasibility Endpoint)

1) 연구대상자 모집 시간(Recruitment time to prespecified number of subjects): 각 시험군별로 6.7.1절에 정의된 대로 연구대상자 모집 시간을 계산한다. 이렇게 계산된 연구대상자 모집 시간에 대해 각 시험군별 및 시험군내 병원별로 제시한다.
2) 등록된 연구대상자의 연구 참여 완료율 (retention of included participants): 각 시험군에서 연구대상자의 연구 참여 완료율을 계산하고 이 비율에 대해 카이제곱

검정(chi-square test) 또는 피셔의 정확검정(Fisher's exact test)을 실시하여 두 군 간 비교를 실시한다.

3) 돌발성 방문 요청 빈도 (frequency of calls for breakthrough visit): 돌발성 방문 요청 빈도를 계산하여 제시한다. 돌발성 방문 요청 빈도는 집중 관리군에서만 측정되는 변수이기 때문에 대조군에서의 결과는 제시되지 않는다.
4) 돌발성 방문 요청 응답률 (rate of patients who responded to the calls for breakthrough visit): 돌발성 방문 요청 응답률을 계산하여 제시한다. 돌발성 방문 요청 응답률은 집중 관리군에서만 측정되는 변수이기 때문에 대조군에서의 결과는 제시되지 않는다.

5) 혈압측정 기준을 만족하는 환자의 분율: 각 시험군에서 연구대상자의 혈압측정 기준을 만족하는 환자의 분율을 계산하고 이 율에 대해 카이제곱 검정(chi-square test) 또는 피셔의 정확검정(Fisher's exact test)을 실시하여 두 군 간 비교를 실시한다.

- 유효성 평가 결과의 분석 (Statistical Analysis of Efficacy Endpoint)

1) 수축기 혈압(SBP)의 (미리 지정된) 적정 범위 이탈율: 6.7절에 제시된 "적정범위 이탈율 (proportion of OOR hits over the number of total measurements)" 및 "가중된 적정범위 이탈율 (weighted hit score: 두번 연속에서 범위에서 벗어 나면 2를 곱해 가중치를 부여)"에 대한 기술통계량(평균, 표준편차, 중앙값, 사분위수범위, 최소값, 최대값)을 계산하고, Student's t-test 또는 윌콕슨 순위합 검정(Wilcoxon rank sum test)을 실시하여 두 군 간 비교를 실시한다.
2) 심혈관계 사건 발생율 (vascular event): 각 시험군에서 심혈관계 사건이 발생한 연구대상자의 비율을 계산하고, 이 비율에 대해 카이제곱 검정(chi-square test) 또는 피셔의 정확검정(Fisher's exact test)을 실시하여 두 군 간 비교를 실시한다.

- 이상반응 평가 결과의 분석 (Statistical Analysis of Safety Endpoint)

1) 어지러움, 낙상 또는 기립성 저혈압과 관련된 사건들 (dizziness, fall, or orthostatic hypotension related events): 각 시험군에서 어지러움, 추락 또는 저혈압과 관련된 사건이 발생한 연구대상자의 비율을 계산하고, 이 비율에 대해 카이제곱 검정(chi-

square test) 또는 피셔의 정확검정(Fisher's exact test)을 실시하여 두 군 간 비교를 실시한다.

2) 혈압과 관련이 있을 것으로 예상되는 기타 이상반응 발생율 (other adverse events potentially related): 각 시험군에서 혈압과 관련이 있을 것으로 예상되는 기타 이상반응이 발생한 연구대상자의 비율을 계산하고, 이 비율에 대해 카이제곱 검정(chi-square test) 또는 피셔의 정확검정(Fisher's exact test)을 실시하여 두 군 간 비교를 실시한다.
3) 사망률 (mortality): 각 시험군에서 사망이 발생한 연구대상자의 비율을 계산하고, 연구대상자의 비율에 대해 카이제곱 검정(chi-square test) 또는 피셔의 정확검정(Fisher's exact test)을 실시하여 처리군 간 비교를 실시한다.

- 기타 안전성 자료(Other Safety Data)

기타 안전성 자료는 연속형 자료에 대해서는 기술통계량(평균, 표준편차, 중앙값, 사분위수 범위, 최소값, 최대값)을 제시하고, 각 군 내 변화량에 대해 paired t-test 또는 윌콕슨 부호순위 검정(Wilcoxon sign rank test)을 실시하여 각 군 내 통계적인 유의성을 살펴본다. 범주형 자료에 대해서는 연구대상자의 빈도 및 분율 등 기술통계량을 계산한다. 두 군 간 비교를 위해 연속형 자료에 대해서는 Student's t-test 또는 윌콕슨 순위합 검정(Wilcoxon rank sum test)을 실시하고, 범주형 자료에 대해서는 카이제곱 검정(chi-square test) 또는 피셔의 정확 검정(Fisher's exact test)을 사용하여 유의성을 검토한다.

- 표본 수 결정

본 임상시험을 위해 선정된 표본 수는 타당성에 근거한다. 이는 본 임상시험이 혈압 관리 전략에 대한 실현 가능성을 확인하는 임상시험(feasibility trial)이기 때문에 통계적인 고려사항에는 근거하지 않는다. 계획된 수의 연구대상자는 집중 관리군 30 명, 대조군 30 명으로, 전체 연구대상자수는 60 명이 될 것이다.

13. 연구 윤리

1) 임상시험관리기준 준수

본 임상시험은 임상시험계획서를 준수하고 임상시험관리기준(KGCP) 및 헬싱키 선언(Declaration of Helsinki)에 있는 윤리적 원칙과 기타 관련 규정을 준수하여 수행될 것이다.

2) 연구대상자 동의

각 참여기관의 연구책임자는 연구대상자에게 임상시험의 설명과 질의 응답에 대해서 가급적 대상자가 충분히 이해를 할 수 있는 시간을 배려하여 설명하고 동의 취득을 해야 한다. 또한 임상시험에서 사용할 설명문 및 동의서 양식은 각 기관의 IRB 에서 승인된 동의서를 가지고 임상시험을 진행하여야 한다. 임상시험 계획서에 명시된 연구 절차의 시작 및 등록 전 각 연구대상자로부터 동의서를 받아야 한다.
동의서는 대상자에게 친밀한 환경에서 연구 대상자가 자유롭게 의사 소통이 가능한 언어로 진행될 것이다. 환자가 연구 참여 결정에 있어 어떠한 강제적 압력을 받지 않도록, 연구자는 최선을 다 할 것이다. 연구 과정 및 참여 절차에 대해 설명하면서, 언제든 연구 대상자의 의사에 의거하여 연구 참여를 철회할 수 있으며 그러한 결정이 환자의 이후 진료 과정에 영향을 미치지 않음을 분명히 알릴 것이다. 연구에 참여하기로 결정한 대상자에게는 연구 동의서 사본을 제공할 것이며, 연구 대상자가 요청하는 경우 연구 참여 결정 이전에도 동의서 사본을 제공하여 대상자의 가족 및 지인과 상의도록 협조할 것이다.
각 기관의 연구책임자는 동의서의 원본을 잠금 장치가 되어 있는 별도의 장소에 보관하고 대상자에게는 사본을 제공하여야 한다. 또한 동의 취득과정에 대해서는 반드시 근거문서에 기록을 남겨야 한다. 연구대상자가 무학 또는/그리고 문맹으로 인해 동의서를 취득할 수 없는 경우에는 법정 대리인으로부터 동의서를 취득한다.
만약 임상시험계획서에 변경이 생겨 변경된 정보를 제공해야 할 경우, 각 기관의 연구책임자는 변경된 동의서의 정보를 대상자에게 제공해야 하며 서명된 동의서는 원본을 보관하고 연구대상자에게는 사본을 제공하여야 한다.

3) 임상시험 모니터링

대상자의 권리와 복지 보호, 보고된 임상시험 관련 자료가 근거문서와 대조하여 정확하고, 완전하며, 검증이 가능한지 여부 확인, 임상시험이 승인된 계획서, 의약품 임상시험관리기준 및 관련 규정에 따라 수행되는지의 여부 확인을 위하여 모니터링을 실시한다.
임상시험에 대한 모니터링은 연구책임자(PI)가 지정한 모니터 요원의 정기적인 시험기관방문과 전자 증례기록지(eCRF)를 통해서 이루어 질 것이다. 방문 시 모니터 요원은 기본적으로 대상자 기록 원본, 약물 관리 기록, 자료(연구 파일) 보관 내용 등을 확인한다.
또한 모니터 요원은 임상시험 진행과정을 잘 살피고, 문제가 있을 경우 연구책임자(PI)와 상의한다. 참여기관 방문의 적절한 시간은 각 기관의 연구책임자와 모니터 요원이 협의하여 배분하여야 한다. 각 기관의 연구책임자는 또한 의약품 임상시험관리기준에 정의된 것과 같이, 모니터가 eCRF 에 기입된 자료들을 확인할 수 있는 대상자의 원 자료들(source documents: 병원 또는 개인 차트, 실험실 결과 기록 등)을 볼 수 있도록 제공 하여야 한다.

4) 근거문서에 대한 직접 열람

본 임상시험에서 근거문서는 연구대상자의 자료를 담고 있는 모든 자료를 근거문서로서 정한다. 따라서 연구대상자의 의무기록지, 결과지 및 전산 상의 자료도 근거문서로서 인정한다. 그러나 근거문서는 ICH-GCP 에서 정하고 있는 ALCOA 원칙에 따라 누구에 의해 기록된 것인지가 명확하고 언제라도 명확히 읽을 수 있어야 하며 임상시험의 진행이 이루어 진 동시에 기록된 최초의 자료를 근거문서로서 인정한다. 그 자료는 명확하고 정확하여야 한다.
연구대상자가 동의서 및 설명문에 포함된 정보를 통해 임상시험에 참여하는 서명을 한 이후에는 연구대상자의 자료를 본 임상시험 참여 연구진에게 열람을 허용하는 것으로 인정되며, 각 기관의 연구책임자는 의뢰자측의 모니터에게 직접 열람을 할 수 있도록 적절한 장소 및 절차에 대해 협조하여야 한다.

5) 기밀 유지

대상자의 신원을 파악할 수 있는 기록은 비밀로 보장될 것이며, 임상시험의 결과가 출판될 경우에도 대상자의 신원을 비밀상태로 유지한다. eCRF 등 임상시험에 관련된

모든 서류에는 환자 이름이 아닌 대상자식별코드 (일반적으로 환자의 이니셜)로 기록하고 구분한다.

14. 참고 문헌

1. Meschia JF, Bushnell C, Boden-Albala B, Braun LT, Bravata DM, Chaturvedi S, et al. Guidelines for the primary prevention of stroke: A statement for healthcare professionals from the american heart association/american stroke association. *Stroke*. 2014;45:3754-3832
2. Kannel WB, Schwartz MJ, McNamara PM. Blood pressure and risk of coronary heart disease: The framingham study. 1969. *Chest*. 2009;136:e23
3. Kannel WB, Wolf PA, Verter J, McNamara PM. Epidemiologic assessment of the role of blood pressure in stroke: The framingham study. 1970. *JAMA*. 1996;276:1269-1278
4. Kernan WN, Ovbiagele B, Black HR, Bravata DM, Chimowitz MI, Ezekowitz MD, et al. Guidelines for the prevention of stroke in patients with stroke and transient ischemic attack: A guideline for healthcare professionals from the american heart association/american stroke association. *Stroke*. 2014;45:2160-2236
5. Leonardi-Bee J, Bath PM, Phillips SJ, Sandercock PA, Group ISTC. Blood pressure and clinical outcomes in the international stroke trial. *Stroke*. 2002;33:1315-1320
6. James PA, Oparil S, Carter BL, Cushman WC, Dennison-Himmelfarb C, Handler J, et al. 2014 evidence-based guideline for the management of high blood pressure in adults: Report from the panel members appointed to the eighth joint national committee (jnc 8). *JAMA*. 2014;311:507-520
7. Oparil S, Schmieder RE. New approaches in the treatment of hypertension. *Circ Res*. 2015;116:1074-1095
8. Group SR, Wright JT, Jr., Williamson JD, Whelton PK, Snyder JK, Sink KM, et al. A randomized trial of intensive versus standard blood-pressure control. *N Engl J Med*. 2015;373:2103-2116
9. Friedberg JP, Rodriguez MA, Watsula ME, Lin I, Wylie-Rosett J, Allegrante JP, et al. Effectiveness of a tailored behavioral intervention to improve hypertension control: Primary outcomes of a randomized controlled trial. *Hypertension*. 2015;65:440-446
10. Egan BM, Zhao Y, Axon RN. Us trends in prevalence, awareness, treatment, and control of hypertension, 1988-2008. *JAMA*. 2010;303:2043-2050
11. Haller H, Ito S, Izzo JL, Jr., Januszewicz A, Katayama S, Menne J, et al. Olmesartan for the delay or prevention of microalbuminuria in type 2 diabetes. *N Engl J Med*. 2011;364:907-917
12. Hirohata A, Yamamoto K, Miyoshi T, Hatanaka K, Hirohata S, Yamawaki H, et al. Impact of olmesartan on progression of coronary atherosclerosis a serial volumetric intravascular ultrasound analysis from the olivus (impact of olmesarten on progression of coronary atherosclerosis: Evaluation by intravascular ultrasound) trial. *J Am Coll Cardiol*. 2010;55:976-982
13. de la Sierra A, Volpe M. Olmesartan-based therapies: An effective way to improve blood pressure control and cardiovascular protection. *J Hypertens*. 2013;31 Suppl 1:S13-17
14. Ogawa H, Kim-Mitsuyama S, Matsui K, Jinnouchi T, Jinnouchi H, Arakawa K, et al. Angiotensin ii receptor blocker-based therapy in japanese elderly, high-risk, hypertensive patients. *Am J Med*. 2012;125:981-990

15. Niiranen TJ, Jula AM, Kantola IM, Reunanen A. Comparison of agreement between clinic and home-measured blood pressure in the finnish population: The finn-home study. *J Hypertens*. 2006;24:1549-1555
16. Bonafini S, Fava C. Home blood pressure measurements: Advantages and disadvantages compared to office and ambulatory monitoring. *Blood Press*. 2015;24:325-332
17. Krakoff LR. Blood pressure out of the office: Its time has finally come. *Am J Hypertens*. 2016;29:289-295
18. Webb AJ, Wilson M, Lovett N, Paul N, Fischer U, Rothwell PM. Response of day-to-day home blood pressure variability by antihypertensive drug class after transient ischemic attack or nondisabling stroke. *Stroke*. 2014;45:2967-2973
19. Ogedegbe GO, Boutin-Foster C, Wells MT, Allegrante JP, Isen AM, Jobe JB, et al. A randomized controlled trial of positive-affect intervention and medication adherence in hypertensive african americans. *Arch Intern Med*. 2012;172:322-326
23. Sanford M, Keam SJ. Olmesartan medoxomil/amlodipine. Drugs. 2009;69(6):717-29
24. Neutel JM, Smith DH, Weber MA, Wang AC, Masonson HN. Use of an olmesartan medoxomil-based treatment algorithm for hypertension control. J Clin Hypertens (Greenwich). 2004 Apr;6(4):168-74.

15. 동의서 및 환자 설명서

연구 대상자 설명문

연구 제목: 급성 허혈 뇌졸중에서 혈압 관리의 최적화 전략에 관한 제 2 상 무작위 대조군, 맹검 결과 평가 및 실현 가능성 평가 임상 시험 [BOSS-Trial I]

귀하께 본 임상 연구에 참여하여 주실 것을 요청 드립니다. 본 연구에 참여하실 것을 결정하기 전에 본 연구의 수행 목적과 절차에 대하여 귀하께서 정확하게 이해하시는 것은 매우 중요합니다. 아래의 글을 통하여 귀하께 본 연구의 수행 목적과 절차를 안내하여 드리고, 본 연구 참여를 통하여 경험하실 잠재적인 이득과 위험 요소를 말씀드릴 것입니다. 충분한 시간을 가지고 본 연구 대상자 설명문을 읽으실 것을 권해 드립니다. 귀하의 가족 혹은 관련된 분과 상의하시는 것도 괜찮습니다. 또한 궁금하신 사항이 있는 경우, 본 연구의 담당자 및 귀하를 진료하는 의사에게 문의하셔도 됩니다. 본 임상 연구 참여는 전적으로 귀하의 자발적인 의사에 의하여 이루어지며, 본 연구에 참여하지 않으신다고 하더라도 귀하께서는 어떠한 불이익을 받지 않을 것입니다. 또한 귀하의 연구 참여 의사는 언제든지 귀하의 자발적인 결정에 의하여 철회될 수 있습니다.

1. 본 임상 연구의 목적

귀하께서는 급성 허혈성 뇌졸중(뇌경색)으로 진단을 받으셨으며, 뇌졸중 발생 이후 초기에 혈압이 높아서 본 연구 참여 대상자로 선정 되셨습니다.
고혈압은 뇌경색의 중요한 원인이며 또한 뇌경색의 재발을 예방하기 위하여 조절할 수 있는 중요한 임상적 지표입니다. 따라서 뇌경색 이후에도 혈압을 잘 조절하는 것은 매우 중요합니다. 그렇지만 아직까지 혈압 관리의 실제적인 원칙과 요소에 대해서는 알려져 있지 않은 부분이 많습니다. 최근 IT 기술의 발달과 함께, 집에서 측정한 혈압을 스마트폰을 통해 바로 서버에 전송하여 저장하는 시스템을 구축할 수 있게 되었습니다.
그러한 맥락에서 본 연구는, 뇌경색 환자가 집에서 측정한 혈압을 블루투스 내장 혈압계를 통해 서버에 저장하고, 그 혈압 데이터에 기반하여 이후 혈압 약물을 조절하는 방식의 임상적 유효성을 탐색하고자 합니다.

2. 본 임상 연구는 연구 목적으로 수행됩니다.

본 임상 연구에서 주로 연구하고자 하는 것은, 블루투스 혈압계를 이용한 혈압 수집 및 그러한 혈압 관리 전략의 유효성입니다. 이러한 전략이 뇌경색 환자에서 재발을 예방할 수 있는 확고한 근거는 아직 충분하지 않으며, 따라서 본 연구 및 관련 활동은 연구 목적으로 수행됩니다.

3. 본 임상 연구는 개발 중인 약물을 투여하는 것을 목적으로 하지 않음.

본 임상 연구는 환자에게 개발 중인 약물을 투여하는 것을 연구 대상 및 목적으로 하지 않습니다. 본 임상 연구 과정에서 환자의 혈압 관리를 위하여 투여되는 혈압약은 그 효능 및 안전성이 과학적으로 확립되어 시중에서 판매되는 것입니다.

4. 향후 연구의 절차

본 연구에 참여 의사를 밝히시게 되면, 우선 연구 담당자가 귀하를 방문하여 블루투스 혈압계를 사용하실 수 있는지 확인할 것입니다. 이후 연구 담당자에게 귀하의 스마트폰에 블루투스 혈압계를 사용할 수 있도록 세팅을 할 것이며, 혈압계 사용 및 혈압 측정에 대한 교육을 할 것입니다. 귀하는 퇴원 이후 혈압 측정에 대하여 교육을 받은 대로 혈압계를 사용하십시오.
본 연구는 블루투스 혈압계를 이용한 혈압 수집, 행동 자극 및 사전에 규정된 혈압 강하제 조절 알고리듬이, 연구 대상자의 혈압 조절에 미치는 영향을 수집하고자 하는 목적을 갖고 있습니다. 본 연구 참여자는 적극적으로 행동 자극을 받고 사전에 규정된 혈압 강하제 조절 알고리듬에 따라 혈압 강하제를 투여 받는 집단 혹은 통상적인 혈압 관리를 받는 집단, 두 가지로 분류될 것입니다. 두 집단에 포함되는 대상자의 숫자는 동일합니다. 이러한 분류는 무작위적으로 이루어지며 귀하를 진료하는 의사는 그 분류 과정에 개입하지 않습니다.
귀하의 본 임상 연구 참여는 3 개월 간 지속됩니다. 연구 참여 기간 동안 귀하는 아래의 규칙에 따라 혈압을 측정하도록 권고받게 될 것입니다.

- 아침- 기상후 15 분, 식사하지 않고 약 10 분 휴식 후
- 저녁- 저녁 식사후 2 시간, 약 10 분 휴식 후
- 측정시 2 번 이상 측정
- 적어도 주 5 일 이상 (10 번/주 이상)

귀하의 임상 연구 참여가 종료되는 시점에 귀하는 외래를 다시 방문하게 됩니다.

5. 연구 대상자가 준수하여야 할 사항

귀하께서 본 연구에 참여하시는 경우, 귀하는 연구 담당자에게 교육을 받은 대로 블루투스 혈압계를 이용하여 혈압을 측정하셔야 합니다. 블루투스 혈압계를 이용하여 귀하가 아닌 다른 사람이 혈압을 측정하지 않아야 합니다.
퇴원 이후 연구 담당자가 귀하에게 연락을 할 수 있습니다. 만약 연구 담당자가 귀하에게 중도 외래 방문을 요청하는 경우, 귀하는 외래를 방문하셔야 합니다.
연구 담당자가 귀하에게 연구 진행, 혈압 측정 및 혈압 수준과 관련된 접촉을 하기 위하여, 본 연구진은 암호화된 형태로 귀하의 전화번호를 보관할 것입니다. 본 전화번호는 연구 진행 및 귀하의 안전을 위한 목적으로만 사용될 것이며, 그 외의 어떠한 목적으로도 이용되지 않을 것입니다. 귀하의 전화번호는 귀하의 연구 참여 기간이 종료되는 즉시 영구적으로 삭제될 것입니다.
귀하의 건강에 변화가 있거나 본 임상 연구에 대하여 어떠한 우려가 있다면 즉시 연구 담당자에게 알려야 합니다.

6. 본 연구 참여를 통하여 연구 대상자가 얻을 수 있는 이익

귀하께서 본 연구에 참여하시는 동안, 귀하는 블루투스 혈압계를 자유롭게 이용하여 혈압을 측정할 수 있습니다. 귀하가 블루투스 혈압계를 이용하여 측정한 혈압은 본 연구팀의 서버에 저장되어 귀하의 혈압 조절을 위하여 사용될 것입니다.
본 연구팀은 연구 참여의 대가로 금전적 이득을 제공하지 않습니다.

7. 본 임상 연구에 참여함으로써 연구 대상자가 치루어야 하는 비용 혹은 위험

본 연구에서 사용되는 블루투스 혈압계는 이미 관련 기관의 검증을 받아 시중에서 자유롭게 구입할 수 있는 것입니다. 또한 혈압 관리를 위하여 투여하는 약물 역시 관련 기관에서 시판을 허가 받은 것입니다. 따라서 귀하께서 본 임상 연구에 참여하신다고 하여 새로운 위험을 감수하시게 되는 상황은 발생하지 않을 것으로 생각됩니다.
연구 참여가 결정된 이후, 귀하는 혈압의 급격한 변동을 예방하기 위해 연구진이 제공하는 혈압 강하제인 Olmesartan 을 복용하게 될 것이며, 방문 2(퇴원시)에는 Olmesartan 한달 분과 방문 4(3 개월 방문시)에는 그에 해당하는 약제비(보험 100

%)를 아래와 같이 제공받을 것입니다. 이 비용은 연구자가 부담하며, 귀하께서는 지불하실 필요가 없습니다. 혈압약은 올메사르탄, 암로디핀 혹은 다이아클로지드의 조합으로 구성됩니다.

방문 2(퇴원시, 한달 분): 혈압강하제(Olmesartan)
방문 4(3 개월 방문시, 두달 분): 70,000 원

귀하께서 3 개월의 본 임상 연구 참여 기간을 마치고 중도에 연구 참여 의사를 철회하지 않으시는 경우, 본 연구팀은 귀하께 그 동안 사용하신 블루투스 혈압계를 기증할 것입니다.

8. 연구 대상자가 선택할 수 있는 다른 치료 방법이나 종류 및 그 치료 방법의 잠재적 위험과 이익

본 임상 연구는, 블루투스를 이용한 혈압 수집 및 그러한 혈압 관리 전략의 유효성을 검증하고자 합니다. 따라서 귀하께서 본 임상 연구에 참여하지 않으시는 경우, 담당 의료진은 통상적인 절차 (1-3 개월 간격의 외래 방문 및 외래 방문 시점에 측정한 혈압에 근거한 혈압약 투여)에 따라 귀하를 치료할 것입니다.

9. 임상 연구와 관련된 손상이 발생할 경우 연구 대상자에게 주어질 보상이나 치료 방법

혈압계 사용과 관련한 상완부 압박감 및 멍 등이 발생할 수 있으나, 이는 본 연구와 관련 없이 혈압계의 측정 방식에 의한 것입니다.
그 외에 임상 연구 참여 기간 중 의료진은 귀하의 안전을 지키려고 최선을 다해 노력할 것이고 중대한 이상 반응 발생시는 빠르고 적절한 조치를 취하여 가능한 한 그 이상반응을 최소화 할 것입니다.
이 임상 연구에 참여하면서 블루투스 혈압계의 사용 혹은 혈압 강하제의 투여 관련된 피해가 발생할 경우에는 책임 연구자가 법적인 책임을 지고 피해보상에 관한 규약에 의거하여 피해 보상할 것이며, 이상 반응 및 질환의 악화의 경우에는 가능한 한 최선의 치료 방법으로 치료할 것입니다.

10. 연구 대상자는 본 임상 연구에 자발적으로 참여하며, 연구 참여에 대하여 어떠한 강제도 없을 것이라는 점. 또한 자발적으로 연구에 참여하였더라도, 이후 언제든지 연구 대상자의 의사 표명에 의하여 연구 참여를 중단할 수 있다는 점.

본 임상 연구는 철저하게 귀하의 자발적인 참여 의사를 존중합니다. 본 연구진 및 담당 의료진은 귀하에게 연구 참여를 강제하지 않을 것입니다. 귀하께서 연구에 참여하지 않는다고 하여, 담당 의료진이 귀하에게 불이익을 입히거나 이후 진료 과정을 소홀히 하는 일은 발생하지 않을 것입니다.
귀하께서는 언제든지 중도에 연구 참여 의사를 철회하실 수 있습니다. 귀하께서 중도에 연구 참여 의사를 철회하신다고 하여, 귀하께서 불이익을 당하는 일은 없을 것입니다.

11. 연구 대상자의 개인 정보는 철저하게 비밀리에 관리될 것이며, 향후 본 연구의 결과가 출판되더라도 연구 대상자의 신원이 노출되지 않을 것이라는 점. 모니터 요원 혹은 점검자가 연구 대상자의 의무 기록을 열람할 수 있으며, 연구 대상자 혹은 대리인의 동의서 서명이 이러한 자료 열람을 허용한다는 사실.

본 임상 연구의 수행 과정에서 귀하의 개인 정보를 식별할 수 있는 정보는 기록되지 않습니다. 귀하에게는 임의의 관리 번호가 부여될 것이며, 귀하의 성함은 영문 이니셜로 변경되어 기록될 것입니다. 연구 진행 중에 연구의 윤리적 수행을 검증하기 위하여 감시 요원이 활동할 것입니다. 본 감시 요원은 암호화된 파일을 열람하여 귀하의 병록 번호를 확인하고 귀하의 의무 기록을 열람하여, 연구의 올바른 수행 및 임상 연구에서 수집된 자료의 품질을 검증할 것입니다.
임상시험 진행 중 및 종료 후에도 임상시험의 모니터요원, 점검을 실시하는 자, 심사위원회 및 식품의약품안전처장, 보건복지부장관 등은 귀하의 비밀보장을 침해하지 않고 관련규정이 정하는 범위 안에서 임상시험 실시 절차와 자료의 신뢰성을 검증하기 위해 귀하의 의무기록을 포함한 연구 관련 자료를 직접 열람할 수 있으며, 대상자 서명 동의서에 서명하심으로써 귀하 또는 귀하의 대리인께서는 이러한 자료의 직접열람을 허용함을 의미 합니다.
이후 연구의 결과를 공개적으로 출판하는 경우에도, 귀하의 개인적인 정보 및 개인을 식별할 수 있는 정보는 절대로 출판물에 포함되지 않을 것입니다.

12. 연구 대상자의 임상 시험 지속 여부에 영향을 줄 수 있는 새로운 정보가 알려지는 경우, 이 사실을 즉시 연구 대상자에게 알린다는 점.

본 임상 연구의 수행 과정에서 새로운 연구 결과가 발표되어 본 임상 연구를 지속하는 것이 필요하지 않거나 윤리적으로 그릇된 상황이 발생하는 경우, 본 연구진은 이 사실을 즉시 귀하에게 알리고 이후 연구 참여 지속 여부에 대해 논의할 것입니다.

13. 본 임상 연구 및 연구 대상자의 권리와 이익에 관하여 추가적인 정보를 얻고자 하거나 임상 연구와 관련하여 문의 및 상의하고자 하는 경우의 연락처

언제라도 귀하 혹은 귀하의 대리인이 전화 면담을 요청할 수 있으며, 연락처는 다음과 같습니다.
연구 책임자: 배희준 교수
임상 시험에서 발생한 문제, 우려, 질문에 대하여 상의할 연구 담당자의 연락처:010-3806-7975

연구 대상자의 권익에 대한 문제, 우려, 질문이 있을 때 상의할 생명윤리심의위원회 또는 임상연구윤리센터 연락처: 031-787-1376

14. 연구 도중 연구 대상자의 임상 연구 참여가 중지되는 경우 및 그 사유

귀하께서는 언제라도 귀하의 요청에 따라 본 연구 참여를 중단하실 수 있습니다.
또한 안전 혹은 행정적 사유에 근거한 연구 책임자의 결정에 따라 귀하의 연구 참여가 중단될 수 있습니다. 연구 책임자는 1) 연구를 지속하는 것이 귀하의 안전을 위협한다고 판단되는 경우, 2) 귀하의 건강 상황이 연구를 지속하기 어렵다고 판단되는 경우, 3) 귀하의 제반 상황이 연구를 지속하기 어렵다고 판단되는 경우, 귀하께 연구 참여 중단을 요청할 수 있습니다.

15. 연구 대상자의 임상 연구 예상 참여 기간

본 임상 연구는 약 3 개월간 지속됩니다.

16. 임상 연구에 참여하는 대략의 대상자 수

본 임상 연구는 국내에서 여러 병원이 참여하며, 총 60 여 명의 대상자가 모일 것으로 예상됩니다.

연구 대상자 동의서

연구의 제목 : 급성 허혈 뇌졸중에서 혈압 관리의 최적화 전략에 관한 제 2 상 무작위 대조군, 맹검 결과 평가 및 실현 가능성 평가 임상 시험 [BOSS-Trial I]

1. 나는 본 연구에 대한 모든 정보들에 관하여 담당 의사 혹은 연구 담당자로부터 자세하게 설명을 듣고 충분히 이해하였습니다.
2. 나는 또한 연구 대상자 설명문을 읽어 보았으며, 그 내용을 충분히 이해하였으며 본 연구가 연구 목적으로 수행된다는 사실을 알고 있습니다.
3. 나의 연구 참여여부 결정은 자발적인 것이며, 연구 기간 중 언제라도 개인적인 사유 등으로 지속적인 참여를 중도에 거부하거나 자유로이 참가를 중단할 수 있으며, 이로 인해 진료 및 기타 어떠한 불이익도 받지 않음을 알고 있습니다.
4. 나는 본 연구 과정에서 모종의 피해가 있을 경우, 피해보상에 관한 규약에 의거하여 '연구자'가 부담한다는 사실을 알고 있습니다.
5. 나는 연구 관련하여 의문이 있을 경우에는 언제라도 시험자에게 문의할 수 있으며, 나의 의무기록을 연구 목적으로만 직접 열람하는 데에 동의합니다.

이에 나의 자유로운 의사에 따라 본 연구에 참여할 것을 동의합니다.

	성 명	서 명	서 명 날 짜
**연구대상자**			년 월 일
**시험책임자 (또는 공동연구자)**			년 월 일

16. CRF 및 이상 반응 보고 서식

증례기록서

CASE REPORT FORM

급성 허혈성 뇌졸중에서 혈압관리의 최적화 전략에 관한 제 2 상 전향적, 무작위 대조군, 공개, 실현가능성 평가 임상시험 [BOSS – Trial I]

Optimization of blood pressure management after acute ischemic stroke and its prognostic significance: Prospective, randomized, open, feasibility Trial [BOSS – Trial I]

**CRF Version**	**1.3**
**임상시험기관**	
**피험자 이니셜(영문)**	__ __ __
**피험자 번호(Subject No.)**	__ __ __ __

Baseline

BOSS-Trial I<br>CRF Version 1.3<br>on 2017-01-20	**PATIENT INITIALS**<br>└──┴──┴──┘	**SUBJECT No.**<br>└──┴──┴──┴──┘	**BASELINE**

성별 : 남 / 여<br><br>나이:	내원시각 년 월 일 시 분<br>발병시각 Clear onset : 년 월 일 시 분<br>Unclear onset : LNT 년 월 일 시 분<br>FAT 년 월 일 시 분
History of Risk Factor<br><br>_ Pre stroke Hx _HTN _ DM _ Hyperlipidemia _ smoking<br>_ Af _ Coronary heart disease	키:<br>몸무게:<br>Abd circumference:<br><br>Initial NIHSS(total) :<br>Previous mRS :
**내원 경로** _ OPD _ ER _ in-hospital ☐ Transfer-in	
**NR expertise time 년 월 일 시 분**	

TOAST

_ LAA _ SVO _ CE _ Other-determined _ Undetermined_ 2 or more _ Negative _ Incomplete

Previous TIA Hx ☐ No ☐ Yes
Previous Stroke Hx ☐ No ☐ Yes
Type _ Hemorrhagic _ Ischemic _ Mixed _ Unknown
Family Hx of Premature CHD ☐ No ☐ Yes
Peripheral Arterial Disease ☐ No ☐ Yes
Symp. carotid artery Disease ☐ No ☐ Yes

Coronary Heart Disease (다중선택가능)
☐ Hx of AMI
☐ Evidence of silent MI or MI
☐ Hx of angina
☐ Hx of coronary angioplasty or surgery

BOSS-Trial I<br>CRF Version 1.3<br>on 2017-01-20	**PATIENT INITIALS**<br>|__|__|__|	**SUBJECT No.**<br>|__|__|__|__|	**BASELINE**

Hypertension

유무 ☐ No ☐ Yes

조절상태 ☐ Never advised of high BP(Dx at Adm ☐ No ☐ Yes) ☐ HTN but never anti-HTN

☐ Ever Used anti-HTN

○ Ceased using anti-HTN

○ Currently using anti-HTN(☐ with regular Tx ☐ without regular Tx)

DM

유무 ☐ No ☐ Yes

조절상태 ☐ Never advised of high BST (Dx at Adm ☐ No ☐ Yes)

☐ DM but never anti-DM

☐ Ever Used anti-DM

○ Ceased using anti-DM

○ Currently using anti-DM(☐ with regular Tx ☐ without regular Tx)

Dyslipidemia

유무 ☐ No ☐ Yes

조절상태 ☐ Never advised of high lipid(Dx at Adm ☐ No ☐ Yes)

☐ HL but never anti- lipid

☐ Ever Used anti- lipid

○ Ceased using anti-HL

○ Currently using anti-HL(☐ with regular Tx ☐ without regular Tx)

Smoking ☐ No ☐ Yes [☐Current smoker (PY;) ☐ EX- (<5y) ☐ Ex-(≥5y)]

AF ☐ No ☐ Yes

☐ Dx at Adm ☐ Hx of Af (☐ with regular Tx ☐ without regular Tx)

Potential Sources of CE

High-risk

_ Mechanical prosthetic valve
_ Mitral stenosis with atrial fibrillation
_ Atrial fibrillation (other than lone AF)
_ Left atrial/atrial appendage thrombus
_ Sick sinus syndrome
_ Recent MI (<4 week)
_ Left ventricular thrombus
_ Dilated cardiomyopathy
_ Akinetic left ventricular segment
_ Atrial myxoma
_ Infective endocarditis

Medium-risk

_ Mitral valve prolapse
_ Mitral annulus calcification
_ Mitral stenosis without atrial fibrillation
_ Left atrial turbulence(smoke)
_ Atrial septal aneurysm
_ Patent foramen ovale
_ Atrial flutter
_ Lone atrial fibrillation
_ Bioprosthetic cardiac valve
_ Nonbacterial thrombotic endocarditis
_ Congestive heart failure
_ Hypokinetic left ventricular segment
_ MI (>4weeks, <6months)

Antiplatelets ☐ No ☐ Yes ☐ With 7days

(_ Aspirin _ Clopidogre l _ Aspirin + Dipyridamole _ Cilostazol _ Triflusal _ Ticlopidine _other _______)

Anticoagulation ☐ No ☐ Yes

Anti-hypertension ☐ No ☐ Yes

(_diuretics _Ca blocker _ß blocker _ ACE _ARB _α blocker _unknown)

Anti-hyperlipidemia **statin** ☐ No ☐ Yes **other** ☐ No ☐ Yes

Anti-DM ☐ No ☐ Yes

급성기 치료 (48 시간 이내)

Thrombolytic	Route	_ IV	_ IA	_ IV+IA

Antiplatelet _ Aspirin _ Clopidogre l _ Aspirin + Dipyridamole _ Cilostazol _ Triflusal _ Ticlopidine)

Anticoagulation
_ Heparin _ Warfarin _ Apixaban _Dabigatran _Rivaroxabn _ LMWH
_ Thrombin inhibitor _ others
_ others ( )

other treatments ___________________________

Visit 1

BOSS-Trial I<br>CRF Version 1.3<br>on 2017-01-20	PATIENT INITIALS |__|__|__|	SUBJECT No. |__|__|__|__|	VISIT 1<br>(SCREENING)

방문일	|__|__|__|__|__|__| 년 YY 월 MM 일 DD

선정기준	Yes	No
1. 발병 일주일 내에 입원하여 뇌영상에서 급성 뇌경색이 확인되었습니까?	☐	☐
2. 만 19 세 이상의 남성 또는 여성 입니까?	☐	☐
3. 환자의 상태가 안정되어 본 임상시험에서 규정된 혈압약의 투여가 가능합니까?	☐	☐
4. 뇌졸중 증상을 처음으로 인지한 시점에서 최소한 24 시간이 경과한 이후, 연구대상으로 등록되기 전 이틀 동안 측정한 평균 수축기 혈압이 135mmHg 이상입니까?	☐	☐
5. 경구 약물 투여가 가능합니까?	☐	☐
6. 블루투스 기반의 혈압측정계를 사용할 수 있으며 계획된 방문, Breakthrough visit 를 비롯한 중재 계획, 기타 시험 절차를 따를 것으로 합리적으로 기대되는 환자입니까?	☐	☐
7. 본 시험에 관한 설명을 듣고 참여하고자 직접 서면으로 동의하였습니까? 동의 날짜 (YY/MM/DD) |__|__|__|__|__|__|	☐	☐

제외기준	Yes	No
1. 임신부, 30 일 이내의 출산부, 수유 중입니까?	☐	☐
2. 다른 중재적 (interventional) 임상시험에 참여 중입니까?	☐	☐
3. 요양/재활병원, 요양원, 혹은 다른 급성기 치료병원으로 전원 예정입니까?	☐	☐
4. 3 개월의 시험기간 동안 다른 혈관 또는 두개내 시술이 계획되어 있습니까?	☐	☐
5. Olmesartan, amlodipine, hydrochlorothiazide 에 알려진 과민반응이 있습니까?	☐	☐
6. 중증 간질환(예: 복수 또는 혈액응고병증의 징후)이 알려진 환자입니까?	☐	☐
7. 투석을 요하는 신부전 환자입니까?	☐	☐
**본 임상시험에 참여하기 적합한 피험자입니까?**<br>(피험자로 선정되기 위해서는 선정기준 항목이 모두 "Yes", 제외기준 항목이 "No"이어야 합니다.)	☐	☐

등록 전 평균 수축기 혈압				
	측정일	측정시간	혈압	비고
#1	___/___/___/(yyyy/mm/dd)	___/___/(h/m)	___/___/ mmHg	
#2	___/___/___/(yyyy/mm/dd)	___/___/(h/m)	___/___/ mmHg	
#3	___/___/___/(yyyy/mm/dd)	___/___/(h/m)	___/___/ mmHg	
#4	___/___/___/(yyyy/mm/dd)	___/___/(h/m)	___/___/ mmHg	
#5	___/___/___/(yyyy/mm/dd)	___/___/(h/m)	___/___/ mmHg	
#6	___/___/___/(yyyy/mm/dd)	___/___/(h/m)	___/___/ mmHg	
평균 수축기 혈압			___/___/ mmHg	

Pregnancy Test(Urine HCG)	☐ Positive ☐ Negative ☐ N/A
현재 복용중인 병용약물이 있습니까?<br>(스크리닝 시 복용중인 약물에 대해서는 병용약물 투약내역에 기록해 주십시오.)	☐ 예 ☐ 아니오

서명일	___ 년 YY ___ 월 MM ___ 일 DD	연구자 서명

SCREENING FAILURE

BOSS-Trial I<br>CRF Version 1.3<br>on 2017-01-20	PATIENT INITIALS<br>└─┴─┴─┘	SUBJECT No.<br>└─┴─┴─┴─┘	SCREENING FAILURE

SCREENING FAILURE	
1. 연구대상자가 SCREENING FAIL 되었습니까?	□ 예 □ 아니오
2. '예'인 경우, 아래에 그 사유를 표시하여 주십시오.<br>□ 본 임상시험에서 규정된 혈압약의 투여 후 과민반응을 비롯한 예측하지 못한 이상반응이 발생하여 임상시험의 지속이 힘들다고 의학적으로 판단되는 경우<br>□ Run-in period 동안 신경학적 악화가 발생하거나 기타 임상시험의 지속이 힘들다고 연구책임자가 판단하는 경우<br>□ 임상시험에 계획된 대로 블루투스 기반의 혈압측정계의 사용이 힘들 것으로 판단되는 경우<br>□ 연구대상자가 연구에 계속 참여를 거부하는 경우<br>□ 기타 (Specify: ________________________________	
3. SCREENING FAIL 된 날짜	└┴┘└┴┘└┴┘ (YY/MM/DD)

시험자 서명 (Investigator's signature)	
본 증례기록서의 모든 항목을 검토하였으며 빠짐없이 정확하게 기재하였음을 확인합니다	
서명일 └─┴─┘└─┴─┘└─┴─┘<br>년 YY 월 MM 일 DD	연구자 서명:

Visit 2

BOSS-Trial I<br>CRF Version 1.3<br>on 2017-01-20	PATIENT INITIALS	SUBJECT No.	VISIT 2<br>(RANDOMIZATION)

방문일	년 YY 월 MM 일 DD
1. 혈압계 작동 및 데이터 전송에 대한 교육이 이루어졌습니까?	☐ 예 ☐ 아니오
2. 무작위 배정을 시행하였습니까?	☐ 예 ☐ 아니오
3. 무작위 배정 일시	년YY 월MM 일DD ____/____ (h/m)
4. 무작위 배정번호	
5. 시험 그룹	☐ Intensive management group ☐ Control group
6. 이전 방문 이후 병용약물의 변경이나 추가가 있습니까?<br>* 이전 방문 이후 병용약물의 변경이나 추가가 있는 경우, 병용약물 투약 내역에 기록해 주십시오.	☐ 예 ☐ 아니오

혈압약 투약내역(Run-in period, <5d)				
약제명	투여여부	1 일 용량	투여시작일 (YY/MM/DD)	Ongoing/ 투여종료일 (YY/MM/DD)
Olmesartan	☐ Y ☐ N	(________mg/d)		☐ /
Amlodipine	☐ Y ☐ N	(________mg/d)		☐ /
Hydrochlorothiazide	☐ Y ☐ N	(________mg/d)		☐ /
베타차단제 (성분명: ________)	☐ Y ☐ N	(________mg/d)		☐ /
기타혈압약	☐ Y ☐ N			☐ /

기타혈압약						
성분명 (또는 제품명)	투여경로	1 일 투여량	Unit/일	투여 횟수	투여시작일 (YY/MM/DD)	Ongoing/투여종료일 (YY/MM/DD)
						☐ /
						☐ /
						☐ /

BOSS-Trial I<br>CRF Version 1.3<br>on 2017-01-20	**PATIENT INITIALS**	**SUBJECT No.**	**VISIT 2**<br>(RANDOMIZATION)

						□ /

서명일	년 YY 월 MM 일 DD	연구자 서명	

Visit 3

BOSS-Trial I<br>CRF Version 1.3<br>on 2017-01-20	PATIENT INITIALS	SUBJECT No.	VISIT 3<br>(Month 1)

방문일	년 YY 월 MM 일 DD
1. Study on going	□ 예 □ 아니오<br>('아니오'인 경우 연구중단일: _ _년 _ _월 _ _일, 증례결론 작성)
2. 혈압측정	혈압:_____/_____/ mmHg 측정시간: ___/___/(h/m)
3. 혈압계 작동 및 데이터 전송	□ 예 □ 아니오<br>('아니오'인 경우 사유:)
3.1. 자가혈압측정 횟수	평균 일_____회 평균 주_____회

이상반응	
4. 이전 방문 이후 새로운 이상반응이 발생 하였습니까?<br>* 이전 방문 이후 새로운 이상반응이 발생한 경우, 이상반응 페이지를 작성해 주십시오.	□ 예 □ 아니오

병용약물	
5. 이전 방문 이후 병용약물의 변경이나 추가가 있습니까?<br>* 이전 방문 이후 병용약물의 변경이나 추가가 있는 경우, 병용약물 투약 내역에 기록해 주십시오.	□ 예 □ 아니오

6. 순응도<br>방문일 이전 지난 10 일 간 혈압약 미복용일이 있습니까?		□ 예 □ 아니오<br>_____일
7. 해당 기간 동안 다음 사건 발생		
사건 종류	발생여부	발생일<br>(YY/MM/DD)
뇌졸중	□ 예 (□ Ischemia □ Hemorrhage □ TIA)<br>□ 아니오	
심근경색	□ 예 □ 아니오	
사망	□ 예 □ 아니오	

혈압약 투약내역

약제명	투여여부	1 일 용량	투여시작일<br>(YY/MM/DD)	Ongoing/<br>투여종료일<br>(YY/MM/DD)
Olmesartan	☐ Y ☐ N	(________mg/d)		☐ /
Amlodipine	☐ Y ☐ N	(________mg/d)		☐ /
Hydrochlorothiazide	☐ Y ☐ N	(________mg/d)		☐ /
베타차단제<br>(성분명: ________)	☐ Y ☐ N	(________mg/d)		☐ /
기타혈압약	☐ Y ☐ N			☐ /

기타혈압약

성분명<br>(또는 제품명)	투여경로	1 일<br>투여량	Unit/일	투여<br>횟수	투여시작일<br>(YY/MM/DD)	Ongoing/투여종료일<br>(YY/MM/DD)
						☐ /
						☐ /
						☐ /
						☐ /

서명일	년 YY 월 MM 일 DD	연구자 서명	

Visit 4

BOSS-Trial I<br>CRF Version 1.3<br>on 2017-01-20	PATIENT INITIALS	SUBJECT No.	VISIT 4<br>(Month 3)

방문일	년 YY 월 MM 일 DD
1. Study on going	☐ 예 ☐ 아니오<br>('아니오'인 경우 연구중단일: _ _년 _ _월 _ _일, 증례결론 작성)
2. 혈압측정	혈압: ___/___/ mmHg 측정시간: __/__/(h/m)
3. 혈압계 작동 및 데이터 전송	☐ 예 ☐ 아니오<br>('아니오'인 경우 사유:)
3.1. 자가혈압측정 횟수	평균 일_____회 평균 주_____회

이상반응	
4. 이전 방문 이후 새로운 이상반응이 발생 하였습니까?<br>* 이전 방문 이후 새로운 이상반응이 발생한 경우, 이상반응 페이지를 작성해 주십시오.	☐ 예 ☐ 아니오

병용약물	
5. 이전 방문 이후 병용약물의 변경이나 추가가 있습니까?<br>* 이전 방문 이후 병용약물의 변경이나 추가가 있는 경우, 병용약물 투약 내역에 기록해 주십시오.	☐ 예 ☐ 아니오

6. 순응도<br>방문일 이전 지난 10 일 간 혈압약 미복용일이 있습니까?		☐ 예 ☐ 아니오<br>_____일
7. 해당 기간 동안 다음 사건 발생		
사건 종류	발생여부	발생일<br>(YY/MM/DD)
뇌졸중	☐ 예 (☐ Ischemia ☐ Hemorrhage ☐ TIA)<br>☐ 아니오	
심근경색	☐ 예 ☐ 아니오	
사망	☐ 예 ☐ 아니오	

BOSS-Trial I<br>CRF Version 1.3<br>on 2017-01-20	PATIENT INITIALS<br>└┴┴┘	SUBJECT No.<br>└┴┴┴┘	VISIT 4<br>(Month 3)

혈압약 투약내역				
약제명	투여여부	1 일 용량	투여시작일<br>(YY/MM/DD)	Ongoing/<br>투여종료일<br>(YY/MM/DD)
Olmesartan	☐ Y ☐ N	(________mg/d)	└┴┴┴┴┘	☐ / └┴┴┴┴┘
Amlodipine	☐ Y ☐ N	(________mg/d)	└┴┴┴┴┘	☐ / └┴┴┴┴┘
Hydrochlorothiazide	☐ Y ☐ N	(________mg/d)	└┴┴┴┴┘	☐ / └┴┴┴┴┘
베타차단제<br>(성분명: ________)	☐ Y ☐ N	(________mg/d)	└┴┴┴┴┘	☐ / └┴┴┴┴┘
기타혈압약	☐ Y ☐ N		└┴┴┴┴┘	☐ / └┴┴┴┴┘

기타혈압약						
성분명<br>(또는 제품명)	투여경로	1 일<br>투여량	Unit/일	투여<br>횟수	투여시작일<br>(YY/MM/DD)	Ongoing/투여종료일<br>(YY/MM/DD)
					└┴┴┴┴┘	☐ / └┴┴┴┴┘
					└┴┴┴┴┘	☐ / └┴┴┴┴┘
					└┴┴┴┴┘	☐ / └┴┴┴┴┘
					└┴┴┴┴┘	☐ / └┴┴┴┴┘

서명일	└┴┴┴┴┘<br>년 YY 월 MM 일 DD	연구자 서명	

Breakthrough Visit

(돌발성 방문을 요청할 때마다 작성해주십시오)

No. of Breakthrough Visit: ________

BOSS-Trial I<br>CRF Version 1.3<br>on 2017-01-20	PATIENT INITIALS	SUBJECT No.	BREAKTHROUGH VISIT

**1. 돌발성 방문 요청일**	년 YY 월 MM 일 DD
**2. 돌발성 방문 요청사유**	□ 혈압상승 □ 혈압저하 □ 기타(Specify:________________)
**3. 돌발성 방문 요청에 따른 실제 방문여부**	**□ 예 ('예'인 경우 4 번 이하 항목 작성)**<br>**□ 아니오 ('아니오'인 경우 사유:____________________)**
**4. 돌발성 방문 요청에 따른 실제 방문일**	년 YY 월 MM 일 DD
**5. Study on going**	□ 예 □ 아니오<br>('아니오'인 경우 연구중단일: _ _년 _ _월 _ _일, 증례결론 작성)
**6. 혈압측정**	혈압:______/_______/ mmHg 측정시간: ___/___/(h/m)
**7. 혈압계 작동 및 데이터 전송**	□ 예 □ 아니오<br>('아니오'인 경우 사유:)

**이상반응**	
8. 이전 방문 이후 새로운 이상반응이 발생 하였습니까?<br>* 이전 방문 이후 새로운 이상반응이 발생한 경우, 이상반응 페이지를 작성해 주십시오.	□ 예 □ 아니오

**병용약물**	
9. 이전 방문 이후 병용약물의 변경이나 추가가 있습니까?<br>* 이전 방문 이후 병용약물의 변경이나 추가가 있는 경우, 병용약물 투약 내역에 기록해 주십시오.	□ 예 □ 아니오

10. 순응도<br>방문일 이전 지난 10 일 간 혈압약 미복용일이 있습니까?	□ 예 □ 아니오<br>_____일
11. 해당 기간 동안 다음 사건 발생	

사건 종류	발생여부	발생일 (YY/MM/DD)
뇌졸중	□ 예 (□ Ischemia □ Hemorrhage □ TIA)<br>□ 아니오	
심근경색	□ 예 □ 아니오	
사망	□ 예 □ 아니오	

BOSS-Trial I<br>CRF Version 1.3<br>on 2017-01-20	PATIENT INITIALS	SUBJECT No.	BREAKTHROUGH VISIT

혈압약 투약내역				
약제명	투여여부	1 일 용량	투여시작일 (YY/MM/DD)	Ongoing/ 투여종료일 (YY/MM/DD)
Olmesartan	☐ Y ☐ N	(_________mg/d)		☐ /
Amlodipine	☐ Y ☐ N	(_________mg/d)		☐ /
Hydrochlorothiazide	☐ Y ☐ N	(_________mg/d)		☐ /
베타차단제<br>(성분명: _________)	☐ Y ☐ N	(_________mg/d)		☐ /
기타혈압약	☐ Y ☐ N			☐ /

기타혈압약						
성분명<br>(또는 제품명)	투여경로	1 일<br>투여량	Unit/일	투여<br>횟수	투여시작일<br>(YY/MM/DD)	Ongoing/투여종료일<br>(YY/MM/DD)
						☐ /
						☐ /
						☐ /
						☐ /

서명일	년 YY 월 MM 일 DD	연구자 서명

Unplanned Visit

(Optional)

BOSS-Trial I<br>CRF Version 1.3<br>on 2017-01-20	PATIENT INITIALS<br>└─┴─┴─┘	SUBJECT No.<br>└─┴─┴─┴─┘	UNPLANNED VISIT

방문일	└─┴─┴─┴─┴─┘<br>년 YY 월 MM 일 DD
1. Study on going	☐ 예 ☐ 아니오<br>('아니오'인 경우 연구중단일: _ _년 _ _월 _ _일, 증례결론 작성)
2. 혈압측정	혈압:______/_______/ mmHg 측정시간: ___/___/(h/m)
3. 혈압계 작동 및 데이터 전송	☐ 예 ☐ 아니오<br>('아니오'인 경우 사유:)
4. 방문 사유	☐ 혈압상승 ☐ 혈압저하 ☐ 기타(Specify:______________________)

이상반응	
5. 이전 방문 이후 새로운 이상반응이 발생 하였습니까?<br>* 이전 방문 이후 새로운 이상반응이 발생한 경우, 이상반응 페이지를 작성해 주십시오.	☐ 예 ☐ 아니오

병용약물	
6. 이전 방문 이후 병용약물의 변경이나 추가가 있습니까?<br>* 이전 방문 이후 병용약물의 변경이나 추가가 있는 경우, 병용약물 투약 내역에 기록해 주십시오.	☐ 예 ☐ 아니오

7. 순응도<br>방문일 이전 지난 10 일 간 혈압약 미복용일이 있습니까?		☐ 예 ☐ 아니오<br>______일
8. 해당 기간 동안 다음 사건 발생		
사건 종류	발생여부	발생일<br>(YY/MM/DD)
뇌졸중	☐ 예 (☐ Ischemia ☐ Hemorrhage ☐ TIA)<br>☐ 아니오	└─┴─┴─┘
심근경색	☐ 예 ☐ 아니오	└─┴─┴─┘
사망	☐ 예 ☐ 아니오	└─┴─┴─┘

BOSS-Trial I<br>CRF Version 1.3<br>on 2017-01-20	PATIENT INITIALS	SUBJECT No.	UNPLANNED VISIT

혈압약 투약내역				
약제명	투여여부	1 일 용량	투여시작일 (YY/MM/DD)	Ongoing/ 투여종료일 (YY/MM/DD)
Olmesartan	☐ Y ☐ N	(_________mg/d)		☐ /
Amlodipine	☐ Y ☐ N	(_________mg/d)		☐ /
Hydrochlorothiazide	☐ Y ☐ N	(_________mg/d)		☐ /
베타차단제 (성분명: _________)	☐ Y ☐ N	(_________mg/d)		☐ /
기타혈압약	☐ Y ☐ N			☐ /

기타혈압약						
성분명 (또는 제품명)	투여경로	1 일 투여량	Unit/일	투여 횟수	투여시작일 (YY/MM/DD)	Ongoing/투여종료일 (YY/MM/DD)
						☐ /
						☐ /
						☐ /
						☐ /

서명일	년 YY 월 MM 일 DD	연구자 서명	

증례결론
(End of Study)

임상시험이 종료(완료, 탈락)된 피험자의 경우, 상세내용을 기록합니다.

BOSS-Trial I<br>CRF Version 1.3<br>on 2017-01-20	PATIENT INITIALS<br>|___|___|___|	SUBJECT No.<br>|___|___|___|___|	UNPLANNED VISIT

증례결론	
1. 연구대상자가 임상시험 동안의 계획된 방문을 모두 완료하고 종료하였습니까?	□ 예 □ 아니오
2. '아니오'인 경우, 중도 탈락된 사유를 표시하여 주십시오.<br>□ 사망<br>□ 시험대상자의 동의철회<br>□ 임상시험 미준수<br>□ 계획서 위반<br>□ 연구자에 의한 중단<br>□ 기타 (Specify: ____________________)	
3. 중도 탈락일	|___|___|___|(YY/MM/DD)
4. 마지막 접촉	□ 직접 방문 □ 전화 방문 □ 기타: specify :__________
5. 연구 참여기간동안 휴대전화의 교체가 있었습니까?	□ 예 □ 아니오<br>('예'인 경우 교체 횟수: ______회)

COMMENT (필요시)

시험자 서명 (Investigator's signature)	
본 증례기록서의 모든 항목을 검토하였으며 빠짐없이 정확하게 기재하였음을 확인합니다	
서명일 |___|___|___| 년 YY 월 MM 일 DD	연구자 서명:

SPECIAL FORM I

이상반응

BOSS-Trial I<br>CRF Version 1.3<br>on 2017-01-20	PATIENT INITIALS<br>└─┴─┴─┘	SUBJECT No.<br>└─┴─┴─┴─┘	SPECIAL FORM I<br>(이상반응)

이상반응
Adverse event

임상시험 기간 동안 발현된 이상반응을 기재하십시오

**이상반응명**			
**발현일 (YY/MM/DD)**			
**소실일 (YY/MM/DD)**	지속 ☐	지속 ☐	지속 ☐
**중증도**	☐ 경증<br>☐ 중등증<br>☐ 중증	☐ 경증<br>☐ 중등증<br>☐ 중증	☐ 경증<br>☐ 중등증<br>☐ 중증
**중대한 이상반응**	☐ 예 ☐ 아니오<br>* '예'인 경우 중대한 이상반응 보고서를 작성해 주십시오.	☐ 예 ☐ 아니오<br>* '예'인 경우 중대한 이상반응 보고서를 작성해 주십시오.	☐ 예 ☐ 아니오<br>* '예'인 경우 중대한 이상반응 보고서를 작성해 주십시오.
**관련성**	☐ 명확히 관련 있음<br>☐ 관련이 있음<br>☐ 관련 가능성 있음<br>☐ 관련이 없음<br>☐ 명확히 관련 없음<br>☐ 불명	☐ 명확히 관련 있음<br>☐ 관련이 있음<br>☐ 관련 가능성 있음<br>☐ 관련이 없음<br>☐ 명확히 관련 없음<br>☐ 불명	☐ 명확히 관련 있음<br>☐ 관련이 있음<br>☐ 관련 가능성 있음<br>☐ 관련이 없음<br>☐ 명확히 관련 없음<br>☐ 불명
**조치**	☐ 없음<br>☐ 일시 중단<br>☐ 영구 중단<br>☐ 알 수 없음<br>☐ 해당사항 없음	☐ 없음<br>☐ 일시 중단<br>☐ 영구 중단<br>☐ 알 수 없음<br>☐ 해당사항 없음	☐ 없음<br>☐ 일시 중단<br>☐ 영구 중단<br>☐ 알 수 없음<br>☐ 해당사항 없음
**치료**	☐ 치료 안함<br>☐ 약물치료<br>☐ 비약물치료<br>☐ 약물+비약물치료	☐ 치료 안함<br>☐ 약물치료<br>☐ 비약물치료<br>☐ 약물+비약물치료	☐ 치료 안함<br>☐ 약물치료<br>☐ 비약물치료<br>☐ 약물+비약물치료
**결과**	☐ 회복/해결됨<br>☐ 회복/해결되었으나 후유증 남음<br>☐ 회복/해결되지 않음<br>☐ 사망<br>☐ 알 수 없음	☐ 회복/해결됨<br>☐ 회복/해결되었으나 후유증 남음<br>☐ 회복/해결되지 않음<br>☐ 사망<br>☐ 알 수 없음	☐ 회복/해결됨<br>☐ 회복/해결되었으나 후유증 남음<br>☐ 회복/해결되지 않음<br>☐ 사망<br>☐ 알 수 없음
**서명 날짜 (YY/MM/DD)**			
**comment**			
**연구자 서명**			

SPECIAL FORM II

중대한 이상반응 보고서

BOSS-Trial Ⅰ<br>CRF Version 1.3<br>on 2017-01-20	PATIENT INITIALS<br>└─┴─┴─┘	SUBJECT No.<br>└─┴─┴─┴─┘	SPECIAL FORM Ⅱ<br>(중대한 이상반응)

중대한 이상반응 보고서

SERIOUS ADVERSE EVENT REPORT

REPORT TYPE :

☐ Initial Report (YY/MM/DD: ________________________________)

☐ Follow-up Report<br>(YY/MM/DD: ____________)	Follow-up Report<br>No: __	The latest Follow-up Report Date:<br>(YY/MM/DD: ____________)

Section 1 Demographic Data

생년월일 :<br>(YYYY/MM/DD: ____________)	성별<br>☐ 남 ☐ 여	신장<br>__________cm	몸무게<br>__________kg

Section 2 SAE Information

**이상반응 명**		**중증도**	☐ 경증<br>☐ 중등증<br>☐ 중증
**발현일**<br>**(YY/MM/DD)**	└─┴─┴─┘	**소실일**<br>**(YY/MM/DD)**	└─┴─┴─┘ 지속 ☐
**SAE 종류**	☐ 사망<br>✓ *사망일(YY/MM/DD) :* └─┴─┴─┘<br>✓ *사인:* ______________________<br>✓ *부검여부* ☐ *Yes* ☐ *No* ☐ *Unknown*<br>✓ *부검소견:*______________________<br>☐ 생명을 위협<br>☐ 입원 또는 입원기간의 연장<br>☐ 지속적 또는 의미 있는 불구나 기능저하 초래<br>☐ 선천성 기형 또는 이상 초래<br>☐ 기타 의학적으로 중요한 상황		
**연관성**	☐ 명확히 관련 있음<br>☐ 관련이 있음<br>☐ 관련 가능성이 있음<br>☐ 관련이 없음<br>☐ 명확히 관련 없음<br>☐ 불명	**결과**	☐ 회복/해결됨<br>☐ 회복/해결되지 않음<br>☐ 회복/해결되었으나 후유증 남음<br>☐ 사망<br>☐ 추적조사 실패/알 수 없음
**조치**	☐ 중재 중지<br>☐ 변화 없음<br>☐ 알 수 없음<br>☐ 해당사항 없음		

Section 3 Medical History

Specify past or current medical disorders, allergies, surgeries, etc. that can help explain the SAE	Start Date (YY/MM/DD)	End Date (YY/MM/DD)	Condition Present at Time of the SAE? *Y=Yes N=No UK=Unknown*	Comments

Section 4 Concomitant Medications Information

Recent/Concomitant Drug(s) (within 14 days of the onset of SAE) (exclude those used to treat events)	Daily Dose(s)	Start Date (YY/MM/DD)	End Date (YY/MM/DD)	Indication for Use

Reporter's Comments :

Reporting Information

Reporter's Name/ Reporting Date : / (YY/MM/DD)

Principal Investigator's Name / Sign / Date: / / (YY/MM/DD)

SPECIAL FORM III

병용약물 투약내역

BOSS-Trial Ⅰ<br>CRF Version 1.3<br>on 2017-01-20	**PATIENT INITIALS**	**SUBJECT No.**	**SPECIAL FORMⅢ<br>(병용약물 투약내역)**

병용약물 투약 내역						
성분명<br>(또는 제품명)	투여경로	1 일 투여량	Unit/일	투여<br>횟수	투여시작일<br>(YY/MM/DD)	Ongoing/투여종료일<br>(YY/MM/DD)
						□ /
						□ /
						□ /
						□ /
						□ /
						□ /
						□ /
						□ /
						□ /
						□ /
						□ /
						□ /
						□ /
						□ /
						□ /
						□ /
