# Supplementary material for: Remote blood pressure monitoring and behavioral intensification for stroke: A randomized controlled feasibility trial
Source: PLoS One. 2020 Mar 11;15(3):e0229483. doi: 10.1371/journal.pone.0229483 (PMC7065804; doi:10.1371/journal.pone.0229483)
Supplement: S2 Protocol — (PDF) [file pone.0229483.s017.pdf]

Optimization of Blood Pressure Management After Acute Ischemic Stroke and Its Prognostic Significance

The safety and scientific validity of this study is the responsibility of the study sponsor and investigators. Listing a study does not mean it has been evaluated by the U.S. Federal Government. Read our disclaimer for details.

ClinicalTrials.gov Identifier: NCT03024476

Recruitment Status : Completed
First Posted : January 18, 2017
Last Update Posted : April 6, 2018

Sponsor:
Seoul National University Bundang Hospital

Collaborator:
Daiichi Sankyo Korea Co., Ltd.

Information provided by (Responsible Party):
Hee-Joon Bae, Seoul National University Bundang Hospital

Study Description

Go to

Brief Summary:

BOSS-Trial I is a phase 2 clinical trial with the following objectives;

1. to prove the feasibility of a Bluetooth-equipped sphygmomanometer system in real-world clinical practice and wireless connection to the main server;
2. to prove the feasibility of the BP management strategy, including the pre-specified BP

range, BP management algorithm, and behavioral; and

3. to gather information for the phase 3 trial including BP variability indices and their potentials as a treatment guidance.

Condition or disease	Intervention/treatment	Phase
Ischemic Stroke<br>Hypertension	Behavioral: Behavioral intensification<br>Drug: Pharmacological intensification based on olmesartan<br>Device: Bluetooth-equipped sphygmomanometer	Phase 2

Detailed Description:

- Elevated blood pressure is an established risk factor for recurrent stroke and vascular events in ischemic stroke survivors, but
- Current guideline (JNC VIII) has omitted or only partially covered a number of practical and important issues as follow;
  - When and how we measure blood pressure?
  - Is it justifiable to apply the same blood pressure threshold for office BP and home BP?
  - Should stroke survivors be treated by the same BP goal for non-stroke subjects?
  - Is it justifiable to apply the universal BP threshold for different mechanisms of ischemic stroke?
  - Is it really about only blood pressure or might it really be "beyond blood pressure?"
- Lifestyle modification should accompany all the pharmacological intervention but is usually in adequate to initiate behavioral changes.
- Frequent BP measurement at home will provide more detailed and reliable information than occasional office BP's.

Study Design

Go to

Study Type :	Interventional (Clinical Trial)
Actual Enrollment :	67 participants
Allocation:	Randomized
Intervention Model:	Parallel Assignment
Masking:	None (Open Label)
Primary Purpose:	Treatment

Official Title:	Optimization of Blood Pressure Management After Acute Ischemic Stroke and Its Prognostic Significance: Prospective, Randomized, Open, Blinded Outcome Evaluation, and Feasibility Trial
Actual Study Start Date :	September 1, 2016
Actual Primary Completion Date :	December 7, 2017
Actual Study Completion Date :	December 7, 2017

Resource links provided by the National Library of Medicine NIH NLM

Genetics Home Reference related topics:
Hypertension

Drug Information available for: Olmesartan

U.S. FDA Resources

Arms and Interventions

Go to

Arm	Intervention/treatment
Experimental: Intensive management arm<br>Description:<br>• Interventions in the intensive management arm consist of 1) behavioral intensification and 2) pharmacological intensification based on olmesartan<br>• Participants will be given a wireless Bluetooth-equipped sphygmomanometer system, which is connected to the main server through the participants' own smartphone. Every blood pressure and heart rate measured will be encrypted and stored in the main server (Identical for both intervention and control groups).<br>• Regarding behavioral intensification, an automated texting and call for breakthrough visit will be sent from the	Behavioral: Behavioral intensification<br>Suggested algorithm for behavioral intensification:<br>• If frequency of BP measurement ≤5 in a week, send a texting message<br>• If frequency of BP measurement ≤3 in a week, make a telephone contact by research nurse<br>• Target range of home-systolic blood pressure: 110 - 135<br>• If rate of outlier exceeds 50% in a week (based on ≥5 measurements in a week), make a telephone contact by research nurse (applied at least 2 weeks after randomization). A call for breakthrough visit may be issued when clinically required (decided by

main server to encourage regular measurements of BP and maintain a desirable goad of BP.
- Regarding pharmacological intensification, a specific algorithm for BP-lowering medication prescription will be provided to the responsible physicians by the steering committee.

the institutional investigator)
- If frequency of BP measurement ≥ 10 in a week and frequency of outlier = 0 in a week, send a texting message to encourage and compliment on excellent BP management

Drug: Pharmacological intensification based on olmesartan

- Study drug will be provided from the roll-in period.
- Step I:
  - Use olmesartan 20 mg only if mean systolic blood pressure ≤150 mm Hg during the immediate past 2 days
  - Use olmesartan 40 mg if mean systolic blood pressure >150 during the immediate past 2 days
- Step II: olmesartan 20 mg or 40 mg + amlodipine 5 mg
- Step III: olmesartan 20 mg or 40 mg + amlodipine 10 mg
- Step IV: olmesartan 20 mg or 40 mg + amlodipine 10 mg + hydrochlorothiazide 12.5 mg
- If blood pressure profile showed high degree variability to evoke clinical concern, consider discontinuation of hydrochlorothiazide or reduction of olmesartan dose.
- Use of beta-blockers is permitted if clinically indicated.
- At each clinic visit regardless of regular or breakthrough visit, follow the pharmacological intensification

	rule.<br><br>Device: Bluetooth-equipped sphygmomanometer<br>• Participants will be given a wireless Bluetooth-equipped sphygmomanometer system, which is connected to the main server through the participants' own smartphone. (Identical for both intervention and control groups)<br>• Every blood pressure and heart rate measured will be encrypted and stored in the main server. (Identical for both intervention and control groups)
Active Comparator: Control arm<br>Description:<br>• Other than a bluetooth-equipped sphygmomanometer, standard managements abiding by the most current guideline will be provided from the responsible physicians.<br>• Participants will be given a Bluetooth-equipped sphygmomanometer, which is connected to the main server through the participants' own smartphone. Every blood pressure and heart rate measured will be encrypted and stored in the main server (Identical for both intervention and control groups).	Device: Bluetooth-equipped sphygmomanometer<br>• Participants will be given a wireless Bluetooth-equipped sphygmomanometer system, which is connected to the main server through the participants' own smartphone. (Identical for both intervention and control groups)<br>• Every blood pressure and heart rate measured will be encrypted and stored in the main server. (Identical for both intervention and control groups)

Outcome Measures

Go to

Primary Outcome Measures :

1. Recruitment time to prespecified number of subjects [Time Frame: At 3 months after randomization]

   Difference in days between recruitment of the first subject and last subject

2. Retention of included participants [Time Frame: At 3 months after randomization]

   Ratio of completed subject over randomized subjects in each group

3. Frequencies of calls for breakthrough visit [Time Frame: At 3 months after randomization]

   Mean and standard deviation of breakthrough visits per each patient in the intensive management group

4. Rate of patients who responded to the calls for breakthrough visit [Time Frame: At 3 months after randomization]

   ratio of subjects response over the breakthrough visit calls

5. Control of blood pressure [Time Frame: At 3 months after randomization]

   ratio of subjects with well-controlled BP in each group

Secondary Outcome Measures :

1. Frequency of out-of-range measurement [Time Frame: At 3 months after randomization]

   Frequency of BP measurements out of the desirable BP range in a week

2. Weighted hit score of BP [Time Frame: At 3 months after randomization]

   When two consecutive hits crossed over or below the margin of desirable BP, give 2x weight. Final score will be generated by dividing by total number of measurements

3. Vascular events [Time Frame: At 3 months after randomization]

   Recurrent vascular events including recurrent stroke, myocardial infarction or vascular death

4. Hypotensive events [Time Frame: Until 3 months after randomization]

   Complaint of dizzy spells, falls or low-BP related events by patients

Eligibility Criteria

Go to

Information from the National Library of Medicine

NIH NLM

Choosing to participate in a study is an important personal decision. Talk with your doctor and family members or friends about deciding to join a study. To learn more about this study, you or your doctor may contact the study research staff using the contacts provided below. For general information, *Learn About Clinical Studies.*

Ages Eligible for Study:	19 Years and older (Adult, Older Adult)
Sexes Eligible for Study:	All
Accepts Healthy Volunteers:	No

Criteria

Inclusion Criteria:

- Ischemic stroke survivors within 7 days after onset
- ≥19 year-old male or female
- Medically and neurologically stabilized enough to take BP-lowering medication
- Mean systolic blood pressure ≥135 mm Hg during two days between at least 24 hours after onset and randomization (whether BP-lowering medication was prescribed or not)
- Capable of taking oral medication
- Capable of operating a wireless Bluetooth-equipped sphygmomanometer system and being expected to follow required procedures of the clinical trial
- Patients who provided written informed consent

Exclusion Criteria:

- Pregnant, puerperium ≤30 days or on breastfeeding
- enrolled in other interventional clinical trial
- Being transferred to rehabilitation center or institutionalized
- Being expected to have cerebral artery interventions within 3 months after randomization
- Known allergic reactions to olmesartan, amlodipine or hydrochlorothiazide
- Known severe hepatic disease
- Advanced kidney dysfunction requiring dialysis
- Being unlikely, in the opinion of the investigator, to comply with the clinical trial protocol or

being unsuitable for any other reason.

Contacts and Locations

Go to

Information from the National Library of Medicine

To learn more about this study, you or your doctor may contact the study research staff using the contact information provided by the sponsor.

Please refer to this study by its ClinicalTrials.gov identifier (NCT number): ***NCT03024476***

Locations

Korea, Republic of

Seoul National University Bundang Hospital
Seongnam, Gyeonggi, Korea, Republic of, 13520

Nowon Eulji Medical Center, Eulji University
Seoul, Korea, Republic of, 01830

Seoul Medical Center
Seoul, Korea, Republic of, 02053

Sponsors and Collaborators

Seoul National University Bundang Hospital

Daiichi Sankyo Korea Co., Ltd.

Investigators

Principal Investigator: Hee-Joon Bae, MD.PhD Seoul National University Bundang Hospital

More Information

Go to

Responsible Party:	Hee-Joon Bae, Professor, Seoul National University Bundang Hospital
ClinicalTrials.gov Identifier:	NCT03024476 History of Changes
Other Study ID Numbers:	B-1604/343
First Posted:	January 18, 2017 Key Record Dates
Last Update Posted:	April 6, 2018
Last Verified:	April 2018

Individual Participant Data (IPD) Sharing Statement:

Plan to Share IPD: Undecided

Keywords provided by Hee-Joon Bae, Seoul National University Bundang Hospital:
behavioral intervention
prespecified medication algorithm
bluetooth-equipped sphygmomanometer

Additional relevant MeSH terms:

Stroke	Pathologic Processes
Ischemia	Brain Infarction
Cerebral Infarction	Brain Ischemia
Cerebrovascular Disorders	Olmesartan
Brain Diseases	Olmesartan Medoxomil
Central Nervous System Diseases	Antihypertensive Agents
Nervous System Diseases	Angiotensin II Type 1 Receptor Blockers
Vascular Diseases	Angiotensin Receptor Antagonists
Cardiovascular Diseases	Molecular Mechanisms of Pharmacological Action
