# Supplementary material for: Remote blood pressure monitoring and behavioral intensification for stroke: A randomized controlled feasibility trial
Source: PLoS One. 2020 Mar 11;15(3):e0229483. doi: 10.1371/journal.pone.0229483 (PMC7065804; doi:10.1371/journal.pone.0229483)
Supplement: Attachment — Submitted filename: BOSS Trial_PLoS One_17_Response to Reviewers.pdf [file pone.0229483.s018.pdf]

Rebuttal to the reviewers' comment

PLoS One
Manuscript ID, PONE-D-19-21291
Manuscript title: Remote Blood Pressure Monitoring And Behavioral Intensification for Stroke: A Randomized Controlled Feasibility Trial
Lead author: Beom Joon Kim
Corresponding Author: Hee-Joon Bae

December 1st, 2019

Dear reviewers and editorial staffs of PLoS One;

We would like to thank the reviewers for their thoughtful comments on our manuscript. We provide responses to the reviewers' comments below. Responses will follow the verbatim of reviewers' comments (blue color). Additionally, we modified several sentences to make our point clearer. All the changes were specified in the marked version of our draft.

Reviewer #1;

The authors have reported an excellent method for the remote BP measurement in ischemic stroke patients and demonstrated that it is feasible and safe. The study and its design is appropriate and the results have been well analysed.

We appreciate the reviewer's positive evaluation of our study.

The following minor suggestions are provided to make the manuscript more appealing for the readers.

1. The authors should add a relevant recent ref. for smartphone based mobile healthcare devices in the introduction.

Vashist, S. K., & Luong, J. H. T. (2019). Commercially Available Smartphone-Based Personalized Mobile Healthcare Technologies. In Point-of-Care Technologies Enabling Next-Generation Healthcare Monitoring and Management (pp. 81-115). Springer, Cham.

Thank you for the reviewer's recommendation for valuable and informative reference. We cited the above-mentioned book chapter as reference #6 with the following statements in page 5;

Recently, there are many smartphone-based mobile healthcare devices that are commercially available due to the development of communication technology. Many companies offer FDA-cleared or EC Medical-certified wireless blood pressure monitors

for telemedicine or user convenience. (6)

2. The authors should mention about the regulatory status of the Bluetooth-equipped sphygmomanometer (HEM-9200T, Omron Healthcare, Co Ltd, Kyoto, Japan) whether it is FDA and/or CE certified. Do the authors know whether the device has been checked for its equivalence to a clinically-validated predicate device for BP measurement?

Before starting this study, we received data on the regulation of HEM-9200T equipment from OMRON and used it as an attachment to IRB review. This wireless blood pressure measurement device is a minor modification to the currently marketed HEM-7311 cleared to market under 510 (k) K133379. The following statement is added to the text in page 6.

The HEM-9200T, a bluetooth-equipped sphygnomamometer for the current trial, is a minor modification to the currently marketed HEM-7311 cleared to market under 510 (k) K133379, with minor button changes and addition BLE communication function.

3. If there are any limitations of the approach or the mobile healthcare technology involved, these need to be clearly reported in discussion. The most critical is the interface of the commercial mobile healthcare devices to the regulatory healthcare information systems (HIS) being used in healthcare. The second is the security of the data.

4. Some companies such as iHealth have developed dedicated softwares such as iHealth Pro in which they can integrate data from several mobile healthcare devices used by patients directly into a single software that can be easily viewed by doctor and easy to integrate with HIS. The authors should comment on this and discuss it in the discussion section.

We concur to the reviewer's here. According to the reviewer #3's comments, we revised the manuscript to discuss more about the lessons from our trial for future researchers and trialists. In this regard, we added the following paragraph to the end of discussion section;

Several practical points are worthy of discussion for future trials or researches. Future implementation of a similar remote system should comply with the local regulations in the security of data storage and transmission. The data server in this trial had physically separated from the hospital information system. Smartphones are widely used, but the elderly usually confronts with many difficulties in using and manipulating smartphones. Interfaces between smartphones and wireless healthcare devices, including blood pressure monitors, are still too difficult for them. To overcome the issue of "IT literacy," a consumer-friendly user interface design such as iHealth Labs will be helpful.

5. The most significant advantage of mobile healthcare is the significantly reduced healthcare monitoring and management costs and more compliance expected from the patients together with their comfort and reduced time spent in clinics for follow ups. It would be useful if authors can comment on this imp aspect.

We agree to the Reviewer #1's point here. Unfortunately, however, this study was not designed to prove or document changes in the overall healthcare cost either directly or indirectly. In this sense, it would be inappropriate for us to make comments on this point.

Reviewer #2;

Authors present a report of a feasibility trial testing an intervention comprising of remote BP monitoring and behavioral intensification involving 60 ischemic stroke survivors. This report is well written and presents the data in a coherent manner. I wonder why it took a bit of time to reach sample size in reasonable time from 3 study sites.

The reviewer's point was duly noted.

The best candidates for the current trial had to be limited to ischemic stroke survivors who were young and being familiar to IT technology with mild neurological deficits. We also developed the smartphone application only for Android system, as the market share of iPhone in Korea is estimated to be one-quarter and iOS phones are particularly popular among younger generations. Therefore, the strict selection criteria of our trial made it rather difficult to enroll majority of stroke admissions.

However, the speed of enrollment was rather constant throughout the trial period, as shown in the figure below.

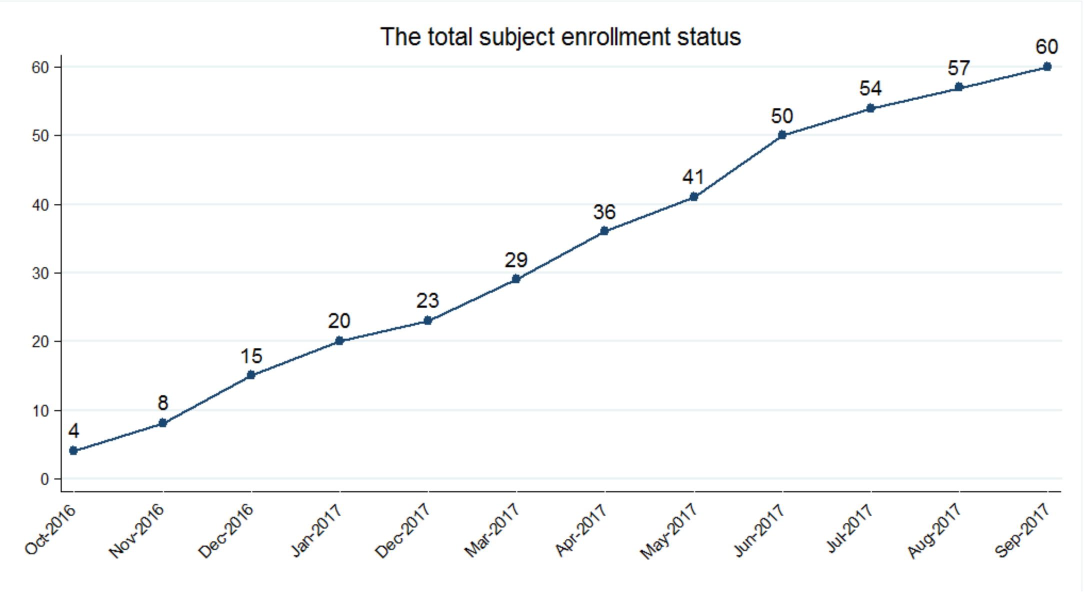

As to our response to the Reviewer #1, future trials and researches need to overcome the issue of "IT literacy" to facilitate patient recruitment and wider implementation of such system. This point was added to the discussion section as following;

Several practical points are worthy of discussion for future trials or researches. Future implementation of a similar remote system should comply with the local regulations in

the security of data storage and transmission. The data server in this trial had physically separated from the hospital information system. Smartphones are widely used, but the elderly usually confronts with many difficulties in using and manipulating smartphones. Interfaces between smartphones and wireless healthcare devices, including blood pressure monitors, are still too difficult for them. To overcome the issue of "IT literacy," a consumer-friendly user interface design such as iHealth Labs will be helpful.

Figure 4, week 15 systolic BP bar plot for the intensive group does not have confidence intervals showing in the figure.

The last visit of a study subject was supposed to occur at week 14. However, three subjects from the intensive management group visited during week #15 and thus the whisker mark of the box-and-whisker plot cannot be drawn.

Reviewer #3;

This study tested the feasibility of a trial for effects of a mobile BP management strategy vs. “usual care” for patients with acute ischemic stroke. Sixty participants were assigned randomly to intensive management or control groups. Five primary feasibility outcomes were examined, with 57 participants included in the primary analysis.

The authors are to be commended for presenting an exhaustive review of the trial findings; the study was obviously very well planned, and conducted and documented with great care. While the writing could benefit from careful review by a native English speaker to correct a number of minor grammatical errors, overall the results are clearly presented, and they are presented in an impressive amount of detail.

We appreciate the reviewer's positive evaluation of our study.

I do have some comments and suggestions that I believe would improve this manuscript; these are primarily related to the way certain aspects of the study are reported and the conclusions that have been reached.

1. The authors are actually varying two things in this study: (1) the monitoring system with or without fairly intensive support and (2) prescribed vs discretionary hypertension treatment. While the “control” group is receiving “usual care” in terms of being treated at the discretion of their physician, I am assuming that being given home monitoring equipment is not “usual” for ischemic stroke patients, so “usual care” isn’t really correct. This should be made more clear. Had a difference in BP management been observed, I’m not sure if the authors would have been able to differentiate the effects of intense monitoring from effects of differences in BP treatment. The authors should explain/justify why the treatment strategies were different for the two groups. This was either their decision, or they had no choice. Either way, this should be explained/justified.

The reviewer's points were duly noted.

In this trial, study subjects were provided a bluetooth-equipped sphygmomanometer, and that would have worked as a behavioral stimulus to them. However, for the sake of secondary prevention, it is usual practice to urge stroke patients to check their blood pressure at home, write it down, and bring it back to regular clinic visits. In this sense, we assume the behavioral stimulus by getting the BP check devices at discharge will soon fade out eventually without any recurring reinforcement.

We added the following statement in the discussion section;

Providing a sphygnomanometer itself may have worked as a behavioral stimulus.

2. Recruiting took a very long time, and did not meet the authors' "success criteria." Since a larger trial would require an even larger cohort, it would be helpful if the authors discussed why they felt this was a problem and what they think might be done about it. The authors state that only 4.4% of patients with ischemic stroke during the time of the study were randomized, but the screening figure only gives information on those who were actually screened. Was the problem the fact that the eligibility criteria ruled out the majority of stroke patients, perhaps due to lack of technical ability? What percentage of the other 95.6% were actually eligible? How many eligible patients were approached and declined? How many would have been eligible except for a lack of technical skill? This information will be valuable in planning future recruiting efforts.

The best candidates for the current trial had to be limited to ischemic stroke survivors who were young and being familiar to IT technology with mild neurological deficits. We also developed the smartphone application only for Android system, as the market share of iPhone in Korea is estimated to be one-quarter and iOS phones are particularly popular among younger generations. Therefore, the strict selection criteria of our trial made it rather difficult to enroll majority of stroke admissions.

However, the speed of enrollment was rather constant throughout the trial period, as shown in the figure below.

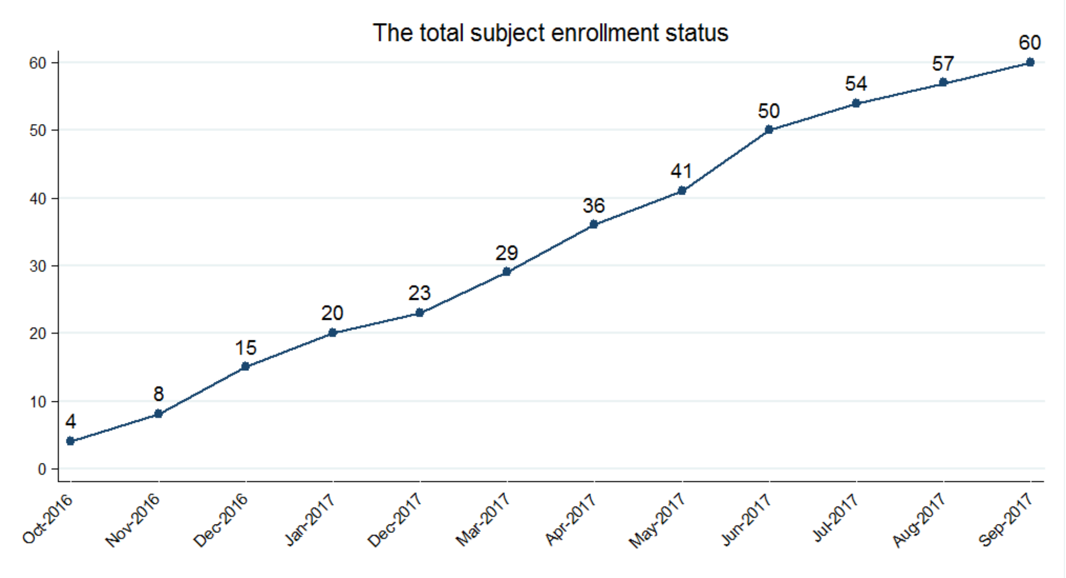

Regarding the screened-out subjects, we have to admit that we do not have the information. Because our IRBs regard a screening process itself as a study procedure, it should be started after getting relevant consent from the patient. Therefore, we have no information how patients were screened out before getting consent. We had randomized 60 out of 61 screened subjects as described in the main text. The one subject had a low-priced smartphone manufactured by TCL, that we could not establish secure bluetooth connection with the BP measuring device.

3. The authors state in the Results that "Unexpected and unexplained technical failures infrequently occurred during the trial period" but in the Discussion say "The Bluetooth-based wireless BP collection system faced mechanical failure not infrequently and required centralized monitoring and technical interventions." Is the statement in the Results section incorrect?

We apologize for our error here. The statement in the result section was corrected as; Unexpected and unexplained technical failures occasionally occurred during the trial period.

4. It is not clear to me what happened if there was transmission failure in the control group. (Intervention group received breakthrough calls, but not the control group). The paragraph on technical failures does not differentiate by group; how were technical problems dealt with in the control group? Does this account for the difference in duration of transmission failure per subject/day (Table 2)?

As we did not monitor the frequency of BP measurements in the control group, mechanical failure data were retrospectively collected on the regular clinic visit. We asked all the study subjects at study enrollment, both intensive management and control groups, to bring the BP measurement device back for their regular clinic visit. When transmission failure or any errors in the device occurred, all the BP measurements stored in the device were downloaded at the clinic.

We elaborated it in the method section as following;

All the subjects were asked to bring back the sphygmomanometer to the regular clinic visit. (...) When data transmission failure occurred in the control group, stored BP measurements during the failure period were downloaded directly from the device.

5. It seems like the issue of non-portability is another limitation that should be discussed, as it could discourage potential subjects from wanting to participate. Having to record things manually while away from home would be prone to error.

We do agree to the Reviewer's point here. However, other than estimating BP using arterial tonometry, the mainstay of modern BP monitoring requires pneumatic pump which physically make BP monitors cumbersome.

This point was added to the discussion section as following;

The non-portability of BP monitors may discourage potential participants and manual

recording of BP out of home would be prone to errors.

6. I feel that the authors have focused the discussion section largely on their conclusion that they have proved feasibility; however, I wonder whether feasibility has really been proven. Based on the evidence presented, the authors have overstated the conclusions (i.e., proven feasibility) of conducting such a study on a larger scale. As the authors themselves state, "securing reliable connection…would be a key component. However, we have experienced unexpected technical obstacles…the smartphone application experienced one major upgrade and 72 minor modifications. Future trials need to consider the instability of such system." This does not sound like a system that is "ready for prime time." A more appropriate conclusion would be that, as designed, the system has not been perfected to the point that it did not require intense monitoring and adjustment. Therefore, the system itself would certainly need to be perfected before any larger scale trials could be considered. The authors conclude that BP control in the intensive management group did not improve over that in the control group. However, the performance from a technical standpoint WAS improved by intensive management. While the conclusion of "no difference" between intense and no management may be true for BP control, isn't the more important point that intense management was needed to make the system work accurately at all? In the interest of performing future trials, I think that it would have been much more helpful if the discussion were focused on what didn't work, and why, and how to fix it, rather than emphasizing the positive features of the trial and concluding that feasibility had been proven. There is a lot of valuable and important information here, but I don't think the authors have presented it in the most accurate or helpful light.

We do appreciate the Reviewer's thoughtful advice and considerate sentences for the conclusion. We totally accept the Reviewer #3's point and modified our conclusion as suggested;

The current phase II randomized clinical trial was able to prove the feasibility and safety of the wireless home BP measurement, transfer, storage and monitoring system and behavioral intervention to improve BP measurement and control among acute ischemic stroke patients. Technical consideration is required to establish secure connection for BP data transmission dependent on a smartphone. We also documented that the telemedicine system still requires human interface to overcome technical failures and sustain behavioral changes for motivation. The telehealth system, as applied in the current trial, has not been perfected to the point that it did not require intense monitoring and adjustment by human. Therefore, although the feasibility of the telehealth system in BP control of stroke patients has been documented, the system itself would certainly need to be perfected before any larger scale trials could be considered.

Additionally, we added a paragraph to the discussion section to discuss our lessons from the current trial for future researches.

Several practical points are worthy of discussion for future trials or researches. Future implementation of a similar remote system should comply with the local regulations in the security of data storage and transmission. The data server in this trial had

physically separated from the hospital information system. Smartphones are widely used, but the elderly usually confronts with many difficulties in using and manipulating smartphones. Interfaces between smartphones and wireless healthcare devices, including blood pressure monitors, are still too difficult for them. To overcome the issue of "IT literacy," a consumer-friendly user interface design such as iHealth Labs will be helpful.

Minor comments:

• While the individual BP plots are beautiful and impressive, they really aren't needed here, and take the focus away from the more important problems the investigators faced.

The reviewer's point here was duly noted. However, as for the data transparency of a clinical trial, we have intentionally provided the graph in the online supplements. We do respect the Reviewer #3's point, but may we have the permission to provide the individual BP plot as in the current form?

• The authors describe response to breakthrough times as "immediate"; however, in 4/7 cases, the response was delayed by 3 or more days, which in a short trial, could result in a sizeable loss of data. Again, be more realistic here; it is more helpful to look at the individual data and discuss where problems existed and why, than to make a judgment based on median delay which is indeed short.

We appreciate Reviewer #3 to let us clarify our trial method. By "immediate," we asked the trial subjects to visit outpatient clinics on the next business day. Inevitable delay has intervened due to weekend or public holiday.

In this sense, we modified the following sentence;

issued a breakthrough call on the sustained failure of the data transfer to visit outpatient clinics on the next business day.

• The percentage of half-day blocks reported for the intensive management group in Table 2 has a 95% confidence interval of 60% to 112%. This strongly implies that the data are not normally distributed and should not be summarized with a mean and sd or compared to a mean "success criteria." A more meaningful standard would be to set the success criteria at or above a certain percentile (75th? 90th?), and evaluate the data with non-parametrics.

• This is also the case for duration of transmission failures. Please use non-parametric standards.

We do appreciate Reviewer #3 here and we modified the Table 2 accordingly.

Overall, this is a very nice piece of work. Obviously, a lot of time was spent summarizing a great deal of data quite beautifully. I think the study is very interesting, but I do feel like the authors failed in the discussion to really address the more interesting and more important aspects of their trial in sufficient detail. There were definitely some major problems in terms

of feasibility, and the discussion would be much more valuable to future researchers (and realistic) if the authors addressed these. Information on why something ISN'T feasible is just as important (if not more so) than a (questionable) conclusion that it is.

We appreciate the reviewer's positive evaluation of our study and hope the current revision would suffice the reviewer’s requests.
