# Supplementary material for: Remote blood pressure monitoring and behavioral intensification for stroke: A randomized controlled feasibility trial
Source: PLoS One. 2020 Mar 11;15(3):e0229483. doi: 10.1371/journal.pone.0229483 (PMC7065804; doi:10.1371/journal.pone.0229483)
Supplement: Attachment — Submitted filename: BOSS Trial_PLoS One_27_Response to Reviewers.pdf [file pone.0229483.s019.pdf]

Rebuttal to the reviewers' comment

PLoS One
Manuscript ID, PONE-D-19-21291R1
Manuscript title: Remote Blood Pressure Monitoring And Behavioral Intensification for Stroke: A Randomized Controlled Feasibility Trial
Lead author: Beom Joon Kim
Corresponding Author: Hee-Joon Bae

January 29th, 2019

Dear reviewers and editorial staffs of PLoS One;

We would like to thank the reviewers and editor for their positive evaluation and obliging suggestion on our manuscript. We provide responses to the reviewers' comments below. Responses will follow the verbatim of reviewers' comments (blue color). *Additionally, we modified several sentences to make our point clearer. All the changes were specified in the marked version of our draft.*

Reviewer #3;

I thank the authors for their careful consideration of my comments and the manner in which they have been addressed. This is a small point, but I still feel that the conclusion as stated in the abstract and discussion (and conclusion) should be tempered to reflect that the authors have not proven feasibility with the tested system, as it is "not feasible" to give patients a device that requires so much maintenance. Perhaps a more cautious conclusion would be that intensive monitoring in itself was feasible, insofar as patients complied with the intervention, but that the device utilized would need to be improved prior to a larger trial. Also, the paper still needs some work grammatically.

Editorial staffs;

Specifically, I think Reviewer 3 makes a valid point about overstating your conclusion in both the abstract and main paper and you may think about using their suggested wording instead. The phrasing and grammar of the text would also benefit from attention and you may wish to get a native English speaker to revise it. Therefore, we invite you to submit a revised version of the manuscript that addresses these points.

> *We appreciate the reviewer #3's considerate suggestion on the conclusion and perspectives of our study results. After contemplation and along with the editor's advices, we concluded both abstract and main text with your wording.*

Conclusion section of the abstract;

The intensive monitoring, including remote BP measurement, data transfer, and centralized monitoring system, engaged with behavioral intensification was feasible if the patients complied with the intervention. However, the device utilized would need further improvement prior to a large trial.

Conclusion section of the main text;

This phase-II randomized clinical trial proved the feasibility and safety of the intensive monitoring system, comprising of wireless home BP measurement, transfer, storage, and monitoring system and behavioral intervention, to improve BP measurement and control in acute ischemic stroke patients. However, technical considerations are important to establish secure connection for BP data transmission dependent on a smartphone. We also documented that the tele-medicine system still requires human interface to overcome technical failures and sustain behavioral changes for motivation. The tele-health system, as applied in the current trial, has not been perfected to the point that it did not require intense monitoring and adjustment by humans. Therefore, although the feasibility of the tele-health system in BP control of stroke patients has been documented and even if patients complied with the intervention, the system itself would certainly need to be perfected before any larger scale trials are considered.

The manuscript has been revised by a professional English editing service and the proof is attached as a supplemental file not intended to publish. Corrected grammars and phrases were not highlighted to focus on the topic. The manuscript style was also modified as for the journal style.
